# Synergistic catalysis: *cis*-cyclopropanation of benzoxazoles†
†Electronic supplementary information (ESI) available. CCDC 1060573. For ESI and crystallographic data in CIF or other electronic format see DOI: 10.1039/c5sc03597j



**DOI:** 10.1039/c5sc03597j

**Published:** 2015-10-30

**Authors:** Marta Meazza, Mark E. Light, Andrea Mazzanti, Ramon Rios

**Affiliations:** a Faculty of Natural & Environmental Sciences , University of Southampton , Highfield Campus , Southampton , SO17 1BJ , UK . Email: R.Rios-Torres@Southampton.ac.UK ; http://www.riosresearchgroup.com; b Department of Industrial Chemistry “Toso Montanari” , School of Science , University of Bologna , Viale Risorgimento 4 , 40136 Bologna , Italy

## Abstract

In the present paper we report our latest efforts in pushing the boundaries of synergistic catalysis. We propose the use of 3 different catalytic cycles working in concert for the formation of *cis* cyclopropane derivatives of benzoxazoles with excellent stereoselectivities. This is the proof of concept that synergistic catalysis could be successfully used in cascade reactions.

## Abstract 2

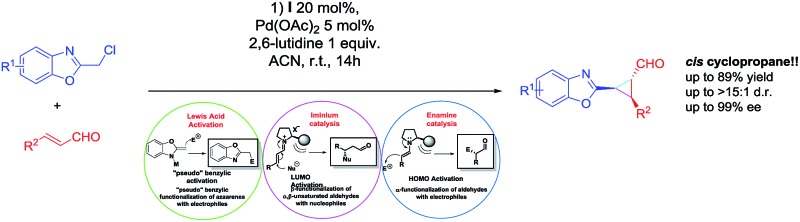
3 catalytic cycles working in concert to generate *cis* cyclopropanes in good yields and stereoselectivities!

## Introduction

In the last years huge efforts have been made to develop new activation modes in order to synthesize complex scaffolds in an enantiopure form. The most common approach has been the development of new catalysts to activate an “unreactive” compound A that will react with a highly reactive compound B to render C. However, recently a new approach pushes the boundaries of organic chemistry: synergistic catalysis, where the concurrent activation of both reactants allows the use of both unreactive substrates to form a new C–C bond. Earlier examples of synergistic catalysis merging transition metals and organocatalysts can be found in the pioneering works of Cordova who reported the α-allylic alkylation of unactivated aldehydes with allyl acetates by using both enamine and Pd chemistry.^1^ Later, Zhang reported an efficient asymmetric variant by using Cordova's synergistic strategy and Pd catalysts with C_2_-symmetrical chiral metallocene ligands.^2^ MacMillan and Sibi reported examples of asymmetric synergistic catalysis based on enamine catalysis and metal-bound electrophiles.^3^

On the other hand, synergistic catalysis with α,β-unsaturated carbonyl compounds has attracted much recent interest. The works of Cordova in β-silylation, β-arylation or β-borylation by using iminium and transition-metal activation demonstrated the importance of this approach.^4^ In this area, List also reported excellent results for allylation and Overman rearrangement reactions by using phosphoric acid derivatives and transition-metal catalysts.^5^ Very recently, our research group became interested in synergistic catalysis.^6^

**Fig. 1:**
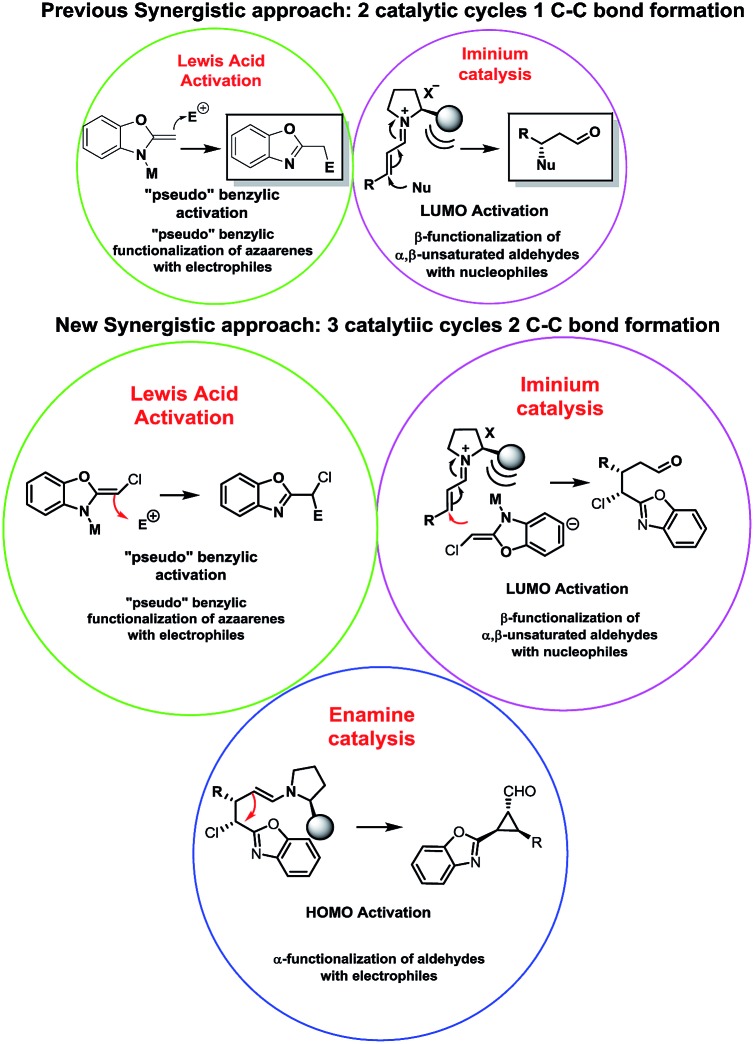
Proposed catalytic cycles.

Our research focuses on the development of methodologies for the stereoselective synthesis of alkyl benzoxazoles based on the concurrent activation of alkyl benzoxazoles by metal-based Lewis acid catalysis (inspired by the pioneering work of Lam)^7^ and organocatalytic activation of several electrophiles.^8^ It is well known that one of the most important concerns of this approach is the possible autoquenching of both catalysts. However, by careful choice of the catalysts and the reaction conditions, we successfully developed the addition of alkylbenzoxazoles to Morita–Baylis–Hillman (MBH) carbonates and enals in good yields and stereoselectivities (Fig. 1).

Despite significant progress and the advantages of using two unreactive substrates, synergistic catalysis presents a clear limitation in terms of range and scope: all these reactions generate only 1 C–C bond, making this approach more expensive and contrary to atom economy (2 catalyst for only 1 bond formation). This drawback attracted our interest and we propose to overcome this limitation by a carefully designed cascade reaction based on a synergistic approach. In our earlier examples we successfully combined metal Lewis activation of azaarenes with iminium catalysis, with good results but with some limitations such as moderate enantioselectivities and low diastereoselectivities due to an epimerization process.^6*b*^

We thought that a good option to develop a cascade reaction based on a synergistic approach and, at the same time, to limit the drawbacks of our previous work, should be the design of an organocascade reaction that could trap the enamine “*in situ*”, rendering a more complex scaffold with high stereoselectivity using the same catalytic system in order to push the boundaries of synergistic catalysis by forming 2 C–C bonds using 3 catalytic cycles.

Due to our experience in organocascade reactions, we envisioned that a cyclopropanation using chloro-alkylbenzoxazoles and enals will fulfil the requirements as a proof of concept of this approach and at the same time will allow us to synthesize an important scaffold in organic chemistry.

Cyclopropanes^9^ are structural motifs widely present in naturally occurring compounds, including terpenes, fatty acid metabolites, pheromones, *etc.*,^10^ and in pharmaceutical compounds with a large spectrum of biological properties.^11^ Cyclopropanes are also useful as synthetic intermediates that can undergo ring-opening or ring-expansion reactions to form a range of functionalized products.^12^

Asymmetric cyclopropanation reactions have fascinated organic chemists for decades and exciting advances have recently been reported; however, the development of new methodologies for the stereoselective synthesis of cyclopropanes is still a major challenge. In the literature, several approaches for their enantioselective synthesis are reported, for example, the venerable Simmons–Smith cyclopropanation,^13^ organometallic methodologies that use Cu, Rh and other transition-metal complexes^14^ or, with the advent of organocatalysis, several Michael-initiated ring-closing domino reactions, *etc.* ^15^

## Results and discussion

**Fig. 2:**
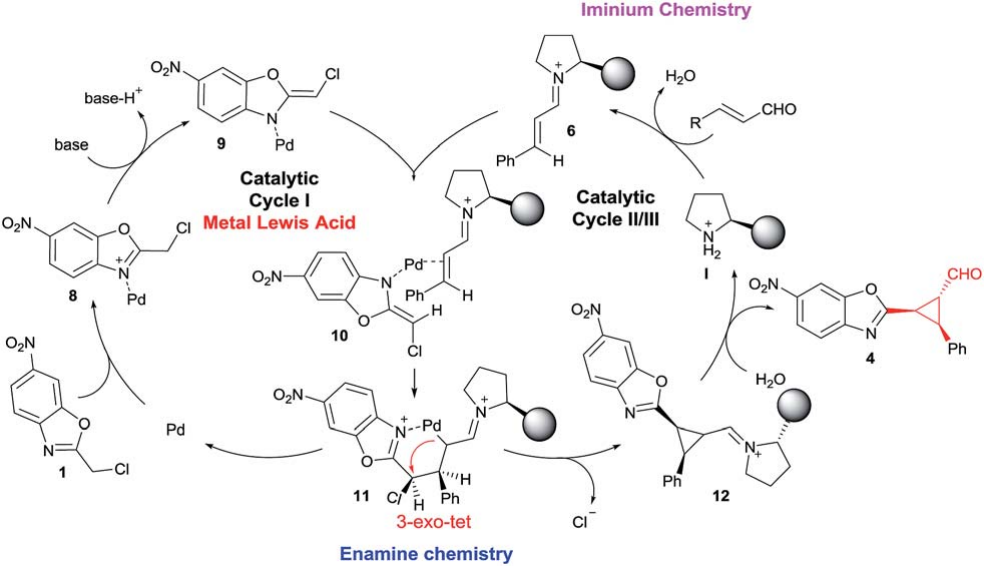
Proposed mechanism.

To show the viability of the present reaction initially we studied the addition of chloro-alkylbenzoxazole **1a** with cinnamaldehyde. The proposed synergistic mechanism is shown in Fig. 2.

As shown in Fig. 2, we envisioned that the metallic Lewis acid would interact with the alkylbenzoxazole **1** by coordinating to the nitrogen atom of **1** and increasing the acidity of the proton in the methylene position. The co-catalyzed mechanism involves the stereoselective addition of palladium enolate (**9**) to chiral iminium intermediate **6**, followed by an intramolecular 3-*exo*-tet cyclization. As suggested by Lam,^7^ at first a coordination of the palladium to the benzoxazole occurs, followed by deprotonation by the external base leading to species **9**. In parallel, iminium intermediate **6** is generated *in situ* by reactions of enals **2** and chiral amine catalyst **I**. Next a coordination between the Pd enolate and the double bond of the imine (as Cordova proved in similar synergistic Pd/secondary amine catalysed reactions) takes place to form the intermediate **10**. Some stereochemical issues must be discussed here: first the coordination of the Pd to the double bond must be by the *Si* face of the iminium due the efficient shielding of the *Re* face of **6** by the bulky chiral group of **I**; in the case of the Pd enolate **9** the azaallyl ligand possesses *E*-stereochemistry to minimize steric interaction (as Lam suggested using other substituents) between the Cl substituent and the iminium. In this step the stereochemistry of the process is established: *Si*-facial nucleophilic attack at the β-carbon takes place, forming the 6 membered cycle C-bound-Pd intermediate **11**. Next, a fast intramolecular alkylation between the new Pd enolate formed in α position of the iminium and the methylene chloride with inversion of configuration leads to the product **12** with the *cis* configuration between the aryl ring and the benzoxazole. Hydrolysis of **12** affords **4** and releases the catalysts, thus completing the catalytic cycle.

After a short screening we found that the best conditions were MeCN as a solvent, Pd(OAc)_2_ as a Lewis acid, Jorgensen–Hayashi catalyst (**I**) as secondary amine and 2,6-lutidine as stoichiometric base. To our delight the reaction rendered the final cyclopropane rings in excellent yields and stereoselectivities. The study of NMR spectra confirmed that the major diastereomer obtained in the cyclopropanation reaction has a *cis* disposition between the aryl ring and the benzoxazole substituent (see ESI†). This result adds, without any doubt, an important value to the present methodology. The synthesis of *cis* di(hetero)aromatic cyclopropanes *via* an intermolecular reaction is very challenging. This unusual *cis* configuration is difficult to obtain by any other enantioselective methodology that forms the C–C bond bearing both aryl groups. Only Mezzetti^16^ and Katzuki^17^ using ruthenium and iridium catalyst got the *cis* compound as a major diastereomer with excellent results in the reaction of styrenes with diazoacetates. Formally, the methodology reported in this paper is stereocomplementary with the MacMillan cyclopropanation that renders trisubstituted cyclopropanes in a *trans* configuration.^18^

**Scheme 1:**
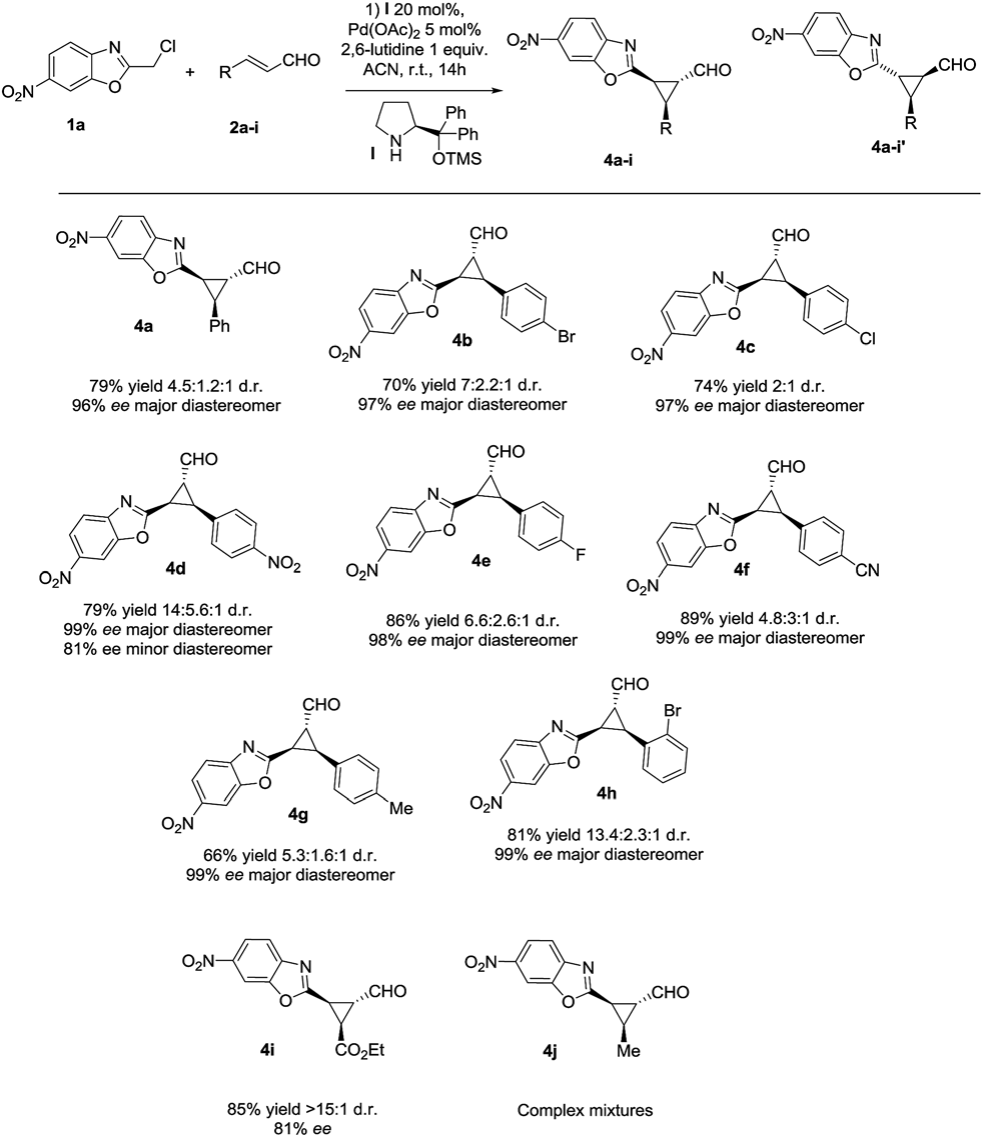
Scope of the reaction with several enals (d.r. determined by ^1^H NMR of the crude mixture, ee determined by chiral HPLC analysis of the isolated product).

With the optimized reaction conditions in hand, we investigated the substrate scope of the reaction with respect to enal derivatives. As shown in Scheme 1, the reactions afford the corresponding formyl cyclopropanes in good to excellent yields with moderate to good diastereoselectivities and excellent enantioselectivities in the major diastereomer. Remarkably, in all the examples the major diastereomer could be easily isolated by column chromatography while the other two diastereomers observed, usually are non-separable mixtures. All the diastereomeric ratios are from the final isolated products.

The reaction is compatible with several substituents in the aromatic ring. Electron-withdrawing groups like 4-NO_2_ (**4d**) or 4-CN (**4f**) render the final cyclopropane in good yields (79% and 63%), good diastereoselectivities and excellent enantioselectivities. Several halides were also tested in the reaction giving similar results, for example 4-Br (**4b**), 4-Cl (**4c**) and 4-F (**4e**) render the predicted formyl cyclopropane in good yields and diastereoselectivities (86–70% yield and up to 3 : 1 d.r.) and exceptional enantioselectivities (up to 99% ee). When the most sterically demanding 2-Br cinnamaldehyde derivative was used, the formyl cyclopropane (**4h**) was obtained with good yields and excellent enantioselectivity in the major diastereomer but with lower diastereoselectivity. To our delight, when we tested the enal derived from glyoxylate, the reaction rendered the cyclopropane (**4i**) with good yield and excellent diastereoselectivity (only one diastereomer) but lower enantioselectivity. This could be due to an epimerization at C3 position of the product during the reaction.

**Fig. 3:**
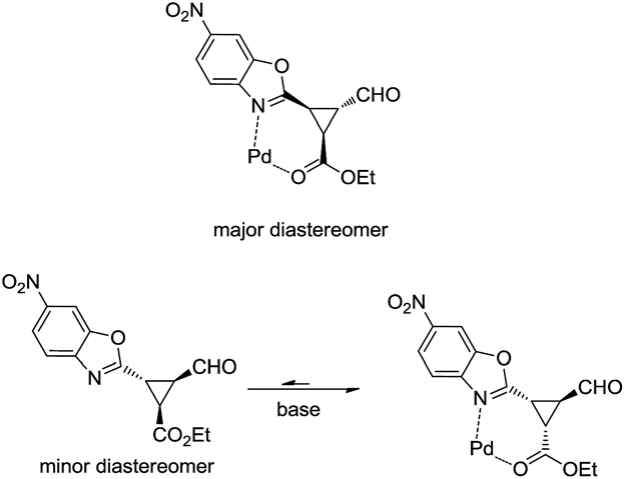
Proposed mechanism for epimerization of **4i**.

As shown in Fig. 3, when an ester moiety is placed in C3, this proton becomes quite acidic and could undergo epimerization with the base to form the most stable *trans* diastereomer, which would be stabilized by a coordination of the metal with the nitrogen from benzoxazole and the carbonyl compound from the ester. If this epimerization occurs, the outcome of the reaction will be one diastereomer with moderate enantioselectivity as observed by us. Finally, the only limitation of the present methodology is with the aliphatic enals that gave complex crude mixtures when subjected to the reaction conditions.

**Fig. 4:**
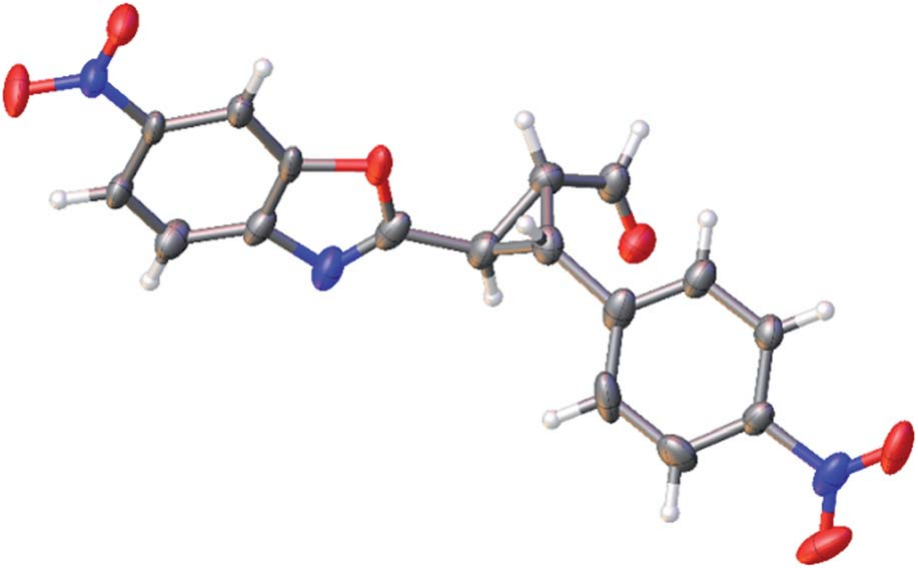
X-ray structure of compound **4d′** (minor diastereomer). The displacement ellipsoids are drawn at the 50% probability level.^20^

The relative configuration of the minor diastereomer was confirmed by X-ray crystallography (Fig. 4), while the relative configuration of the major diastereomer was inferred by NMR spectroscopy (see ESI†).

**Fig. 5:**
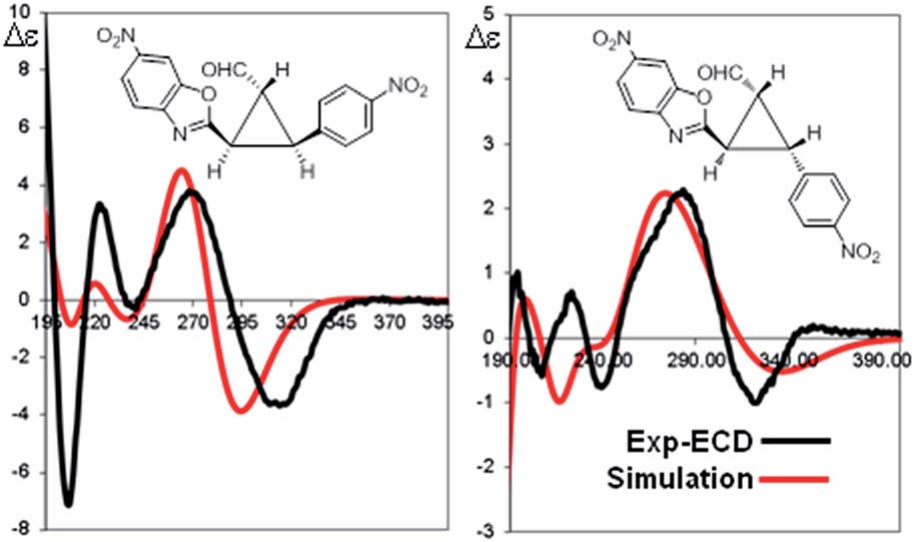
TDDFT simulation of the ECD spectra for the two diastereomers of **4d** [(*S*)-catalyst] and **4d′** [(*R*)-catalyst]. Shown simulations were obtained with TD-DFT at the M06-2X/6-311++G(2d,p) level of theory.

The absolute configuration of the two diastereomers was determined to be 1*R*,2*R*,3*S* for the major diastereomer **4d** (using (*S*)-**I** as catalyst) and 1*R*,2*R*,3*R* for the minor diastereomer **4d′** (using (*R*)-**I** as catalyst) by TD-DFT simulation of the Electronic Circular Dichroism (ECD) spectra (Fig. 5 and ESI†).^19^ This absolute configuration and diastereoselectivity of the major diastereomer is in agreement with the mechanism proposed and with the previous works done with this type of catalysts, where the stereochemistry at β-position of the enal is perfectly controlled by catalyst (**I**).^15^ The relative and absolute configuration of the minor diastereomer could be rationalized by the reaction with the (*Z*)-palladium enolate.

**Scheme 2:**
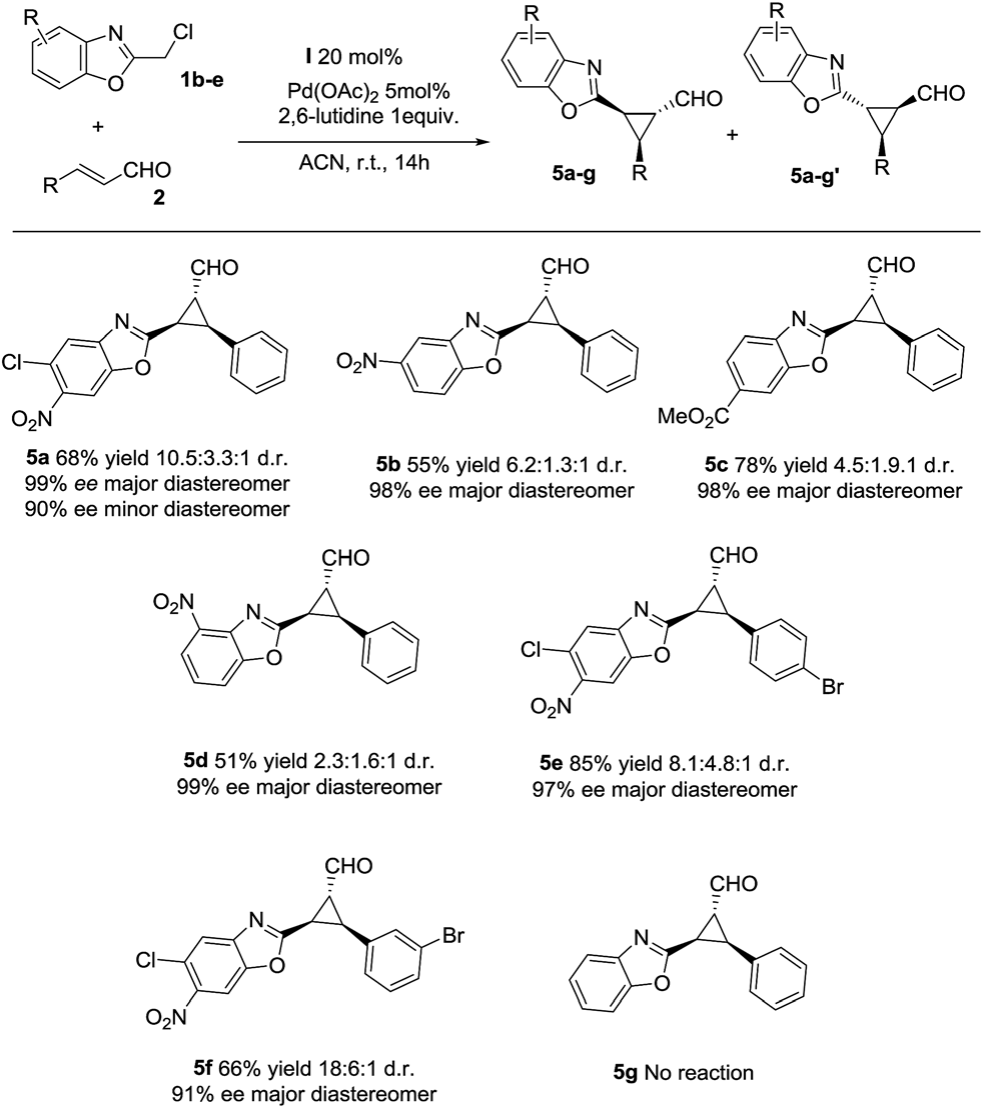
Scope of the reaction with several azaarenes (d.r. determined by ^1^H NMR of the crude mixture, ee determined by chiral HPLC analysis of the isolated product).

Next, we investigated the substrate scope of the reaction with respect to the benzoxazole derivative **1**. Several benzoxazole derivatives were tested (Scheme 2) and the presence of electron-withdrawing groups in the azaarene ring was crucial for the reactivity. Cl-substituted benzoxazole rendered the final product **5a**, **5e** and **5f** with good yields, good stereoselectivities and excellent enantioselectivities. When the benzoxazole had a –NO_2_ group at a different position (**5b** and **5d**), slightly lower yields were observed but good diastereoselectivities and excellent enantioselectivities were obtained. Finally, changing the electron-withdrawing –NO_2_ group to an ester group rendered the final product **5c** in excellent yield, good diastereoselectivity and excellent enantioselectivity. The only limitation, as previously noted in our previous reports on synergistic catalysis,^6a,b^ is that an electron-withdrawing group must be present on the benzoxazole ring. Probably, the electron-withdrawing group decreases the p*K*_a_ value of **1** and allows the deprotonation and the subsequent reaction.

## Conclusions

In summary, we have demonstrated that synergistic catalysis can be applied to the development of a cascade reaction by developing a highly enantioselective cyclopropanation of enals with chloro-alkylbenzoxazoles. For the first time, the metal activation of azaarenes was combined with an organocatalytic cascade reaction, pushing the boundaries of this synergistic approach. Two different catalytic cycles, (i) the metallic Lewis acid activation of azaarenes and (ii) the secondary amine activation of enals, worked in accord to afford the final products in good yields, with excellent enantioselectivity and good diastereoselectivity. Moreover this reaction gives access to *cis* cyclopropanes that are difficult to obtain by any other method. The mechanistic studies, synthetic applications and development of new organocascade reactions based on this concept are currently ongoing in our laboratory.
