# Supplementary material for: Synergistic catalysis: cis-cyclopropanation of benzoxazoles
Source: Chem Sci. 2015 Oct 30;7(2):984–8. doi: 10.1039/c5sc03597j (PMC5633913; doi:10.1039/c5sc03597j)
Supplement: Supplementary file 1 [file SC-007-C5SC03597J-s001.pdf]

This journal is © The Royal Society of Chemistry 2015

1. Contents

2. Crystal Data and Experimental 4d' minor diastereomer obtained with (*R*)-catalyst 2

3. Conformational analysis and absolute configuration 4

4. Screening of Solvents 25

5. Screening of Organic Catalyst 26

6. Screening of Bases 27

7. Screening of Lewis Acid 28

8. Screening of Temperatures 29

9. Screening of Metals with 2,6-lutidine 29

10. Scope of the reaction - aldehydes 30

11. Scope of the reaction – benzoxazoles 31

12. Synthesis of the starting material – benzoxazoles 32

13. Synthesis of the starting material – α,β-unsaturated aldehydes 35

14. General procedure for the synthesis of cyclopropanes 36

15. Final products characterisation 36

16. Notes: 61

17. References 61

18. HPLC traces 62

19. NMR starting materials 87

20. NMR cyclopropanes 94

2. Crystal Data and Experimental 4d' minor diastereomer obtained with (*R*)-catalyst

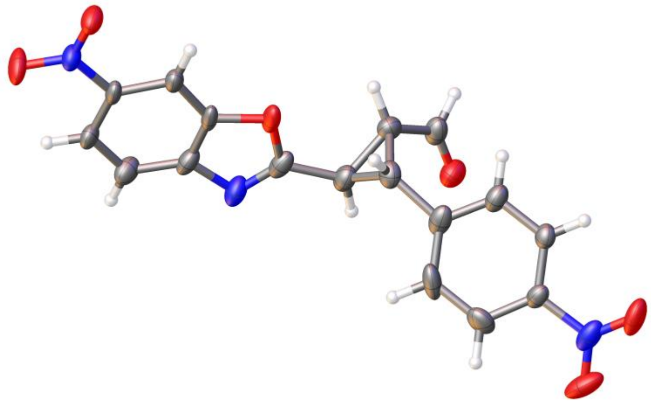

Figure 1. Thermal ellipsoids drawn at the 50 percent probability level.

Experimental. Single clear colourless fragment-shaped crystals of (**2014sot0046**) were recrystallised from a mixture of TCM and hexane by slow evaporation. A suitable crystal (0.09 × 0.08 × 0.05 mm^3) was selected and mounted on a MITIGEN holder in perfluoroether oil on a Rigaku AFC12 FRE-HF diffractometer. The crystal was kept at T = 100(2) K during data collection. Using **Olex2** (Dolomanov et al., 2009), the structure was solved with the ShelXT (Sheldrick, 2008) structure solution program, using the Direct Methods solution method. The model was refined with version of **ShelXL** (Sheldrick, 2008) using Least Squares minimisation.

Crystal Data. $C_{17}H_{11}N_3O_6$, M_r = 353.29, monoclinic, P2$_1$/c (No. 14), a = 9.9877(5) Å, b = 15.1541(7) Å, c = 10.2131(6) Å, β = 103.161(5)$^\circ$, $\alpha = \gamma = 90^\circ$, V = 1505.20(14) Å^3, T = 100(2) K, Z = 4, Z' = 1, μ (MoK$_\alpha$) = 0.121, 14536 reflections measured, 3884 unique (R_{int} = 0.0709) which were used in all calculations. The final wR_2 was 0.3428 (all data) and R_1 was 0.1503 (I > 2(I)).

**Compound**	**2014sot0046**
Formula	$C_{17}H_{11}N_3O_6$
$D_{calc.}$/ g cm^{-3}	1.559
μ/mm^{-1}	0.121
Formula Weight	353.29
Colour	clear colourless
Shape	fragment
Max Size/mm	0.09
Mid Size/mm	0.08
Min Size/mm	0.05
T/K	100(2)
Crystal System	monoclinic
Space Group	P2$_1$/c
a/Å	9.9877(5)
b/Å	15.1541(7)
c/Å	10.2131(6)
$\alpha/^\circ$	90
$\beta/^\circ$	103.161(5)
$\gamma/^\circ$	90
V/Å^3	1505.20(14)
Z	4
Z'	1
$\Theta_{min}/^\circ$	3.380
$\Theta_{max}/^\circ$	28.699
Measured Refl.	14536
Independent Refl.	3884
Reflections Used	3045
R_{int}	0.0709
Parameters	235
Restraints	0
Largest Peak	1.092
Deepest Hole	-0.552
GooF	1.080
wR_2 (all data)	0.3428
wR_2	0.3307
R_1 (all data)	0.1725
R_1	0.1503

Experimental Extended. A clear colourless fragment-shaped crystal with dimensions 0.09 × 0.08 × 0.05 mm^3 was mounted on a MITIGEN holder in perfluoroether oil. Data were collected using a Rigaku AFC12 FRE-HF diffractometer equipped with an Oxford Cryostream low-temperature apparatus operating at T = 100(2) K.

Data were measured using profile data from ω-scans of 1.0° per frame for 15.0 s using MoK_α radiation (Rotating Anode, 45.0 kV, 55.0 mA). The total number of runs and images was based on the strategy calculation from the program **CrystalClear** (Rigaku). The actually achieve resolution was Θ = 28.699.

Cell parameters were retrieved using the **CrysAlisPro** (Agilent, V1.171.37.31, 2014) software and refined using **CrysAlisPro** (Agilent, V1.171.37.31, 2014) on 6234 reflections, 43 of the observed reflections.

Data reduction was performed using the **CrysAlisPro** (Agilent, V1.171.37.31, 2014) software which corrects for Lorentz polarisation. The final completeness is 99.60 out to 28.699 in Θ. The absorption coefficient (MU) of this material is 0.121 and the minimum and maximum transmissions are 0.56971 and 1.00000.

The structure was solved by Direct Methods using the ShelXT (Sheldrick, 2008) structure solution program and refined by Least Squares using version of **ShelXL** (Sheldrick, 2008).

The structure was solved in the space group $P2_1/c$ (# 14). All non-hydrogen atoms were refined anisotropically. Hydrogens positions were calculated geometrically and refined using the riding model.

There is no entry for the cif item _refine_special_details.

Citations

CrysAlisPro Software System, Agilent Technologies UK Ltd, Yarnton, Oxford, UK (2014).

CrystalClear, Rigaku.

O.V. Dolomanov and L.J. Bourhis and R.J. Gildea and J.A.K. Howard and H. Puschmann, Olex2: A complete structure solution, refinement and analysis program, *J. Appl. Cryst.*, (2009), **42**, 339-341.

Sheldrick, G.M., A short history of ShelX, *Acta Cryst.*, (2008), **A64**, 339-341.

3. Conformational analysis and absolute configuration

All the attempts to obtain good enantiopure crystals of the prepared compounds were not successful. For this reason the relative and absolute configuration was determined by a combination of conformational analysis and theoretical simulations of chiro-optical spectra. Compound **4d** was selected as representative compound because good racemic crystals were obtained for the minor diastereomer (**4d-minor**). X-ray data allowed the determination of the relative configuration of the three stereogenic centres of the cyclopropane ring as *R**,*R**,*R**.

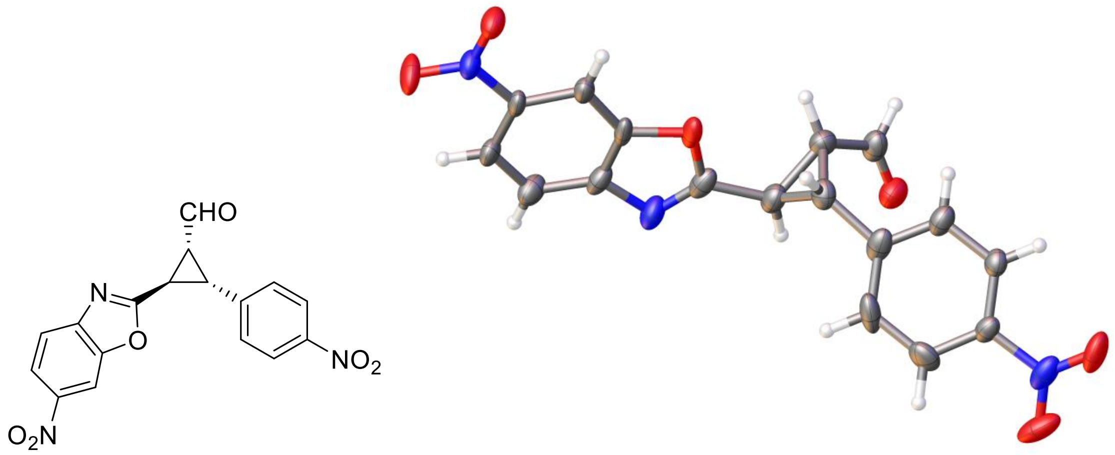

Figure S1. X-ray structure of racemic **4d-minor.**

Although the rigidity of the cyclopropane core reduces the number of conformations to be considered,[1] two conformational degrees of freedom due to the rotation of the aldehyde and of the benzoxazole moiety must be considered for the conformational analysis step.

The whole conformational space was explored by means of Monte Carlo searching together with the MMFF94 molecular mechanics force field as implemented in Titan 1.0.5 (Wavefunction inc.)

All the conformations found by MM search within a 10 kcal/mol window were then optimized using DFT at the B3LYP/6-31+G(d,p) level using the Gaussian 09 suite of

[1] P.L. Polavarapu, E.A. Donahue, G. Shanmugam, G. Scalmani, E.K. Hawkins, C. Rizzo, I. Ibnusaud, G. Thomas, D. Habeland, and D.Sebastian, *J. Phys. Chem. A* 2011, **115**, 5665–5673

programs[2]. The harmonic vibrational frequencies of each optimized conformation were calculated at the same level to confirm their stability (no imaginary frequencies were observed) and to evaluate the ZPE corrected enthalpy and free energy of each conformation. After DFT minimization, four conformations were found to be enclosed in a 1 kcal/mol window as shown in Figure S2 and marked as **a-d** in Table S1 and Table S2.

Table S1. Relative energies of the four conformations of **4d-minor** evaluated using ZPE-corrected enthalpies and different optimization levels: B3LYP/6-31+G(d,p) and M06-2X/6-31+G(d,p). Populations are calculated using Boltzmann distribution at 298°K.

Conformation	H° (B3LYP)	H° (M06-2X)	Pop. (B3LYP)	Pop. (M06-2X)
**a**	0.74	1.25	14	8
**b**	0.48	0.88	21	14
**c**	0.63	0.89	17	14
**d**	0.00	0.00	48	64

Table S2. Relative energies of the four conformations of **4d-minor** evaluated using ZPE-corrected Gibbs free energies and different optimization levels: B3LYP/6-31+G(d,p) and M06-2X/6-31+G(d,p). Populations are calculated using Boltzmann distribution at 298°K.

Conformation	G° (B3LYP)	G° (M06-2X)	Pop. (B3LYP)	Pop. (M06-2X)
**a**	0.47	0.61	18	16
**b**	0.08	0.28	35	29
**c**	0.94	1.00	8	9
**d**	0.00	0.00	39	46

[2] Program Gaussian 09, rev D.01. M. J. Frisch, G. W. Trucks, H. B. Schlegel, G. E. Scuseria, M. A. Robb, J. R. Cheeseman, G. Scalmani, V. Barone, B. Mennucci, G. A. Petersson, H. Nakatsuji, M. Caricato, X. Li, H. P. Hratchian, A. F. Izmaylov, J. Bloino, G. Zheng, J. L. Sonnenberg, M. Hada, M. Ehara, K. Toyota, R. Fukuda, J. Hasegawa, M. Ishida, T. Nakajima, Y. Honda, O. Kitao, H. Nakai, T. Vreven, J. A. Montgomery, Jr., J. E. Peralta, F. Ogliaro, M. Bearpark, J. J. Heyd, E. Brothers, K. N. Kudin, V. N. Staroverov, R. Kobayashi, J. Normand, K. Raghavachari, A. Rendell, J. C. Burant, S. S. Iyengar, J. Tomasi, M. Cossi, N. Rega, J. M. Millam, M. Klene, J. E. Knox, J. B. Cross, V. Bakken, C. Adamo, J. Jaramillo, R. Gomperts, R. E. Stratmann, O. Yazyev, A. J. Austin, R. Cammi, C. Pomelli, J. W. Ochterski, R. L. Martin, K. Morokuma, V. G. Zakrzewski, G. A. Voth, P. Salvador, J. J. Dannenberg, S. Dapprich, A. D. Daniels, Ö. Farkas, J. B. Foresman, J. V. Ortiz, J. Cioslowski, and D. J. Fox, Gaussian, Inc., Wallingford CT, 2009.

As predictable, the four conformations correspond to the four different relative dispositions of the CHO and benzoxazole group, and the relative energies (both as ZPE-corrected enthalpies or Gibbs free energies) suggested that all these conformations should be appreciably populated. To check whether a different theoretical level provided different results, the four ground states were optimized again at the M06-2X/6-31+G(d,p) level with similar results in terms of relative energies. Conformation **d** was always the most stable, albeit it does not correspond to that observed in the solid state, that is conformation **c**. In addition to that, the dihedral angle between the plane of the *p*-nitrophenyl ring and the cyclopropane plane calculated for conformations **c** and **d** is rather different with respect to that observed in the solid state. However, both the calculation levels yield the same results and the different dihedral angle observed in the X-ray structure could be the result of crystal lattice stabilization. Indeed, when the geometry read from X-ray data was used as input to DFT optimization, the *p*-nitrophenyl ring was rotated to provide again conformation **c**.

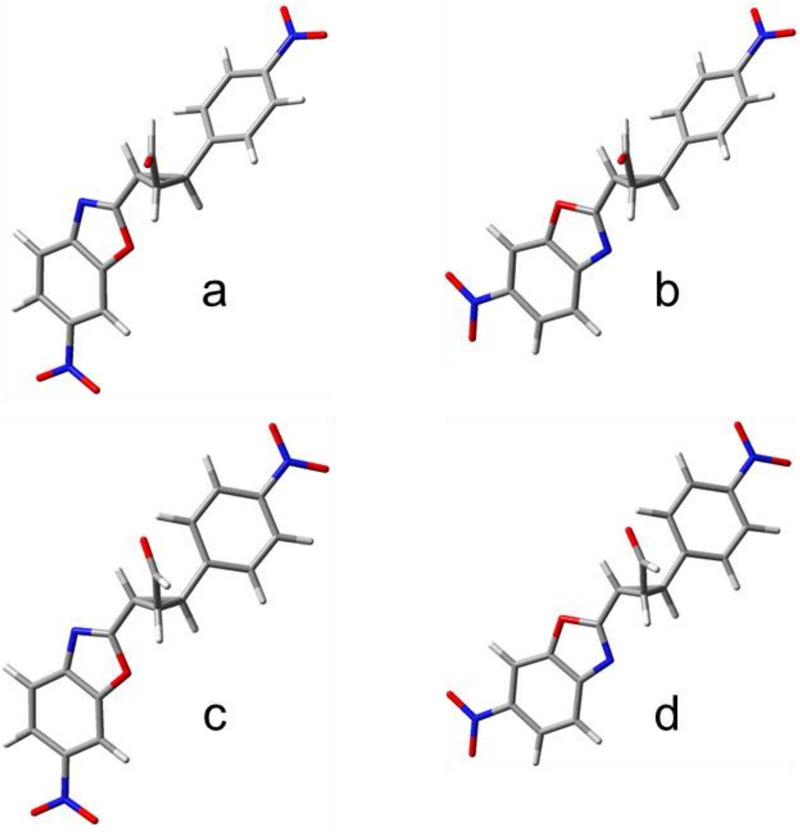

Figure S2. 3D view of the four conformations of the model compound **4d-minor.**

Absolute configuration

The determination of the absolute configuration (AC) of chiral molecules using chiroptical techniques like optical rotation (OR), electronic circular dichroism (ECD), and vibrational circular dichroism (VCD) has gained feasibility and reliability because of the development of methods for the prediction of these properties based on density functional theory (DFT) and on its Time-Dependent formalism (TD-DFT).[3] In the present case the theoretical calculation of the electronic circular dichroism spectra (ECD) was selected for the absolute configuration assignment because of the presence of good UV chromophores. The ECD spectrum of **4d-minor (obtained with (*R*)-catalyst)** was acquired in HPLC-grade acetonitrile solution ($6 \cdot 10^{-5}$ M) with a cell path of 0.5 cm in the 190-400 nm region by the sum of 16 scans at 50 nm/min scan rate (Figure S3). Albeit rather weak, the experimental ECD spectrum exhibits three negative Cotton effects at 321, 238 and 206 nm, a broad positive branch at 265-290 nm, as well as two weak positive branches at 222 and 194 nm.

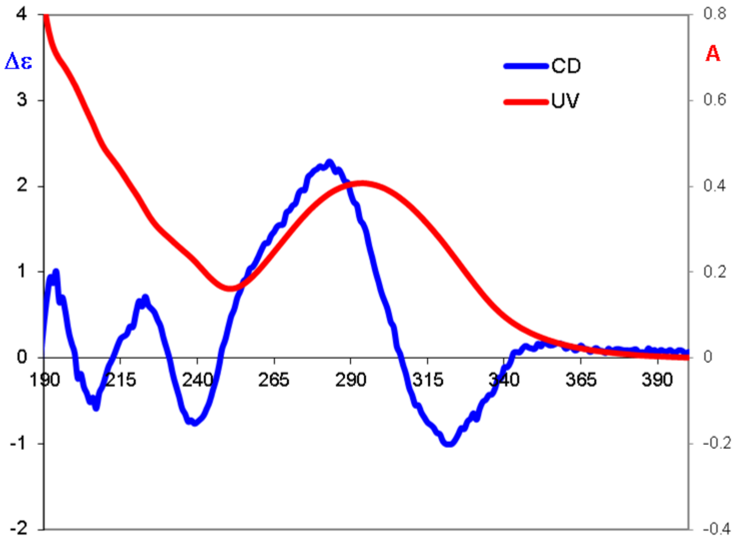

Figure S3: ECD (blue trace) and UV (red trace) spectra of **4d-minor** **(*R*)-catalyst**. Spectra were recorded in acetonitrile, $6 \cdot 10^{-5}$ M, 0.5 cm cell path.

[3]For reviews see: a) G.Bringmann, T. Bruhn, K. Maksimenka, and Y. Hemberger, *Eur. J. Org. Chem*. 2009, 2717-2727.b)T.D. Crawford, M.C. Tam, and M.L. Abrams,*J. Chem. Phys. A* 2007, **111**,12057–12068. c) G. Pescitelli, L. Di Bari and N. Berova, *Chem.Soc.Rev.* 2011, **40**, 4603-4625.For a review on conformational analysis for the absolute configuration determination see: A. Mazzanti, and D. Casarini, D. *WIRESComput. Mol. Sci.*2012, **2**, 613-641

The electronic excitation energies and rotational strengths have been calculated for the isolated molecule in the gas phase for the four conformations **a-d** using TD-DFT. In a preliminary test, two different basis sets (6-311++G(2d,p) and def2-TZVPP) were employed to calculate the ECD spectrum of conformation **d** using the CAM-B3LYP functional[4] and the two geometries provided by the B3LYP and M06-2X optimization steps. The results are reported in Figure S4, showing that the basis sets and input geometries did not influence the calculated ECD spectrum at a great extent.

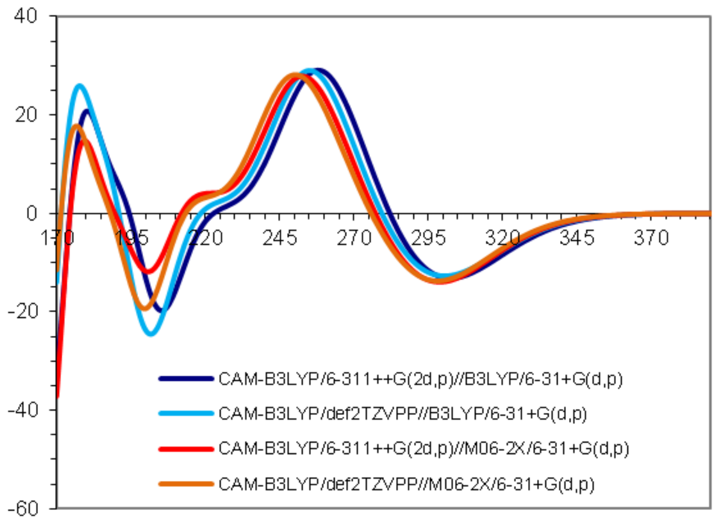

Figure S4.TD-DFT simulated spectra calculated for conformation **d** of **4d-minor** using the same CAM-B3LYP functional, two different basis sets (6-311++G(2d,p) and def2TZVPP) and two different input geometries form B3LYP/6-31+G(d,p) and M06-2X/6-31+G(d,p) optimization. For each calculation the first 60 excited states were calculated, and the spectrum was obtained using a 0.30 eV line width at half height.

The ECD spectra of the four conformations were calculated with four different methods (functionals), to ascertain if different computational approaches provide different shapes of the simulated spectra (Figure S5).[5]

[4] T. Yanai, D. Tewand, and N.Handy, *Chem. Phys. Lett.*2004, **393**, 51-57.
[5]C.E.Check and T.M.Gilbert. *J.Org.Chem.* 2005, **70**, 9828-9834

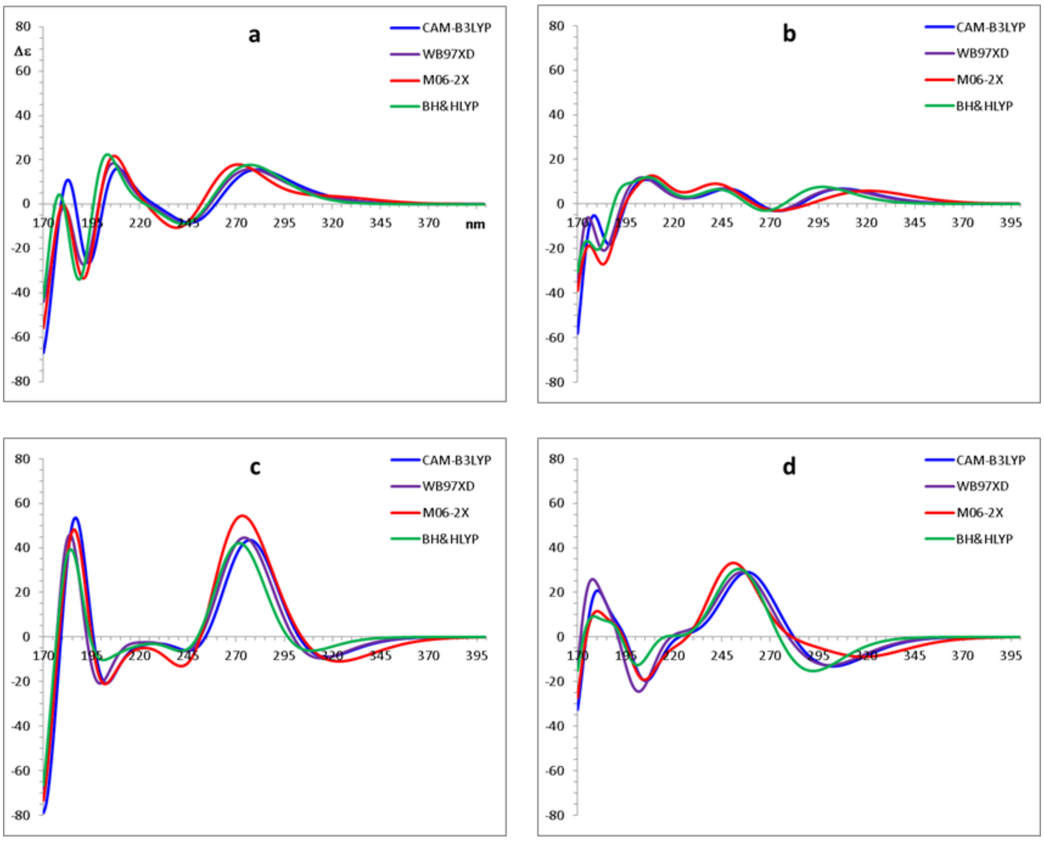

Figure S5.TD-DFT simulated spectra calculated for the four conformations of **4d-minor** using four different functionals (CAM-B3LYP, BH&HLYP, M06-2X, ωB97-XD) and the same 6-311++G(2d,p) basis set. For each conformation the first 60 excited states were calculated, and the spectrum was obtained using a 0.30 eV line width at half height.

Simulations were performed using the B3LYP-optimized geometries with the hybrid functionals BH&HLYP[6] and M06-2X,[7] ωB97XD that includes empirical dispersion,[8] and CAM-B3LYP that includes long range correction using the Coulomb Attenuating Method. Given the result of the preliminary tests, The calculations employed the B3LYP-optimized geometries and the 6-311++G(2d,p) basis set, that is computationally cheaper than def2TZVPP, still providing good accuracy.[9] The rotational strengths were calculated in both

[6] In Gaussian 09 the BH&HLYP functional has the form: $0.5{*}E_X^{HF} + 0.5{*}E_X^{LSDA} + 0.5{*}\Delta E_X^{Becke88} + E_C^{LYP}$
[7]Y. Zhao and D.G. Truhlar, *Theor. Chem. Acc*. 2008, **120**, 215-241.
[8] J-D. Chai and M. Head-Gordon, *Phys. Chem. Chem. Phys.*, 2008, **10**, 6615-6620.
[9] a) P. Gunasekaran, S. Perumal, J. Carlos Menéndez, M. Mancinelli, S. Ranieri, A. Mazzanti, *J. Org. Chem.* **2014**, *79*, 11039–11050. b) L. Caruana, M. Fochi, M. Comes Franchini, S. Ranieri, A. Mazzanti, L. Bernardi,

length and velocity representation, the resulting values being very similar (RMS difference < 5%). Errors due to basis set incompleteness should be therefore very small.[10]

Although the spectra simulated within the same functional for the four conformation are quite different, they are nevertheless consistent with the simulation of the positive Cotton effect in the 245-270 nm region(Figure S5). This part of the UV spectrum is dominated by the two UV transitions of the *p*-nitrophenyl moiety (oriented on the long axis) and of the 5-nitrobenzoxazole moiety. The almost coincidence of the simulated spectra for the same conformation on varying the functional represent a good proof of the simulations consistency.

The population-weighted spectra to be compared with the experimental spectrum were obtained using the percentages derived from ZPE corrected enthalpies (Table S1). As shown in Figure S6, the spectra simulated assuming 1*R,*2*R,3R* absolute configuration match well the Cotton effects at 321, 283 nm. The best simulation was obtained by the ωB97-XD functional, but all the simulated spectra show a good agreement with the experimental one.

Chem. Commun. **2014**, *50*, 445-447. c) M. Ambrogi, A. Ciogli, M. Mancinelli, S. Ranieri, A. Mazzanti, *J. Org. Chem.* **2013**, *78*, 3709-3719. d) L. Caruana, M. Fochi, S. Ranieri, A. Mazzanti, L. Bernardi, *Chem. Commun.* **2013**, *49*, 880-882. e) X. Companyo, A. Mazzanti, A. Moyano, A. Janecka, R. Rios, *Chem. Commun.* **2013**, *49*, 1184-1186. f) G. Cera, M. Chiarucci, A. Mazzanti, M. Mancinelli, M. Bandini, *Org. Lett.* **2012**, *14*, 1350-1353.
[10]P.J. Stephens, D.M. McCann, F.J. Devlin, J.R. Cheeseman and M.J. Frisch, *J.Am.Chem.Soc.*2004, **126**, 7514-7521

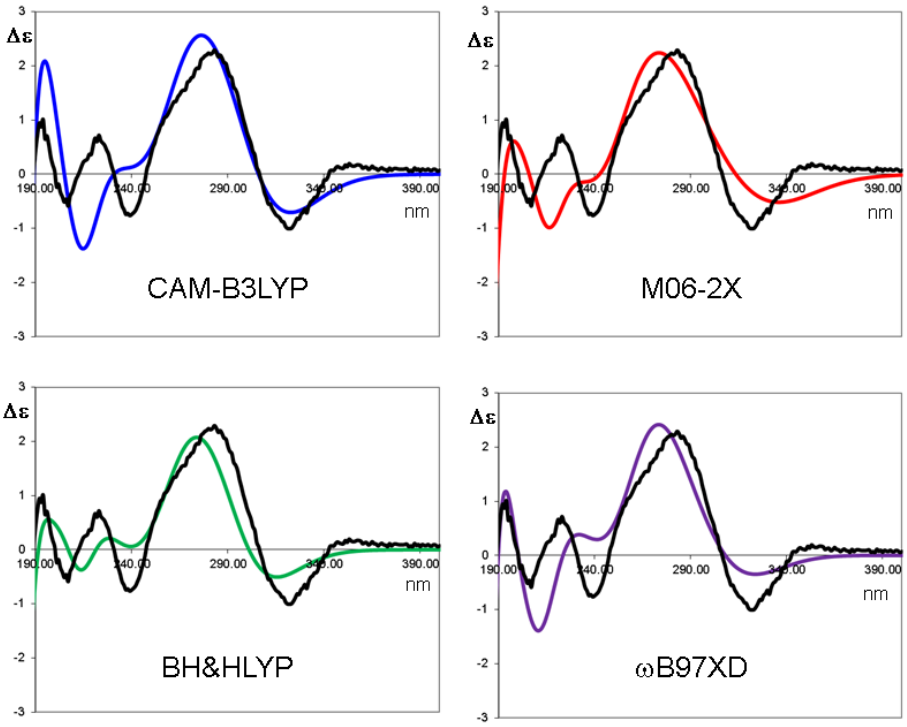

Figure S6: Simulations of the experimental ECD spectrum of **4d-minor (obtained with (*R*)-catalyst)**. For each quadrant, the black line correspond to the experimental spectrum. The colored lines correspond to the simulations obtained using the populations derived from B3LYP/6-31+G(d,p) optimization. The simulated spectra were vertically scaled and red-shifted by 7-14 nm to get the best match with the experimental spectrum. All the simulations are for the 1*R*,2*R*,3*R* absolute configuration.

Major diastereomer

Good single crystals of the major diastereomer could not be obtained and the assignment of the relative configuration was determined by NMR. The ^{1}H and ^{13}C signals were assigned by means of 2D-NMR experiments (COSY, HSQC and HMBC), and NOE spectra were acquired using the DPFGSE sequence.[11] In the case of the major isomer of **4d (obtained with (*S*)-catalyst)** (**4d-major**), the ^{1}H signals of the hydrogens of cyclopropane were heavily overlapped in a variety of solvent ($CDCl_3$, DMSO, CD_3CN), and the compound was not chemically stable in CD_3OD. More resolved spectra were obtained for the parent compound **4a**, that exhibited a resolved ^{1}H spectrum in CD_3CN (Figure S7). A close inspection of the ^{1}H spectrum provided useful information about the relative disposition of the three hydrogens of the cyclopropane ring (named as H1, H2 and H3 in Figure S7).

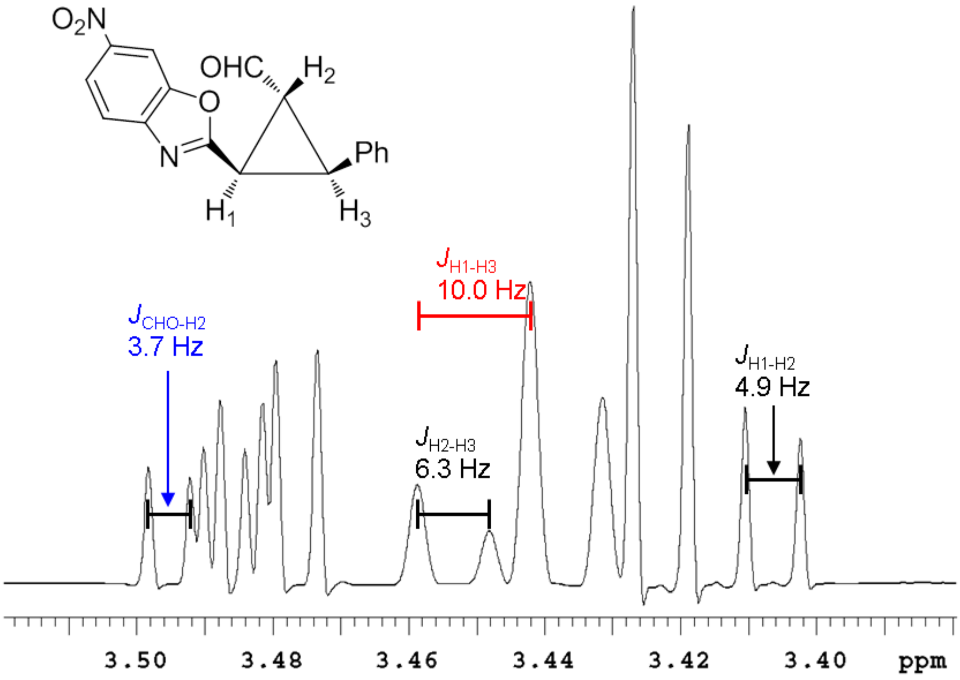

Figure S7. ^{1}H spectrum of the aliphatic region of **4a-major** (600 MHz, CD_3CN).

[11] Stonehouse, J.; Adell, P.; Keeler, J.; Shaka, A. J. *J .Am. Chem. Soc.* **1994**, *116*, 6037–6038.

The two vicinal ^3J coupling constants H2-H1 and H2-H3 have similar values (4.9 and 6.3 Hz, respectively), while the H1-H3 coupling constant between the two CH bearing the aromatic rings is rather large (10.0 Hz). The large value of the latter suggests that the dihedral angle between the two hydrogens is close to 0° (thus a *syn* relationship of the aromatic rings), while the smaller coupling constants of H1 and H3 with H2 are a clear indication of a gauche disposition of H2 with respect to H1 and H3 (thus a *trans* relationship of the hydrogens).

As a confirm, the ^{1}H spectrum of **4d-minor** showed a similar set of rate constants, but the large one (9.9 Hz) took place between H2 and H3 (see Table S3).

To have further support to the assignment based on coupling constant, DFT calculations were run to calculate the coupling constants values of the major isomer supposing the 1*R**,2*R**,3*S** relative configuration. Due to the rigidity of cyclopropane, the relative disposition of the key hydrogens of the stereogenic centres are fixed independently from the different conformations of the CHO and benzoxazole, and the values of the coupling constants can elucidate the relative stereochemistry.[12] Before to run the NMR simulations, a conformational search was run by means of Monte Carlo searching together with the MMFF94 molecular mechanics force field. All the conformations found by MM search were then optimized using DFT at the B3LYP/6-31+G(d,p) level and their stability was checked by vibrational analysis. As for **4d**-**minor**, four conformations were found to be enclosed in a 2 kcal/mol window as shown in Figure S8 and marked as **a-d** in Table S3. Again, the four conformations correspond to the four different relative dispositions of the CHO and benzoxazole group.

[12] J. D. Graham, and M. T. Rogers *J. Am. Chem. Soc.*, **1962**, *84*, pp 2249–2252

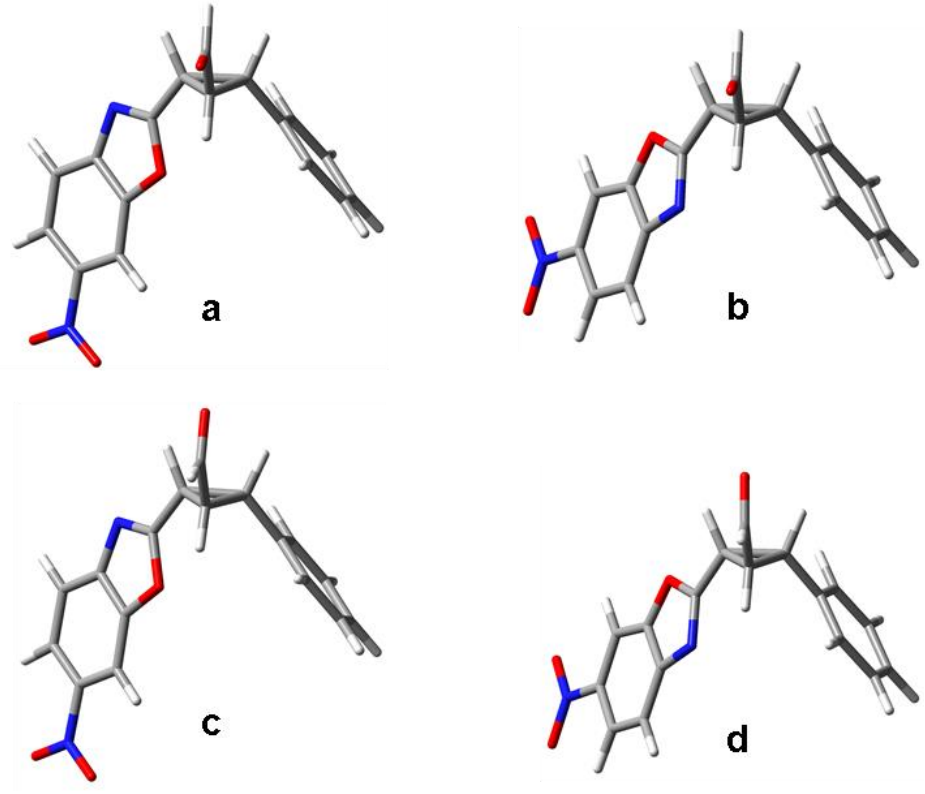

Figure S8. Geometries of the four conformations of **4a-major**.

Table S3. Relative energies of the four conformations of **4a-major** evaluated using ZPE-corrected enthalpies and different optimization levels: B3LYP/6-31+G(d,p) and M06-2X/6-31+G(d,p). Populations are calculated using Boltzmann distribution at 298°K.

Conformation	H° (B3LYP)	H° (M06-2X)	Pop. (B3LYP)	Pop. (M06-2X)
**a**	0.58	0.97	15	9
**b**	0.73	1.37	12	4
**c**	0.00	0.00	41	44
**d**	0.17	0.01	31	43

The simulations of the coupling constants were run at the at the B3LYP/6-311++G(2d,p) level using the GIAO method and including the Fermi contact term (Gaussian 09 keyword: spinspin, mixed). The calculated coupling constants for the 1*R**,2*R**,3*S** relative

configuration (Table S4) are in a very good agreement with the experimental values of **4a-major**.

Table S4. Calculated and experimental coupling constants for the four diastereoisomers of **4d**. Calculations were run at the GIAO-B3LYP/6-311++G(2d,p)//B3LYP/6-31+G(d,p) level. In parenthesis are reported the calculated *J*-couplings of the conformations in which the H2-C-(CO)-H dihedral is close to 180°. In italics are reported the calculated values for those conformations in which the H1-C1-Cq-O ≈ 180°. Plain text values are relative to values for those conformations in which the H1-C1-Cq-O ≈ 0°.

^3J	Calcd. for (1*R**,2*S**,3*S**)	Calcd. for (1*R**,2*S**,3*R**)	Calcd. for (1*R**,2*R**,3*S**)	Calcd. for **4d-minor**[a] (1*R**,2*R**,3*R**)	Expl. **4a-major**	Expl. **4d-minor**
H2-CHO	2.0 (6.9)<br>*1.9 (6.5)*	1.8 (6.7)<br>*1.7 (6.9)*	1.7 (6.9)<br>*1.7 (6.9)*	1.5 (7.6)<br>*1.5 (7.3)*	3.7	3.9
H2-H1	9.5 (10.0)<br>*9.0 (9.6)*	9.7 (10.1)<br>*9.9 (10.4)*	4.9 (5.1)<br>*4.7 (5.1)*	5.5 (4.2)<br>*5.7 (4.8)*	4.9	4.9
H2-H3	10.8 (10.4)<br>*10.1 (10.4)*	5.4 (7.2)<br>*5.5 (6.8)*	6.4 (6.9)<br>*6.2 (6.6)*	11.3 (11.2)<br>*11.1 (11.2)*	6.3	9.9
H1-H3	10.3(10.5)<br>*10.7 (10.7)*	7.2 (6.7)<br>*7.6 (7.2)*	11.2 (11.3)<br>*11.2 (11.3)*	7.4 (6.7)<br>*7.9 (6.9)*	10.0	6.6

---

[a] relative configuration from X-Ray data

As a check of the calculation reliability, the coupling constants were calculated also for **4d-minor**, which relative configuration was known from X-ray data. Also in this case the calculated values fully matched the experimental values. It should be noted that in both compounds the experimental value of the H2-CHO coupling constant clearly results from the weighted average of two conformations where the dihedral angle H2-C-(CO)-H is close to 0° or 180° (see below). The resulting experimental value seems to suggest that both the two conformations are populated roughly at the same extent. The full set of coupling constants were calculated also for the remaining two diastereomers due to inversion at C2 carbon

(Table S4, columns 2 and 3). For both cases the set of calculated couplings does not match the experimental data, thus confirming the previous assignment of the (1*R**,2*R**,3*S**) relative configuration to **4a-major**.

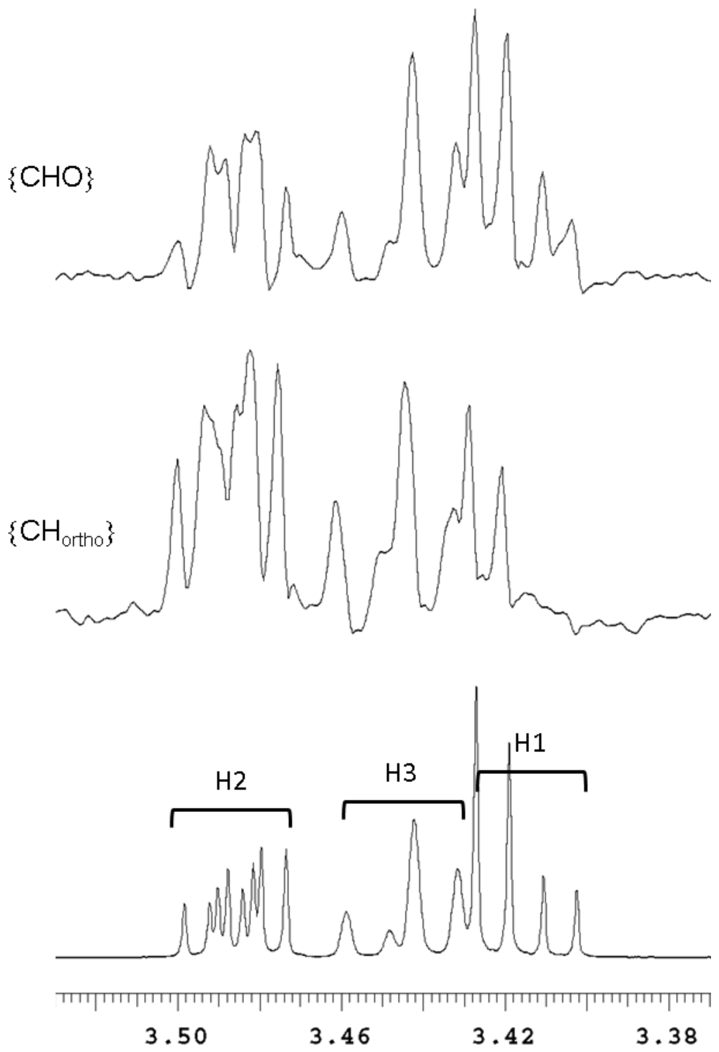

Figure S9. DPFGSE NOE spectra of **4a-major** (600 MHz in CD_3CN). Bottom: control spectrum. Middle trace: NOE obtained on saturation of the *ortho*-phenyl signal. Top trace: NOE obtained on saturation of the CHO signal.

NOE spectra were recorded to further confirm the relative configuration of the major diastereomer of **4a**. These spectra, however, were thwarted by the distance constraints imposed by the cyclopropanic ring and by the partial overlapping of the key signals (H1, H2 and H3). On saturation of the CHO signal (Figure S9), comparable NOEs were observed for

H1 and H3, while on saturation of the *ortho* hydrogens of the phenyl ring the NOE on H2 is larger than that on H3 and H1. These results again confirm the relative configuration previously assigned by the coupling constants analysis.

Absolute configuration of 4d-major

For coherence with the first assignment, the absolute configuration of the major diastereomer was performed on compound **4d-Major (obtained with (*S*)-catalyst)**. The ECD spectrum was acquired in HPLC-grade acetonitrile solution ($1 \cdot 10^{-4}$ M) with a cell path of 0.2 cm in the 195-400 nm region by the sum of 16 scans at 50 nm/min scan rate (Figure S10). The spectrum of **4d-major** is similar to the of the minor isomer, but the relative intensities of the Cotton effects are different. In this case the two branches at 310 and 270 nm seem to generate a weak exciton coupling and that at 245 nm is much more weaker that the corresponding one of the minor isomer.

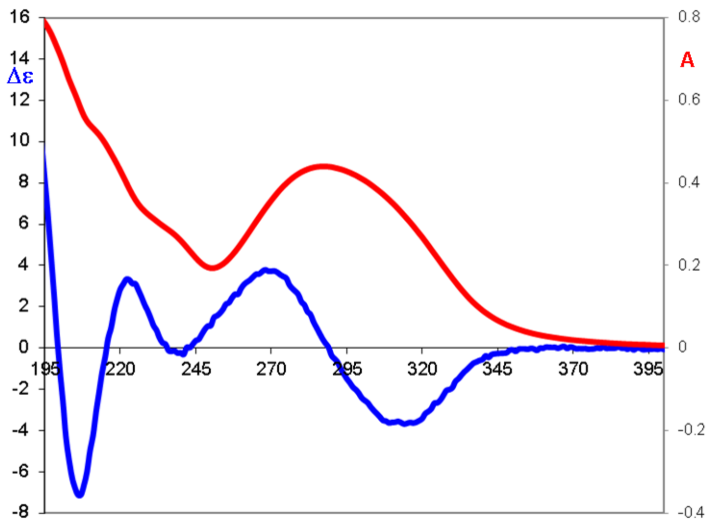

Figure S10. ECD (blue trace) and UV (red trace) spectra of **4d-major (obtained with (*S*)-catalyst)**. Spectra were recorded in acetonitrile, $1 \cdot 10^{-4}$M, 0.2 cm cell path.

The four stable conformations of **4d-major** were again optimized at the B3LYP/6-31+G(d,p) level starting from the geometries obtained for **4a-major**. Calculations were run in the gas-phase and including two different solvents (chloroform and acetonitrile) using the PCM method.[13] The relative energies and corresponding populations derived from Boltzmann statistics are reported in Table S5.

The electronic excitation energies and rotational strengths have been calculated for the

[13] J. Tomasi, B. Mennucci, and R. Cammi, *Chem. Rev.* 2005, **105**, 2999-3093.

isolated molecule in the gas phase with the four different methods (functionals) already employed for the simulation of the ECD spectrum of the minor diastereomer (CAM-B3LYP, BH&HLYP, M06-2X, and ωB97XD). In analogy with **4d-minor**, TD-DFT calculations employed the 6-311++G(2d,p) basis set, yielding the results reported in Figure S11.

Table S5. Relative energies of the four conformations of **4d-major** evaluated using ZPE-corrected enthalpies obtained from B3LYP/6-31+G(d,p) optimizations in gas phase and including two different solvents ($CHCl_3$ and CH_3CN) with the PCM method. Populations are calculated using Boltzmann distribution at 298°K.

Conf.	gas phase	PCM($CHCl_3$)	PCM(CH_3CN)	Pop%	Pop%	Pop%
**a**	1.46	0.20	0.00	5	23	33
**b**	1.12	0.18	0.08	11	22	28
**c**	0.79	0.21	0.25	17	23	22
**d**	0.00	0.00	0.37	67	32	17

Within the same conformation, the four kind of calculations provide very similar traces. However, at a variance with the minor isomer, the simulated spectra for conformations **a** and **d** are nearly opposite to that simulated for **b** and **c**. The two pairs of conformations showing opposite spectra are different because of the ≈ 180° rotation of the benzoxazole ring. A rationale of the opposite calculated traces can be found in a close analysis of the different disposition of the *p*-nitrophenyl ring and benzoxazole in the two conformations. (Figure S12).

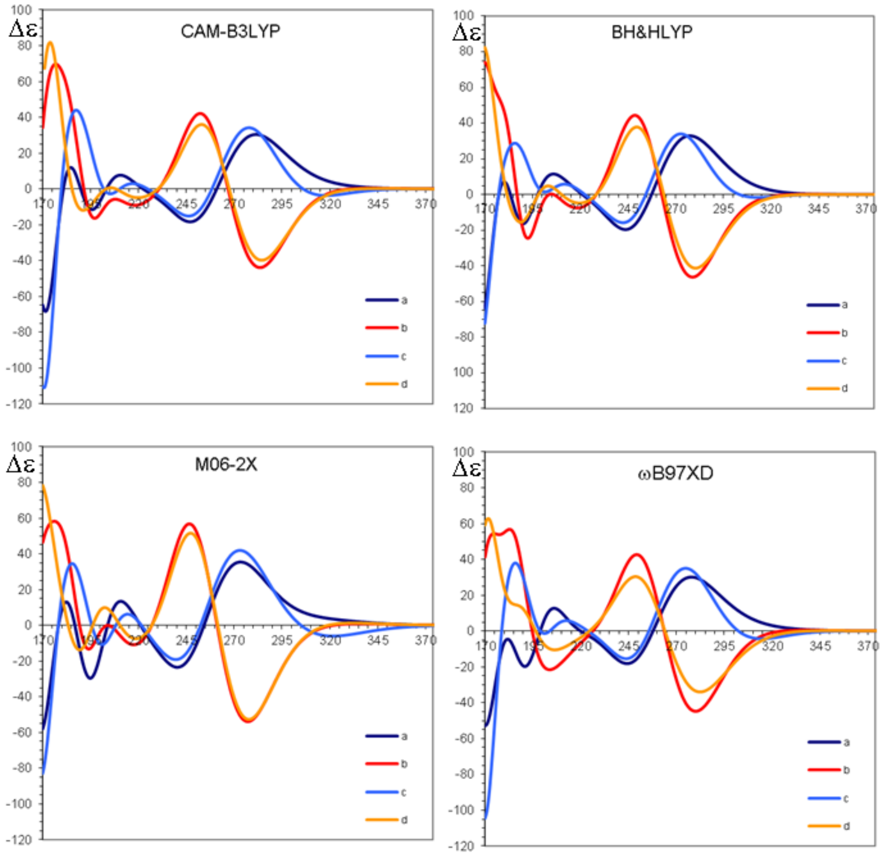

Figure S11. Calculated ECD for each conformation of **4d-major** with different functionals and the same 6-311++G(2d,p) basis set.

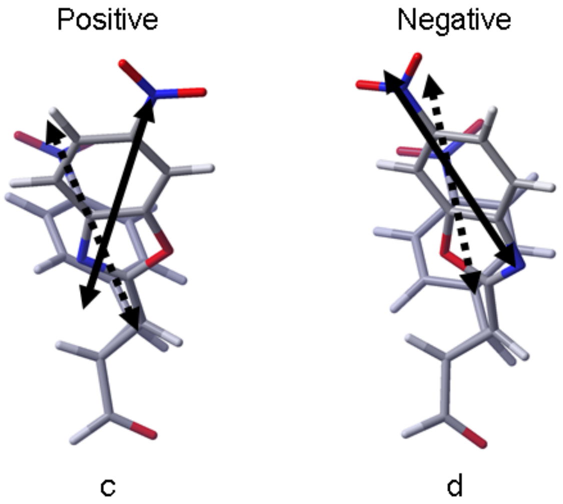

Figure S12: View of the dipoles of **4d-major** acting in the generation of the UV spectrum in the 250-350 nm region. In both conformations the *p*-nitrophenyl ring is far away from the observer and the benzoxazole is close. The dotted arrows correspond to the UV transition of the *p*-nitrophenyl ring oriented along the long axis, while the full arrow are that of benzoxazole.

The dihedral angles generated by the two dipoles of along the long axes of *p*-nitrophenyl and benzoxazole in the two conformations yield opposite sign, thus explaining the opposite exciton coupling in the simulations.

Being the ECD spectrum the weighted average of the spectra of the four conformations, the correct ratio to be used is crucial to the success of the ECD simulation (in the following discussion only conformations **c** and **d** will be considered since the spectrum of the second conformation of each pair due to CHO rotation is identical). In similar cases[14,15] the conformational ratio could be evaluated by Dynamic NMR or NOE experiments, but in the present situation this approach is thwarted by the absence of any benzoxazole hydrogen in the closeness of the cylopropanic ring. To overcome this difficulties, a carefully degassed $CDCl_3$ NMR sample was prepared in order to extend the effective radius of the NOE effect. $CDCl_3$ was selected as solvent because of its low viscosity that allows longer T1 relaxation times. In $CDCl_3$ the two signals of the two CH of cyclopropane bearing the aromatic rings (H1 and H3) are exactly overlapped and yield a doublet, whereas the CH(CHO) signal is a triplet of doublets due to the coupling with the two isochronous CH of cyclopropane and with the CHO. DPFGSE NOE spectra were acquired using long mixing times (4-6 s) corresponding to the T1 relaxation time of the cyclopropanic hydrogens measured at ambient temperature by the inversion-recovery sequence (Figure S13).

[14] E. Paradisi, P. Righi, A. Mazzanti, S. Ranieri, and G. Bencivenni. *Chem. Commun.* 2012, **48**, 11178-11180.
[15] M. Ambrogi, A. Ciogli, M. Mancinelli, S. Ranieri, and A. Mazzanti. *J.Org.Chem.* 2013, **78**, 3709-3719

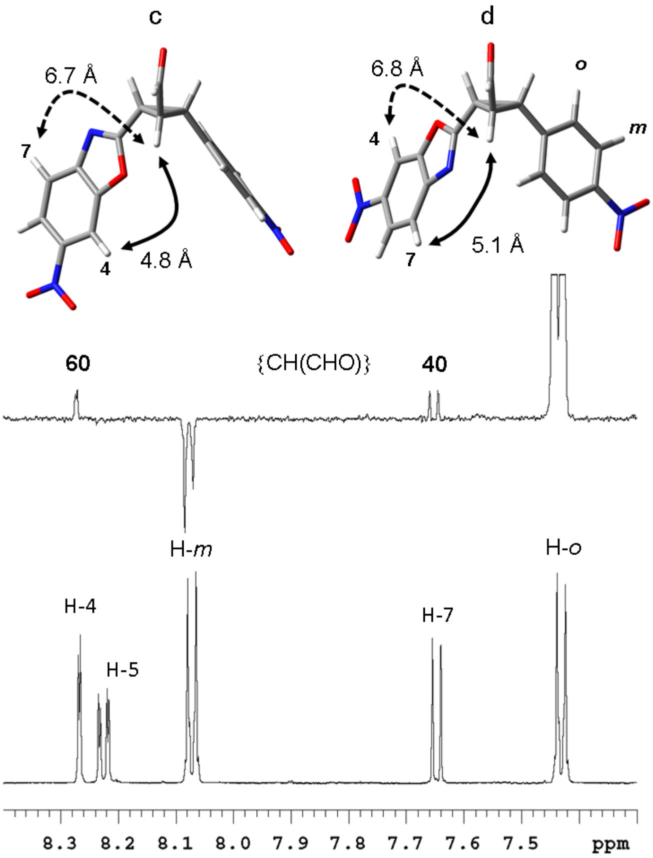

Figure S13. DPFGSE-NOE of **4d-major** recorded on saturation of the CH(CHO) signal and using 4 s mixing time. The negative NOE at 8.08 ppm is due to transferred NOE from the NOE signal at 7.44 ppm.

On saturating the CH(CHO) signal, weak but comparable NOEs were observed on the two aromatic signals in position 4 (*ortho* to the oxygen of benzoxazole) and in position 7 (*ortho* to the nitrogen) of benzoxazole. If only one conformation were populated, NOE should be visible mainly on one signal of the benzoxazole. Taking into account only conformation **c**, the theoretical NOE ratio should be 88:12 in favour of the NOE on H-4. If only conformation **d** were populated, the observed NOE ratio should be reversed to 14:86 (ratio were calculated using the distances of the optimized structures, and using the r^{-6} rule). The experimental evidence of a 60:40 H-4:H-7 ratio suggests that both conformations are appreciably populated. When considering the distances extracted from calculations, the experimental NOE ratio corresponds to a 56:44 ratio in favour of the **c** conformation. Unfortunately the same NOE spectrum taken in acetonitrile did not allow to see any long-range enhancement, most probably because of faster relaxation times that did not allow to develop measurable NOEs for H-4 and H-7. As from Table S5, conformation **d** was calculated

to be the more stable in the gas phase and in chloroform, whereas conformation **a** is the more stable in acetonitrile. Nevertheless, the energy differences are very small and well support the NOE results obtained in chloroform. To evaluate the variations caused by the different conformational ratios, the simulations of the experimental ECD spectrum were obtained using the three different sets of relative energies reported in Table S5. From the simulations reported in Figure S14 it is evident that the simulations obtained using the relative ratio suggested by PCM calculations provide better results than that obtained using the gas-phase conformational ratio. Nevertheless, each simulation well reproduces the experimental trace, and the 1*R*, 2*R*, 3*S* absolute configuration can be reliably assigned to the major isomer of **4d**.

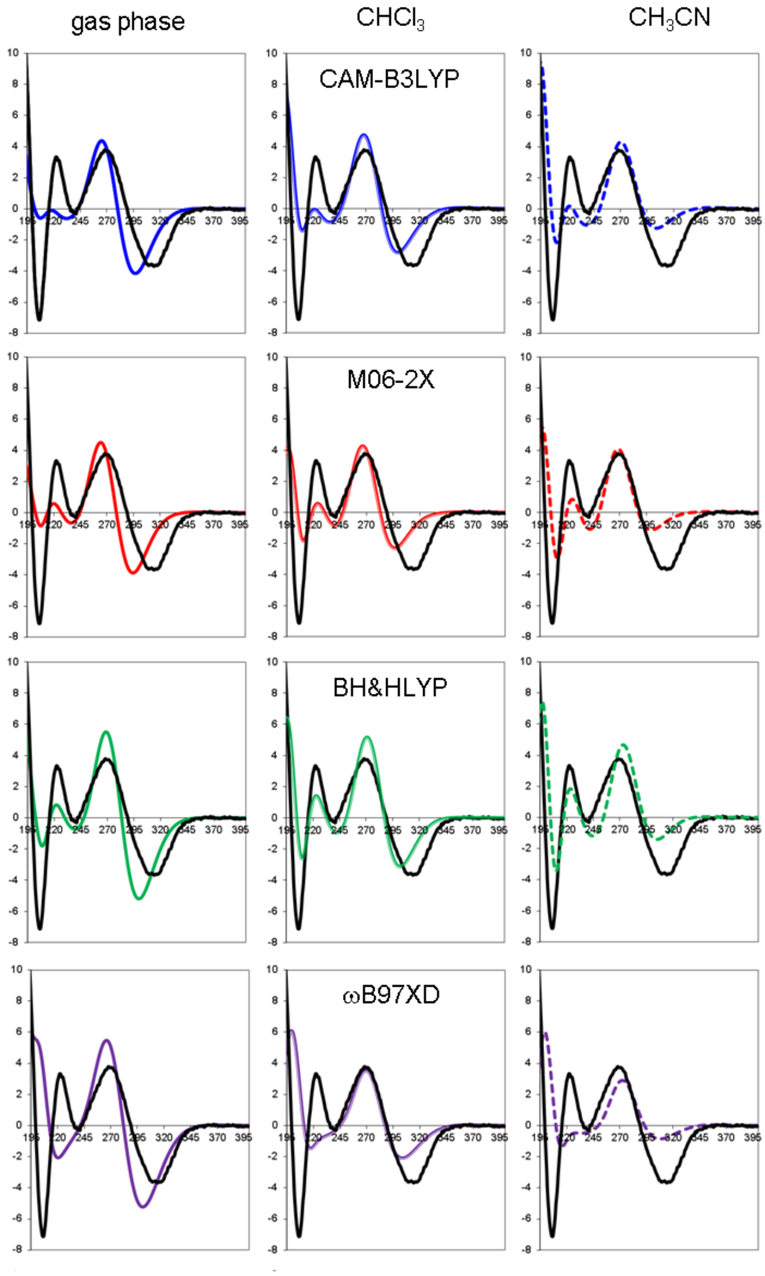

Figure S14. Simulations of the experimental ECD spectrum of **4d-major**. For each graph, the black line correspond to the experimental spectrum. The colored lines correspond to the simulations obtained using the populations derived from B3LYP/6-31+G(d,p) geometry optimizations. Left column: gas-phase optimization; middle column: PCM optimization with chloroform; right column: PCM optimization with acetonitrile. The simulated spectra were vertically scaled and red-shifted by 12-18 nm to get the best match with the experimental spectrum. All the simulations are for the *1R,2R,3S* absolute configuration.

4. Screening of Solvents

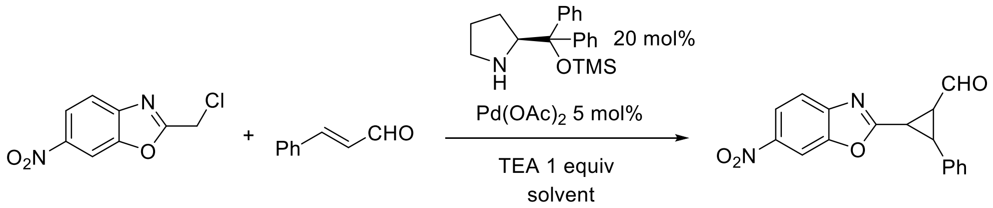

Table S6. Screening of solvents

Entry	Solvent	conversion % (24h)	dr (crude)	*ee* major dia (%)
1	EtOAc	52	2.1:1.7:1	94
2	CH_3CN	59	4:3.2:1	99
3	DMF	33	3.5:2.4:1	---
4	MeOH	97	2:1	> 90
5	DCM	57	2:1.2:1	> 90
6	$CHCl_3$	29	2.4:1.6:1	> 70

5. Screening of Organic Catalyst

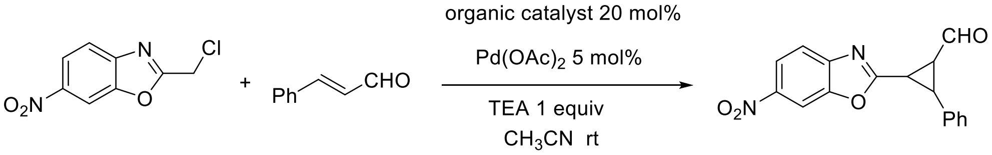

Table S7: Screening of organic catalyst

Entry	Catalyst	Conversion % (24h)	dr (crude)	*ee* %
1	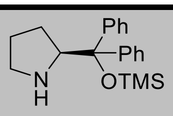	59	4:3.2:1	99
2	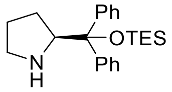	82	2.5:1.1:1	99
3	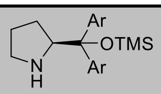 Ar = 3,5-bis(trifluoromethyl)phenyl	traces	---	---
4	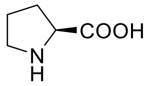	traces	---	---
5	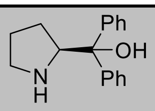	--	---	---
6	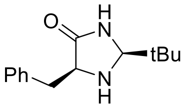	traces	---	---
7	No organic catalyst	---	---	---

a) maybe 4 diastereomers total.

6. Screening of Bases

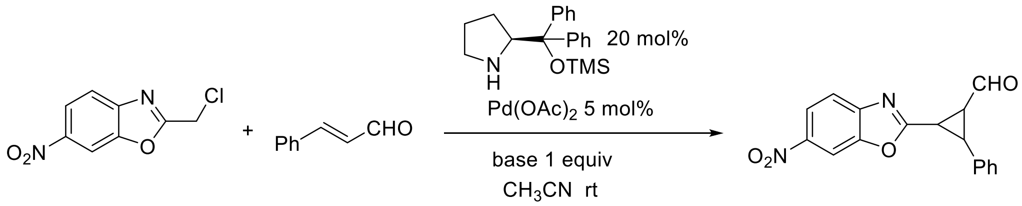

Table S8: Screening of bases

Entry	Base	Conversion % (24h)	dr (crude)	*ee* %
1	TEA	59	4:3.2:1	99
2	DIPEA	full	1.6:0.8:1	99
3	2,6-lutidine	93	4.1:2.4:1	99
4	Cs_2O_3	---	---	---
5	DABCO	34	2.5:1.1:1	> 90
6	No base	29	2.4:1:1	---

7. Screening of Lewis Acid

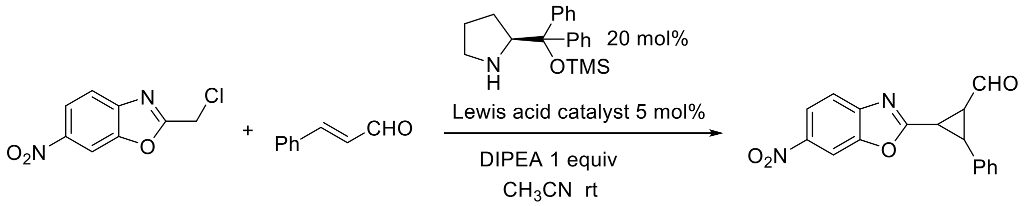

Table S9: Screening of Lewis acid

Entry	Lewis Acid	Conversion % (24h)	dr (crude)	*ee* %
1	$Pd(OAc)_2$	full	1.6:0.8:1	99
2	AgOBz	92	2:1:1	20
3	AgOAc	79	2.7:1.3:1	> 90
4	$Cu(OAc)_2$	61	1.7:0.6:1	> 90
5	$Yb(SO_3CF_3)_2$	---	---	---
6	$Cu(SO_3CF_3)_2$	50	2.5:2.2:1	> 90
7	$PdCl_2$	29	1:0.6:1	> 90
8	No Lewis Acid	traces	---	---

8. Screening of Temperatures

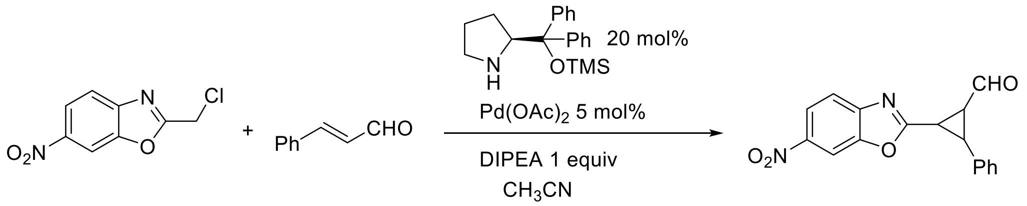

Table S10: Screening of temperatures

Entry	Temperature (°C)	Conversion %	dr (crude)	*ee* %
1	30	63 (24 h)	1.7:1.1:1	> 95
2	4	61 (60 h)	2:0.8:1	> 95
3	rt	full (24h)	1.6:0.8:1	> 95

9. Screening of Metals with 2,6-lutidine

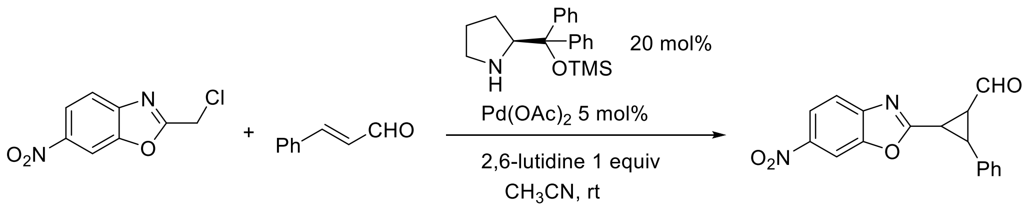

Table S11. Screening of metals with lutidine

Entry	Metal	Conversion % (24h)	dr (crude)
**1**	$Pd(OAc)_2$	full	5.2:2.6:1
**2**	$Cu(OAc)_2$	traces-degradation	---
**3**	AgOAc	Full (NMR less clean than Pd)	2.2:1.4:1

10. Scope of the reaction - aldehydes

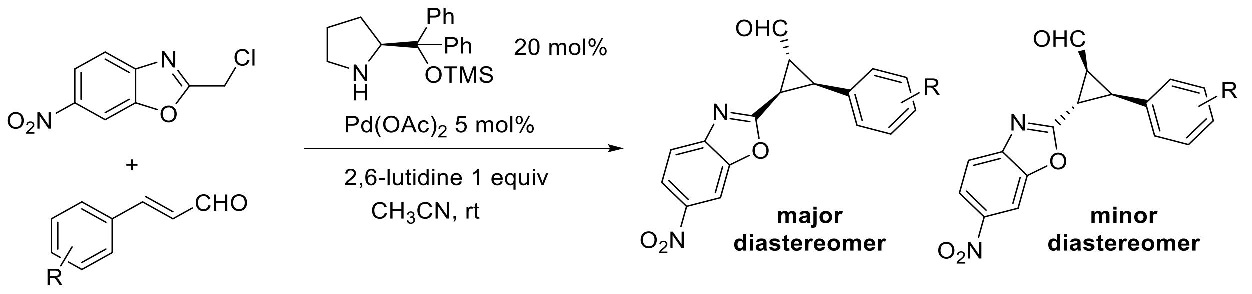

Table S12. Scope of the reaction - aldehydes

**Product**	**Aldehyde**	**Yield %**	**d.r.**	***ee* major**	***ee* minor**
**4a**	**H**	89	4.5 : 1.2 : 1	96 % R / 99 % S	not determined
**4b**	**4-Br**	70	7 : 2.2 : 1	97 % R / 98 % S	not determined
**4c**	**4-Cl**	74	2:1	98 % R / 97 % S	not determined
**4d**	**4-NO_2**	79	14 : 5.6 : 1	>99 % R / >99 % S	89 % S / 81 % R
**4f**	**4-CN**	89	4.8 : 3 : 1	>99 % R / >99 % S	not determined
**4e**	**4-F**	86	6.6 : 2.6 : 1	98 % R / 98 % S	not determined
**4g**	**4-CH_3**	66	5.3 : 1.6 : 1	99 % R / 99 % S	not determined
**4h**	**2-Br**	81	13.4 : 2.3 : 1	>99 % R / >99 % S	not determined
**4i**	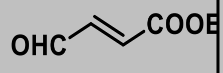	85	> 15:1	81 % R / 87 % S	-----
**4j**	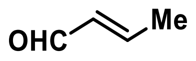	---	---	---	---

11. Scope of the reaction – benzoxazoles

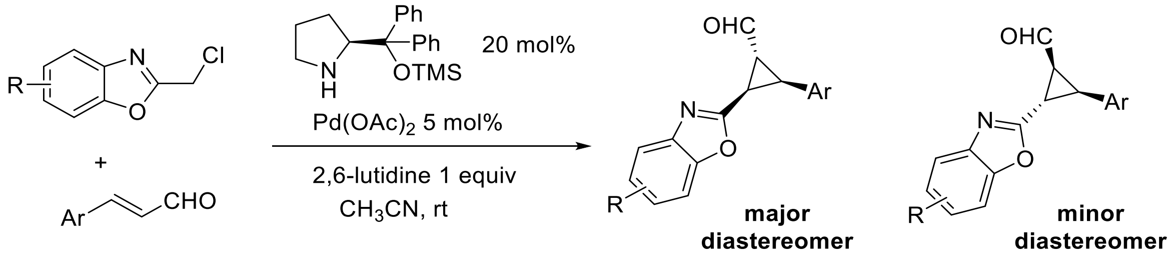

Table S13. Scope of the reaction – benzoxazoles

Product	Benzoxazole	Ar	Yield	dr	*ee* major	*ee* minor
**4a**	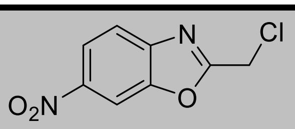	Ph	89%	4.5 : 1.2 : 1	96% R / 99% S	Not determined
**5a**	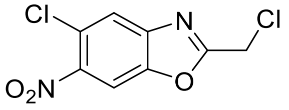	Ph	68%	10.5 : 3.3 : 1	>99% R / >99% S	90% R
**5g**	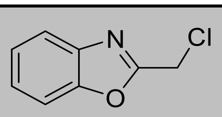	Ph	traces	---	---	---
**5c**	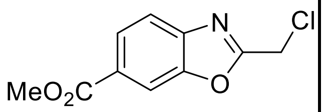	Ph	78%	4.5 : 1.9 : 1	98% R / 98% S	not determined
**5b**	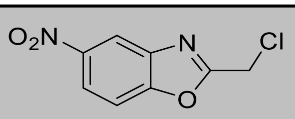	Ph	55%	6.2 : 1.3 : 1	99% R / 98% S	not determined
**5d**	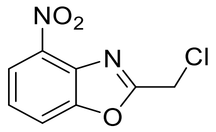	Ph	51%	2.3 : 1.6 : 1	>99% R / >99% S	not determined
**5e**	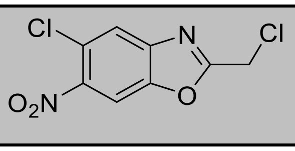	*p*BrC$_6$H$_4$	85%	8.1 : 4.8 : 1	98% R / 97% S	not determined
**5f**	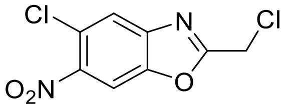	*m*BrC$_6$H$_4$	66%	17.4 : 6.3 : 1	91% R / 97% S	not determined

12. Synthesis of the starting material – benzoxazoles

General procedure:

In a round bottom flask, equipped with a condenser, were added 1 equivalent of aminophenol followed by 1,1 equivalents of 2-chloro-1,1,1-triethoxyethane or 2-chloro-1,1,1-trimethoxyethane. The reaction mixture is stirred and heated. The reaction is followed by TLC. After the reaction is completed, the crude is purified by recrystallization or by flash column chromatography (*n*-hexane/EtOAc) to obtain the desired benzoxazole.

2-(chloromethyl)-6-nitrobenzoxazole (1a)

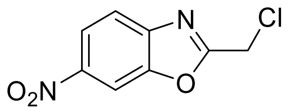

The reaction was performed following the general procedure adding 2-amino-5-nitrophenol (712 mg, 4.623 mmol, 1 equiv) and 2-chloro-1,1,1-triethoxyethane (1 g, 5.085 mmol, 1.1 equiv). The reaction mixture was stirred at 100°C for 4 hours. The crude was purified by recrystallization with EtOH/H_2O to obtain 584 mg of the desired product as dark orange solid. Yield: 59%.

^1H NMR (400 MHz, $CDCl_3$) δ 8.49 (s, 1H), 8.35 (d, *J* = 8.6 Hz, 1H), 7.87 (d, *J* = 8.7 Hz, 1H), 4.81 (s, 2H).

^{13}C NMR (101 MHz, $CDCl_3$) δ 165.4 (Cq), 150.3 (Cq), 145.9 (Cq), 145.9 (Cq), 120.9 (CH), 120.7 (CH), 107.7 (CH), 35.9 (CH_2).

HRMS *m/z* (ESI+) Exact mass calculated for $C_8H_6ClN_2O_3$ $[M+H]^+$: 213.0061, found: 213.0062.

5-chloro-2-(chloromethyl)-6-nitrobenzoxazole (1b)

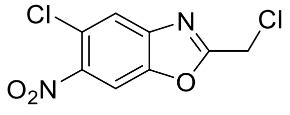

The reaction was performed following the general procedure adding 2-amino-4-chloro-5-nitrophenol (1.66 g, 8.801 mmol, 1 equiv) and 2-chloro-1,1,1-trimethoxyethane (1.3 mL, 9.681 mmol, 1.1 equiv). The reaction mixture was stirred at 100°C for 19 hours. The crude was purified by recrystallization with EtOH/H_2O to obtain 1.715 g of the desired product as dark brown solid. Yield: 79%.

^1H NMR (400 MHz, $CDCl_3$) δ 8.15 (s, 1H), 7.94 (s, 1H), 4.80 (s, 2H).

^{13}C NMR (101 MHz, $CDCl_3$) δ 165.9 (Cq), 148.5 (Cq), 145.6 (Cq), 144.3 (Cq), 124.0 (Cq), 123.2 (CH), 109.0 (CH), 35.7 (CH_2).

HRMS *m/z* (ESI+) Exact mass calculated for $C_8H_5Cl_2N_2O_3$ $[M+H]^+$: 246.9672, found: 246.9671.

methyl 2-(chloromethyl)benzoxazole-6-carboxylate (1d)

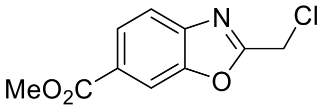

The reaction was performed following the general procedure adding methyl 4-amino-3-hydroxybenzoate (980 mg, 5.862 mmol, 1 equiv) and 2-chloro-1,1,1-trimethoxyethane (0.87 mL, 6.448 mmol, 1.1 equiv). The reaction mixture was stirred at 100°C for 19 hours. The crude was purified by recrystallization with EtOH/H_2O to obtain 1.250 g of the desired product as light brown solid. Yield: 95%.

^1H NMR (400 MHz, $CDCl_3$) δ 8.24 (d, *J* = 1.0 Hz, 1H), 8.09 (dd, *J* = 8.4, 1.4 Hz, 1H), 7.77 (d, *J* = 8.4 Hz, 1H), 4.78 (s, 2H), 3.96 (s, 3H).

^{13}C NMR (101 MHz, $CDCl_3$) δ 166.3 (Cq), 163.5 (Cq), 150.8 (Cq), 144.6 (Cq), 128.1 (Cq), 126.5 (CH), 120.2 (CH), 112.6 (CH), 52.5 (CH_3), 36.2 (CH_2).

HRMS *m/z* (ESI+) Exact mass calculated for $C_{10}H_9ClNO_3$ $[M+H]^+$: 226.0265, found: 226.0269.

2-(chloromethyl)-5-nitrobenzoxazole (1c)

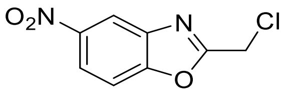

The reaction was performed following the general procedure adding 2-amino-4-nitrophenol (712 mg, 4.623 mmol, 1 equiv) and 2-chloro-1,1,1-triethoxyethane (1 g, 5.085 mmol, 1.1 equiv). The reaction mixture was stirred at 80°C for 12 hours. The residual solvent was evaporated under *vacuum* to obtain 686 mg of the desired product as brown solid. Yield: 70%.

^1H NMR (400 MHz, $CDCl_3$) δ 8.65 (d, *J* = 1.9 Hz, 1H), 8.37 (dd, *J* = 9.0, 2.1 Hz, 1H), 7.69 (d, *J* = 9.0 Hz, 1H), 4.80 (s, 2H).

^{13}C NMR (101 MHz, $CDCl_3$) δ 164.0 (Cq), 154.5 (Cq), 145.6 (Cq), 141.2 (Cq), 122.0 (CH), 117.1 (CH), 111.3 (CH), 35.9 (CH_2).

HRMS *m/z* (ESI+) Exact mass calculated for $C_8H_6ClN_2O_3$ $[M+H]^+$: 213.0061, found: 213.0062.

2-(chloromethyl)-4-nitrobenzoxazole[1] (1e)

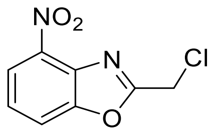

The reaction was performed following the general procedure adding 2-amino-3-nitrophenol (1.355 g, 8.791 mmol, 1 equiv) and 2-chloro-1,1,1-trimethoxyethane (1.305 mL, 9.670 mmol, 1.1 equiv). The reaction mixture was stirred at 100°C for 12 hours. The crude was purified by recrystallization with EtOH/H_2O to obtain 720 mg of the desired product as light brown solid. Yield: 93%.

^{1}H NMR (400 MHz, $CDCl_3$) δ 8.25 (d, *J* = 8.2 Hz, 1H), 7.94 (d, *J* = 8.2 Hz, 1H), 7.59 (t, *J* = 8.2 Hz, 1H), 4.88 (s, 2H).

2-(chloromethyl)benzoxazole[2] (1f)

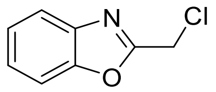

The reaction was performed following the general procedure adding 2-aminophenol (640 mg, 5.865 mmol, 1 equiv) and 2-chloro-1,1,1-trimethoxyethane (0.87 mL, 6.451 mmol, 1.1 equiv). The reaction mixture was stirred at 80°C for 20 hours. The crude was purified by flash column chromatography (*n*-hexane/EtOAc 5:1) to obtain 490 mg of the desired product as light orange oil. Yield: 54%.

^{1}H NMR (400 MHz, $CDCl_3$) δ 7.75 (dd, *J* = 6.7, 2.4 Hz, 1H), 7.56 (dd, *J* = 6.9, 2.2 Hz, 1H), 7.43 – 7.32 (m, 2H), 4.76 (s, 2H).

13. Synthesis of the starting material – α,β-unsaturated aldehydes

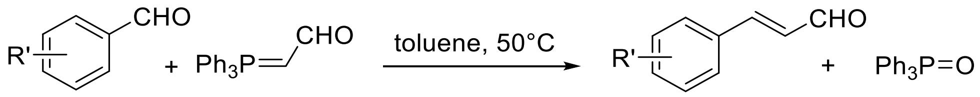

The starting aldehydes were synthesized trough a Wittig reaction, following the procedure described in literature. In a round bottom flask a substituted benzaldehyde derivative (2 equiv) and (triphenylphosphoranyldiene)acetaldehyde (1 equiv) were stirred in anhydrous toluene under reflux at 50°C under argon. The crude mixture was purified by a flash column chromatography.

Literature's references for the aldehydes synthesized:

- (*E*)-3-(4-bromophenyl)acrylaldehyde **(2b)**, (*E*)-3-(*p*-tolyl)acrylaldehyde **(2g)** and (*E*)-3-(4-chlorophenyl)acrylaldehyde **(2c)**[3]
- (*E*)-3-(4-nitrophenyl)acrylaldehyde **(2d)** and (*E*)-3-(2-bromophenyl)acrylaldehyde **(2h)**[4]
- (*E*)-3-(4-fluorophenyl)acrylaldehyde **(2e)**[5]
- (*E*)-4-(3-oxoprop-1-en-1-yl)benzonitrile **(2f)**[6]
- ethyl (*E*)-4-oxobut-2-enoate **(2i)**[7]
- (*E*)-3-(3-bromophenyl)acrylaldehyde **(2k)**[8]

14. General procedure for the synthesis of cyclopropanes

In a closed vial were added in this sequence: the organic catalyst 2-(diphenyl((trimethylsilyl)oxy)methyl)pyrrolidine (20 mol% equiv), α,β-unsaturated aldehyde (2 equiv), azaarene (1 equiv), $Pd(OAc)_2$ (5 mol% equiv) and CH_3CN (1 mL). To the reaction mixture, was finally added 2,6-lutidine (1 equiv). The reaction mixture was stirred at room temperature and then concentrated *in vacuo*. The crude was purified by flash column chromatography (*n*-hexane/EtOAc) to obtain the desired product.

15. Final products characterisation

Compound 4a

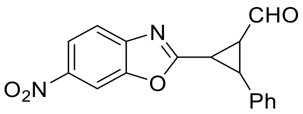

The reaction was performed following the general procedure adding: 2-(diphenyl((trimethylsilyl)oxy)methyl)pyrrolidine (306 mg, 0.941 mmol, 20 mol% equiv), cynnamaldehyde (1.243 g, 9.408 mmol, 2 equiv), 2-(chloromethyl)-6-nitrobenzoxazole (1 g, 4.704 mmol, 1 equiv), $Pd(OAc)_2$ (53 mg, 0.235 mmol, 5 mol% equiv), CH_3CN (10 mL) and 2,6-lutidine (504 mg, 4.704 mmol, 1 equiv). The crude was purified by flash column chromatography (hexane/EtOAc 10:1) to obtain 1.289 g of the desired products as dark yellow oil. Yield: 89%. The diastereomeric ratio was calculated based on the isolated products after column chromatography. d.r.: 4.5:1.2:1

(1*R*,2*R*,3*S*)-2-(6-nitrobenzoxazol-2-yl)-3-phenylcyclopropane-1-carbaldehyde

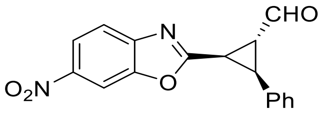

major

IR (liquid film): 2922, 2851, 1709 (CHO), 1570 (aromatic NO_2), 1525, 1345 (aromatic NO_2), 758 cm^{-1}.

^1H NMR (400 MHz, $CDCl_3$) δ 9.89 (d, *J* = 2.8 Hz, 1H), 8.24 – 8.18 (m, 2H), 7.64 (d, *J* = 8.6 Hz, 1H), 7.24 – 7.16 (m, 5H), 3.57 (ddd, *J* = 5.6, 5.6, 2.8 Hz, 1H), 3.41 (bd, *J* = 5.5 Hz, 2H).

^{13}C NMR (101 MHz, $CDCl_3$) δ 197.4 (CH), 166.7 (Cq), 150.0 (Cq), 146.3 (Cq), 145.2 (Cq), 133.0 (Cq), 128.8 (CH), 128.7 (CH), 128.0 (CH), 120.7 (CH), 119.7 (CH), 107.0 (CH), 34.9 (CH), 34.5 (CH), 26.24 (CH).

The enantiomeric excess was determined by HPLC using a Chiralpak IA column (hexane/*i*PrOH = 85:15, flow rate 1.0 mL/min, λ = 210 nm): t_r (S) = 16.1, t_r (R) = 17.0, 96% (R) and 99% (S) *ee*.

$[\alpha]_D^{22}$ = -107.6° (c = 0.1, $CHCl_3$) **(*S* catalyst)**

HRMS *m/z* (ESI+) Exact mass calculated for $C_{17}H_{13}N_2O_4$ $[M+H]^+$: 309.0870, found: 309.0872.

(1*R*,2*R*,3*R*)-2-(6-nitrobenzoxazol-2-yl)-3-phenylcyclopropane-1-carbaldehyde

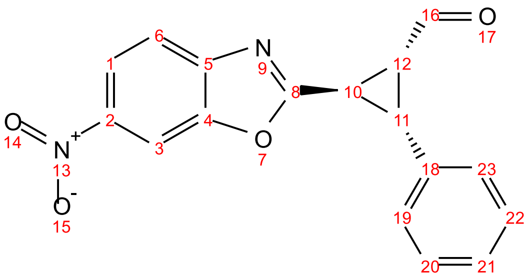

minor

^{1}H NMR (400 MHz, $CDCl_3$) δ 9.16 (d, *J* = 5.0 Hz, 1H,-C<u>H</u>O), 8.43 (d, *J* = 2.1 Hz, 1H,H^3), 8.32 (dd, *J* = 8.8, 2.1 Hz, 1H, H^1), 7.77 (d, *J* = 8.8 Hz, 1H, H^6), 7.38 (m, *J* = 4.4 Hz, 5H, Ph), 3.66 (m, 2H, H^{12}, H^{11}), 3.08 (m, 1H, H^{10}).

^{13}C NMR (101 MHz, $CDCl_3$) δ 197.2 (C^{16}), 166.5 (Cq), 149.8 (Cq), 146.1 (Cq), 145.0 (Cq), 132.8 (Cq), 128.7 (CH), 128.5 (CH), 127.9 (CH), 120.6 (C^1), 119.5 (C^6), 106.9 (C^3), 34.8 (C^{11}), 34.3 (C^{12}), 26.1 (C^{10}).

Proton and carbon were assigned using the COSY and HMBC NMR analysis.

$[\alpha]_D^{21}$ = +21.4° (c = 0.4, $CHCl_3$) **(*R* catalyst)**

Mixture of minor and minor' :

^{1}H NMR (400 MHz, $CDCl_3$) δ 9.69 (d, *J* = 5.7 Hz, 1H'), 9.16 (d, *J* = 5.0 Hz, 1H), 8.41 (d, *J* = 2.1 Hz, 2H), 8.30 (dd, *J* = 8.8, 2.0 Hz, 1H + 1H'), 7.77 (dd, *J* = 8.8, 4.7 Hz, 1H + 1H'), 7.43 – 7.23 (m, 11H Ar), 3.77 (t, *J* = 6.3 Hz, 1H'), 3.72 – 3.63 (m, 2H), 3.20 (dd, *J* = 8.9, 6.5 Hz, 1H'), 3.09 (dt, *J* = 9.7, 5.0 Hz, 1H), 2.80 (dt, *J* = 8.9, 5.9 Hz, 1H').

^{13}C NMR (101 MHz, $CDCl_3$) δ 197.0' (CH), 196.0 (CH), 169.1 (Cq), 167.5 (Cq), 149.9 (Cq), 149.8 (Cq), 146.5 (Cq), 146.3 (Cq), 145.2 (Cq), 145.1 (Cq), 135.8 (Cq), 132.8 (Cq), 129.0 (CH), 129.0 (CH), 128.9 (CH), 128.1 (CH), 128.0 (CH), 126.6 (CH), 120.9 (CH), 120.8 (CH), 119.7 (CH), 119.5 (CH), 107.1 (CH), 107.0 (CH), 39.4 (CH), 38.7 (CH), 35.2 (CH), 32.2 (CH), 26.6 (CH), 22.0 (CH).

HRMS *m/z* (ESI+) Exact mass calculated for $C_{17}H_{13}N_2O_4$ $[M+H]^+$: 309.0870, found: 309.0868.

Compound 4b

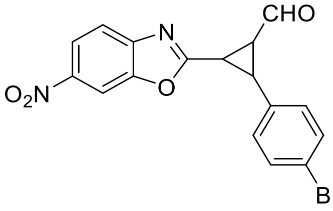

The reaction was performed following the general procedure adding: 2-(diphenyl((trimethylsilyl)oxy)methyl)pyrrolidine (31 mg, 0.094 mmol, 20 mol% equiv), (*E*)-3-(4-bromophenyl)acrylaldehyde (198 mg, 0.940 mmol, 2 equiv), 2-(chloromethyl)-6-nitrobenzoxazole (100 mg, 0.470 mmol, 1 equiv), $Pd(OAc)_2$ (5 mg, 0.024 mmol, 5 mol% equiv), CH_3CN (1 mL) and 2,6-lutidine (50 mg, 0.470 mmol, 1 equiv). The crude was purified by flash column chromatography (hexane/EtOAc 5:1) to obtain 127 mg of the desired products as light yellow solid. Yield: 70%. The diastereomeric ratio was calculated based on the isolated products after column chromatography. d.r.: 7:2.2:1

(1*S*,2*R*,3*S*)-2-(4-bromophenyl)-3-(6-nitrobenzooxazol-2-yl)cyclopropane-1-carbaldehyde

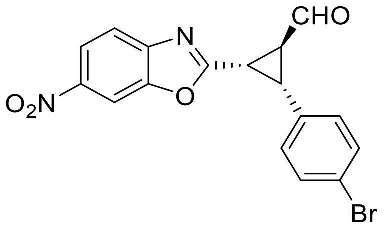

major

IR (liquid film): 2926, 1714 (CHO), 1571 (aromatic NO_2), 1570, 1523, 1344 (aromatic NO_2), 760 cm^{-1}.

^1H NMR (400 MHz, $CDCl_3$) δ 9.88 (d, *J* = 2.7 Hz, 1H), 8.24 (d, *J* = 2.1 Hz, 1H), 8.19 (dd, *J* = 8.8, 2.1 Hz, 1H), 7.64 (d, *J* = 8.7 Hz, 1H), 7.33 – 7.27 (m, 2H), 7.12 – 7.05 (m, 2H), 3.53 (ddd, *J* = 5.9, 5.3, 2.7 Hz, 1H), 3.38 (dd, *J* = 9.8, 5.2 Hz, 1H), 3.32 (dd, *J* = 9.8, 6.0 Hz, 1H).

^{13}C NMR (101 MHz, $CDCl_3$) δ 197.1 (CH), 166.2 (Cq), 149.9 (Cq), 146.1 (Cq), 145.2 (Cq), 132.0 (Cq), 131.7 (Cq), 130.5 (CH), 122.1 (CH), 120.8 (CH), 119.7 (CH), 107.1 (CH), 34.4 (CH), 34.2 (CH), 26.2 (CH).

The enantiomeric excess was determined by HPLC using a Chiralpak OD-H column (hexane/*i*PrOH = 85:15, flow rate 1.0 mL/min, λ = 210 nm): t_r (S) = 56.6, t_r (R) = 43.5, 97% (R) and 98% (S) *ee*.

$[\alpha]_D^{22}$ = +168.0° (c = 0.5, $CHCl_3$) **(*R* catalyst)**

mp: 116-117 °C

HRMS *m/z* (ESI+) Exact mass calculated for $C_{17}H_{12}BrN_2O_4$ $[M+H]^+$: 386.9975, found: 386.9984.

(1*R*,2*R*,3*R*)-2-(4-bromophenyl)-3-(6-nitrobenzooxazol-2-yl)cyclopropane-1-carbaldehyde

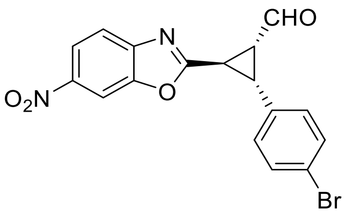

minor

^1H NMR (400 MHz, $CDCl_3$) δ 9.26 (d, *J* = 4.3 Hz, 1H), 8.42 (d, *J* = 2.1 Hz, 1H), 8.32 (dd, *J* = 8.8, 2.1 Hz, 1H), 7.76 (d, *J* = 8.8 Hz, 1H), 7.51 – 7.48 (m, 2H), 7.26 – 7.22 (m, 2H), 3.60 (d, *J* = 6.8 Hz, 2H), 3.13 (ddd, *J* = 7.7, 6.8, 4.3 Hz, 1H).

^{13}C NMR (101 MHz, $CDCl_3$) δ 195.5 (CH), 168.9 (Cq), 150.0 (Cq), 146.6 (Cq), 145.4 (Cq), 132.2 (CH), 131.9 (Cq), 130.8 (CH), 122.4 (Cq), 121.1 (CH), 119.7 (CH), 107.3 (CH), 38.6 (CH), 35.1 (CH), 22.3 (CH).

Mixture of minor and minor':

^1H NMR (400 MHz, $CDCl_3$) δ 9.66 (d, *J* = 5.5 Hz, 1H'), 9.25 (d, *J* = 4.3 Hz, 1H), 8.40 (d, *J* = 1.9 Hz, 1H + 1H'), 8.30 (dd, *J* = 8.8, 2.1 Hz, 1H + 1H'), 7.76 (dd, *J* = 8.7, 6.1 Hz, 1H + 1H'), 7.49 (t, *J* = 7.0 Hz, 2H + 2H'), 7.24 (d, *J* = 8.4 Hz, 2H), 7.13 (d, *J* = 8.4 Hz, 2H'), 3.71 (t, *J* = 6.2 Hz, 1H'), 3.60 (d, *J* = 6.1 Hz, 2H), 3.12 (ddd, *J* = 8.7, 6.0, 4.3 Hz, 1H + m, 1H'), 2.75 (dt, *J* = 9.0, 5.9 Hz, 1H').

mp: 119-120°C

HRMS minor *m/z* (ESI+) Exact mass calculated for $C_{17}H_{12}BrN_2O_4$ $[M+H]^+$: 386.9975, found: 386.9982.

HRMS minor' *m/z* (ESI+) Exact mass calculated for $C_{17}H_{12}BrN_2O_4$ $[M+H]^+$: 386.9975, found: 386.9977.

Compound 4c

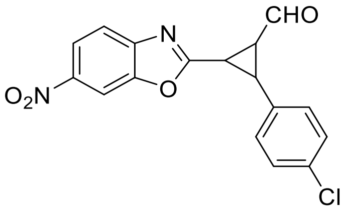

The reaction was performed following the general procedure adding: 2-(diphenyl((trimethylsilyl)oxy)methyl)pyrrolidine (31 mg, 0.094 mmol, 20 mol% equiv), (*E*)-3-(4-chlorophenyl)acrylaldehyde (157 mg, 0.940 mmol, 2 equiv), 2-(chloromethyl)-6-nitrobenzoxazole (100 mg, 0.470 mmol, 1 equiv), $Pd(OAc)_2$ (5 mg, 0.024 mmol, 5 mol% equiv), CH_3CN (1 mL) and 2,6-lutidine (50 mg, 0.470 mmol, 1 equiv). The crude was purified by flash column chromatography (hexane/EtOAc 4:1) to obtain 120 mg of the desired products as orange solid (major dia) and yellow oil (minor dia). Yield: 74%. The diastereomeric ratio was calculated based on the isolated products after column chromatography. d.r.: 43:22:1

(1*S*,2*R*,3*S*)-2-(4-chlorophenyl)-3-(6-nitrobenzoxazol-2-yl)cyclopropane-1-carbaldehyde

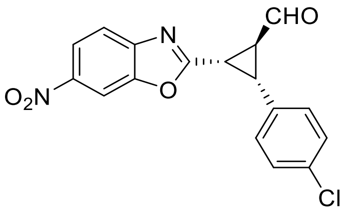

major

IR (liquid film): 3107, 2927, 2924, 2853, 1714 (CHO), 1570 (aromatic NO_2), 1523, 1344 (aromatic NO_2), 751 cm^{-1}.

^{1}H NMR (400 MHz, $CDCl_3$) δ 9.89 (d, *J* = 4.5 Hz, 1H), 8.25 (d, *J* = 2.1 Hz, 1H), 8.21 (dd, *J* = 8.7, 2.1 Hz, 1H), 7.64 (d, *J* = 8.7 Hz, 1H), 7.16 (s, 4H), 3.56 – 3.52 (m, 1H), 3.41 – 3.33 (m, 2H).

^{13}C NMR (101 MHz, $CDCl_3$) δ 197.2 (CH), 166.3 (Cq), 149.9 (Cq), 146.1 (Cq), 145.3 (Cq), 134.0 (Cq), 131.5, 130.2 (CH), 128.9 (CH), 120.8 (CH), 119.8 (CH), 107.1 (CH), 34.4 (CH), 34.2 (CH), 26.3 (CH).

The enantiomeric excess was determined by HPLC using a Chiralpak OD-H column (hexane/*i*PrOH = 80:20, flow rate 1.0 mL/min, λ = 250 nm): t_r (S) = 41.0, t_r (R) = 28.2, 98% (R) and 97% (S) *ee*.

$[\alpha]_D^{22}$ = +132.4° (c = 1.3, $CHCl_3$) **(*R* catalyst)**

mp: 86-87°C

HRMS *m/z* (ESI+) Exact mass calculated for $C_{17}H_{12}ClN_2O_4$ $[M+H]^+$: 343.0480, found: 343.0480.

(1*R*,2*R*,3*R*)-2-(4-chlorophenyl)-3-(6-nitrobenzoxazol-2-yl)cyclopropane-1-carbaldehyde

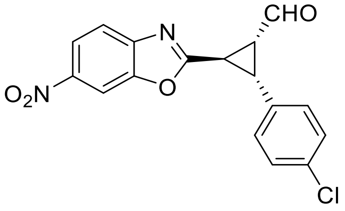

minor

NMR minor diastereomer with traces of the minor':

^{1}H NMR (400 MHz, $CDCl_3$) δ 9.26 (d, *J* = 4.3 Hz, 1H), 8.42 (d, *J* = 2.1 Hz, 1H), 8.32 (dd, *J* = 8.8, 2.1 Hz, 1H), 7.76 (d, *J* = 8.8 Hz, 1H), 7.36 – 7.28 (m, 4H), 3.65 – 3.58 (m, 2H), 3.13 (ddd, *J* = 8.7, 5.7, 4.4 Hz, 1H).

^{13}C NMR (101 MHz, $CDCl_3$) δ 195.4 (CH), 169.0 (Cq), 150.0 (Cq), 146.6 (Cq), 145.4 (Cq), 134.3 (Cq), 131.4 (Cq), 130.5 (CH), 129.3 (CH), 121.1 (CH), 119.8 (CH), 107.3 (CH), 38.7 (CH), 35.0 (CH), 22.4 (CH).

$[\alpha]_D^{21}$ = +36.7° (c = 0.2, $CHCl_3$) **(*R* catalyst)**

Compound 4d

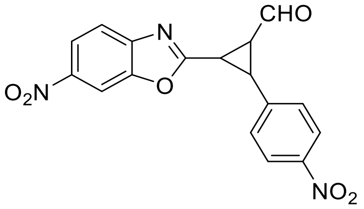

The reaction was performed following the general procedure adding: 2-(diphenyl((trimethylsilyl)oxy)methyl)pyrrolidine (31 mg, 0.094 mmol, 20 mol% equiv), (*E*)-3-(4-nitrophenyl)acrylaldehyde (166 mg, 0.940 mmol, 2 equiv), 2-(chloromethyl)-6-nitrobenzoxazole (100 mg, 0.470 mmol, 1 equiv), $Pd(OAc)_2$ (5 mg, 0.024 mmol, 5 mol% equiv), CH_3CN (1 mL) and 2,6-lutidine (50 mg, 0.470 mmol, 1 equiv). The crude was purified by flash column chromatography (hexane/EtOAc 5:1) to obtain 151 mg of the desired products as orange oil (major dia) and yellow solid (minor dia). Yield: 79%. The diastereomeric ratio was calculated based on the isolated products after column chromatography. d.r.: 14:5.6:1

(1*R*,2*R*,3*S*)-2-(6-nitrobenzoxazol-2-yl)-3-(4-nitrophenyl)cyclopropane-1-carbaldehyde

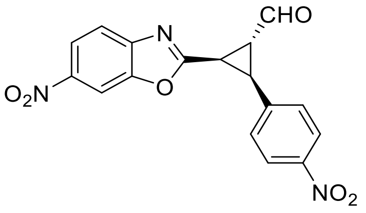

major

IR (liquid film): 3109, 2924, 2852, 1714 (CHO), 1571 (aromatic NO_2), 1518, 1344 (aromatic NO_2), 735 cm^{-1}.

^{1}H NMR (400 MHz, $CDCl_3$) δ 9.96 (d, *J* = 2.4 Hz, 1H), 8.25 (d, *J* = 2.1 Hz, 1H), 8.21 (dd, *J* = 8.8, 2.1 Hz, 1H), 8.06 (bd, *J* = 8.7 Hz, 2H), 7.64 (d, *J* = 8.8 Hz, 1H), 7.44 (bd, *J* = 8.7 Hz, 2H), 3.66 (ddd, *J* = 5.7, 5.6, 2.4 Hz, 1H), 3.46 (bd, *J* = 5.7 Hz, 2H).

^{13}C NMR (101 MHz, $CDCl_3$) δ 196.6 (CH), 165.6 (Cq), 149.9 (Cq), 147.6 (Cq), 145.9 (Cq), 145.4 (Cq), 140.5 (Cq), 129.9 (CH), 123.8 (CH), 121.0 (CH), 119.9 (CH), 107.2 (CH), 34.4 (CH), 34.1 (CH), 26.6 (CH).

The enantiomeric excess was determined by HPLC using a Chiralpak OD-H column (hexane/*i*PrOH = 55:45, flow rate 0.8 mL/min, λ = 210 nm): t_r (S) = 44.5, t_r (R) = 36.0, >99% (R and S) *ee*.

$[\alpha]_D^{21}$ = -50.1° (c = 1.0, $CHCl_3$) **(*S* catalyst)**

HRMS *m/z* (ESI+) Exact mass calculated for $C_{17}H_{12}N_3O_6$ $[M+H]^+$: 354.0721, found: 354.0726.

(1*R*,2*R*,3*R*)-2-(6-nitrobenzoxazol-2-yl)-3-(4-nitrophenyl)cyclopropane-1-carbaldehyde

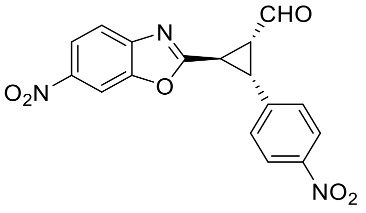

minor

^{1}H NMR (400 MHz, $CDCl_3$) δ 9.44 (d, *J* = 3.2 Hz, 1H), 8.43 (d, *J* = 2.0 Hz, 1H), 8.32 (d, *J* = 8.8 Hz, 1H), 8.22 (d, *J* = 8.7 Hz, 2H), 7.77 (d, *J* = 8.8 Hz, 1H), 7.54 (d, *J* = 8.6 Hz, 2H), 3.71 (bd, *J* = 6.8 Hz, 2H), 3.30 (ddd, *J* = 7.9, 6.7, 3.2 Hz, 1H).

^{13}C NMR (101 MHz, $CDCl_3$) δ 194.7 (CH), 168.4 (Cq), 150.0 (Cq), 147.8 (Cq), 146.5 (Cq), 145.5 (Cq), 140.2 (Cq), 130.2 (CH), 124.1 (CH), 121.2 (CH), 119.9 (CH), 107.3 (CH), 38.6 (CH), 35.5 (CH), 22.7 (CH).

The enantiomeric excess was determined by HPLC using a Chiralpak IB column (hexane/*i*PrOH = 70:30, flow rate 1.0 mL/min, λ = 210 nm): t_r (S) = 40.3, t_r (R) = 38.7, 81% (R) and 89% (S) *ee*.

$[\alpha]_D^{22}$ = +26.9° (c = 0.5, $CHCl_3$) **(*R* catalyst)**

mp: 190°C decomposition

HRMS *m/z* (ESI+) Exact mass calculated for $C_{17}H_{12}N_3O_6$ $[M+H]^+$: 354.0721, found: 354.0718.

Compound 4f

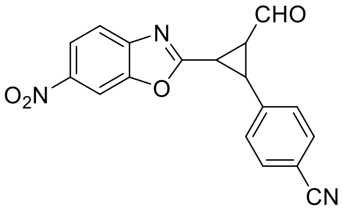

The reaction was performed following the general procedure adding: 2-(diphenyl((trimethylsilyl)oxy)methyl)pyrrolidine (31 mg, 0.094 mmol, 20 mol% equiv), (*E*)-4-(3-oxoprop-1-en-1-yl)benzonitrile (148 mg, 0.940 mmol, 2 equiv), 2-(chloromethyl)-6-nitrobenzoxazole (100 mg, 0.470 mmol, 1 equiv), $Pd(OAc)_2$ (5 mg, 0.024 mmol, 5 mol% equiv), CH_3CN (1 mL) and 2,6-lutidine (50 mg, 0.470 mmol, 1 equiv). The crude was purified by flash column chromatography (hexane/EtOAc 5:1) to obtain 139 mg of the desired products as orange solid (major dia) and yellow oil (minor dia). Yield: 89%. The diastereomeric ratio was calculated based on the isolated products after column chromatography. d.r.: 4.8:3:1

4-((1*S*,2*R*,3*R*)-2-formyl-3-(6-nitrobenzoxazol-2-yl)cyclopropyl)benzonitrile

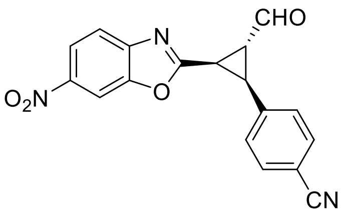

major

IR (liquid film): 2923, 2838, 2229 (CN), 1714 (CHO), 1571 (aromatic NO_2), 1523, 1345 (aromatic NO_2), 759 cm^{-1}.

^{1}H NMR (400 MHz, $CDCl_3$) δ 9.95 (d, *J* = 2.4 Hz, 1H), 8.27 (d, *J* = 2.0 Hz, 1H), 8.23 (dd, *J* = 8.7, 2.1 Hz, 1H), 7.65 (d, *J* = 8.7 Hz, 1H), 7.51 (bd, *J* = 8.4 Hz, 2H), 7.37 (bd, *J* = 8.3 Hz, 2H), 3.62 (ddd, *J* = 5.7, 5.6, 2.4 Hz, 1H), 3.47 – 3.39 (m, 2H).

^{13}C NMR (101 MHz, $CDCl_3$) δ 196.6 (CH), 165.7 (Cq), 149.9 (Cq), 145.9 (Cq), 145.4 (Cq), 138.5 (Cq), 132.4 (CH), 129.7 (CH), 120.9 (CH), 119.9 (CH), 118.4 (Cq), 112.0 (Cq), 107.1 (CH), 34.4 (CH), 34.2 (CH), 26.5 (CH).

The enantiomeric excess was determined by HPLC using a Chiralpak AY-H column (hexane/*i*PrOH = 60:40, flow rate 0.8 mL/min, λ = 210 nm): t_r (S) = 33.8, t_r (R) = 28.8, >99% (R and S) *ee*.

$[\alpha]_D^{22}$ = -169.2° (c = 0.7, $CHCl_3$) **(*S* catalyst)**

mp: 65°C decomposition

HRMS *m/z* (ESI+) Exact mass calculated for $C_{16}H_{12}N_3O_4$ $[M+H]^+$: 334.0822, found: 334.0814.

4-((1*R*,2*R*,3*R*)-2-formyl-3-(6-nitrobenzoxazol-2-yl)cyclopropyl)benzonitrile

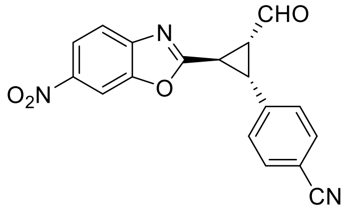

minor

Mixture of minor and minor':

^{1}H NMR (400 MHz, $CDCl_3$) δ 9.69 (d, *J* = 5.3 Hz, 1H'), 9.40 (d, *J* = 3.4 Hz, 1H), 8.42 (bs, 1H + 1H'), 8.32 (bd, *J* = 8.8 Hz, 1H + 1H'), 7.79 – 7.75 (m, 1H + 1H'), 7.69 – 7.65 (m, 2H + 2H'), 7.48 (d, *J* = 8.2 Hz, 2H), 7.38 (d, *J* = 8.3 Hz, 2H'), 3.79 (dd, *J* = 6.3, 6.2 Hz, 1H'), 3.68 (bd, *J* = 7.1 Hz, 2H), 3.30 – 3.19 (m, 1H + 1H'), 2.86 – 2.81 (m, 1H').

^{13}C NMR (101 MHz, $CDCl_3$) δ 196.0 (CH'), 194.8 (CH), 168.5 (Cq), 166.5 (Cq'), 150.1 (Cq'), 150.0 (Cq), 146.5 (Cq), 146.2 (Cq'), 145.4 (Cq), 141.5 (Cq'), 138.2 (Cq), 133.0 (CH'), 132.7 (CH), 130.0 (CH), 127.6 (CH'), 121.2 (CH), 121.1 (CH'), 120.1 (CH'), 119.8 (CH), 118.39 (Cq), 118.38 (Cq'), 112.3 (Cq), 112.2 (Cq'), 107.4 (CH'), 107.3 (CH), 39.2 (CH'), 38.6(CH), 35.6(CH), 31.6 (CH'), 26.9 (CH'), 22.5 (CH).

HRMS *m/z* (ESI+) Exact mass calculated for $C_{16}H_{12}N_3O_4$ $[M+H]^+$: 334.0822, found: 334.0828.

Compound 4e

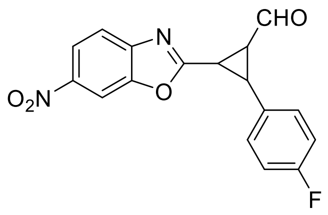

The reaction was performed following the general procedure adding: 2-(diphenyl((trimethylsilyl)oxy)methyl)pyrrolidine (31 mg, 0.094 mmol, 20 mol% equiv), (*E*)-3-(4-fluorophenyl)acrylaldehyde (141 mg, 0.940 mmol, 2 equiv), 2-(chloromethyl)-6-nitrobenzoxazole (100 mg, 0.470 mmol, 1 equiv), $Pd(OAc)_2$ (5 mg, 0.024 mmol, 5 mol% equiv), CH_3CN (1 mL) and 2,6-lutidine (50 mg, 0.470 mmol, 1 equiv). The crude was purified by flash column chromatography (hexane/EtOAc 4:1) to obtain 132 mg of the desired products as yellow oil. Yield: 86%. The diastereomeric ratio was calculated based on the isolated products after column chromatography. d.r.: 6.6:2.6:1

(1*R*,2*S*,3*R*)-2-(4-fluorophenyl)-3-(6-nitrobenzoxazol-2-yl)cyclopropane-1-carbaldehyde

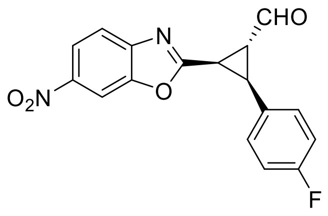

major

IR (liquid film): 3109, 2924, 2850, 1721 (CHO), 1571 (aromatic NO_2), 1513, 1343 (aromatic NO_2), 1232 (aromatic F), 1154 (aromatic F), 757 cm^{-1}.

^1H NMR (400 MHz, $CDCl_3$) δ 9.89 (d, *J* = 2.7 Hz, 1H), 8.24 (d, *J* = 1.4 Hz, 1H), 8.23 – 8.18 (m, 1H), 7.64 (d, *J* = 8.7 Hz, 1H), 7.24 – 7.15 (m, 2H), 6.88 (t, *J* = 8.6 Hz, 2H), 3.53 (ddd, *J* = 5.5, 5.5, 2.8 Hz, 1H), 3.37 (d, *J* = 5.5 Hz, 2H).

^{13}C NMR (101 MHz, $CDCl_3$) δ 197.2 (CH), 166.4 (Cq), 163.6 (Cq), 161.1 (Cq), 149.9 (Cq), 146.1 (Cq), 145.2 (Cq), 130.6 (CH), 130.5 (CH), 128.74 (Cq), 128.71 (Cq), 120.8 (CH), 119.7 (CH), 115.8 (CH), 115.6 (CH), 107.1 (CH), 34.6 (CH), 34.1 (CH), 26.2 (CH).

^{19}F NMR (376 MHz, $CDCl_3$) δ -113.79.

The enantiomeric excess was determined by HPLC using a Chiralpak OD-H column (hexane/*i*PrOH = 80:20, flow rate 1.0 mL/min, λ = 210 nm): t_r (S) = 24.8, t_r (R) = 18.7, 98% (R and S) *ee*.

$[\alpha]_D^{21}$ = -136.6° (c = 1.4, $CHCl_3$) (***S* catalyst**)

HRMS *m/z* (ESI+) Exact mass calculated for $C_{17}H_{12}FN_2O_4$ $[M+H]^+$: 327.0776, found: 327.0771.

(1*R*,2*R*,3*R*)-2-(4-fluorophenyl)-3-(6-nitrobenzoxazol-2-yl)cyclopropane-1-carbaldehyde

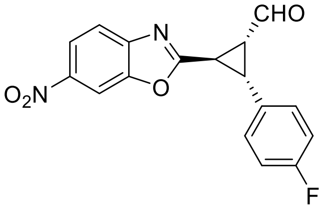

minor

Mixture of minor and minor':

^{1}H NMR (400 MHz, $CDCl_3$) δ 9.67 (d, *J* = 5.6 Hz, 1H'), 9.24 (d, *J* = 4.4 Hz, 1H), 8.43 – 8.42 (m, 1H + 1H'), 8.32 (d, *J* = 8.8, 1H + d, *J* = 2.1 Hz, 1H'), 7.78 (d, *J* = 8.7, 1H), 7.76 (d, *J* = 6.2 Hz, 1H'), 7.34 (dd, *J* = 8.5, 5.3 Hz, 2H), 7.23 (dd, *J* = 5.9, 2.8 Hz, 2H'), 7.10 – 7.03 (m, 2H + 2H'), 3.75 (dd, *J* = 6.3, 6.1 Hz, 1H'), 3.62 (m, 2H), 3.16 – 3.07 (m, 1H + 1H'), 2.77 – 2.72 (m, Hz, 1H').

^{13}C NMR (101 MHz, $CDCl_3$) δ 196.9 (CH'), 195.7 (CH), 169.1 (Cq), 167.3 (Cq'), 163.79 (Cq'), 163.76 (Cq), 161.33 (Cq'), 161.30 (Cq), 150.1 (Cq'), 150.0 (Cq), 146.6 (Cq), 146.4 (Cq'), 145.5 (Cq'), 145.4 (Cq), 131.74 (Cq'), 131.71 (Cq'), 130.9 (CH), 130.8 (CH), 128.7 (Cq), 128.63 (CH'), 128.61 (Cq), 128.5 (CH'), 121.09 (CH), 121.06 (CH'), 120.0 (CH'), 119.7 (CH), 116.3 (CH'), 116.2 (CH), 116.1 (CH'), 115.9 (CH), 107.3 (CH'), 107.2 (CH), 39.4 (CH'), 38.7 (CH), 34.9 (CH), 31.6 (CH'), 26.7 (CH'), 22.5 (CH).

^{19}F NMR (376 MHz, $CDCl_3$) δ -113.43, -113.65'.

HRMS *m/z* (ESI+) Exact mass calculated for $C_{17}H_{12}FN_2O_4$ $[M+H]^+$: 327.0776, found: 327.0769.

Compound 4g

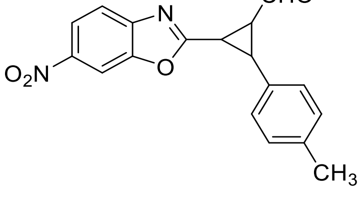

The reaction was performed following the general procedure adding: 2-(diphenyl((trimethylsilyl)oxy)methyl)pyrrolidine (31 mg, 0.094 mmol, 20 mol% equiv), (*E*)-3-(p-tolyl)acrylaldehyde (137 mg, 0.940 mmol, 2 equiv), 2-(chloromethyl)-6-nitrobenzoxazole (100 mg, 0.470 mmol, 1 equiv), $Pd(OAc)_2$ (5 mg, 0.024 mmol, 5 mol% equiv), CH_3CN (1 mL)

and 2,6-lutidine (50 mg, 0.470 mmol, 1 equiv). The crude was purified by flash column chromatography (hexane/EtOAc 5:1) to obtain 100 mg of the desired products as yellow oil. Yield: 66%. The diastereomeric ratio was calculated based on the isolated products after column chromatography. d.r.: 5.3:1.6:1

(1*S*,2*S*,3*R*)-2-(6-nitrobenzoxazol-2-yl)-3-(p-tolyl)cyclopropane-1-carbaldehyde

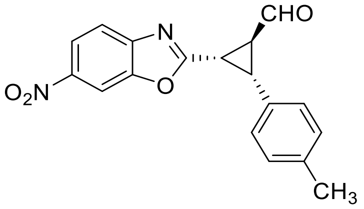

major

IR (liquid film): 3106, 2922, 2853, 1714 (CHO), 1571 (aromatic NO_2), 1522, 1344 (aromatic NO_2), 751, 735 cm^{-1}.

^1H NMR (400 MHz, $CDCl_3$) δ 9.87 (d, *J* = 2.9 Hz, 1H), 8.24 (d, *J* = 1.9 Hz, 1H), 8.21 (dd, *J* = 8.7, 2.1 Hz, 1H), 7.65 (d, *J* = 8.7 Hz, 1H), 7.10 (d, *J* = 8.0 Hz, 2H), 6.99 (d, *J* = 7.9 Hz, 2H), 3.54 (ddd, *J* = 5.6, 5.5, 2.9 Hz, 1H), 3.42 – 3.31 (m, 2H), 2.23 (s, 3H).

^{13}C NMR (101 MHz, $CDCl_3$) δ 197.5 (CH), 166.8 (Cq), 150.0 (Cq), 146.3 (Cq), 145.2 (Cq), 137.8 (Cq), 129.9 (Cq), 129.4 (CH), 128.6 (CH), 120.7 (CH), 119.7 (CH), 107.1 (CH), 34.8 (CH), 34.6 (CH), 26.3 (CH), 21.2 (CH_3).

The enantiomeric excess was determined by HPLC using a Chiralpak AY-H column (hexane/*i*PrOH = 80:20, flow rate 1.0 mL/min, λ = 210 nm): t_r (S) = 22.7, t_r (R) = 21.3, 99% (R and S) *ee*.

$[\alpha]_D^{22}$ = +131.3° (c = 0.5, $CHCl_3$) **(R catalyst)**

HRMS *m/z* (ESI+) Exact mass calculated for $C_{18}H_{15}N_2O_4$ $[M+H]^+$: 323.1026, found: 323.1024.

(1*R*,2*R*,3R)-2-(6-nitrobenzoxazol-2-yl)-3-(p-tolyl)cyclopropane-1-carbaldehyde

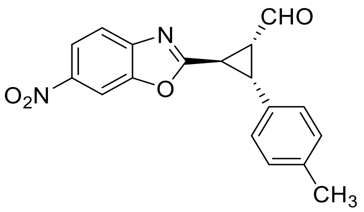

minor

Mixture of minor and minor':

Purity: 62 %, the starting benzoxazole was also present in the NMR

^{1}H NMR (400 MHz, $CDCl_3$) δ 9.66 (d, J = 5.8 Hz, 1H'), 9.14 (d, J = 5.0 Hz, 1H), 8.41 (m, 1H + 1H'), 8.31 (m, 1H + 1H'), 7.76 (d, J = 8.8 Hz, 1H'), 7.75 (d, J = 8.8 Hz, 1H'), 7.26-7.24 (m, 2H), 7.19 – 7.11 (m, 2H + 4H'), 3.72 (dd, J = 6.3, 6.2 Hz, 1H'), 3.61 (m, J = 6.3 Hz, 2H), 3.13 (dd, J = 8.9, 6.5 Hz, 1H'), 3.04 (ddd, J = 9.3, 9.3, 5.0 Hz, 1H), 2.77 – 2.72 (m, 1H'), 2.35 (s, 3H'), 2.34 (s, 3H).

^{13}C NMR (101 MHz, $CDCl_3$) δ 197.3 (CH'), 196.2 (CH), 171.9 (Cq), 169.4 (Cq), 152.4 (Cq), 150.0 (Cq), 146.7 (Cq), 138.2 (Cq), 132.9 (Cq), 129.9 (CH'), 129.8 (CH), 129.0 (CH), 126.7 (CH'), 121.1 (CH), 120.9 (CH'), 119.9 (CH'), 119.7 (CH), 107.3 (CH'), 107.2 (CH), 39.6 (CH'), 38.9 (CH), 35.2 (CH), 32.2 (CH'), 26.8 (CH'), 22.3 (CH), 21.30 (CH), 21.29 (CH').

HRMS *m/z* (ESI+) Exact mass calculated for $C_{18}H_{15}N_2O_4$ $[M+H]^+$: 323.1026, found: 323.1033.

Compound 4h

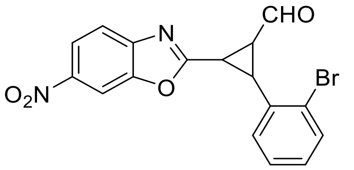

The reaction was performed following the general procedure adding: 2-(diphenyl((trimethylsilyl)oxy)methyl)pyrrolidine (31 mg, 0.094 mmol, 20 mol% equiv), (*E*)-3-(2-bromophenyl)acrylaldehyde (198 mg, 0.940 mmol, 2 equiv), 2-(chloromethyl)-6-nitrobenzoxazole (100 mg, 0.470 mmol, 1 equiv), $Pd(OAc)_2$ (5 mg, 0.024 mmol, 5 mol% equiv), CH_3CN (1 mL) and 2,6-lutidine (50 mg, 0.470 mmol, 1 equiv). The crude was purified by flash column chromatography (hexane/EtOAc 4:1) to obtain 147 mg of the desired products as yellow oil. Yield: 81%. The diastereomeric ratio was calculated based on the isolated products after column chromatography. d.r.: 13.4:2.3:1

(1*R*,2*S*,3*R*)-2-(2-bromophenyl)-3-(6-nitrobenzoxazol-2-yl)cyclopropane-1-carbaldehyde

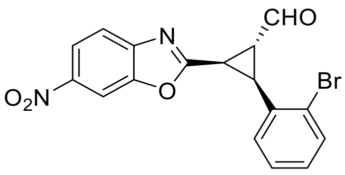

major

IR (liquid film): 3107, 2922, 2850, 1713 (CHO), 1570 (aromatic NO_2), 1513, 1342 (aromatic NO_2), 757 cm^{-1}.

^1H NMR (400 MHz, $CDCl_3$) δ 9.87 (d, *J* = 2.8 Hz, 1H), 8.22 (d, *J* = 2.0 Hz, 1H), 8.19 (dd, *J* = 8.7, 2.1 Hz, 1H), 7.59 (d, *J* = 8.7 Hz, 1H), 7.44 (d, *J* = 7.8 Hz, 1H), 7.24 – 7.20 (m, 2H), 7.13 – 7.07 (m, 1H), 3.54 – 3.47 (m, 2H), 3.40 (dd, *J* = 8.9, 6.9 Hz, 1H).

^{13}C NMR (101 MHz, $CDCl_3$) δ 196.9, 166.8, 149.8, 146.3, 145.1, 132.9, 130.7, 129.7, 127.4, 126.0, 120.7, 119.6, 107.0, 35.8, 35.6, 26.0.

The enantiomeric excess was determined by HPLC using a Chiralpak OD-H column (hexane/*i*PrOH = 70:30, flow rate 0.8 mL/min, λ = 210 nm): t_r (S) = 52.2, t_r (R) = 34.0, >99% (R and S) *ee*.

$[\alpha]_D^{20}$ = -62.3° (c = 1.0, $CHCl_3$) **(S catalyst)**

$[\alpha]_D^{20}$ = +58.7° (c = 1.3, $CHCl_3$) **(R catalyst)**

HRMS *m/z* (ESI+) Exact mass calculated for $C_{17}H_{12}BrN_2O_4$ $[M+H]^+$: 386.9975, found: 386.9964.

(1*R*,2*R*,3*R*)-2-(2-bromophenyl)-3-(6-nitrobenzoxazol-2-yl)cyclopropane-1-carbaldehyde

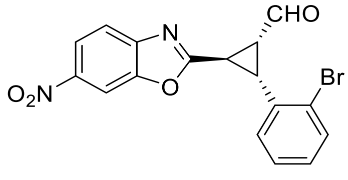

minor

Mainly minor diastereomer present in the NMR with traces of major diastereomer, minor' and starting aldehyde.

^1H NMR (400 MHz, $CDCl_3$) δ 9.38 (d, *J* = 3.7 Hz, 1H), 8.44 (d, *J* = 2.1 Hz, 1H), 8.32 (dd, *J* = 8.7, 2.1 Hz, 1H), 7.78 (d, *J* = 8.8 Hz, 1H), 7.60 (dd, *J* = 7.9, 0.8 Hz, 1H), 7.41 (d, *J* = 7.2 Hz, 1H), 7.37 – 7.33 (m, 1H), 7.24 – 7.20 (m, 1H), 3.61 (dd, *J* = 6.5, 4.9 Hz, 1H), 3.55 (dd, *J* = 9.4, 6.7 Hz, 1H), 3.30 (ddd, *J* = 9.4, 4.6, 3.9 Hz, 1H).

HRMS *m/z* (ESI+) Exact mass calculated for $C_{17}H_{12}BrN_2O_4$ $[M+H]^+$: 386.9975, found: 386.9978.

Compound 4i

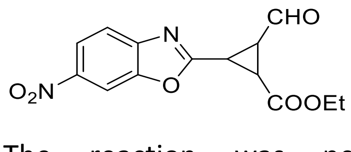

The reaction was performed following the general procedure adding: 2-(diphenyl((trimethylsilyl)oxy)methyl)pyrrolidine (31 mg, 0.094 mmol, 20 mol% equiv), ethyl

(*E*)-4-oxobut-2-enoate (198 mg, 0.940 mmol, 2 equiv), 2-(chloromethyl)-6-nitrobenzoxazole (100 mg, 0.470 mmol, 1 equiv), $Pd(OAc)_2$ (5 mg, 0.024 mmol, 5 mol% equiv), CH_3CN (1 mL) and 2,6-lutidine (50 mg, 0.470 mmol, 1 equiv). The crude was purified by flash column chromatography (hexane/EtOAc 4:1) to obtain 147 mg of the desired products as yellow oil. Yield: 81%. The diastereomeric ratio was calculated based on the isolated products after column chromatography. d.r.: >15

ethyl (1*S*,2*R*,3*R*)-2-formyl-3-(6-nitrobenzoxazol-2-yl)cyclopropane-1-carboxylate

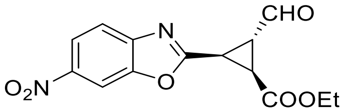

IR (liquid film): 3019, 2920, 2851, 1730 (CHO), 1577 (aromatic NO_2), 1528, 1346 (aromatic NO_2), 1214 (ester), 746 cm^{-1}.

^1H NMR (400 MHz, $CDCl_3$) δ 9.87 (d, *J* = 2.0 Hz, 1H), 8.41 (d, *J* = 2.0 Hz, 1H), 8.29 (dd, *J* = 8.8, 2.1 Hz, 1H), 7.78 (d, *J* = 8.8 Hz, 1H), 4.06 (dq, *J* = 7.1, 2.8 Hz, 2H), 3.53 (ddd, *J* = 5.9, 5.4, 2.1 Hz, 1H), 3.27 (dd, *J* = 9.5, 5.9 Hz, 1H), 2.82 (dd, *J* = 9.5, 5.4 Hz, 1H), 1.12 (t, *J* = 7.1 Hz, 3H).

^{13}C NMR (101 MHz, $CDCl_3$) δ 195.9 (CH), 167.4 (Cq), 165.4 (Cq), 150.1 (Cq), 146.3 (Cq), 145.6 (Cq), 120.9 (CH), 120.2 (CH), 107.4 (CH), 62.1 (CH_2), 33.2 (CH), 29.6 (CH), 24.4 (CH), 14.1 (CH_3).

The enantiomeric excess was determined by HPLC using a Chiralpak AY-H column (hexane/*i*PrOH = 50:50, flow rate 1.0 mL/min, λ = 210 nm): t_r (S) = 31.8, t_r (R) = 35.2, 81% (R) / 87% (S) *ee*.

$[\alpha]_D^{22}$ = -45.8° (c = 0.4, $CHCl_3$) **(S catalyst)**

HRMS *m/z* (ESI+) Exact mass calculated for $C_{14}H_{13}N_2O_6$ $[M+H]^+$: 305.0768, found: 305.0763.

Compound 5a

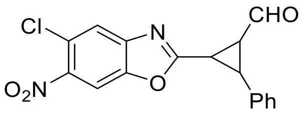

The reaction was performed following the general procedure adding: 2-(diphenyl((trimethylsilyl)oxy)methyl)pyrrolidine (26 mg, 0.081 mmol, 20 mol% equiv), cynnamaldehyde (107 mg, 0.810 mmol, 2 equiv), 5-chloro-2-(chloromethyl)-6-nitrobenzoxazole (100 mg, 0.405 mmol, 1 equiv), $Pd(OAc)_2$ (4.5 mg, 0.020 mmol, 5 mol%

equiv), CH_3CN (1 mL) and 2,6-lutidine (43 mg, 0.405 mmol, 1 equiv). The crude was purified by flash column chromatography (hexane/EtOAc 10:1) to obtain 106 mg of the desired products as yellow oil. Yield: 68%. The diastereomeric ratio was calculated based on the isolated products after column chromatography. d.r.: 10.5:3.3:1

(1*S*,2*S*,3*R*)-2-(5-chloro-6-nitrobenzoxazol-2-yl)-3-phenylcyclopropane-1-carbaldehyde

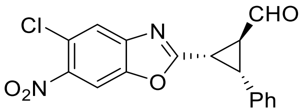

major

IR (liquid film): 3101, 2922, 2849, 1713 (CHO), 1564 (aromatic NO_2), 1531, 1344 (aromatic NO_2), 752 cm^{-1}.

^1H NMR (400 MHz, $CDCl_3$) δ 9.89 (d, *J* = 2.7 Hz, 1H), 7.89 (s, 1H), 7.69 (s, 1H), 7.22 – 7.17 (m, 5H), 3.56 (ddd, *J* = 6.1, 5.1, 2.8 Hz, 1H), 3.44 – 3.33 (m, 2H).

^{13}C NMR (101 MHz, $CDCl_3$) δ 197.2 (CH), 167.3 (Cq), 148.2 (Cq), 144.71 (Cq), 144.67 (Cq), 132.8 (Cq), 128.8 (CH), 128.7 (CH), 128.1 (CH), 123.8 (Cq), 122.2 (CH), 108.4 (CH), 35.0 (CH), 34.5 (CH), 26.1 (CH).

The enantiomeric excess was determined by HPLC using a Chiralpak IC column (hexane/*i*PrOH = 85:15, flow rate 1.0 mL/min, λ = 210 nm): t_r (S) = 30.7, t_r (R) = 33.9, >99% (R and S) *ee*.

$[\alpha]_D^{21}$ = +99.6° (c = 0.4, $CHCl_3$) **(R catalyst)**

HRMS *m/z* (ESI+) Exact mass calculated for $C_{17}H_{12}ClN_2O_4$ $[M+H]^+$: 343.0480, found: 343.0472.

(1*S*,2*S*,3*S*)-2-(5-chloro-6-nitrobenzoxazol-2-yl)-3-phenylcyclopropane-1-carbaldehyde

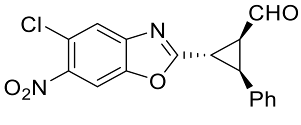

minor

Mixture of minor and minor':

^1H NMR (400 MHz, $CDCl_3$) δ 9.57 (d, *J* = 5.5 Hz, 1H'), 9.06 (d, *J* = 4.9 Hz, 1H), 7.99 (s, 1H + 1H'), 7.73 (s, 1H'), 7.71 (s, 1H), 7.29 – 7.13 (m, 5H + 5H'), 3.63 (dd, *J* = 6.3, 6.3 Hz, 1H'), 3.59 – 3.48 (m, 2H), 3.05 (dd, *J* = 8.9, 6.5 Hz, 1H'), 2.97 (dt, *J* = 9.7, 4.9 Hz, 1H), 2.70 (ddd, *J* = 8.9, 6.2, 5.7 Hz, 1H').

^{13}C NMR (101 MHz, $CDCl_3$) δ 196.9 (CH'), 195.9 (CH), 169.9 (Cq), 168.2 (Cq'), 148.4 (Cq'), 148.3 (Cq), 145.2 (Cq), 145.0 (Cq'), 144.97 (Cq'), 144.8 (Cq), 135.8 (Cq'), 132.8 (Cq), 129.3 (CH'), 129.2 (CH), 129.1 (CH), 128.4 (CH), 128.3 (CH'), 126.8 (Cq), 124.2, 122.5 (CH'), 122.3 (CH), 108.8 (CH'), 108.7 (CH), 39.5 (CH'), 38.9 (CH), 35.6 (CH), 32.5 (CH'), 26.7 (CH'), 22.1 (CH).

The enantiomeric excess was determined by HPLC using a Chiralpak IC column (hexane/*i*PrOH = 85:15, flow rate 1.0 mL/min, λ = 210 nm): t_r (S) = 37.7, t_r (R) = 40.1, 90% (R) *ee*.

$[\alpha]_D^{21}$ = -15.4° (c = 0.6, $CHCl_3$) **(S catalyst)**

HRMS *m/z* (ESI+) Exact mass calculated for $C_{17}H_{12}ClN_2O_4$ $[M+H]^+$: 343.0480, found: 343.0476.

Compound 5c

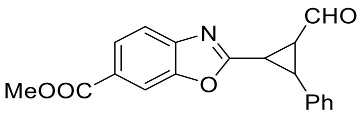

The reaction was performed following the general procedure adding: 2-(diphenyl((trimethylsilyl)oxy)methyl)pyrrolidine (29mg, 0.089 mmol, 20 mol% equiv), cynnamaldehyde (117 mg, 0.886 mmol, 2 equiv), methyl 2-(chloromethyl)benzoxazole-6-carboxylate (100 mg, 0.443 mmol, 1 equiv), $Pd(OAc)_2$ (5 mg, 0.022 mmol, 5 mol% equiv), CH_3CN (1 mL) and 2,6-lutidine (48 mg, 0.443 mmol, 1 equiv). The crude was purified by flash column chromatography (hexane/EtOAc 5:1) to obtain 111 mg of the desired products as yellow oil. Yield: 78%. The diastereomeric ratio was calculated based on the isolated products after column chromatography. d.r.: 4.5:1.9:1

methyl 2-((1*R*,2*R*,3*S*)-2-formyl-3-phenylcyclopropyl)benzoxazole-6-carboxylate

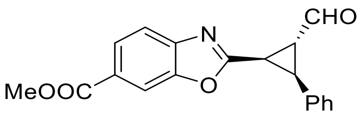

major

IR (liquid film): 3061, 2952, 2847, 1717 (CHO, CO ester), 1434, 1287 (ester), 1269 (ester), 746 cm^{-1}.

^{1}H NMR (400 MHz, $CDCl_3$) δ 9.87 (d, *J* = 2.9 Hz, 1H), 8.00 (d, *J* = 0.9 Hz, 1H), 7.98 (dd, *J* = 8.3, 1.5 Hz, 1H), 7.58 (d, *J* = 8.3 Hz, 1H), 7.24 – 7.14 (m, 5H), 3.91 (s, 3H), 3.54 (ddd, *J* = 5.5, 5.5, 2.9 Hz, 1H), 3.41 – 3.33 (m, 2H).

^{13}C NMR (101 MHz, $CDCl_3$) δ 197.6 (CH), 166.5 (Cq), 164.3 (Cq), 150.4 (Cq), 144.8 (Cq), 133.2 (Cq), 128.7 (CH), 128.4 (CH), 127.7 (CH), 126.9 (Cq), 126.1 (CH), 119.2 (CH), 111.9 (CH), 52.4 (CH_3), 34.4 (CH), 34.3 (CH), 26.2 (CH).

The enantiomeric excess was determined by HPLC using a Chiralpak AY-H column (hexane/*i*PrOH = 80:20, flow rate 1.0 mL/min, λ = 230 nm): t_r (S) = 21.1, t_r (R) = 20.0, 98% (R) / 98% (S) *ee*.

$[\alpha]_D^{22}$ = -104.6° (c = 0.8, $CHCl_3$) **(S catalyst)**

$[\alpha]_D^{22}$ = +105.8° (c = 0.4, $CHCl_3$) **(R catalyst)**

HRMS *m/z* (ESI+) Exact mass calculated for $C_{19}H_{16}NO_4$ $[M+H]^+$: 322.1074, found: 322.1079.

methyl 2-((1*R*,2*R*,3*S*)-2-formyl-3-phenylcyclopropyl)benzoxazole-6-carboxylate

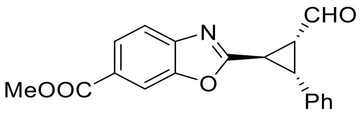

minor

Mixture of minor and minor' (inverted compared to the previous products as in this case the minor' is prevalent):

^{1}H NMR (400 MHz, $CDCl_3$) δ 9.64 (d, *J* = 6.0 Hz, 1H'), 9.13 (d, *J* = 5.2 Hz, 1H), 8.22 – 8.18 (m, 1H + 1H'), 8.09 (d, *J* = 8.4 Hz, 1H + d, *J* = 8.4 Hz, 1H'), 7.71 (d, *J* = 8.3, Hz, 1H'), 7.70 (d, *J* = 8.3, Hz, 1H), 7.41 – 7.22 (m, 5H + 5H'), 3.97 (s, 3H + 3H'), 3.76 (dd, *J* = 6.3, 6.3 Hz, 1H'), 3.69 – 3.59 (m, 2H), 3.17 (dd, *J* = 8.9, 6.5 Hz, 1H'), 3.04 (ddd, *J* = 9.7, 5.4, 5.0 Hz, 1H), 2.73 (ddd, *J* = 8.9, 6.0, 6.0 Hz, 1H').

^{13}C NMR (101 MHz, $CDCl_3$) δ 197.3 (CH'), 196.3 (CH), 166.9 (Cq), 166.53 (Cq'), 166.50 (Cq), 165.2 (Cq'), 150.4 (Cq'), 150.3 (Cq), 145.1 (Cq), 144.9 (Cq'), 136.1 (Cq'), 133.1, 129.0 (CH'), 129.0 (CH + CH'), 128.9 (CH), 128.0 (CH), 127.9 (CH'), 127.3 (Cq), 127.1 (Cq'), 126.6 (CH'), 126.5 (CH), 119.4 (CH'), 119.1 (CH), 112.14 (CH'), 112.07 (CH), 52.4 (CH_3 + CH_3'), 39.5 (CH'), 38.5 (CH), 34.8 (CH), 31.7 (CH'), 26.6 (CH'), 22.1 (CH).

HRMS *m/z* (ESI+) Exact mass calculated for $C_{19}H_{16}NO_4$ $[M+H]^+$: 322.1074, found: 322.1079.

Compound 5b

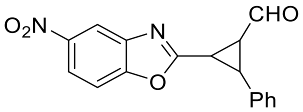

The reaction was performed following the general procedure adding: 2-(diphenyl((trimethylsilyl)oxy)methyl)pyrrolidine (31mg, 0.094 mmol, 20 mol% equiv), cynnamaldehyde (124 mg, 0.940 mmol, 2 equiv), 2-(chloromethyl)-5-nitrobenzoxazole (100 mg, 0.470 mmol, 1 equiv), $Pd(OAc)_2$ (5 mg, 0.024 mmol, 5 mol% equiv), CH_3CN (1 mL) and 2,6-lutidine (50 mg, 0.470 mmol, 1 equiv). The crude was purified by flash column chromatography (hexane/EtOAc 3:1) to obtain 80 mg of the desired products as dark yellow oil. Yield: 55%. The diastereomeric ratio was calculated based on the isolated products after column chromatography. d.r.: 6.2:1.3:1

(1*R*,2*R*,3*S*)-2-(5-nitrobenzoxazol-2-yl)-3-phenylcyclopropane-1-carbaldehyde

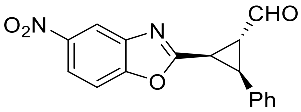

major

IR (liquid film): 3105, 2923, 2852, 1714 (CHO), 1526, 1347 (aromatic NO_2), 743 cm^{-1}.

^1H NMR (400 MHz, $CDCl_3$) δ 9.89 (d, *J* = 2.8 Hz, 1H), 8.44 (d, *J* = 2.3 Hz, 1H), 8.19 (dd, *J* = 8.9, 2.3 Hz, 1H), 7.42 (d, *J* = 9.0 Hz, 1H), 7.23 – 7.14 (m, 5H), 3.56 (ddd, *J* = 5.5, 5.4, 2.8 Hz, 1H), 3.38 (d, *J* = 5.5 Hz, 2H).

^{13}C NMR (101 MHz, $CDCl_3$) δ 194.5 (CH), 162.0 (Cq), 151.3 (Cq), 142.4 (Cq), 138.4 (Cq), 130.1 (Cq), 125.8 (CH), 125.6 (CH), 125.0 (CH), 118.1 (CH), 113.2 (CH), 107.6 (CH), 31.8 (CH), 31.4 (CH), 23.1 (CH).

The enantiomeric excess was determined by HPLC using a Chiralpak OD-H column (hexane/*i*PrOH = 70:30, flow rate 1.0 mL/min, λ = 210 nm): t_r (S) = 20.6, t_r (R) = 19.8, 99% (R) / 98% (S) *ee*.

$[\alpha]_D^{22}$ = -41.6° (c = 0.8, $CHCl_3$) **(S catalyst)**

$[\alpha]_D^{22}$ = +36.2° (c = 0.5, $CHCl_3$) **(R catalyst)**

HRMS *m/z* (ESI+) Exact mass calculated for $C_{17}H_{13}N_2O_4$ $[M+H]^+$: 309.0870, found: 309.0868.

(1*R*,2*R*,3*R*)-2-(5-nitrobenzoxazol-2-yl)-3-phenylcyclopropane-1-carbaldehyde

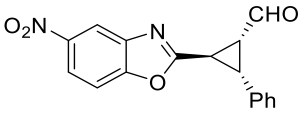

minor

Mixture of minor and minor':

^{1}H NMR (400 MHz, $CDCl_3$) δ 9.66 (d, *J* = 5.7 Hz, 1H'), 9.15 (d, *J* = 5.0 Hz, 1H), 8.57 (d, *J* = 2.2 Hz, 1H'), 8.55 (d, *J* = 2.2 Hz, 1H), 8.31 (dd, *J* = 8.9, 2.5 Hz, 1H + dd, *J* = 8.9, 2.5 Hz, 1H'), 7.62 (bd, *J* = 8.9 Hz, 1H + 1H'), 7.41 – 7.24 (m, 10H), 3.75 (dd, *J* = 6.3, 6.3 Hz, 1H'), 3.69 – 3.59 (m, 2H), 3.16 (dd, *J* = 8.9, 6.5 Hz, 1H'), 3.06 (ddd, *J* = 9.7, 5.0, 4.9 Hz, 1H), 2.77 (ddd, *J* = 8.9, 5.9, 5.9 Hz, 1H').

^{13}C NMR (101 MHz, $CDCl_3$) δ 197.2 (CH), 196.1 (CH), 167.6 (Cq), 165.9 (Cq), 154.4 (Cq), 154.3 (Cq), 145.7 (Cq), 141.9 (Cq), 141.7 (Cq), 136.0 (Cq), 132.9 (Cq), 129.2 (CH), 129.1 (CH), 129.0 (CH), 128.3 (CH), 128.2 (CH), 126.8 (CH), 121.4 (CH), 121.2 (CH), 116.4 (CH), 116.1 (CH), 110.9 (CH), 110.8 (CH), 39.4 (CH), 38.6 (CH), 35.2 (CH), 32.1 (CH), 26.6 (CH), 22.0 (CH).

HRMS *m/z* (ESI+) Exact mass calculated for $C_{17}H_{13}N_2O_4$ $[M+H]^+$: 309.0870, found: 309.0877.

Compound 5d

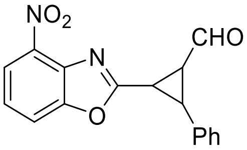

The reaction was performed following the general procedure adding: 2-(diphenyl((trimethylsilyl)oxy)methyl)pyrrolidine (31mg, 0.094 mmol, 20 mol% equiv), cynnamaldehyde (124 mg, 0.940 mmol, 2 equiv), 2-(chloromethyl)-4-nitrobenzoxazole (100 mg, 0.470 mmol, 1 equiv), $Pd(OAc)_2$ (5 mg, 0.024 mmol, 5 mol% equiv), CH_3CN (1 mL) and 2,6-lutidine (50 mg, 0.470 mmol, 1 equiv). The crude was purified by flash column chromatography (hexane/EtOAc 3:1) to obtain 74 mg of the desired products as dark yellow oil. Yield: 51%. The diastereomeric ratio was calculated based on the isolated products after column chromatography. d.r.: 2.3:1.6:1

(1*R*,2*R*,3*S*)-2-(4-nitrobenzoxazol-2-yl)-3-phenylcyclopropane-1-carbaldehyde

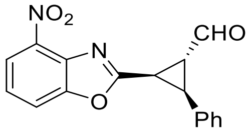

major

Mixture of major (m) and minor:

IR (liquid film): 3020, 2852, 1713 (CHO), 1561 (aromatic NO_2), 1526, 1343 (aromatic NO_2), 1214, 747 cm^{-1}.

^1H NMR (400 MHz, $CDCl_3$) δ 9.88 (d, *J* = 2.8 Hz, $1H^m$), 9.14 (d, *J* = 5.0 Hz, 1H), 8.20 (d, *J* = 8.3 Hz, 1H), 8.10 (d, *J* = 8.2 Hz, $1H^m$), 7.85 (d, *J* = 8.1 Hz, 1H), 7.61 (d, *J* = 8.1 Hz, $1H^m$), 7.49 (dd, *J* = 8.2 Hz, 1H), 7.39 – 7.15 (m, $6H^m$ + 5H), 3.77 – 3.72 (m, 2H), 3.64 (ddd, *J* = 5.6, 5.6, 2.9 Hz, $1H^m$), 3.50 (dd, *J* = 9.8, 5.1 Hz, $1H^m$), 3.44 – 3.34 (m, $1H^m$), 3.12 (ddd, *J* = 9.2, 5.2, 5.2Hz, 1H).

^{13}C NMR (101 MHz, $CDCl_3$) δ 197.3 (CH^m), 195.9 (CH), 167.9 (Cq), 165.5 (Cq^m), 152.3 (Cq), 152.2 (Cq^m), 138.8 (Cq), 138.7 (Cq^m), 136.1 (Cq), 135.7 (Cq^m), 132.9 (Cq^m), 132.8 (Cq), 129.0 (CH), 128.9 (CH), 128.7 (CH^m), 128.4 (CH^m), 128.1 (CH), 127.8 (CH^m), 124.4 (CH), 124.2 (CH^m), 121.1 (CH), 120.8 (CH^m), 116.4 (CH), 116.2 (CH^m), 38.6 (CH), 35.1 (CH), 34.7 (CH^m), 34.5 (CH^m), 26.2 (CH^m), 22.1 (CH).

The enantiomeric excess was determined by HPLC using a Chiralpak AY-H column (hexane/*i*PrOH = 70:30, flow rate 1.0 mL/min, λ = 210 nm): t_r (S) = 37.7, t_r (R) = 21.7, >99% (R and S) *ee*.

$[\alpha]_D^{22}$ = -5.2° (c = 0.8, $CHCl_3$) **(*S* catalyst)**

$[\alpha]_D^{22}$ = +6.6° (c = 0.7, $CHCl_3$) **(*R* catalyst)**

HRMS *m/z* (ESI+) Exact mass calculated for $C_{17}H_{13}N_2O_4$ $[M+H]^+$: 309.0870, found: 309.0871.

(1*R*,2*R*,3*R*)-2-(4-nitrobenzoxazol-2-yl)-3-phenylcyclopropane-1-carbaldehyde

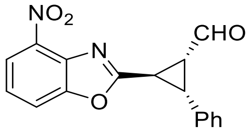

minor

Mixture of $major^m$, minor, minor' and traces of starting benzoxazole:

^1H NMR (400 MHz, $CDCl_3$) δ 9.88 (d, *J* = 2.8 Hz, $1H^m$), 9.73 (d, *J* = 5.6 Hz, 1H'), 9.14 (d, *J* = 5.0 Hz, 1H), 8.20 (d, *J* = 8.3 Hz, 1H), 8.10 (d, *J* = 8.2 Hz, $4H^m$), 7.85 (ddd, *J* = 8.1, 4.7, 0.8 Hz, 4H + '), 7.64 – 7.57 (m, $4H^m$), 7.50 (td, *J* = 8.2, 3.3 Hz, 5H +'), 7.42 – 7.13 (m, 51H), 3.85 (dd, *J* = 6.3, 6.3 Hz, 1H'), 3.78 – 3.72 (m, 2H), 3.64 (ddd, *J* = 6.0, 5.2, 2.9 Hz, $1H^m$), 3.51 (dd, *J* = 9.8,

5.1 Hz, 1H^m), 3.45 – 3.34 (m, 1H^m), 3.29 (dd, *J* = 8.9, 6.6 Hz, 1H'), 3.12 (ddd, *J* = 9.2, 5.2, 5.2 Hz, 1H), 2.79 (ddd, *J* = 8.9, 5.9, 5.9 Hz, 1H').

^{13}C NMR (101 MHz, $CDCl_3$) δ 197.3 (CH^m), 197.1 (CH'), 195.9 (CH), 167.9 (Cq), 166.4 (Cq'), 165.5 (Cq^m), 152.3 (Cq'), 152.3 (Cq), 152.2 (Cq^m), 138.8 (Cq), 138.6 (Cq^m), 136.1 (Cq), 135.9 (Cq'), 135.8 (Cq'), 135.7 (Cq^m), 132.9 (Cq^m), 132.8 (Cq), 129.0 (CH), 128.97 (CH'), 128.90 (CH), 128.7 (CH^m), 128.4 (CH^m), 128.1 (CH), 128.0 (CH'), 127.8 (CH^m), 126.6 (CH'), 125.7 (CH'), 124.6 (CH'), 124.4 (CH), 124.2 (CH^m), 121.1 (CH), 120.8 (CH^m), 117.2 (CH'), 116.5 (CH'), 116.4 (CH), 116.2 (CH^m), 39.7 (CH'), 38.6 (CH), 35.9 (CH'), 35.1 (CH), 34.7 (CH^m), 34.5 (CH^m), 32.1 (CH'), 26.6 (CH'), 26.3 (CH^m), 22.1 (CH).

HRMS *m/z* (ESI+) Exact mass calculated for $C_{17}H_{13}N_2O_4$ [M+H]$^+$: 309.0870, found: 309.0871.

Compound 5e

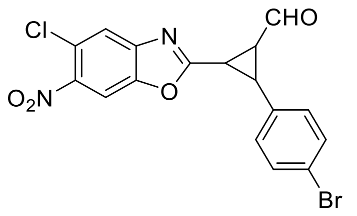

The reaction was performed following the general procedure adding: 2-(diphenyl((trimethylsilyl)oxy)methyl)pyrrolidine (26 mg, 0.081 mmol, 20 mol% equiv), (*E*)-3-(4-bromophenyl)acrylaldehyde (171 mg, 0.810 mmol, 2 equiv), 5-chloro-2-(chloromethyl)-6-nitrobenzoxazole (100 mg, 0.405 mmol, 1 equiv), $Pd(OAc)_2$ (4.5 mg, 0.020 mmol, 5 mol% equiv), CH_3CN (1 mL) and 2,6-lutidine (43 mg, 0.405 mmol, 1 equiv). The crude was purified by flash column chromatography (hexane/EtOAc 4:1) to obtain 145 mg of the desired products as yellow oil. Yield: 85%. The diastereomeric ratio was calculated based on the isolated products after column chromatography. d.r.: 8.1:4.8:1

(1*R*,2*S*,3*R*)-2-(4-bromophenyl)-3-(5-chloro-6-nitrobenzoxazol-2-yl)cyclopropane-1-carbaldehyde

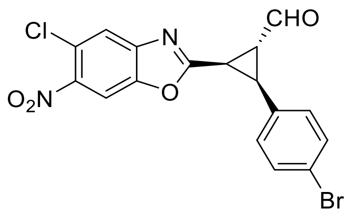

major

IR (liquid film): 3101, 2923, 2850, 1713 (CHO), 1565 (aromatic NO_2), 1531, 1446, 1345 (aromatic NO_2), 755 cm^{-1}.

^{1}H NMR (400 MHz, $CDCl_3$) δ 9.89 (d, *J* = 2.6 Hz, 1H), 7.92 (s, 1H), 7.69 (s, 1H), 7.33 (d, *J* = 8.4 Hz, 2H), 7.09 (d, *J* = 8.4 Hz, 2H), 3.53 (dd, *J* = 5.6, 5.6, 2.6 Hz, 1H), 3.35 (ddd, *J* = 9.8, 5.7 Hz, 2H).

^{13}C NMR (101 MHz, $CDCl_3$) δ 196.8 (CH), 166.7 (Cq), 148.1 (Cq), 144.6 (Cq), 144.4 (Cq), 131.7 (CH), 130.3 (CH), 123.8 (Cq), 122.2 (CH), 122.1 (Cq), 108.4 (CH), 34.2 (CH), 34.2 (CH), 25.9 (CH).

The enantiomeric excess was determined by HPLC using a Chiralpak OD-H column (hexane/*i*PrOH = 85:15, flow rate 1.0 mL/min, λ = 210 nm): t_r (S) = 39.2, t_r (R) = 30.6, 98% (R) / 97% (S) *ee*.

$[\alpha]_D^{21}$ = -153.8° (c = 0.6, $CHCl_3$) **(S catalyst)**

HRMS *m/z* (ESI+) Exact mass calculated for $C_{17}H_{11}BrClN_2O_4$ $[M+H]^+$: 420.9585, found: 420.9587.

(1*R*,2*R*,3*R*)-2-(4-bromophenyl)-3-(5-chloro-6-nitrobenzoxazol-2-yl)cyclopropane-1-carbaldehyde

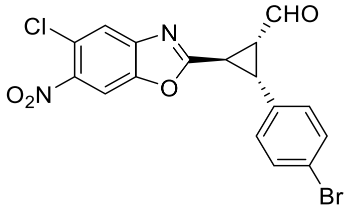

minor

Mixture of minor and minor':

^{1}H NMR (400 MHz, $CDCl_3$) δ 9.66 (d, *J* = 5.3 Hz, 1H'), 9.26 (d, *J* = 4.2 Hz, 1H), 8.09 (s, 1H + 1H'), 7.83 (s, 1H'), 7.81 (s, 1H), 7.51 (d, *J* = 8.3 Hz, 2H'), 7.49 (d, *J* = 8.3 Hz, 2H), 7.23 (d, *J* = 8.4 Hz, 2H), 7.13 (d, *J* = 8.4 Hz, 2H'), 3.69 (dd, *J* = 6.3, 6.3 Hz, 1H'), 3.58 (bd, *J* = 6.8 Hz, 2H), 3.15 – 3.08 (m, 1H + 1H'), 2.80 – 2.72 (m, 1H').

^{13}C NMR (101 MHz, $CDCl_3$) δ 196.2, 195.1, 169.4, 167.6, 148.1, 144.9, 144.7, 144.7, 134.7, 132.2, 132.0, 131.6, 130.6, 128.3, 124.1, 122.4, 122.3, 122.1, 122.0, 108.6, 108.5, 39.0, 38.5, 35.0, 31.6, 26.4, 22.0.

HRMS *m/z* (ESI+) Exact mass calculated for $C_{17}H_{11}BrClN_2O_4$ $[M+H]^+$: 420.9585, found: 420.9585.

Compound 5f

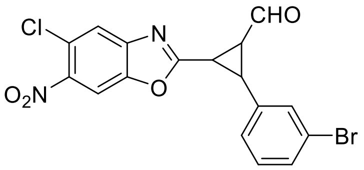

The reaction was performed following the general procedure adding: 2-(diphenyl((trimethylsilyl)oxy)methyl)pyrrolidine (26 mg, 0.081 mmol, 20 mol% equiv), (*E*)-3-(3-bromophenyl)acrylaldehyde (171 mg, 0.810 mmol, 2 equiv), 5-chloro-2-(chloromethyl)-6-nitrobenzoxazole (100 mg, 0.405 mmol, 1 equiv), $Pd(OAc)_2$ (4.5 mg, 0.020 mmol, 5 mol% equiv), CH_3CN (1 mL) and 2,6-lutidine (43 mg, 0.405 mmol, 1 equiv). The crude was purified by flash column chromatography (hexane/EtOAc 4:1) to obtain 112 mg of the desired products as yellow oil. Yield: 66%. The diastereomeric ratio was calculated based on the isolated products after column chromatography. d.r.: 17.4:6.3:1

(1*R*,2*S*,3*R*)-2-(3-bromophenyl)-3-(5-chloro-6-nitrobenzoxazol-2-yl)cyclopropane-1-carbaldehyde

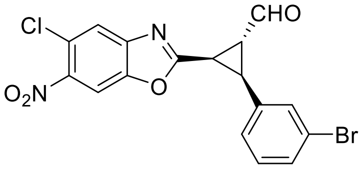
major

IR (liquid film): 3101, 2922, 2850, 1714 (CHO), 1564(aromatic NO_2), 1531, 1446, 1344(aromatic NO_2), 754 cm^{-1}.

^{1}H NMR (400 MHz, $CDCl_3$) δ 9.92 (d, *J* = 2.5 Hz, 1H), 7.94 (s, 1H), 7.72 (s, 1H), 7.44 (dd, *J* = 1.7, 1.7 Hz, 1H), 7.33 (ddd, *J* = 7.7, 1.6, 1.6 Hz, 1H), 7.14 – 7.05 (m, 2H), 3.56 (ddd, *J* = 5.6, 5.6, 2.5 Hz, 1H), 3.41 – 3.34 (m, 2H).

^{13}C NMR (101 MHz, $CDCl_3$) δ 196.7 (CH), 166.6 (Cq), 148.1 (Cq), 144.7 (Cq), 144.4 (Cq), 134.9 (Cq), 132.0 (CH), 131.2 (CH), 130.0 (CH), 127.2 (CH), 123.8, 122.5 (Cq), 122.2 (CH), 108.4 (CH), 34.2 (CH), 34.1 (CH), 26.0 (CH).

The enantiomeric excess was determined by HPLC using a Chiralpak OD-H column (hexane/*i*PrOH = 70:30, flow rate 1.0 mL/min, λ = 210 nm): t_r (S) = 38.2, t_r (R) = 32.9, 91% (R) / 97% (S) *ee*.

$[\alpha]_D^{21}$ = -107.2° (c = 1.3, $CHCl_3$) **(*S* catalyst)**

$[\alpha]_D^{21}$ = +97.9° (c = 2.0, $CHCl_3$) (***R* catalyst)**

HRMS *m/z* (ESI+) Exact mass calculated for $C_{17}H_{11}BrClN_2O_4$ $[M+H]^+$: 420.9585, found: 420.9581.

(1*R*,2*R*,3*R*)-2-(3-bromophenyl)-3-(5-chloro-6-nitrobenzoxazol-2-yl)cyclopropane-1-carbaldehyde

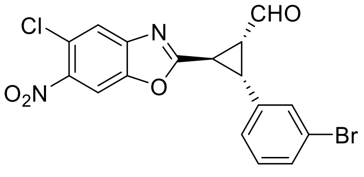

minor

Mixture of minor and minor':

^{1}H NMR (400 MHz, $CDCl_3$) δ 9.67 (d, *J* = 5.3 Hz, 1H'), 9.26 (d, *J* = 4.3 Hz, 1H), 8.10 (s, 1H + 1H'), 7.84 (s, 1H'), 7.82 (s, 1H), 7.54 (bs, 1H), 7.49 – 7.43 (m, 1H + 1H'), 7.41 (t, *J* = 1.7 Hz, 1H'), 7.32 – 7.17 (m, 2H + 2H'), 3.71 (dd, *J* = 6.3, 6.3 Hz, 1H'), 3.64 – 3.60 (m, 2H), 3.19 – 3.08 (m, 1H + 1H'), 2.84 – 2.76 (ddd, *J* = 9.0, 6.0, 5.4 Hz, 1H').

^{13}C NMR (101 MHz, $CDCl_3$) δ 196.2 (CH'), 195.0 (CH), 169.3 (Cq), 167.5 (Cq'), 148.1 (Cq'), 148.0 (Cq), 144.9 (Cq), 144.7 (Cq'), 138.0 (Cq'), 134.8 (Cq), 132.1 (CH), 131.4 (CH), 131.2 (CH'), 130.6 (CH'), 130.4 (CH), 129.8 (CH'), 127.6 (CH), 125.4 (CH'), 124.1 (Cq), 124.0 (Cq'), 123.1 (Cq'), 122.9 (Cq), 122.4 (CH'), 122.1 (CH), 108.6, 108.6, 38.9 (CH'), 38.5 (CH), 34.8 (CH), 31.5 (CH'), 26.4 (CH'), 21.9 (CH).

HRMS *m/z* (ESI+) Exact mass calculated for $C_{17}H_{11}BrClN_2O_4$ $[M+H]^+$: 420.9585, found: 420.9589.

16. Notes:

Thin layer chromatography (TLC) was performed on Merck TLC Silicagel 60 F_{254}. Product spots were visualized by UV-light at 254nm, and developed with potassium permanganate. Column chromatography was effectuated using silica gel (Geduran Si60, 40-63µm).

Infra-red spectra were recorded on a Nicolet 280 FT-IR; the IR analyses were performed as a liquid IR with the compounds dissolved in $CHCl_3$.

^{1}H-NMR, ^{13}C-NMR, ^{19}F-NMR were recorded with a Bruker DPX400 NMR.

High resolution mass spectra were recorded using a MaXis (Bruker Daltonics, Bremen, Germany) mass spectrometer equipped with a Time of Flight (TOF) analyser.

17. References

[1] W. S. Saari, J. J. S. Wai, T. E. Fisher, C. M. Thomas, J. M. Hoffman, C. S. Rooney, A. M. Smith, J. H. Jones, D. L. Bamberger, M. E. Goldman, et al., *J. Med. Chem* **1992**, *2*, 232–233.

[2] Y. Xie, L. Li, *Tetrahedron Lett.* **2014**, *55*, 3892–3895.

[3] A. Nordqvist, C. Björkelid, M. Andaloussi, A. M. Jansson, S. L. Mowbray, A. Karlén, M. Larhed, *J. Org. Chem.* **2011**, *76*, 8986–8998.

[4] T.-S. Jiang, J.-H. Li, *Chem. Commun. (Camb).* **2009**, 7236–7238.

[5] H. Chen, H. Jiang, C. Cai, J. Dong, W. Fu, *Org. Lett.* **2011**, *13*, 992–994.

[6] J. Zhu, J. Liu, R. Ma, H. Xie, J. Li, H. Jiang, W. Wang, *Adv. Synth. Catal.* **2009**, *351*, 1229–1232.

[7] F. Cermola, *J. Chem. Res.* **2005**, *2005*, 677–681.

[8] E. Kolehmainen, K. Laihia, P. Manttari, *Magn. Reson. Chem.* **1991**, *29*, 1109–1113.

18. HPLC traces

The racemic mixtures used in the HPLC traces were prepared by mixing the product obtained using the organic catalyst with the R configuration and the product obtained using the organic catalyst with the S configuration.

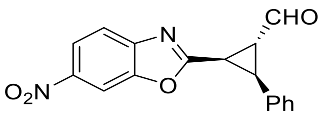

4a major

Mixture of S and R: (IA, 85.15, 210, 1ml/min)

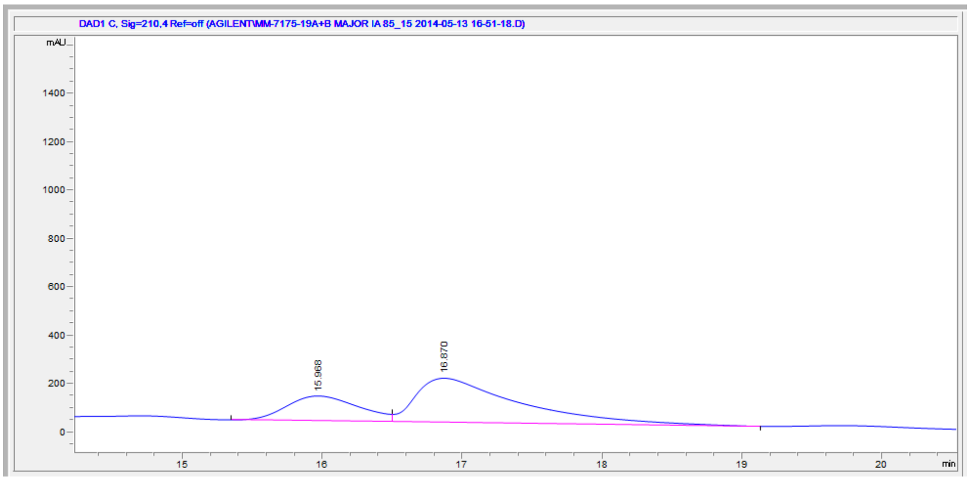

Peak #	RetTime [min]	Type	Width [min]	Area [mAU*s]	Height [mAU]	Area %
1	15.968	BV	0.5569	3712.38623	102.91290	28.3069
2	16.870	VB	0.7268	9402.38672	182.58131	71.6931

Chiral S:

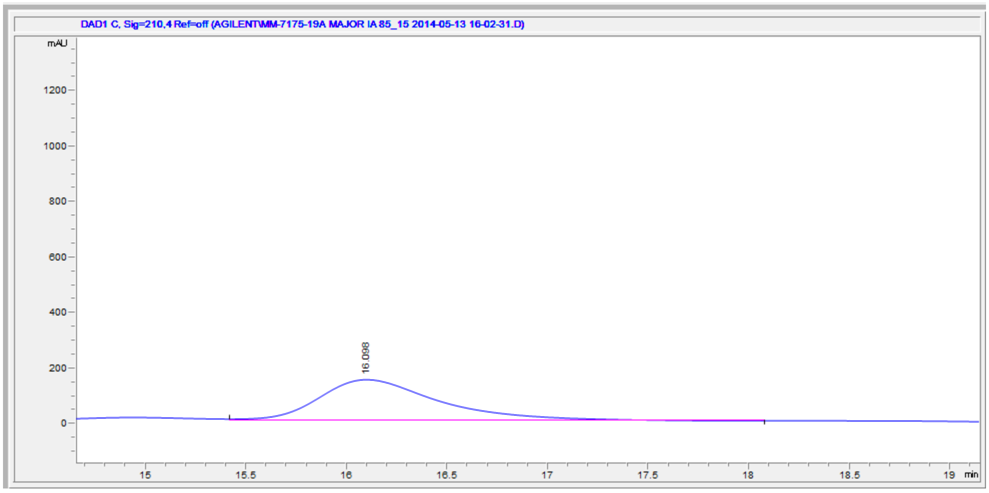

Peak #	RetTime [min]	Type	Width [min]	Area [mAU*s]	Height [mAU]	Area %
1	16.098	VB	0.6283	6266.22656	147.88811	100.0000

Chiral R:

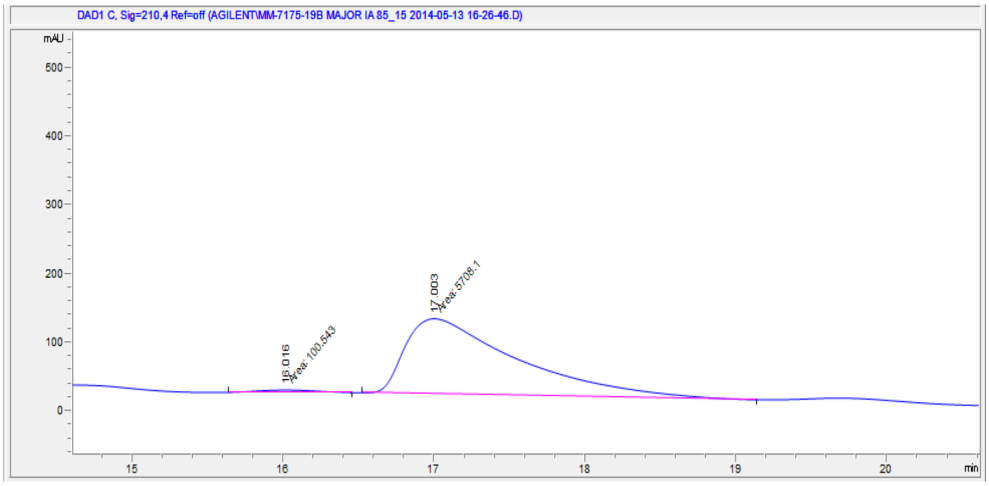

Peak #	RetTime [min]	Type	Width [min]	Area [mAU*s]	Height [mAU]	Area %
1	16.016	MM	0.4590	100.54274	3.65084	1.7309
2	17.003	MM	0.8683	5708.09961	109.55972	98.2691

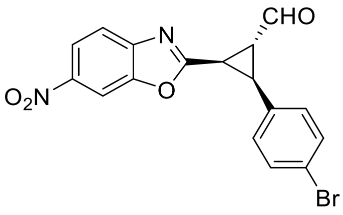

4b major

Mixture of S and R: (OD-H, 85.15, 210 nm, 1ml/min)

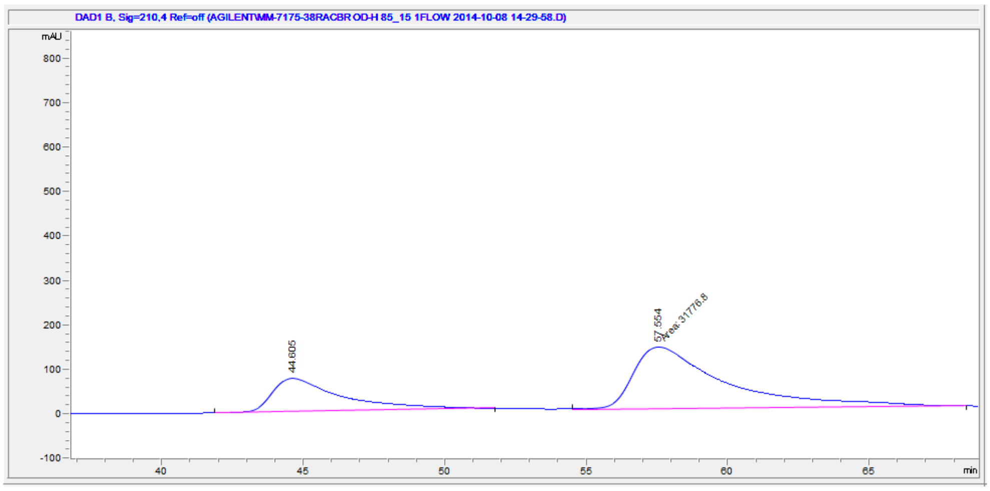

Peak #	RetTime [min]	Type	Width [min]	Area [mAU*s]	Height [mAU]	Area %
1	44.605	BB	2.2703	1.28171e4	75.13073	28.7418
2	57.554	MM	3.7924	3.17768e4	139.64941	71.2582

Chiral S:

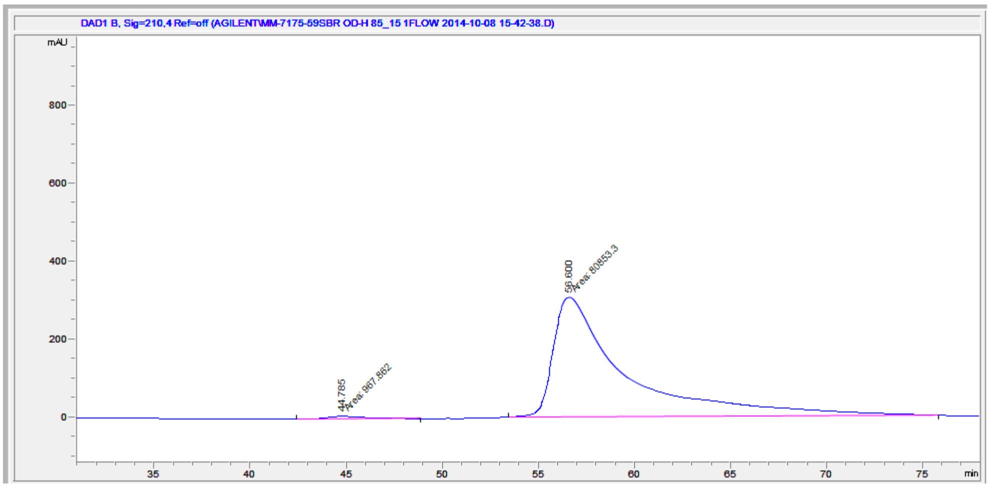

Peak #	RetTime [min]	Type	Width [min]	Area [mAU*s]	Height [mAU]	Area %
1	44.785	MM	2.3673	967.86176	6.81401	1.1829
2	56.600	MM	4.3933	8.08533e4	306.72745	98.8171

Chiral R:

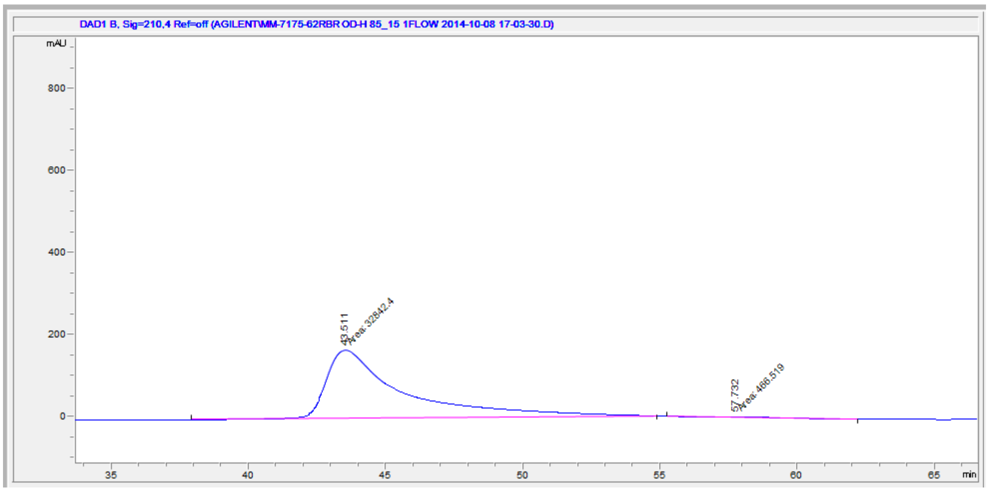

Peak #	RetTime [min]	Type	Width [min]	Area [mAU*s]	Height [mAU]	Area %
1	43.511	MM	3.2714	3.28424e4	167.31866	98.5994
2	57.732	MM	3.1711	466.51904	2.45191	1.4006

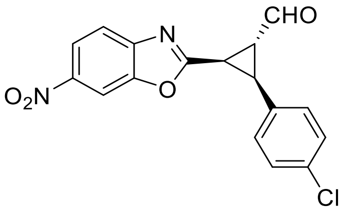

4c major

Mixture of S and R: (OD-H, 80.20, 250 nm, 1ml/min)

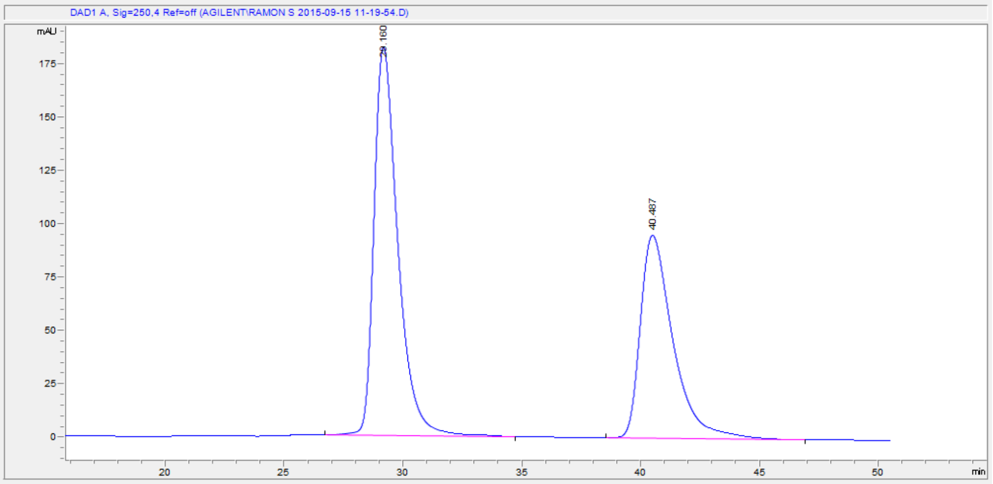

Peak #	RetTime [min]	Type	Width [min]	Area [mAU*s]	Height [mAU]	Area %
1	29.160	BB	1.0484	1.27333e4	182.41916	58.2436
2	40.487	BB	1.4318	9128.81836	95.27521	41.7564

Chiral S:

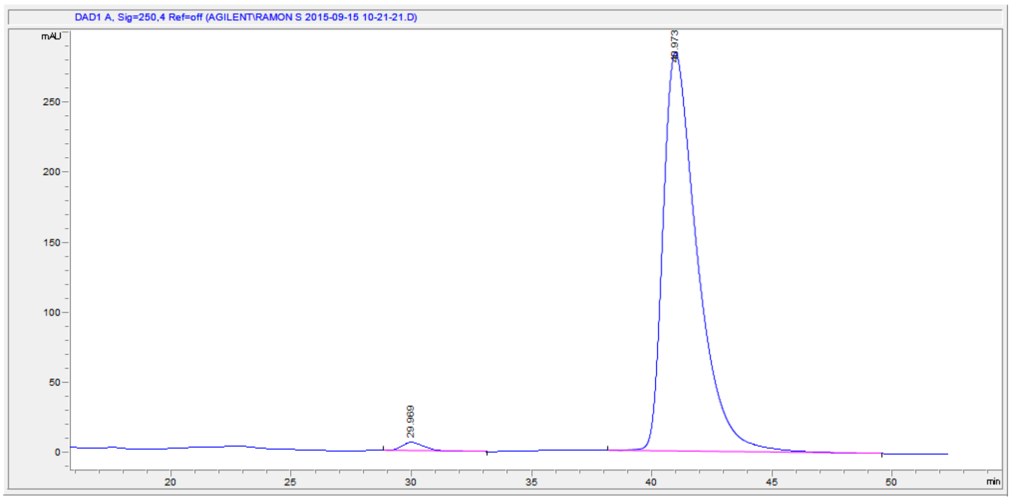

Peak #	RetTime [min]	Type	Width [min]	Area [mAU*s]	Height [mAU]	Area %
1	29.969	BB	0.8179	392.81580	6.01512	1.3698
2	40.973	BB	1.4759	2.82844e4	285.48447	98.6302

Chiral R:

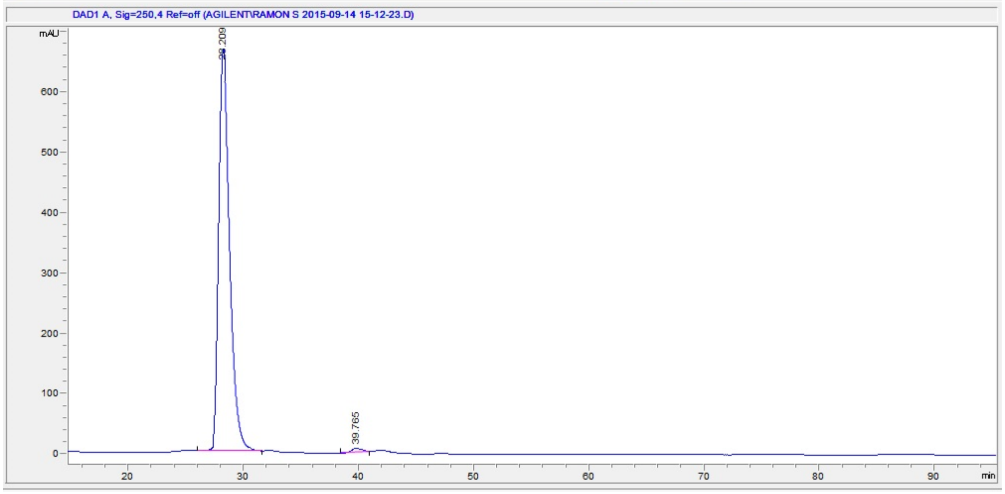

Peak #	RetTime [min]	Type	Width [min]	Area [mAU*s]	Height [mAU]	Area %
1	28.209	BB	1.0039	4.38354e4	665.96399	99.0009
2	39.765	BB	0.8426	442.39969	6.32062	0.9991

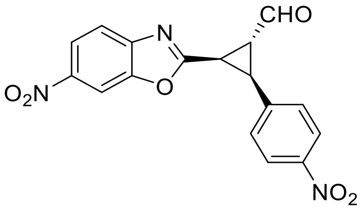

4d major

Mixture of S and R: (OD-H, 55.45, 210 nm, 0.8 ml/min)

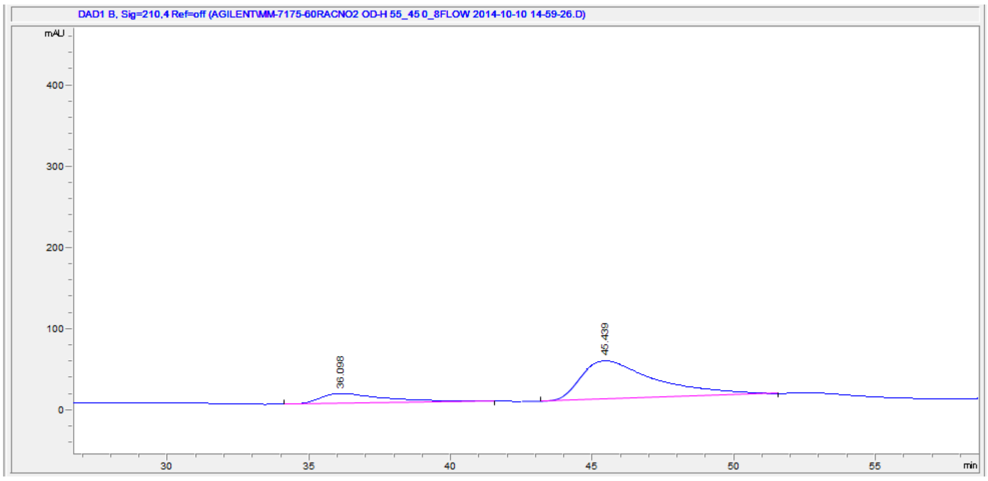

Peak #	RetTime [min]	Type	Width [min]	Area [mAU*s]	Height [mAU]	Area %
1	36.098	BB	1.9035	2012.17493	12.43705	18.9008
2	45.439	BB	2.1550	8633.78027	47.31137	81.0992

Chiral S:

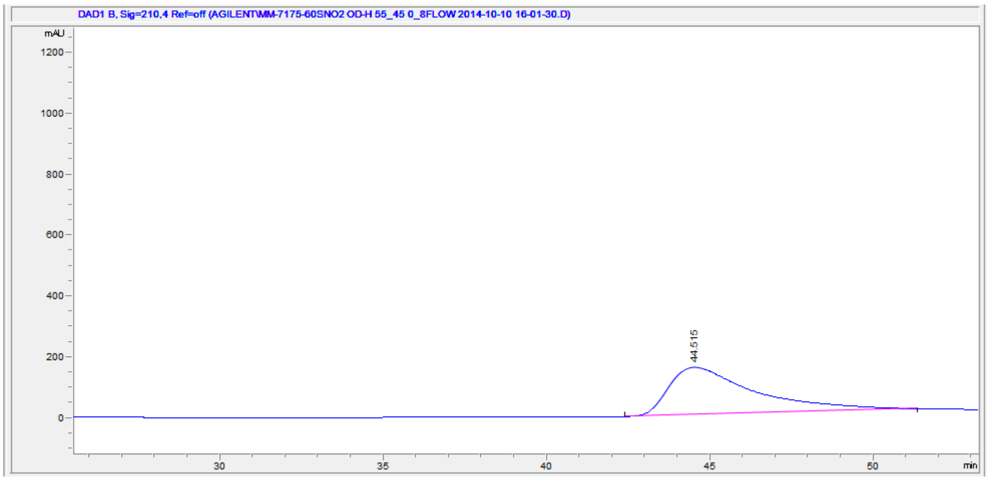

Peak #	RetTime [min]	Type	Width [min]	Area [mAU*s]	Height [mAU]	Area %
1	44.515	BB	2.5273	2.83576e4	155.96089	100.0000

Chiral R:

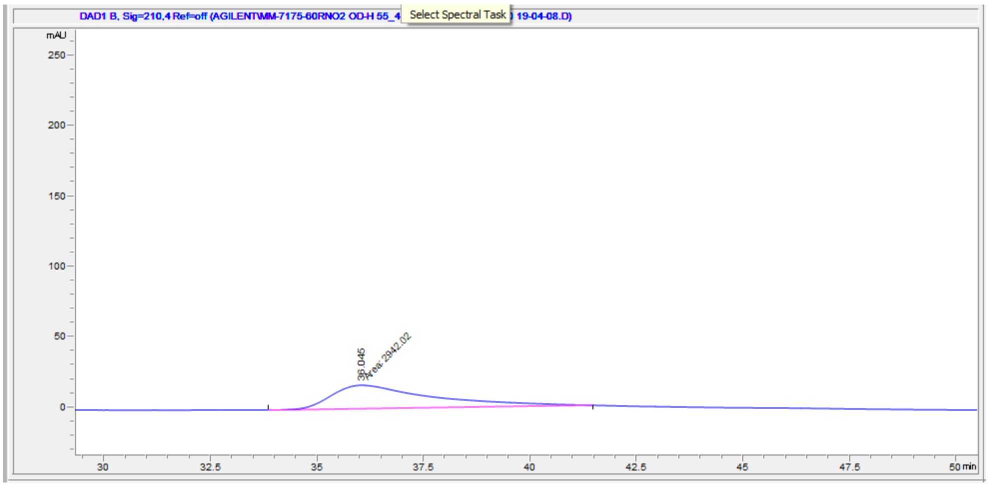

Peak #	RetTime [min]	Type	Width [min]	Area [mAU*s]	Height [mAU]	Area %
1	36.045	MM	2.8547	2942.01685	17.17661	100.0000

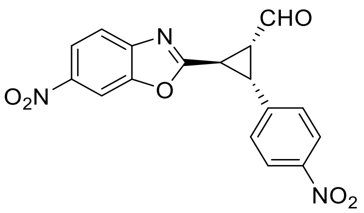

4d minor

Mixture of S and R: (IB, 70.30, 210 nm, 1 ml/min)

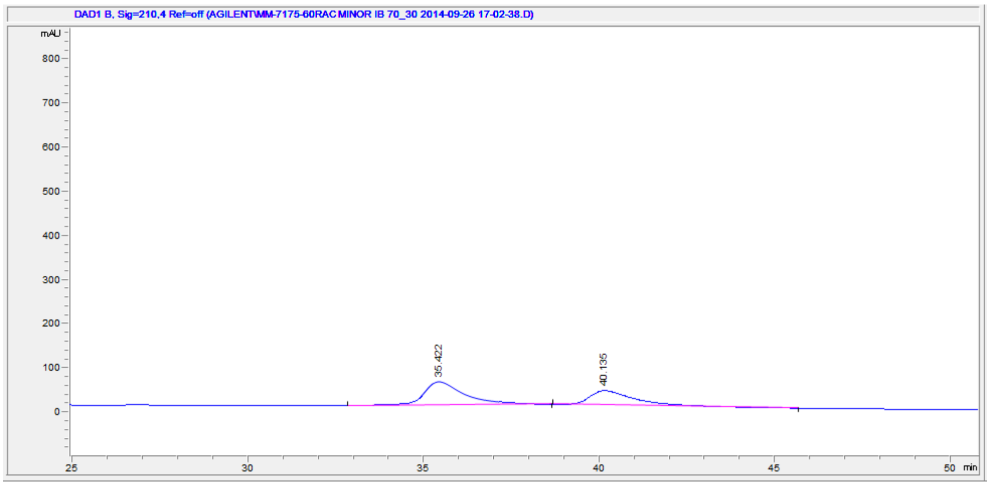

Peak #	RetTime [min]	Type	Width [min]	Area [mAU*s]	Height [mAU]	Area %
1	35.422	BB	1.1864	4404.99756	53.17717	59.1106
2	40.135	BB	1.2752	3047.12915	32.75777	40.8894

Chiral S:

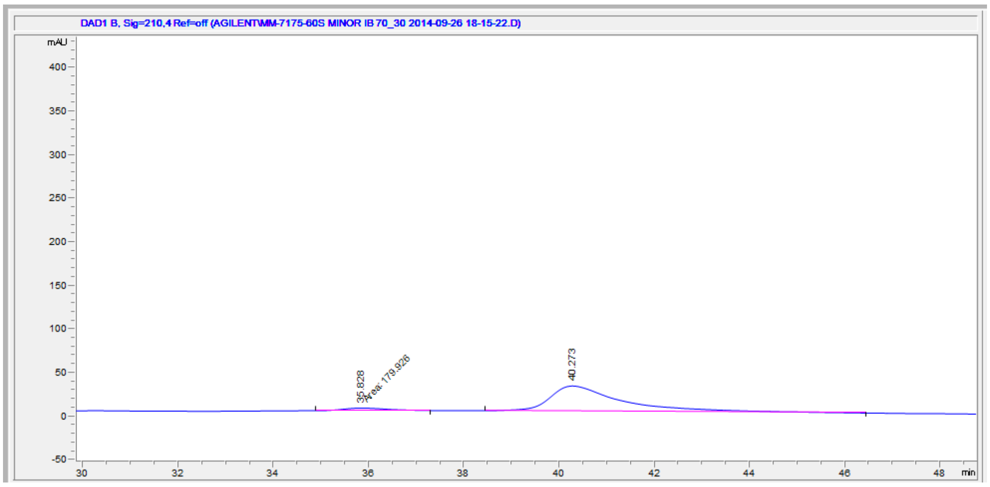

Peak #	RetTime [min]	Type	Width [min]	Area [mAU*s]	Height [mAU]	Area %
1	35.828	MM	1.0256	179.92630	2.92397	5.4440
2	40.273	BB	1.4220	3125.11719	28.93576	94.5560

Chiral R:

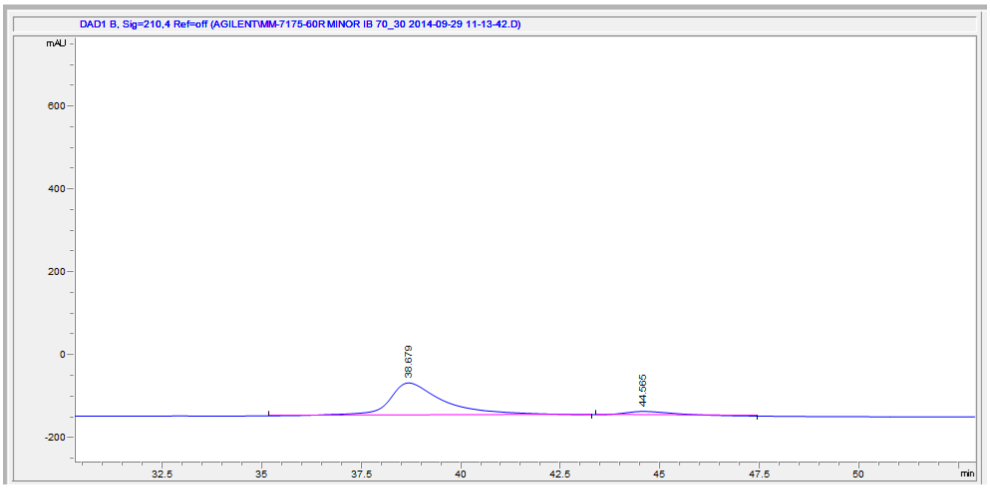

Peak #	RetTime [min]	Type	Width [min]	Area [mAU*s]	Height [mAU]	Area %
1	38.679	BB	1.3699	7624.45313	78.03210	90.6252
2	44.565	BB	1.1033	788.72156	8.85343	9.3748

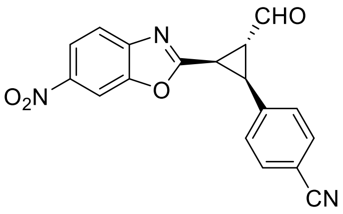

4f major

Mixture of S and R: (AY-H, 60.40, 210 nm, 0.8 ml/min)

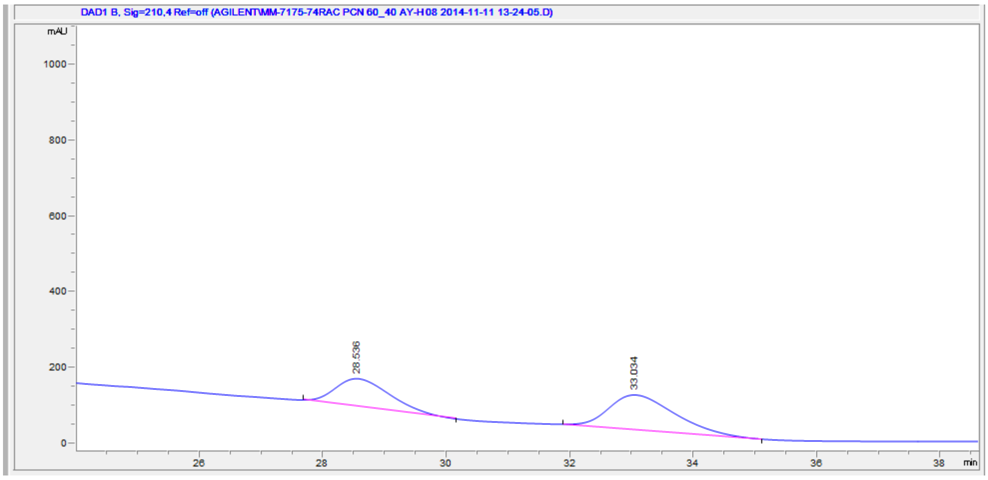

Peak #	RetTime [min]	Type	Width [min]	Area [mAU*s]	Height [mAU]	Area %
1	28.536	BB	0.9626	4597.28223	73.81712	40.1968
2	33.034	BB	1.1206	6839.66553	91.66003	59.8032

Chiral S:

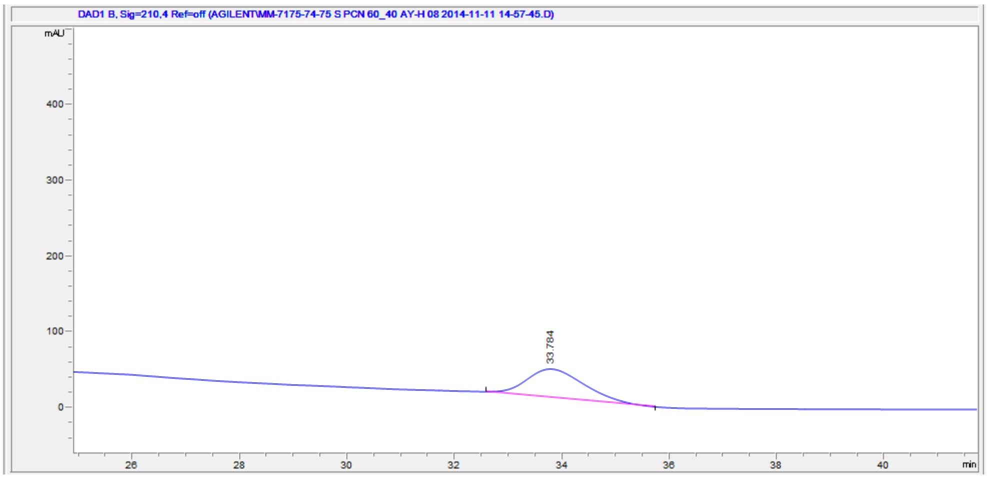

Peak #	RetTime [min]	Type	Width [min]	Area [mAU*s]	Height [mAU]	Area %
1	33.784	BB	1.1124	2820.95850	37.55339	100.0000

Chiral R:

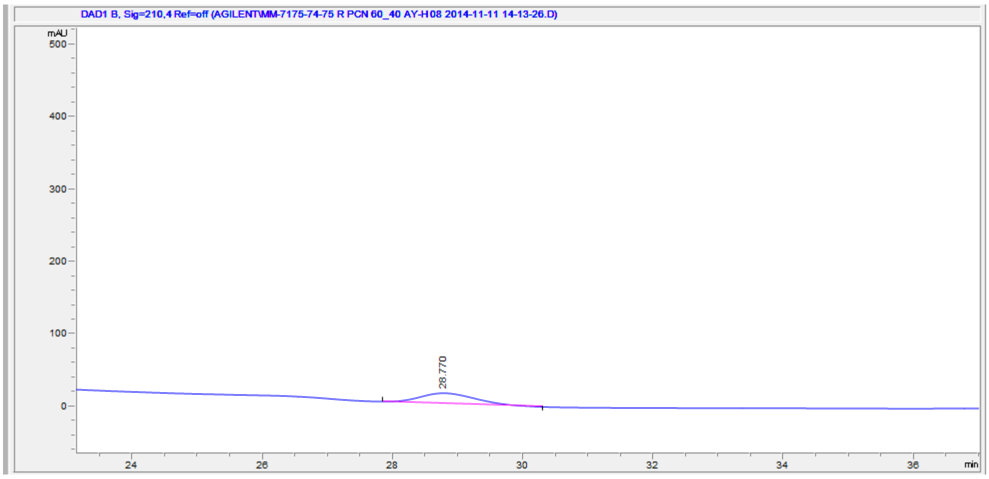

Peak #	RetTime [min]	Type	Width [min]	Area [mAU*s]	Height [mAU]	Area %
1	28.770	BB	0.8675	854.49066	14.33286	100.0000

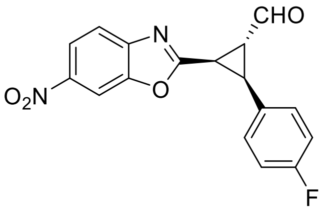

4e major

Mixture of S and R: (OD-H, 80.20, 210 nm, 1 ml/min)

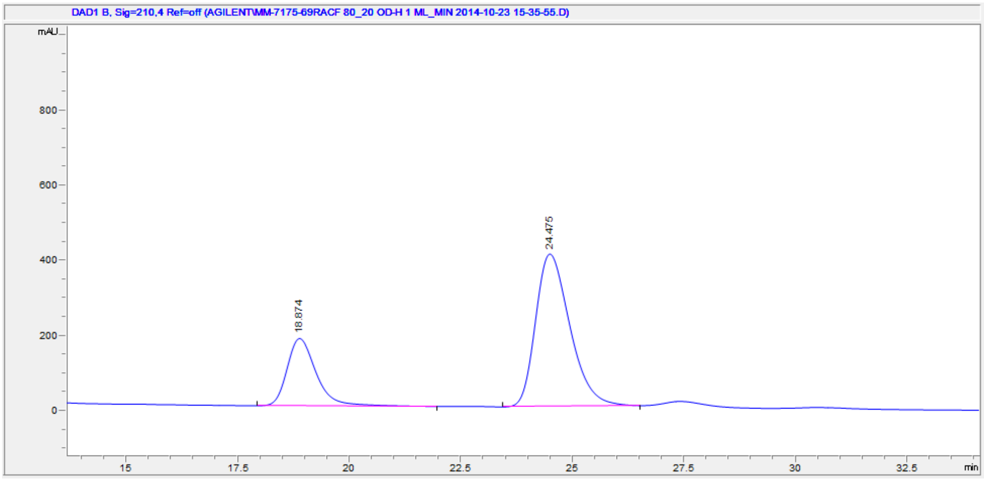

Peak #	RetTime [min]	Type	Width [min]	Area [mAU*s]	Height [mAU]	Area %
1	18.874	BB	0.6916	8130.86133	179.11133	27.3120
2	24.475	BB	0.8246	2.16394e4	405.50552	72.6880

Chiral S:

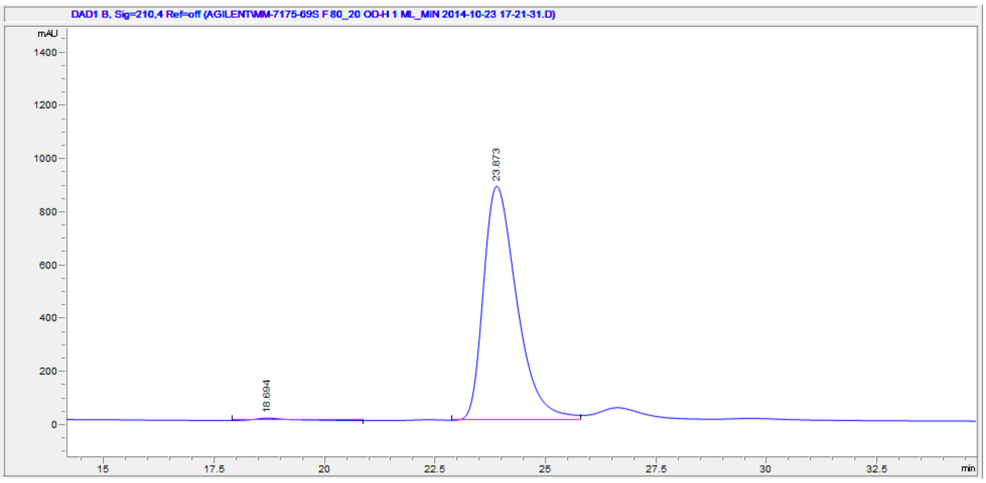

Peak #	RetTime [min]	Type	Width [min]	Area [mAU*s]	Height [mAU]	Area %
1	18.826	BB	0.6974	1.77314e4	386.41232	98.9789
2	24.756	MM	0.9648	182.91948	3.15982	1.0211

Chiral R:

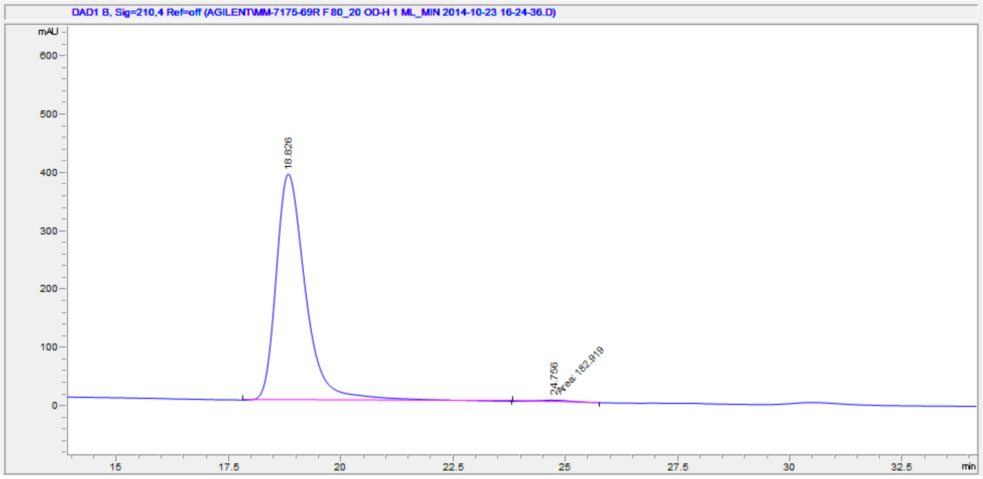

Peak #	RetTime [min]	Type	Width [min]	Area [mAU*s]	Height [mAU]	Area %
1	18.694	BB	0.7854	476.68951	8.53718	1.0226
2	23.873	BV	0.8031	4.61405e4	878.01160	98.9774

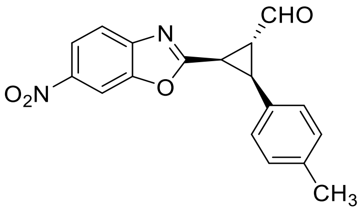

4g major

Mixture of S and R: (AY-H, 80.20, 210 nm, 1 ml/min)

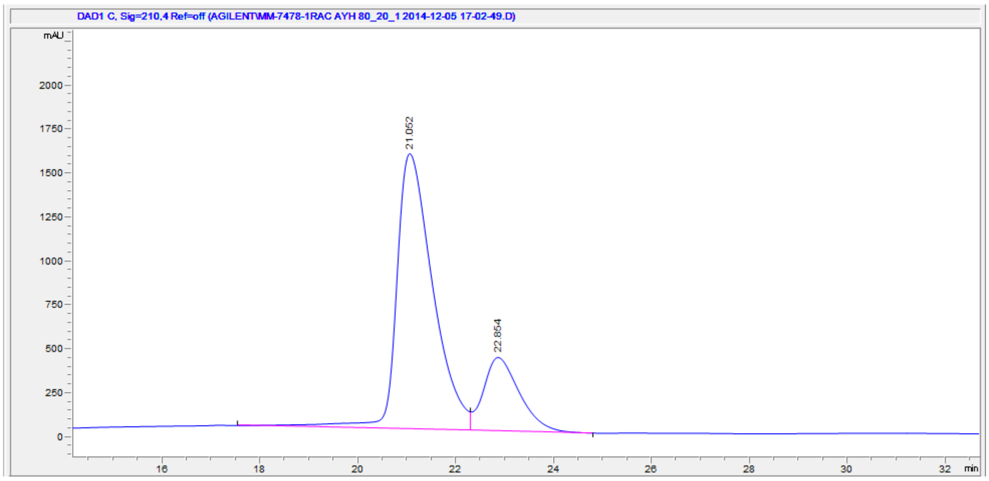

Peak #	RetTime [min]	Type	Width [min]	Area [mAU*s]	Height [mAU]	Area %
1	21.052	BV	0.7827	8.09678e4	1567.39832	79.1073
2	22.854	VB	0.7699	2.13840e4	418.67255	20.8927

Chiral S:

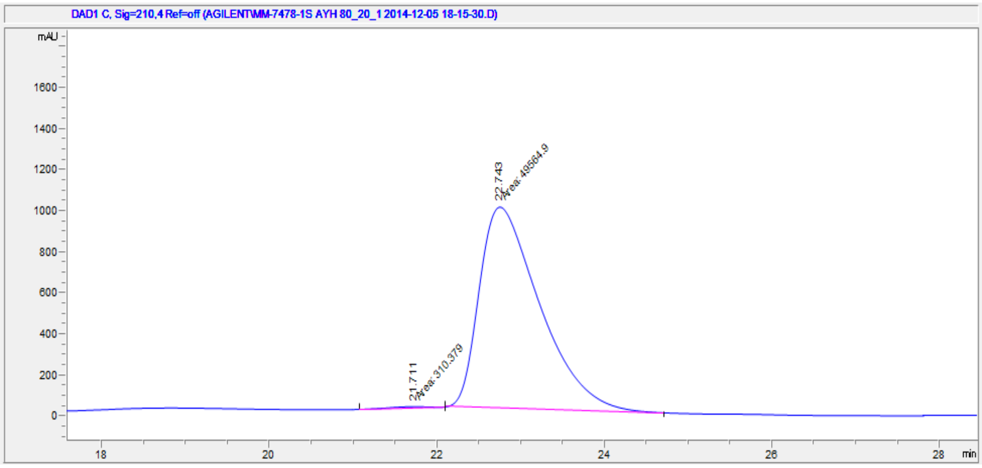

Peak #	RetTime [min]	Type	Width [min]	Area [mAU*s]	Height [mAU]	Area %
1	21.711	MM	0.6588	310.37949	7.85263	0.6223
2	22.743	MM	0.8401	4.95649e4	983.27905	99.3777

Chiral R:

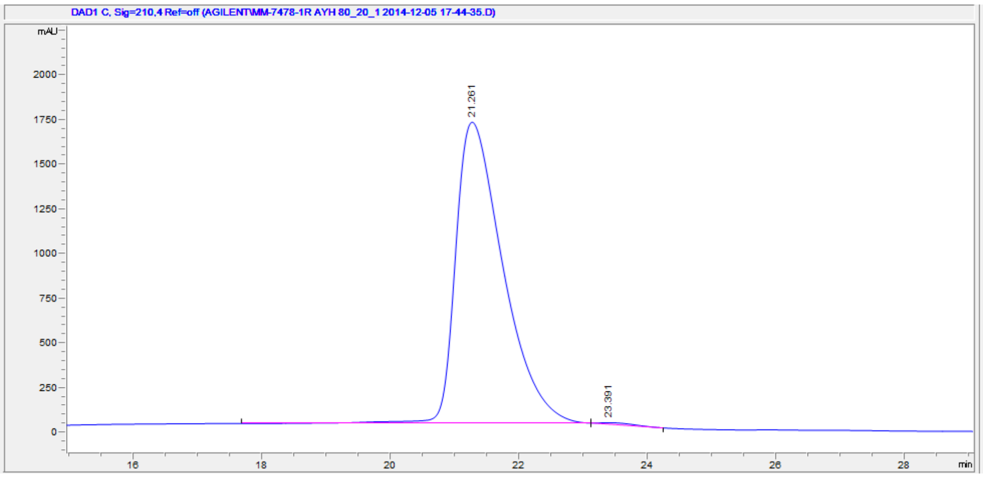

Peak #	RetTime [min]	Type	Width [min]	Area [mAU*s]	Height [mAU]	Area %
1	21.261	BB	0.7802	8.60503e4	1683.88342	99.5977
2	23.391	BB	0.6158	347.60397	8.45234	0.4023

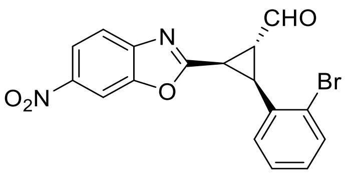

4h major

Mixture of S and R: (OD-H, 70.30, 210 nm, 0.8 ml/min)

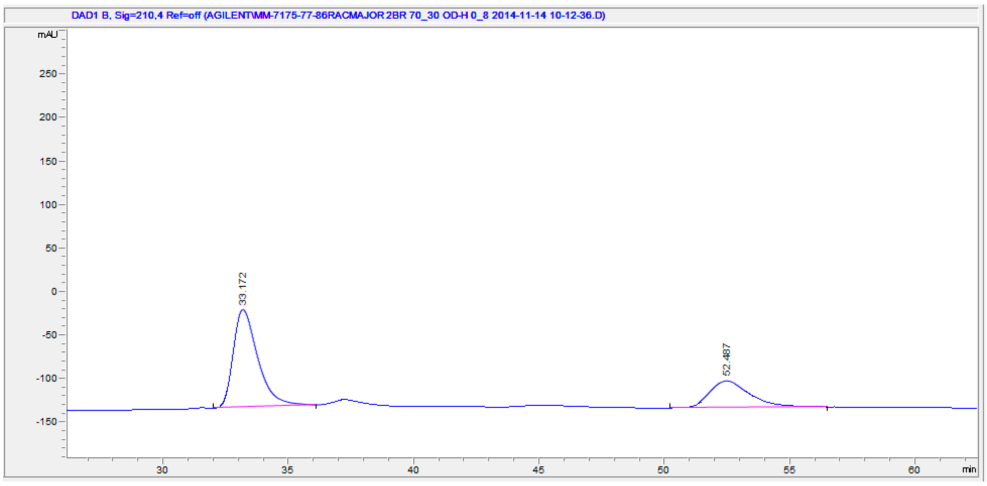

Peak #	RetTime [min]	Type	Width [min]	Area [mAU*s]	Height [mAU]	Area %
1	33.172	BB	1.0412	7677.73486	112.07510	69.1795
2	52.487	BB	1.4034	3420.53955	30.66447	30.8205

Chiral S:

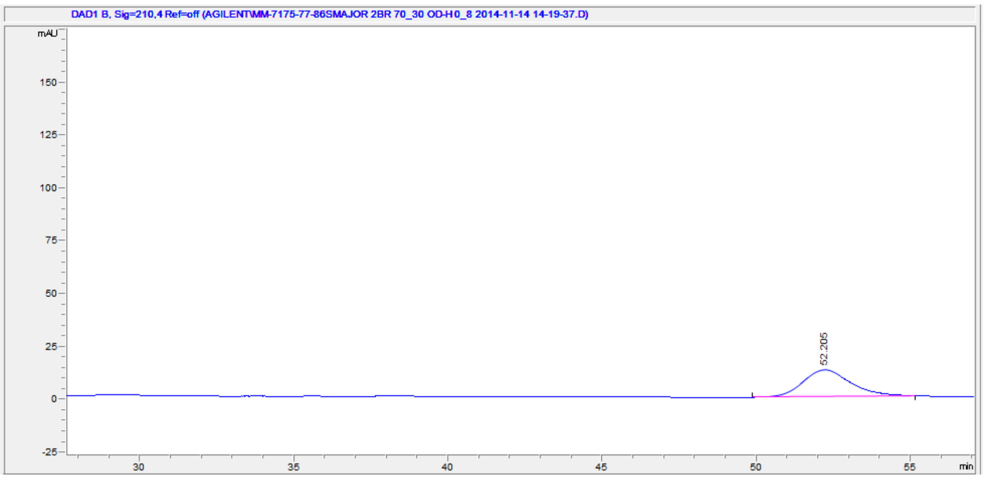

Peak #	RetTime [min]	Type	Width [min]	Area [mAU*s]	Height [mAU]	Area %
1	52.205	BB	1.2947	1362.70691	12.59043	100.0000

Chiral R:

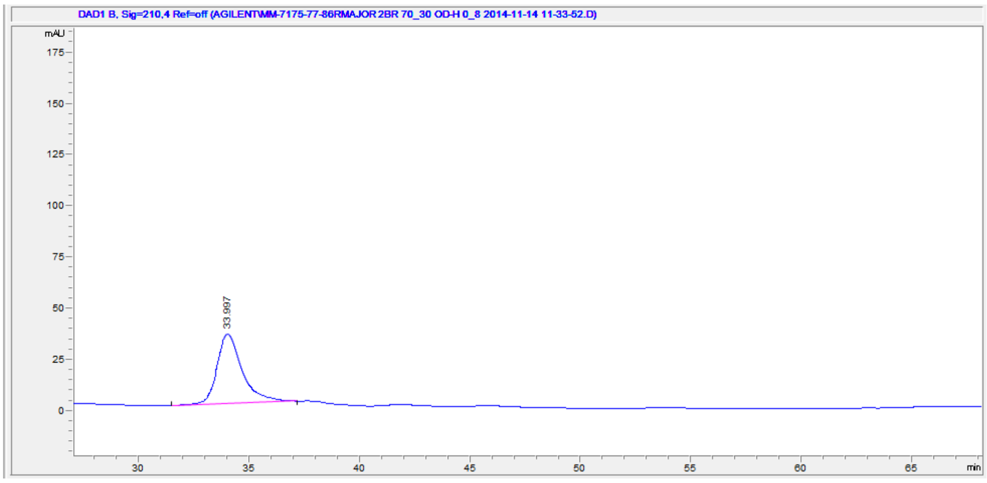

Peak #	RetTime [min]	Type	Width [min]	Area [mAU*s]	Height [mAU]	Area %
1	33.997	BB	1.1382	2712.94531	34.09796	100.0000

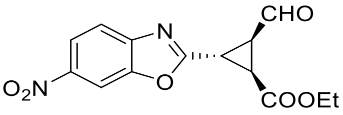

4i major

Mixture of S and R: (AY-H, 50.50, 210 nm, 1 ml/min)

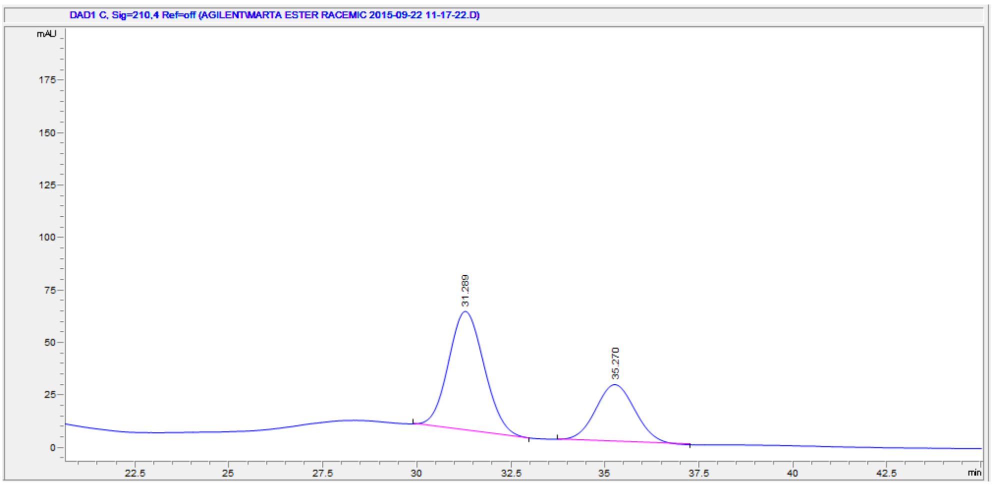

Peak #	RetTime [min]	Type	Width [min]	Area [mAU*s]	Height [mAU]	Area %
1	31.289	BB	1.0116	3765.18457	56.78594	65.3319
2	35.270	BB	1.0351	1997.97766	27.26040	34.6681

Chiral S:

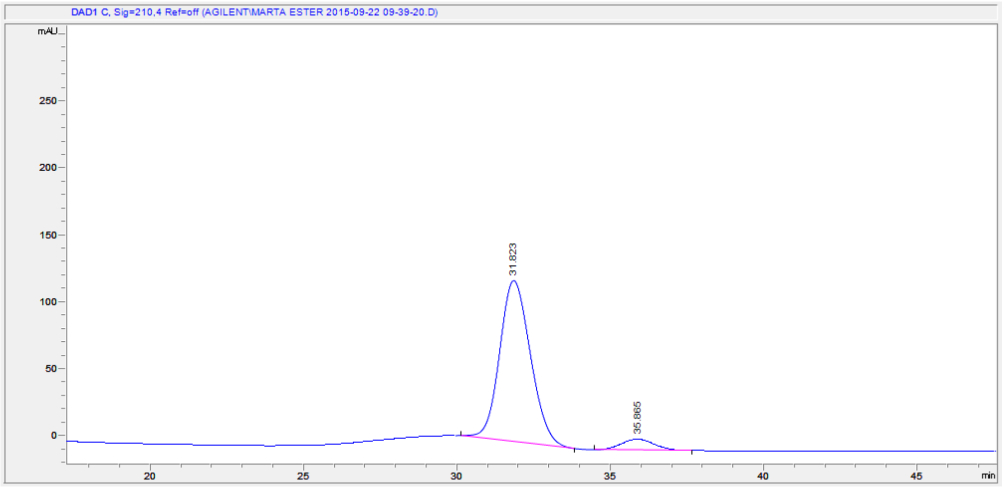

Peak #	RetTime [min]	Type	Width [min]	Area [mAU*s]	Height [mAU]	Area %
1	31.823	BB	1.0799	8389.00195	120.24745	93.3400
2	35.865	BB	0.8745	598.56952	8.11550	6.6600

Chiral R:

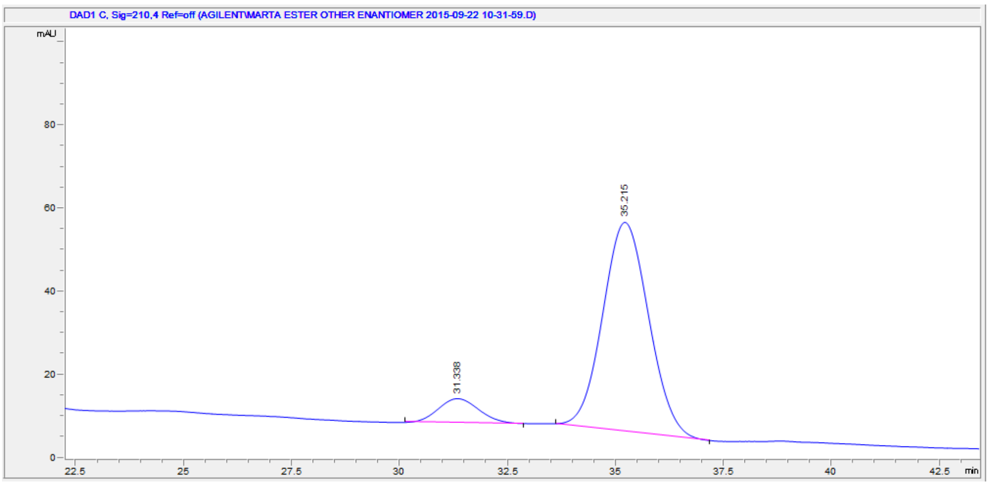

Peak #	RetTime [min]	Type	Width [min]	Area [mAU*s]	Height [mAU]	Area %
1	31.338	BB	0.7819	385.97067	5.87918	9.3632
2	35.215	BB	1.1297	3736.24438	50.46899	90.6368

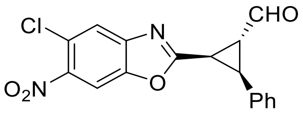

5a major

Mixture of S and R: (IC, 85.15, 210 nm, 1 ml/min)

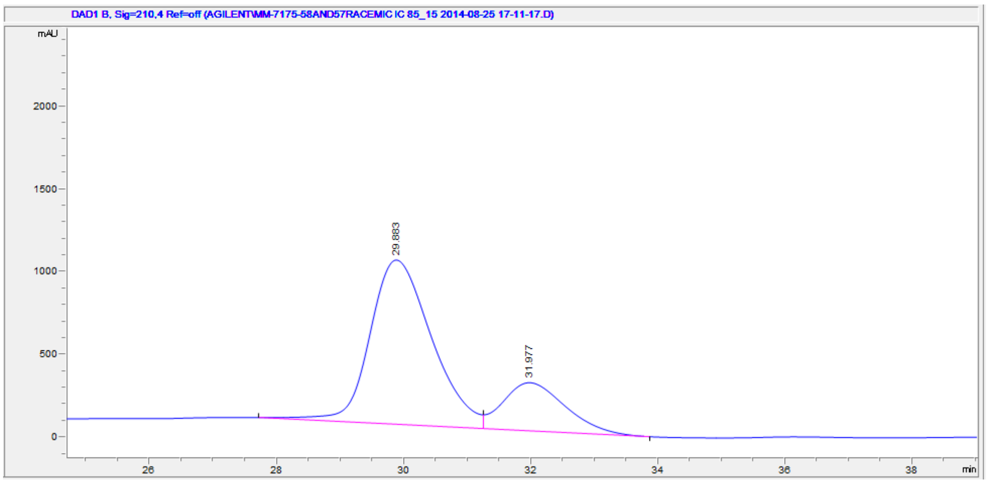

Peak #	RetTime [min]	Type	Width [min]	Area [mAU*s]	Height [mAU]	Area %
1	29.883	BV	1.0365	6.70024e4	991.41626	77.1635
2	31.977	VB	1.0480	1.98293e4	290.62515	22.8365

Chiral S:

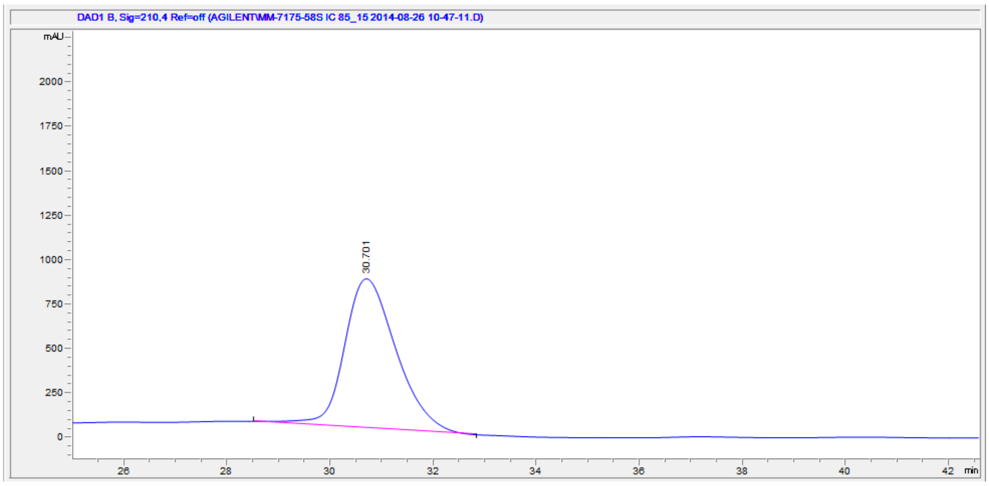

Peak #	RetTime [min]	Type	Width [min]	Area [mAU*s]	Height [mAU]	Area %
1	30.701	BB	1.0580	5.91403e4	843.49738	100.0000

Chiral R:

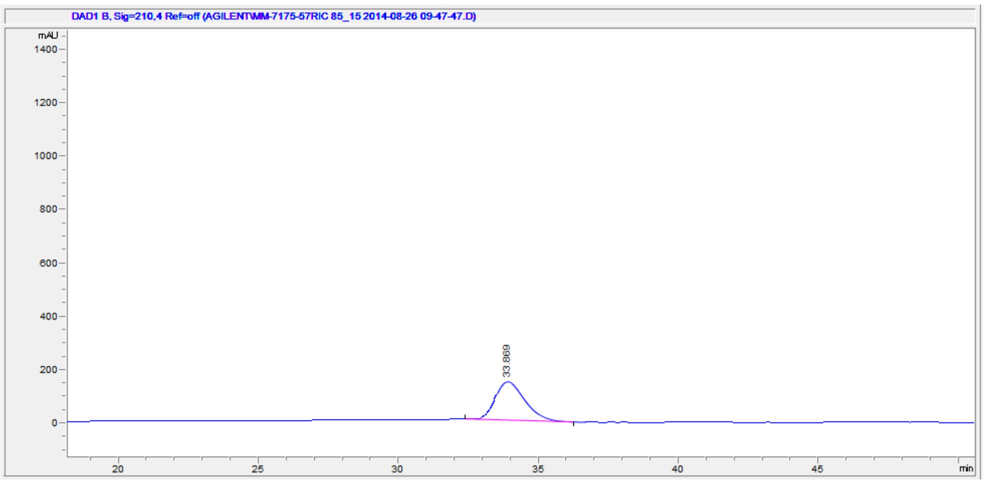

Peak #	RetTime [min]	Type	Width [min]	Area [mAU*s]	Height [mAU]	Area %
1	33.869	BB	1.1023	1.04667e4	143.95297	100.0000

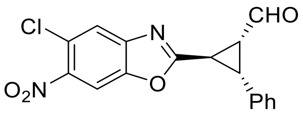

5a minor

Mixture of S and R: (IC, 85.15, 210 nm, 1 ml/min)

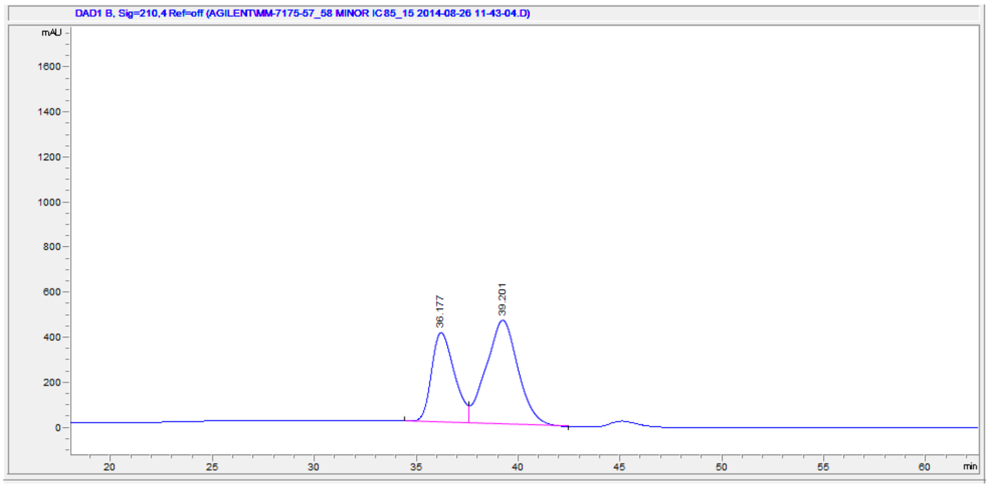

Peak #	RetTime [min]	Type	Width [min]	Area [mAU*s]	Height [mAU]	Area %
1	36.177	BV	1.1856	3.10209e4	396.98383	38.1902
2	39.201	VB	1.5418	5.02063e4	462.02722	61.8098

Chiral R:

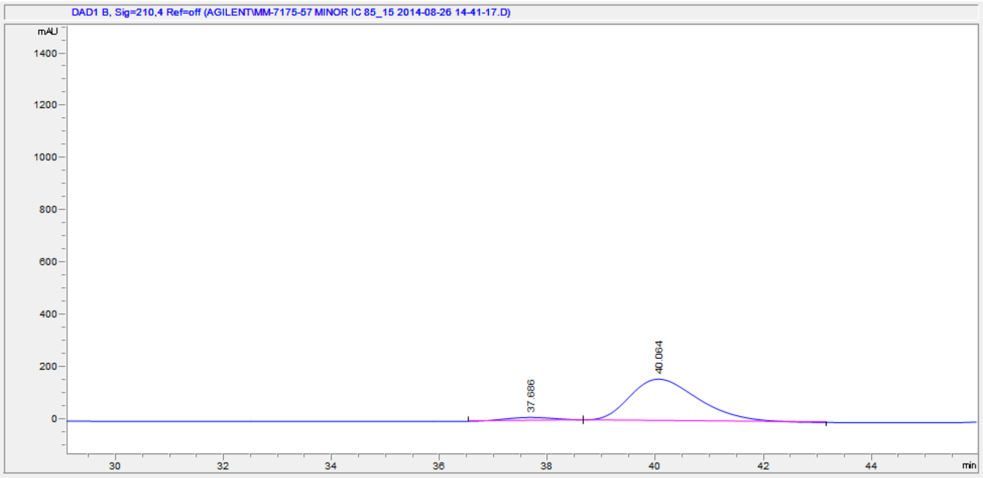

Peak #	RetTime [min]	Type	Width [min]	Area [mAU*s]	Height [mAU]	Area %
1	37.686	BB	0.8948	733.99097	11.75006	4.9646
2	40.064	BB	1.3182	1.40506e4	158.49655	95.0354

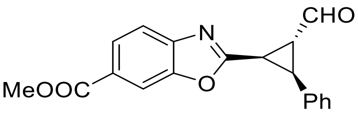

5c major

Mixture of S and R: (AY-H, 80.20, 230 nm, 1ml/min)

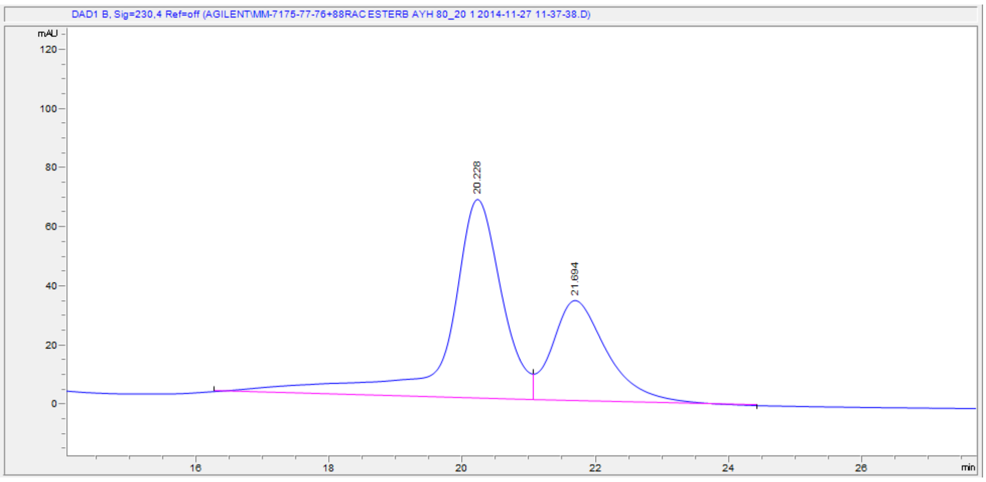

Peak #	RetTime [min]	Type	Width [min]	Area [mAU*s]	Height [mAU]	Area %
1	20.228	BV	0.8164	3785.40845	67.48930	65.8862
2	21.694	VB	0.8453	1959.96216	34.06174	34.1138

Chiral S:

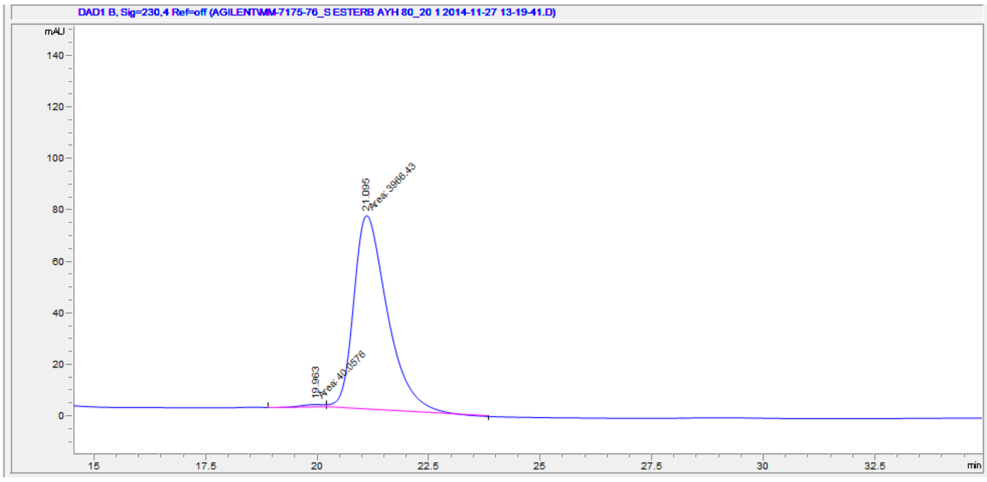

Peak #	RetTime [min]	Type	Width [min]	Area [mAU*s]	Height [mAU]	Area %
1	19.963	MM	0.6765	40.05763	9.86947e-1	0.9998
2	21.095	MM	0.8796	3966.42554	75.15952	99.0002

Chiral R:

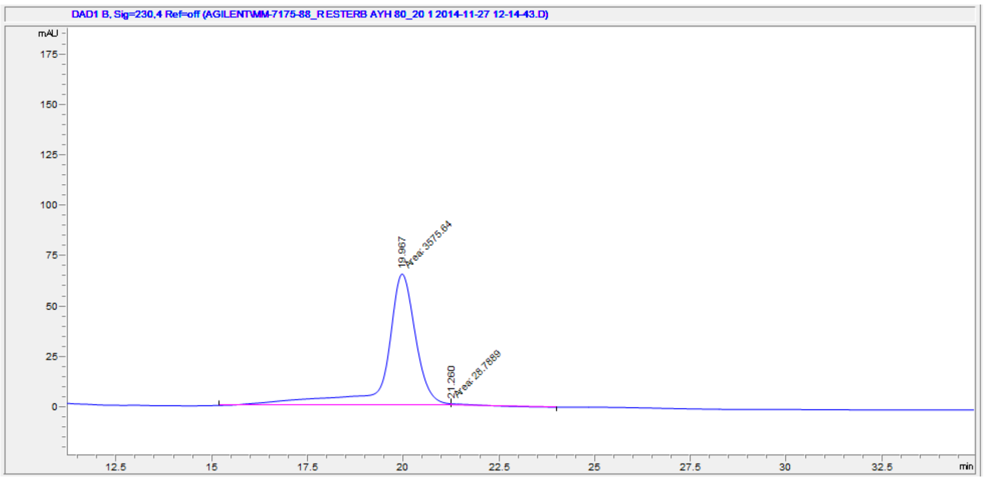

Peak #	RetTime [min]	Type	Width [min]	Area [mAU*s]	Height [mAU]	Area %
1	19.967	MM	0.9136	3575.64111	65.23161	99.2013
2	21.260	MM	0.5517	28.78887	6.16726e-1	0.7987

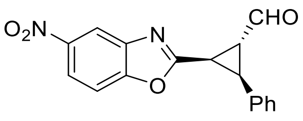

5b major

Mixture of S and R: (OD-H, 70.30, 210 nm, 1 ml/min)

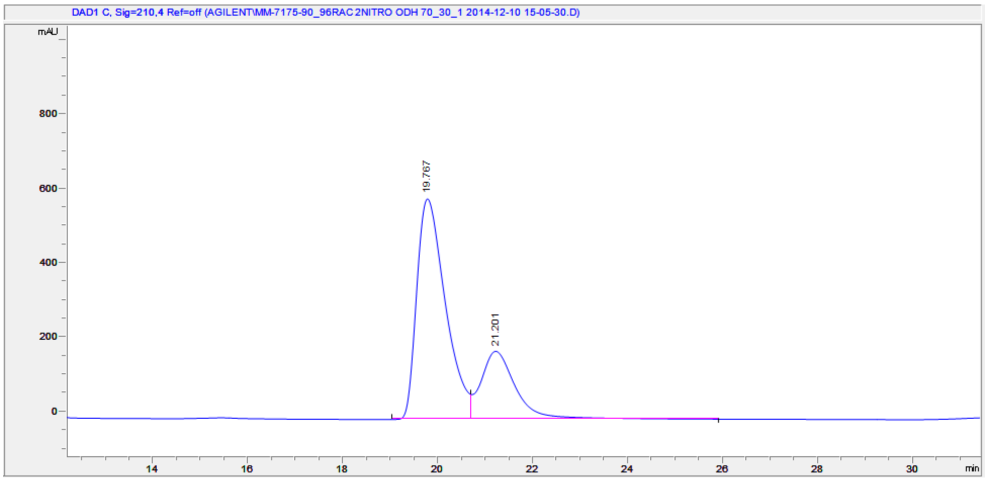

Peak #	RetTime [min]	Type	Width [min]	Area [mAU*s]	Height [mAU]	Area %
1	19.767	BV	0.6455	2.49488e4	594.60162	72.7906
2	21.201	VB	0.7510	9325.92969	183.46526	27.2094

Chiral S:

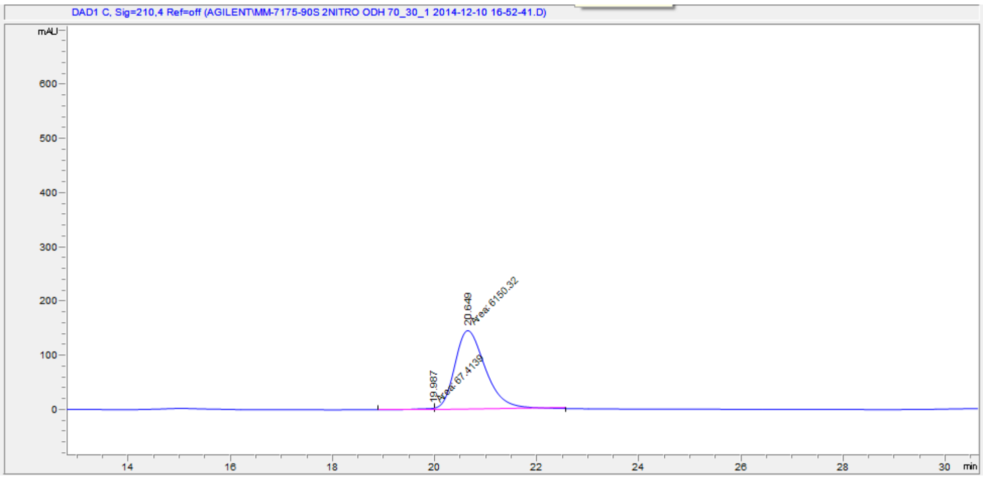

Peak #	RetTime [min]	Type	Width [min]	Area [mAU*s]	Height [mAU]	Area %
1	19.987	MM	0.3926	67.41388	2.86209	1.0842
2	20.649	MM	0.7032	6150.31982	145.77878	98.9158

Chiral R:

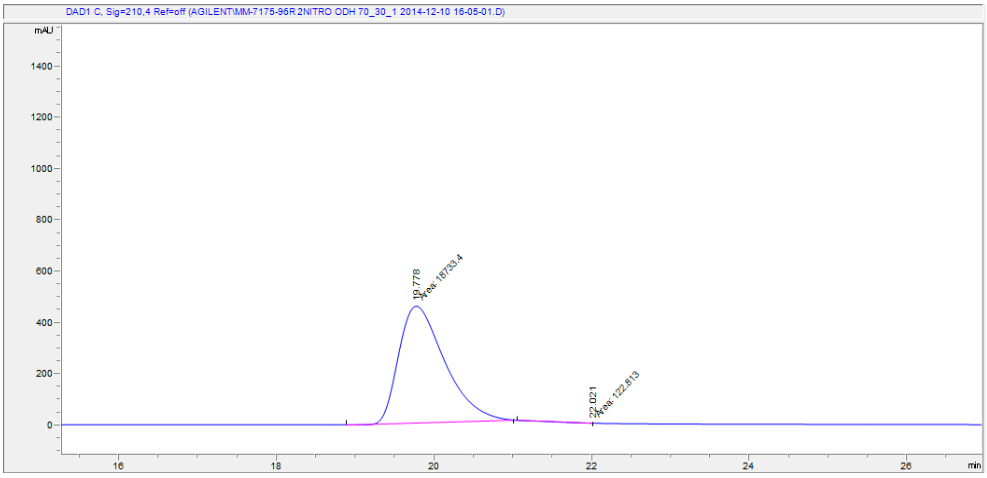

Peak #	RetTime [min]	Type	Width [min]	Area [mAU*s]	Height [mAU]	Area %
1	19.778	MM	0.6811	1.87334e4	458.38950	99.3487
2	22.021	MM	0.5521	122.81274	3.70713	0.6513

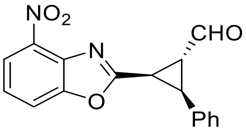

5d major

Mixture of S and R: (AY-H, 70.30, 210 nm, 1 ml/min)

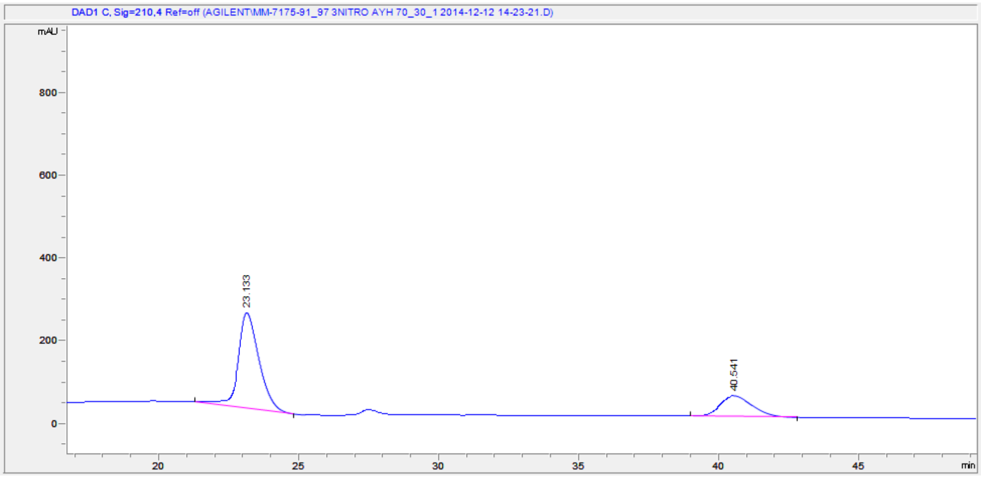

Peak #	RetTime [min]	Type	Width [min]	Area [mAU*s]	Height [mAU]	Area %
1	23.132	BB	0.7765	9084.07031	176.49504	74.9754
2	40.541	BB	1.1741	3031.99414	38.19610	25.0246

Chiral S:

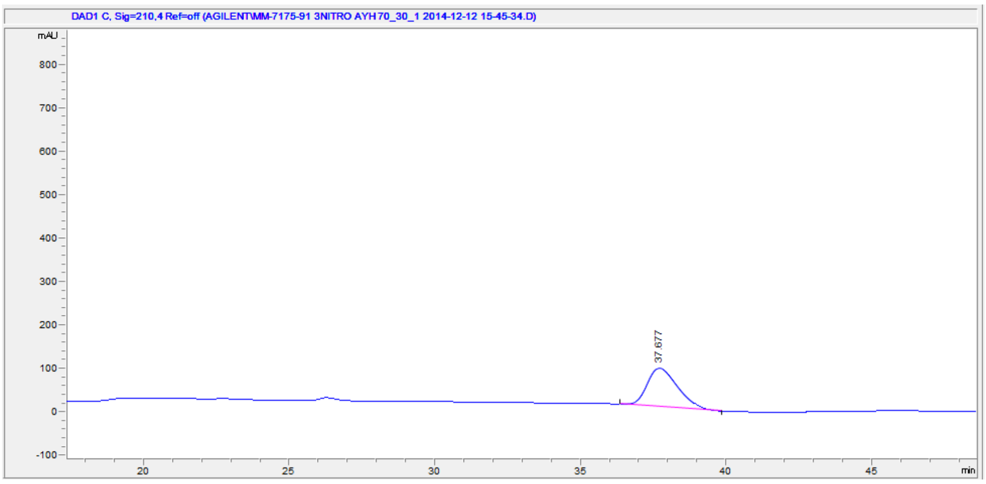

Peak #	RetTime [min]	Type	Width [min]	Area [mAU*s]	Height [mAU]	Area %
1	37.677	BB	1.1013	6581.40137	89.35355	100.0000

Chiral R:

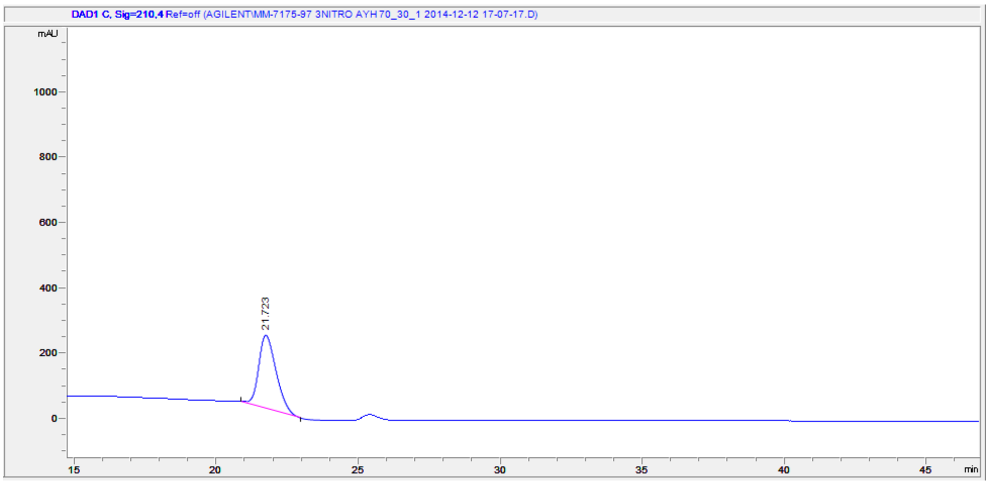

Peak #	RetTime [min]	Type	Width [min]	Area [mAU*s]	Height [mAU]	Area %
1	21.723	BB	0.6580	9639.62207	224.94237	100.0000

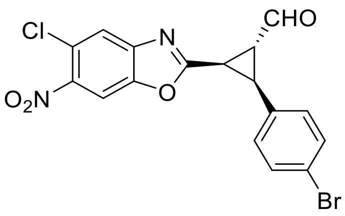

5e major

Mixture of S and R: (OD-H, 70.30, 230 nm, 1 ml/min)

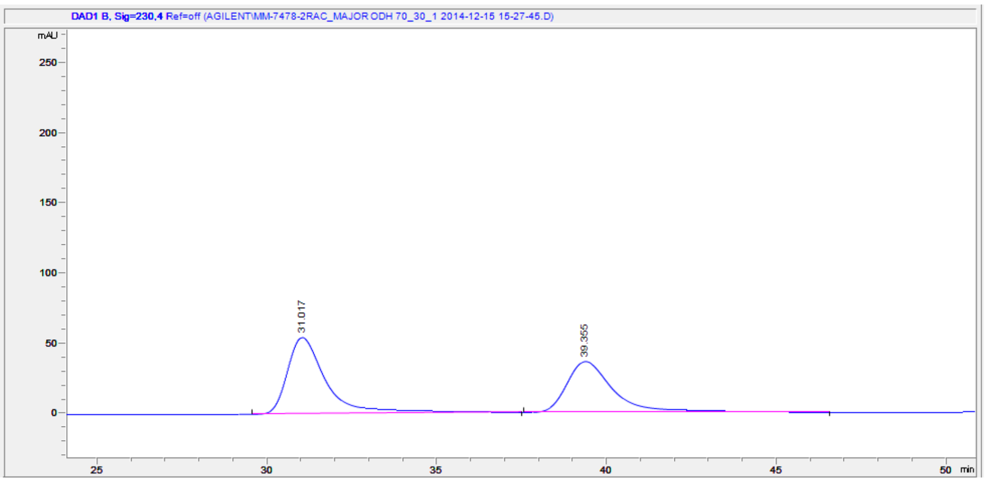

Peak #	RetTime [min]	Type	Width [min]	Area [mAU*s]	Height [mAU]	Area %
1	31.017	BB	1.1881	4379.56885	54.58246	55.2011
2	39.355	BB	1.3760	3554.28076	36.43872	44.7989

Chiral S:

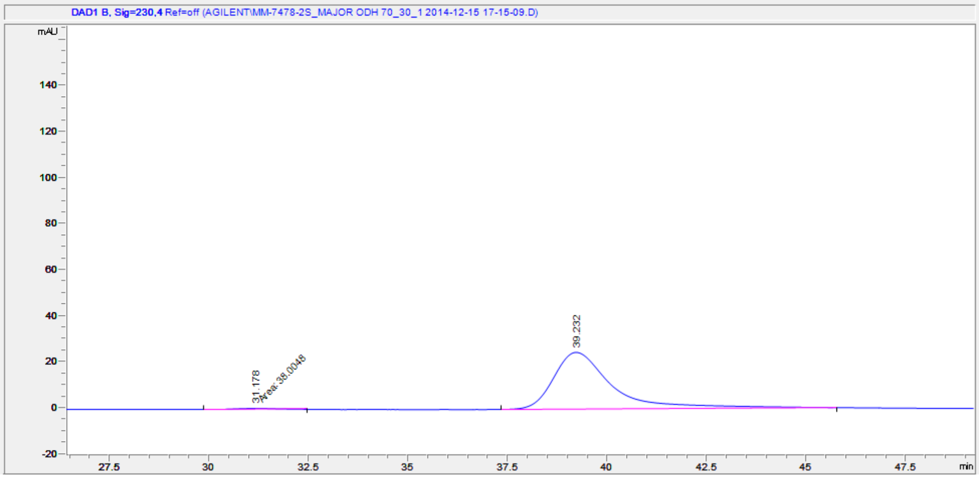

Peak #	RetTime [min]	Type	Width [min]	Area [mAU*s]	Height [mAU]	Area %
1	31.178	MM	1.2502	38.00478	5.06661e-1	1.4972
2	39.232	BB	1.4477	2500.42114	24.56225	98.5028

Chiral R:

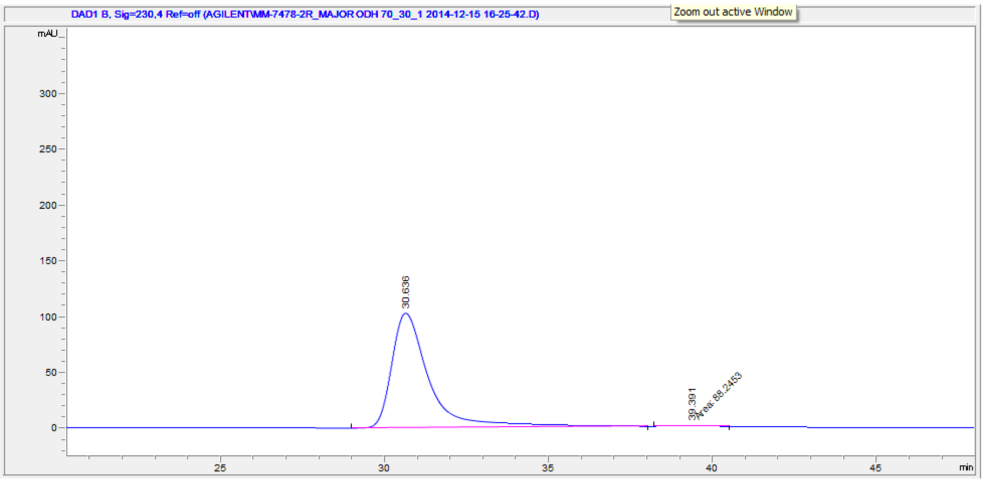

Peak #	RetTime [min]	Type	Width [min]	Area [mAU*s]	Height [mAU]	Area %
1	30.636	BB	1.1803	8144.36621	102.36057	98.9281
2	39.391	MM	1.3601	88.24529	1.08140	1.0719

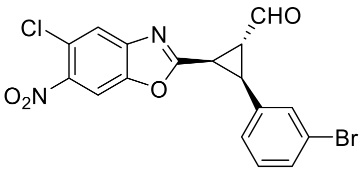

5f major

Mixture of S and R: (OD-H, 70.30, 210 nm, 1 ml/min)

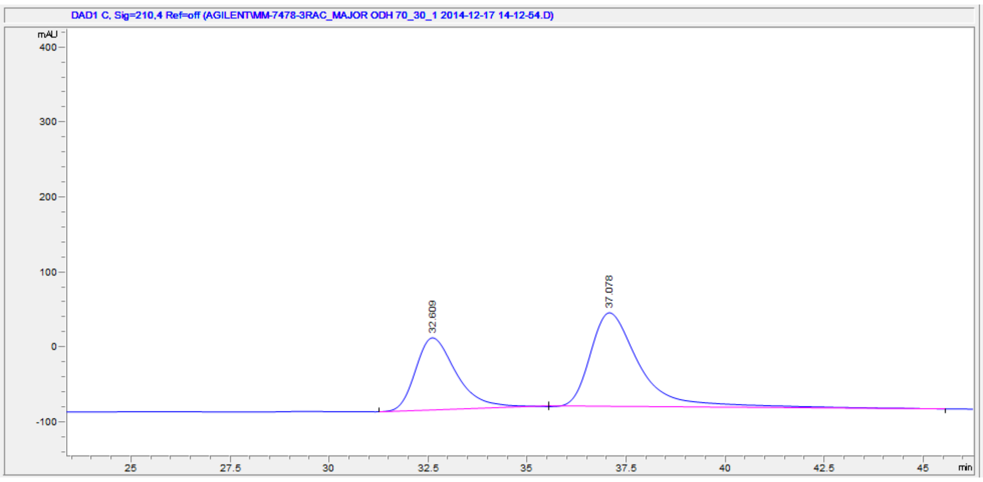

Peak #	RetTime [min]	Type	Width [min]	Area [mAU*s]	Height [mAU]	Area %
1	32.609	BB	1.0866	6947.40869	96.18931	38.4527
2	37.078	BB	1.3132	1.11200e4	125.31839	61.5473

Chiral S:

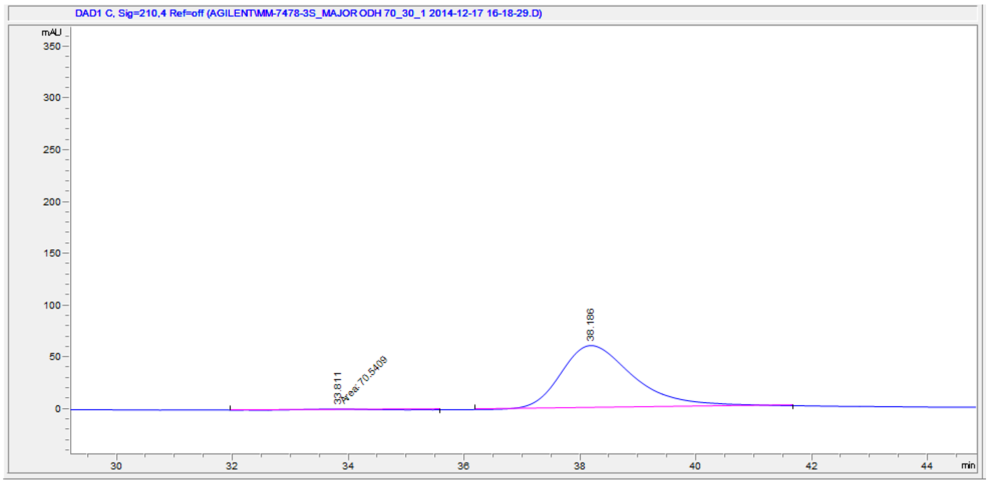

Peak #	RetTime [min]	Type	Width [min]	Area [mAU*s]	Height [mAU]	Area %
1	33.811	MM	1.4206	70.54089	8.27576e-1	1.3019
2	38.186	BB	1.2748	5347.89258	60.40890	98.6981

Chiral R:

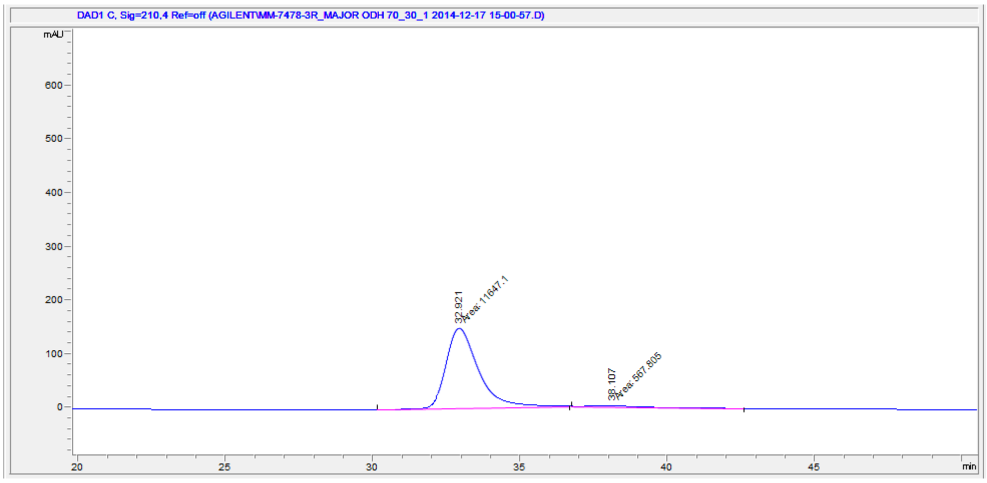

Peak #	RetTime [min]	Type	Width [min]	Area [mAU*s]	Height [mAU]	Area %
1	32.921	MM	1.2967	1.16471e4	149.70476	95.3515
2	38.107	MM	2.9513	567.80548	3.20654	4.6485

19. NMR starting materials

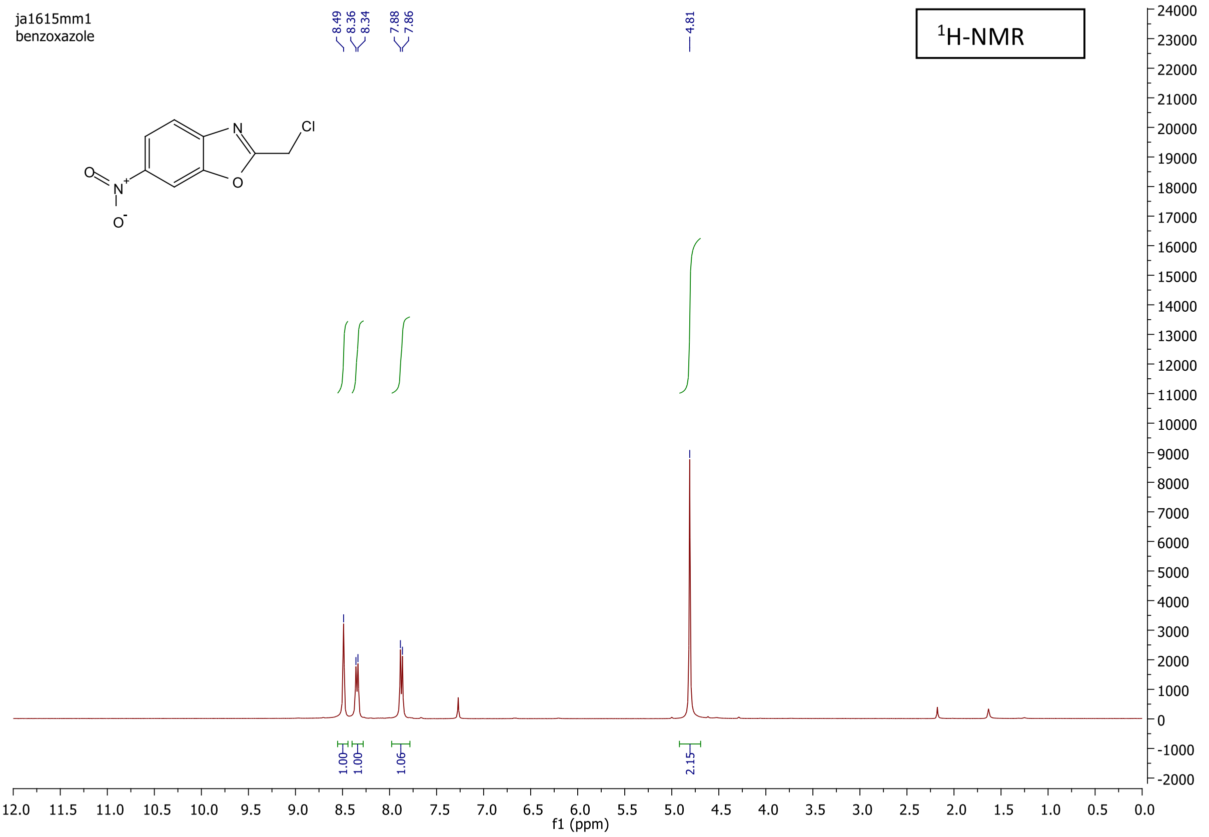

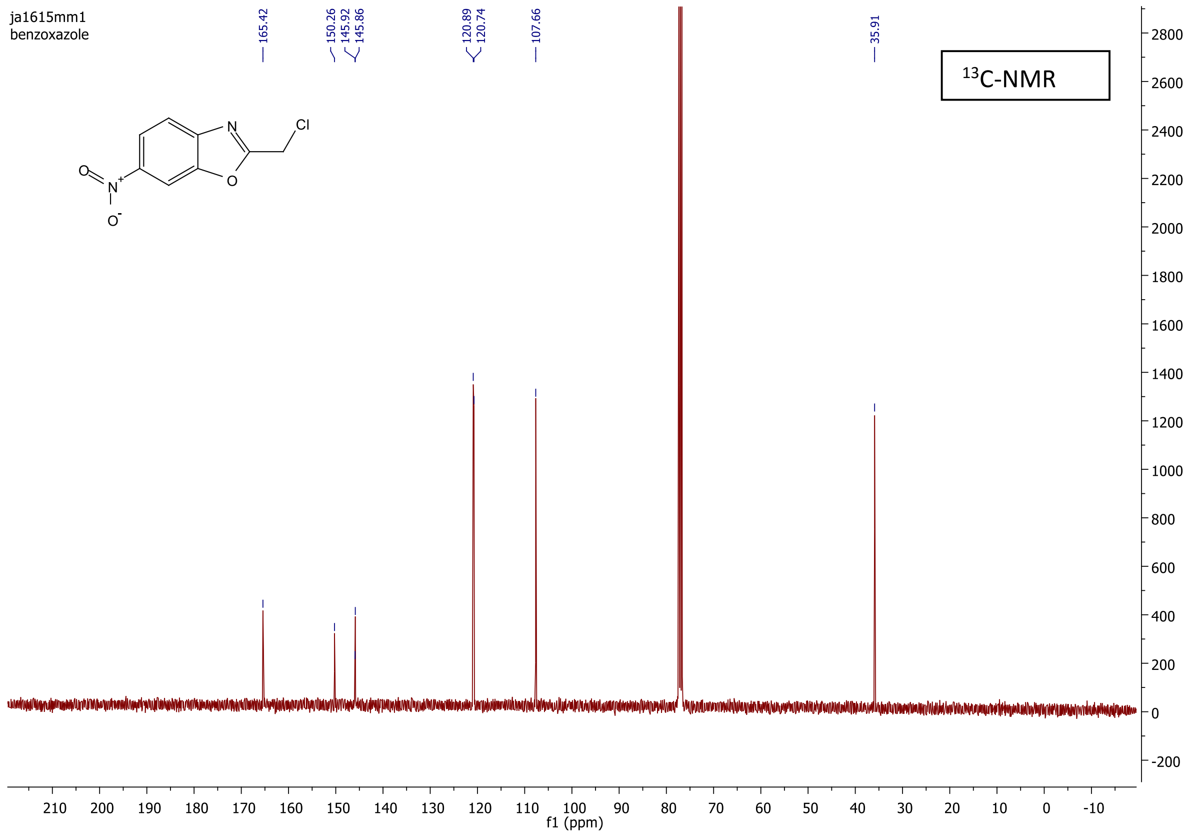

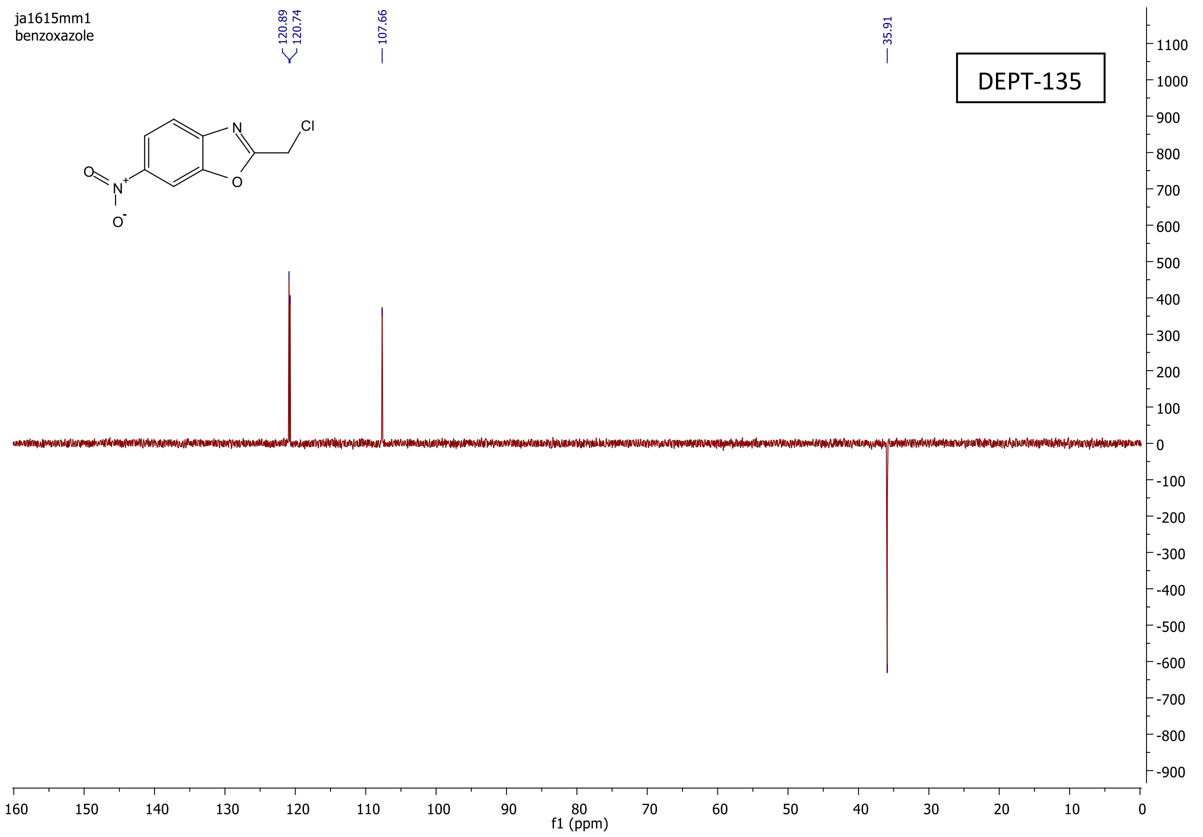
ja1615mm1
benzoxazole
120.89
120.74
107.66
35.91
DEPT-135
f1 (ppm)

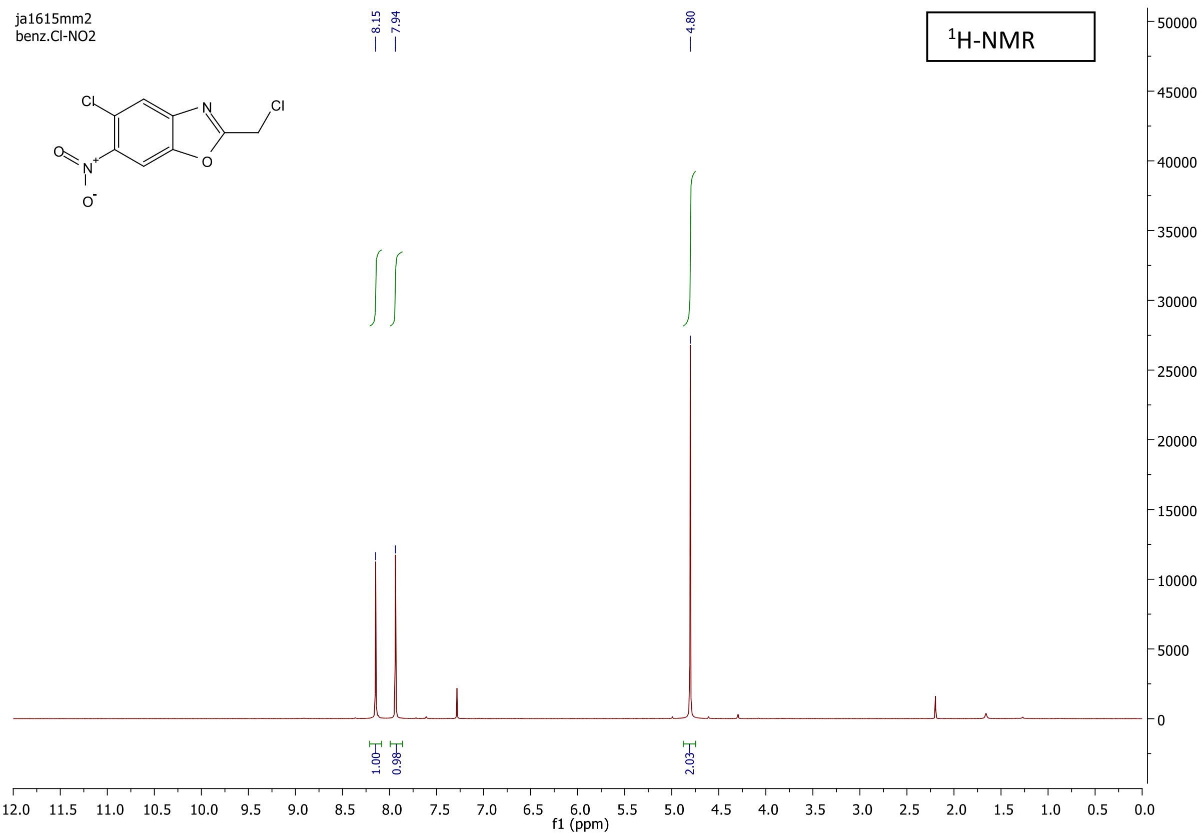
ja1615mm2
benz.Cl-NO2
8.15
7.94
4.80
1H-NMR
1.00
0.98
2.03
f1 (ppm)

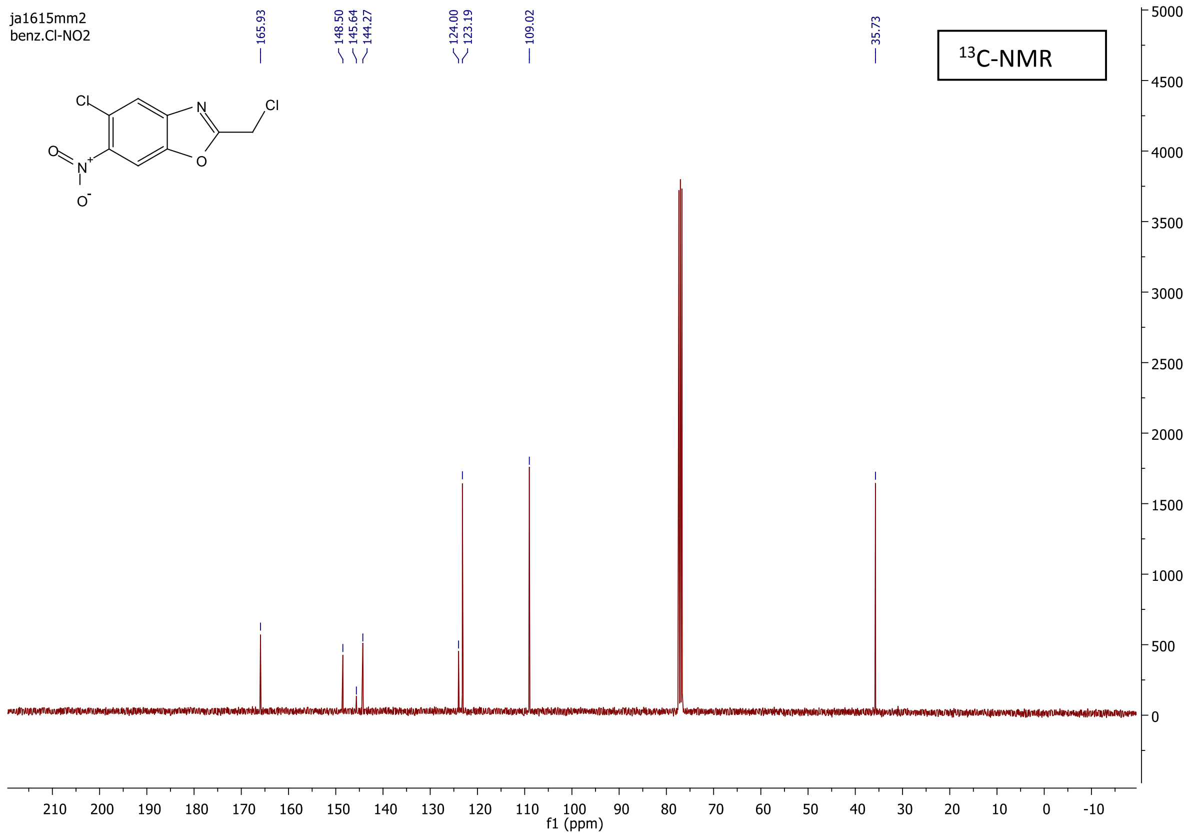

ja1615mm2
benz.Cl-NO2
165.93
148.50
145.64
144.27
124.00
123.19
109.02
35.73
13C-NMR
Cl
N
Cl
O
N+
O-
O
f1 (ppm)

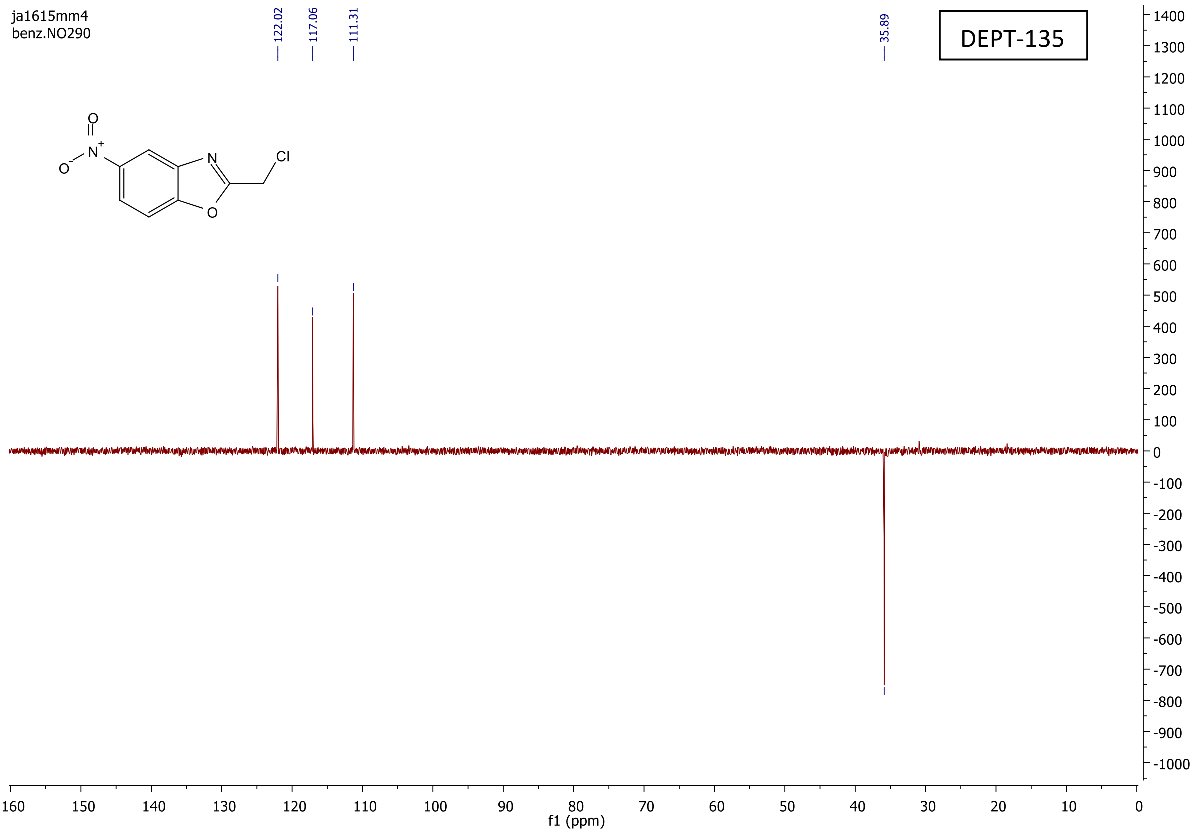

ja1615mm4
benz.NO290
122.02
117.06
111.31
35.89
DEPT-135
O
N+
O-
N
Cl
O
f1 (ppm)

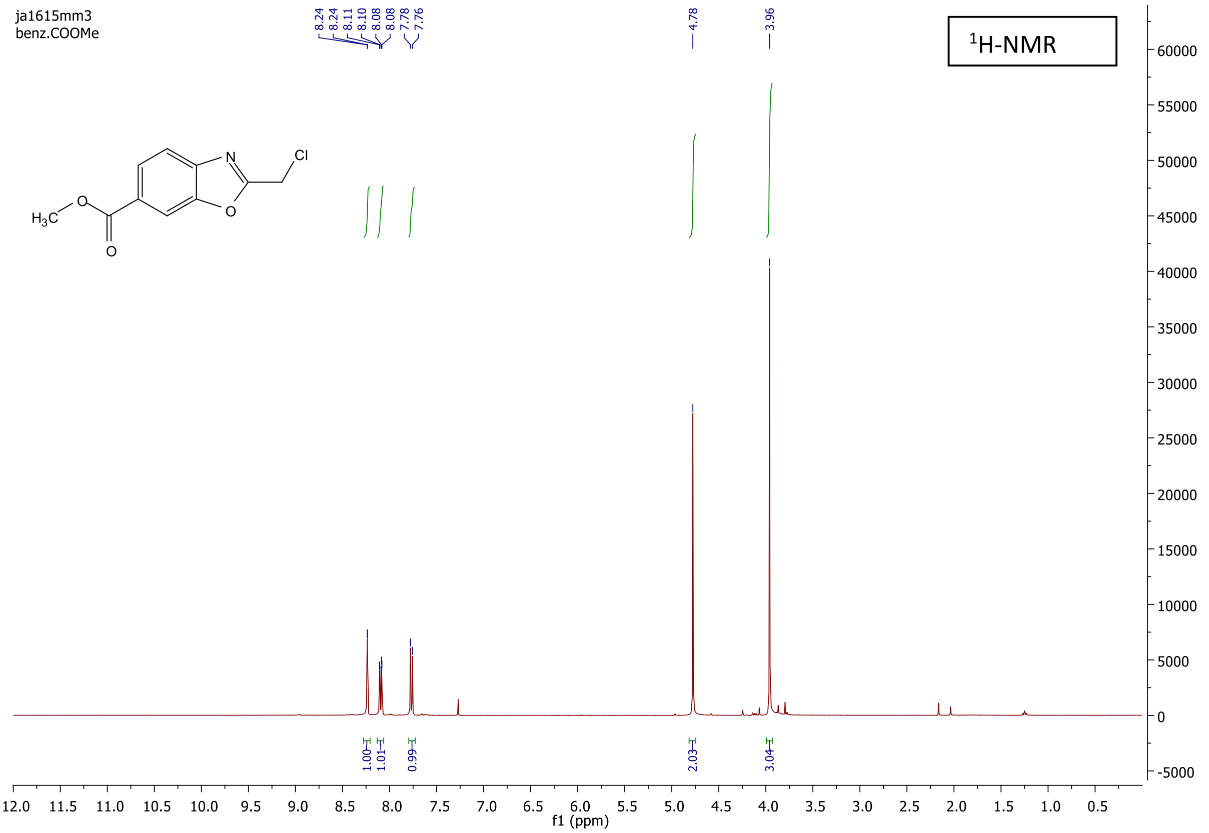
ja1615mm3
benz.COOMe
8.24
8.24
8.11
8.10
8.08
8.08
7.78
7.76
4.78
3.96
1H-NMR
N
Cl
O
H3C
O
O
1.00
1.01
0.99
2.03
3.04
f1 (ppm)

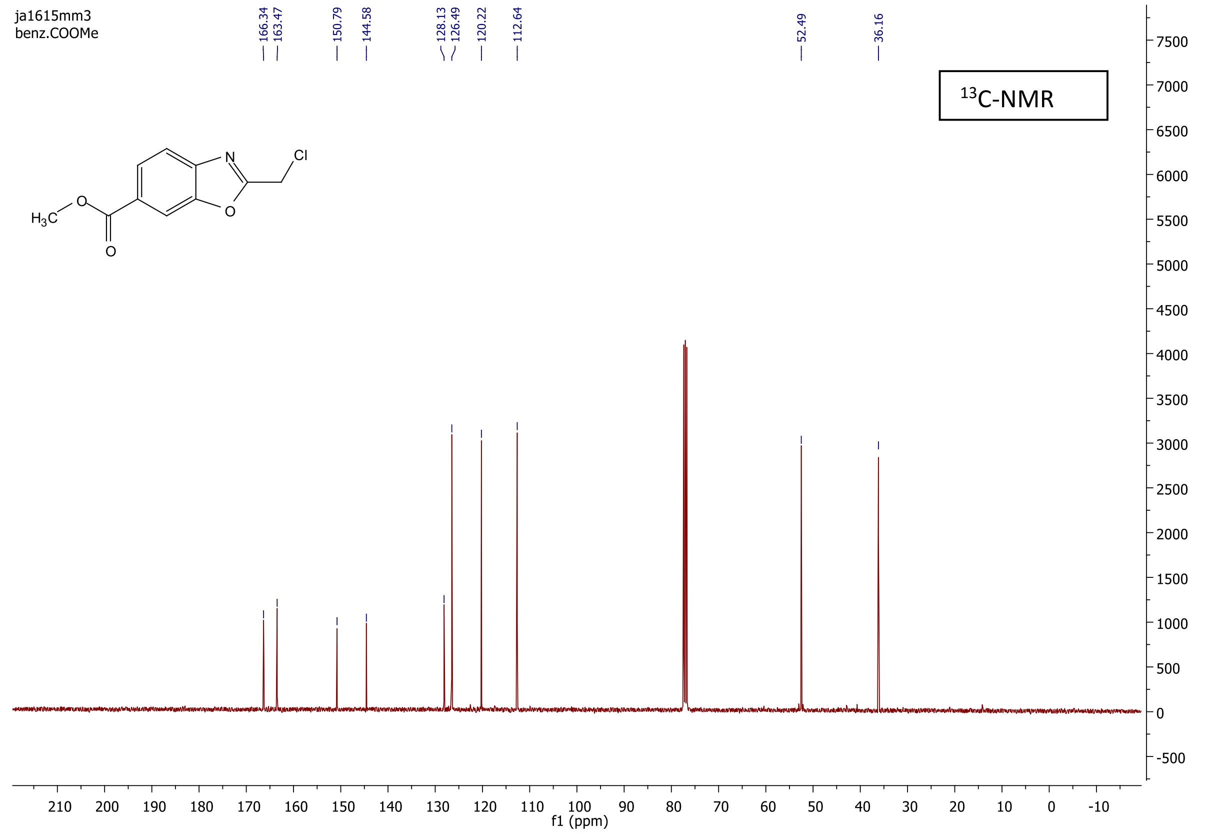
ja1615mm3
benz.COOMe
166.34
163.47
150.79
144.58
128.13
126.49
120.22
112.64
52.49
36.16
13C-NMR
N
Cl
O
H3C
O
O
f1 (ppm)

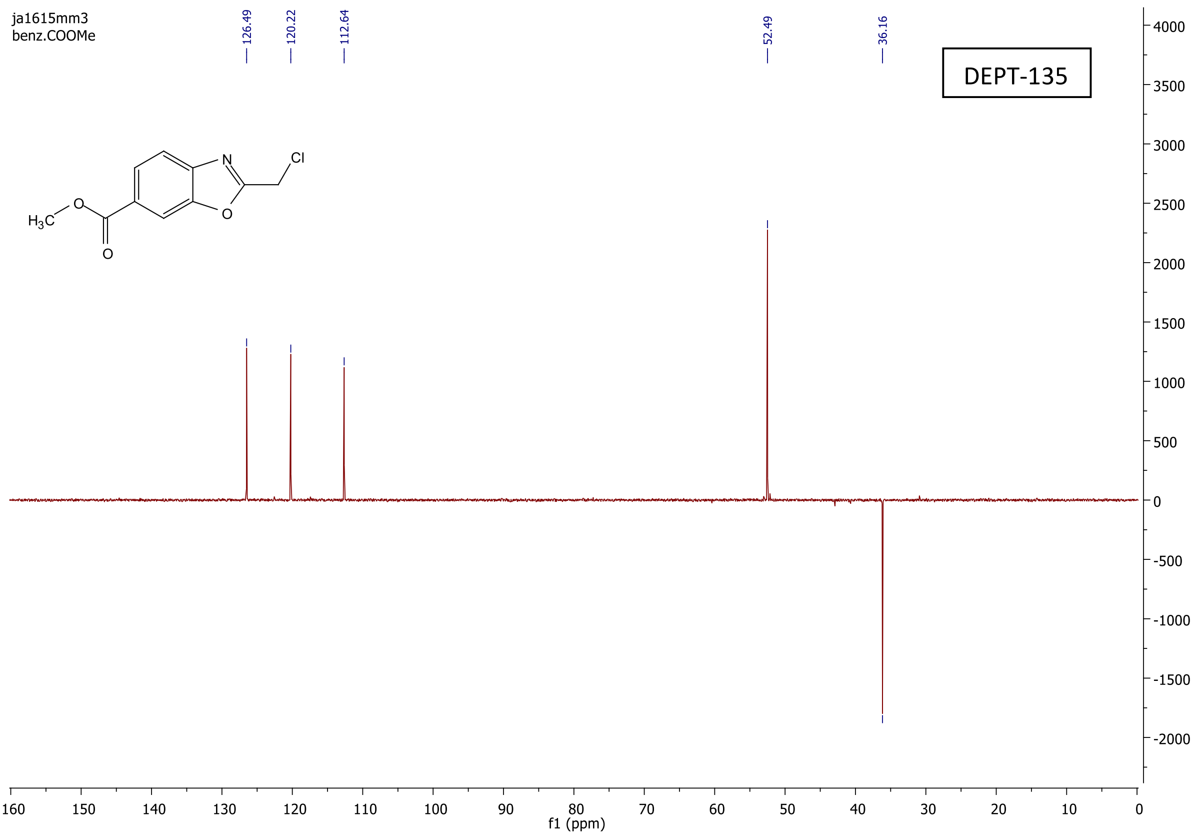
ja1615mm3
benz.COOMe
126.49
120.22
112.64
52.49
36.16
DEPT-135
N
Cl
O
H_3C
O
O
4000
3500
3000
2500
2000
1500
1000
500
0
-500
-1000
-1500
-2000
160 150 140 130 120 110 100 90 80 70 60 50 40 30 20 10 0
f1 (ppm)

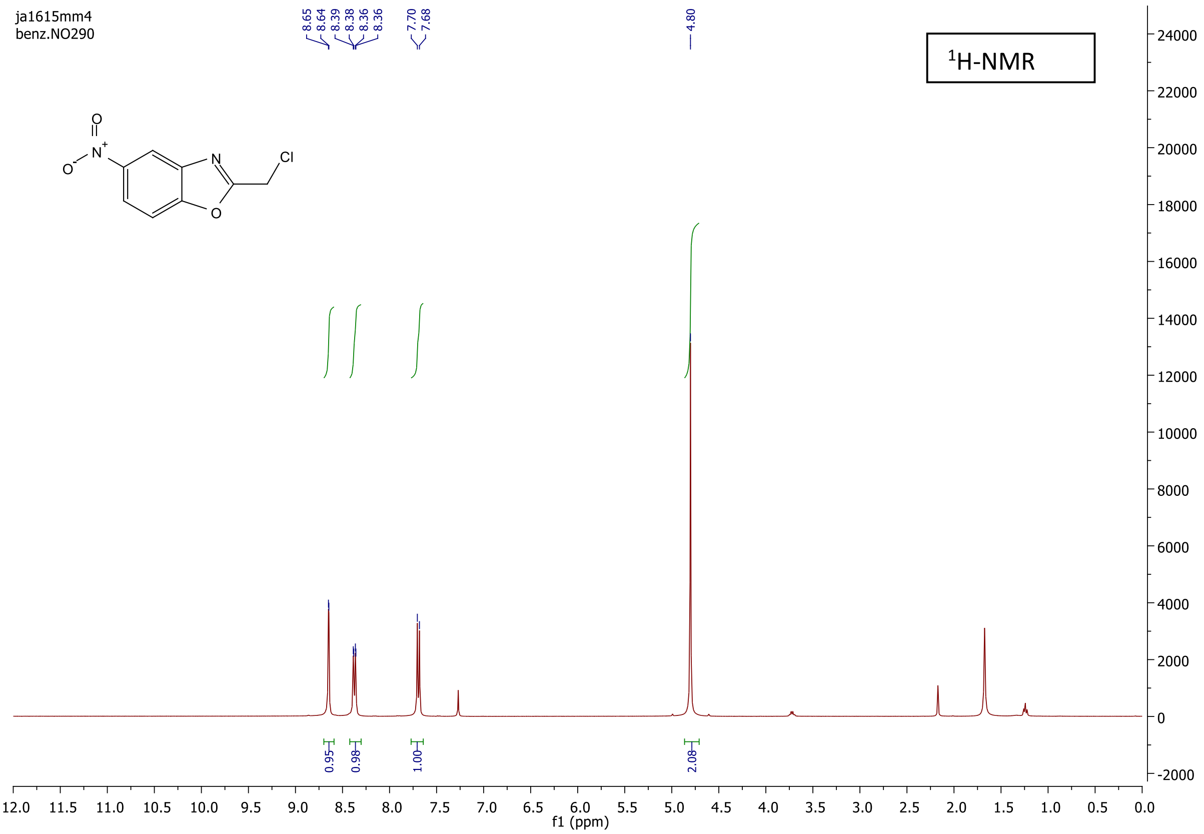
ja1615mm4
benz.NO290
8.65
8.64
8.39
8.38
8.36
8.36
7.70
7.68
4.80
^{1}H-NMR
O
N^+
O^-
N
Cl
O
24000
22000
20000
18000
16000
14000
12000
10000
8000
6000
4000
2000
0
-2000
0.95
0.98
1.00
2.08
12.0 11.5 11.0 10.5 10.0 9.5 9.0 8.5 8.0 7.5 7.0 6.5 6.0 5.5 5.0 4.5 4.0 3.5 3.0 2.5 2.0 1.5 1.0 0.5 0.0
f1 (ppm)

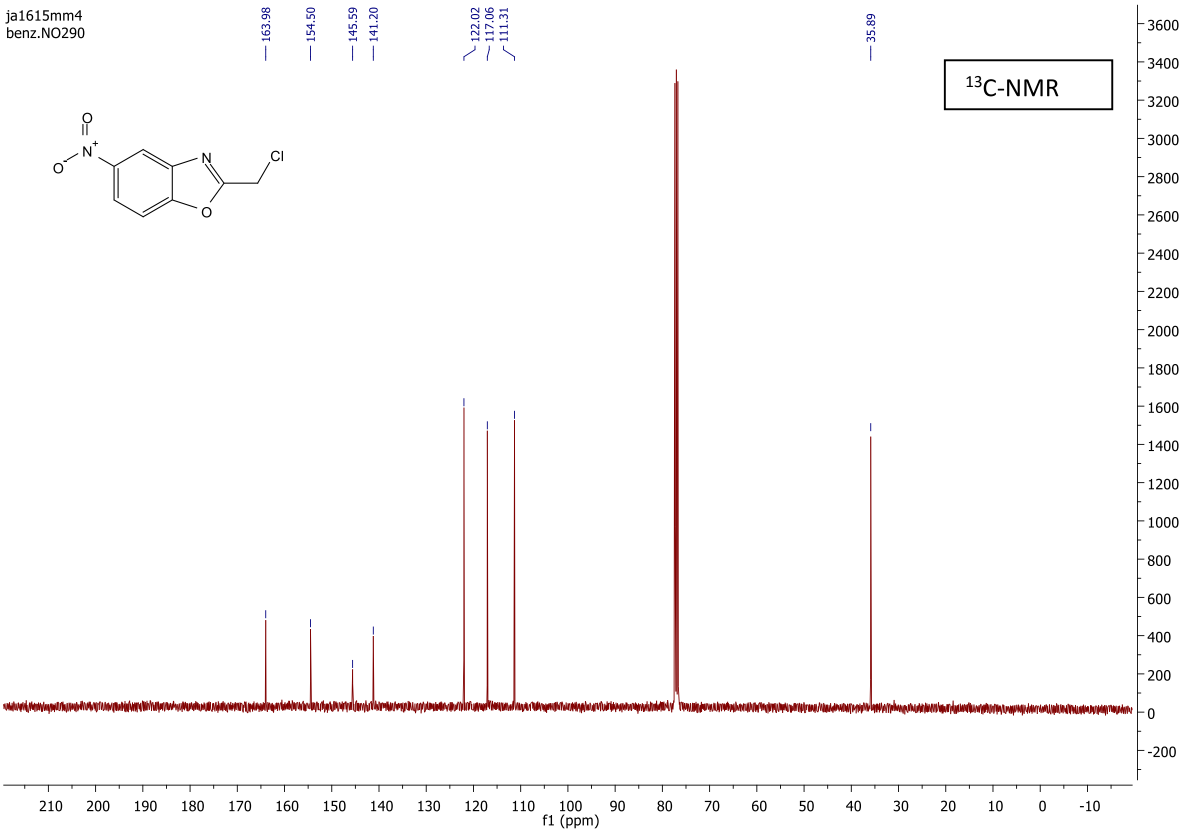
ja1615mm4
benz.NO290
163.98
154.50
145.59
141.20
122.02
117.06
111.31
35.89
^{13}C-NMR
O
N
O
N
Cl
O
f1 (ppm)

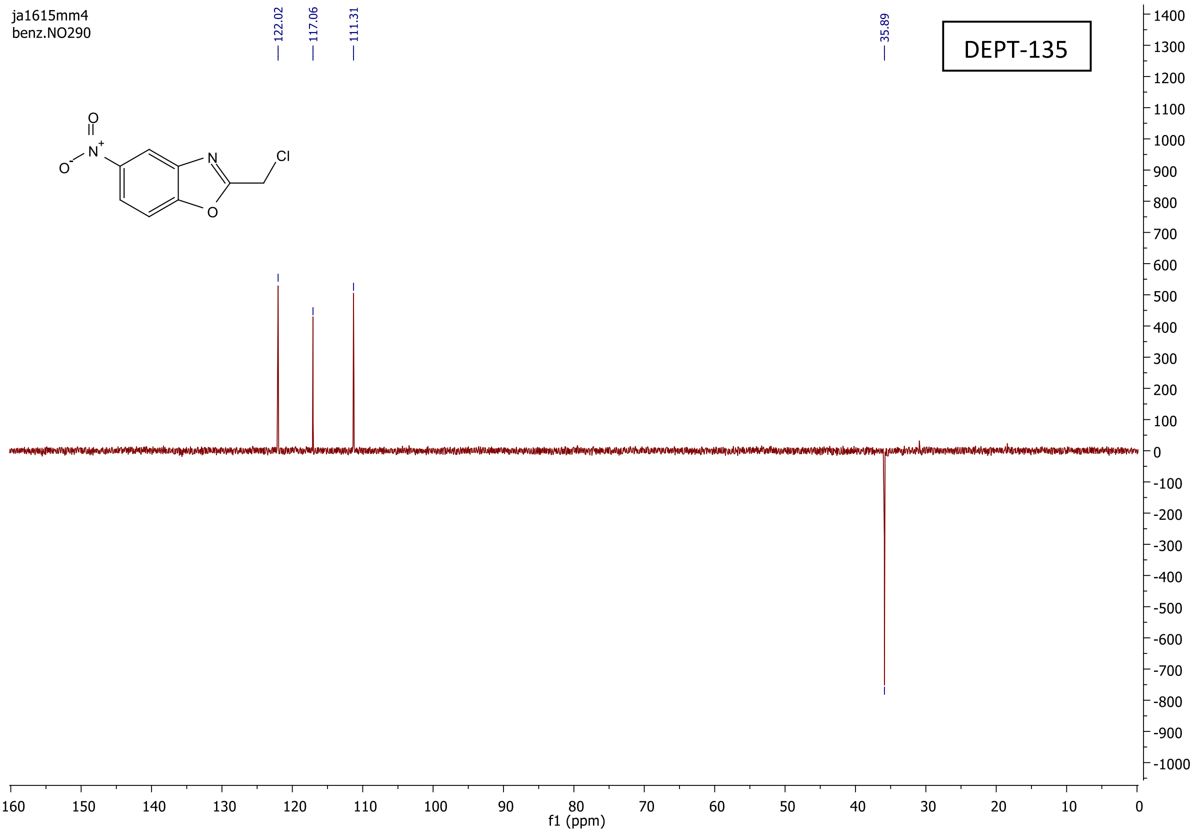
ja1615mm4
benz.NO290
122.02
117.06
111.31
35.89
DEPT-135
O
N
O
N
Cl
O
f1 (ppm)

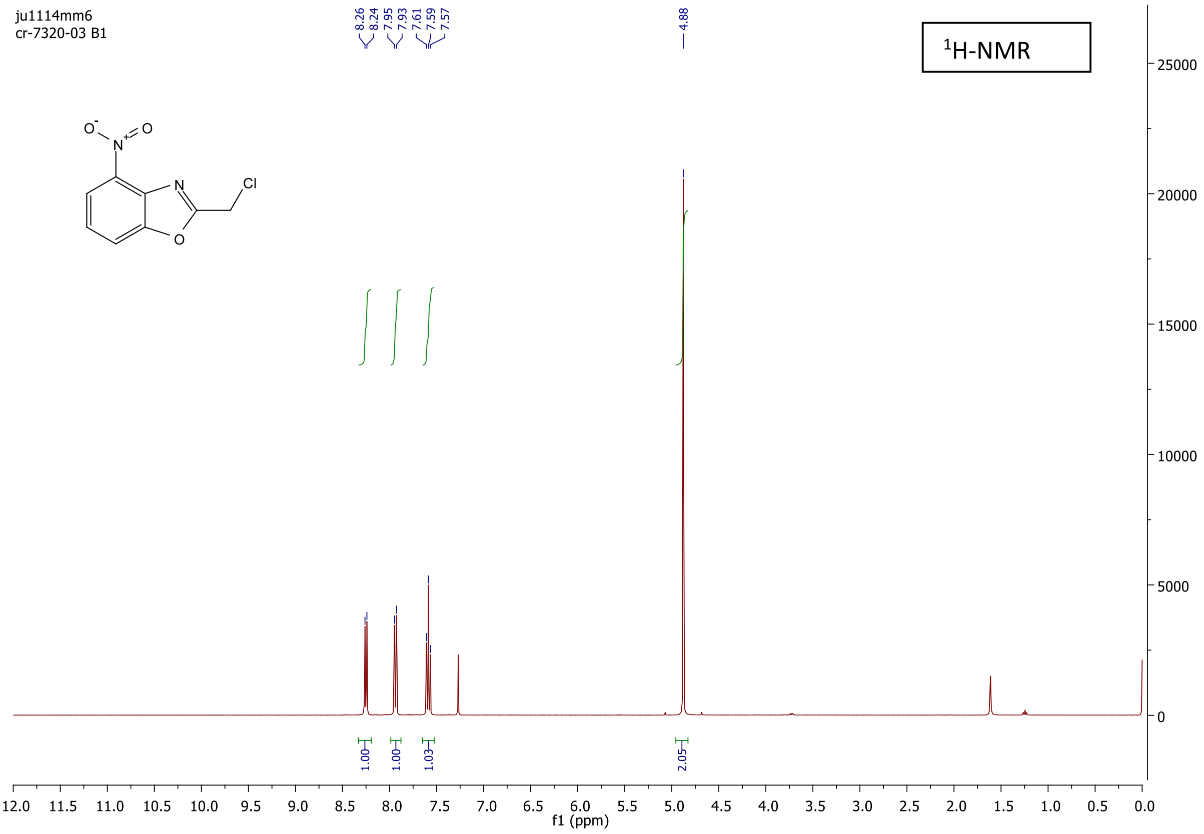
ju1114mm6
cr-7320-03 B1
8.26
8.24
7.95
7.93
7.61
7.59
7.57
4.88
^{1}H-NMR
1.00
1.00
1.03
2.05
f1 (ppm)

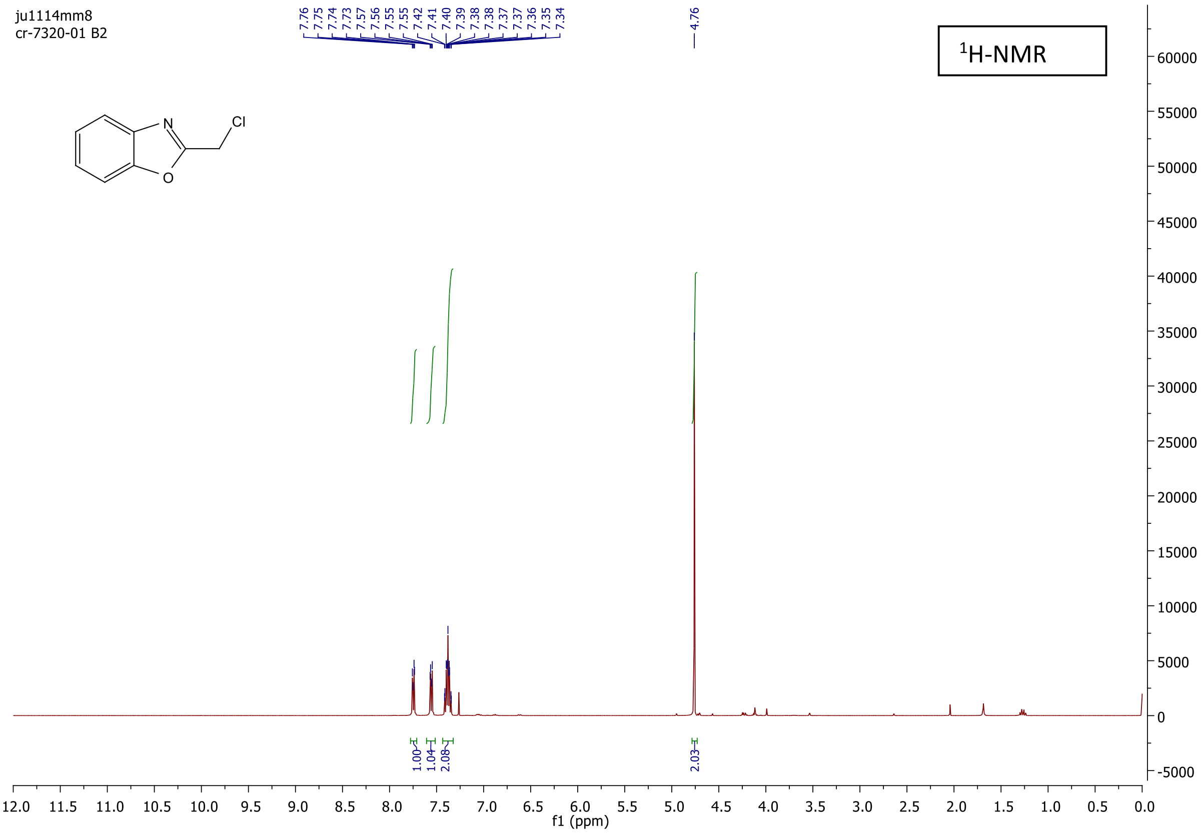
ju1114mm8
cr-7320-01 B2
7.76
7.75
7.74
7.73
7.57
7.56
7.55
7.55
7.42
7.41
7.40
7.39
7.38
7.38
7.37
7.37
7.36
7.35
7.34
4.76
^{1}H-NMR
1.00
1.04
2.08
2.03
f1 (ppm)

20. NMR cyclopropanes

Product **4a** major diastereomer:

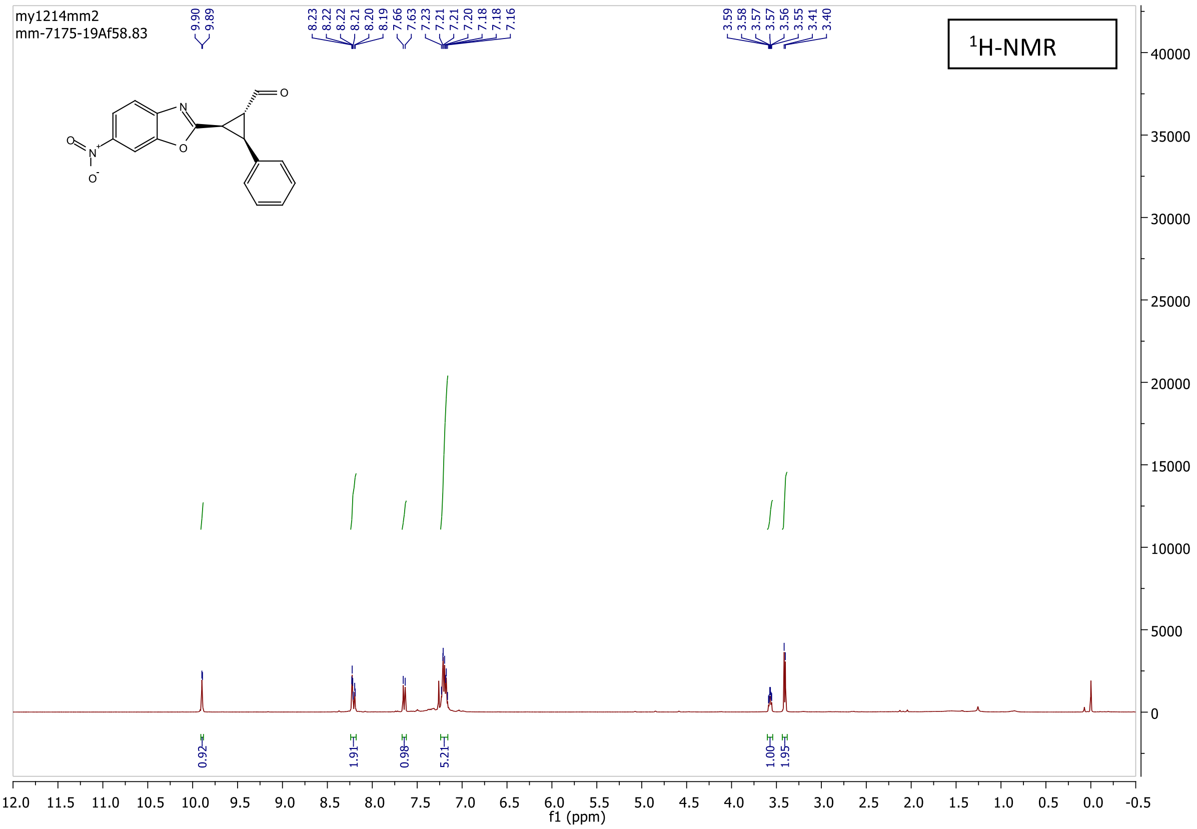

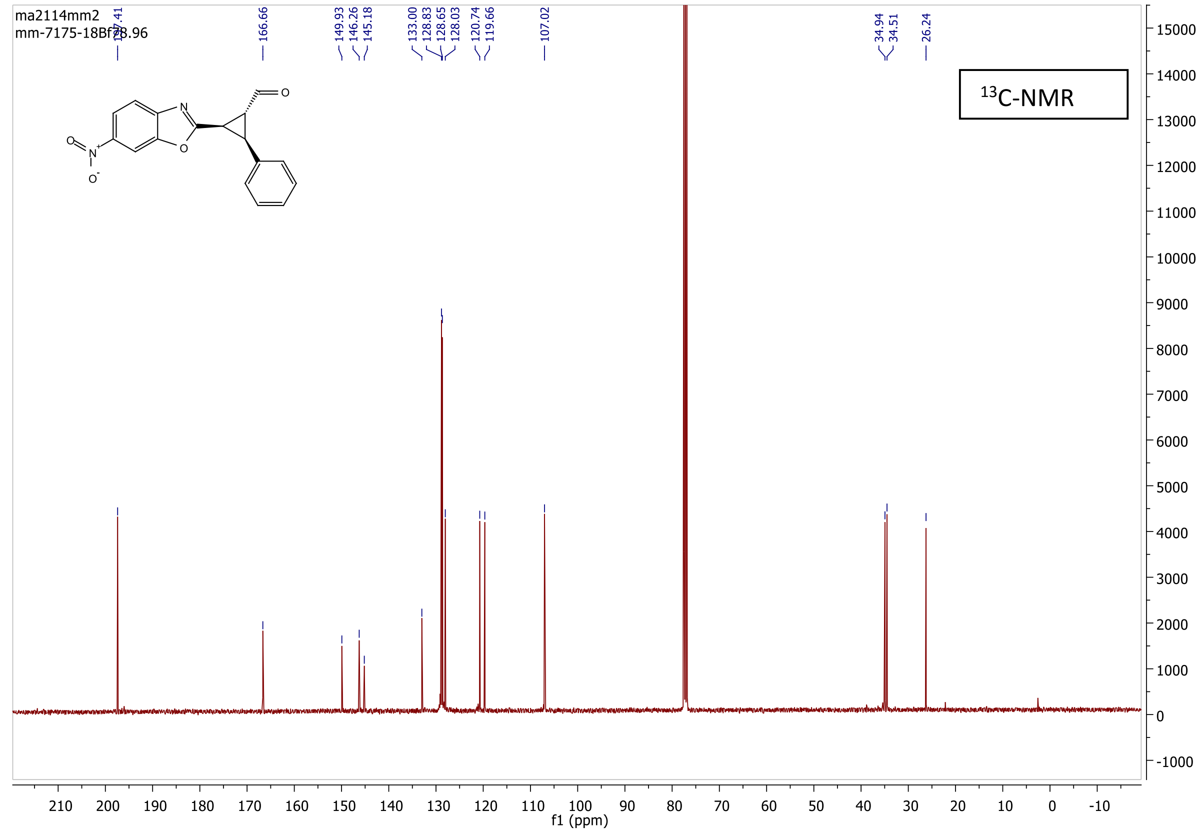

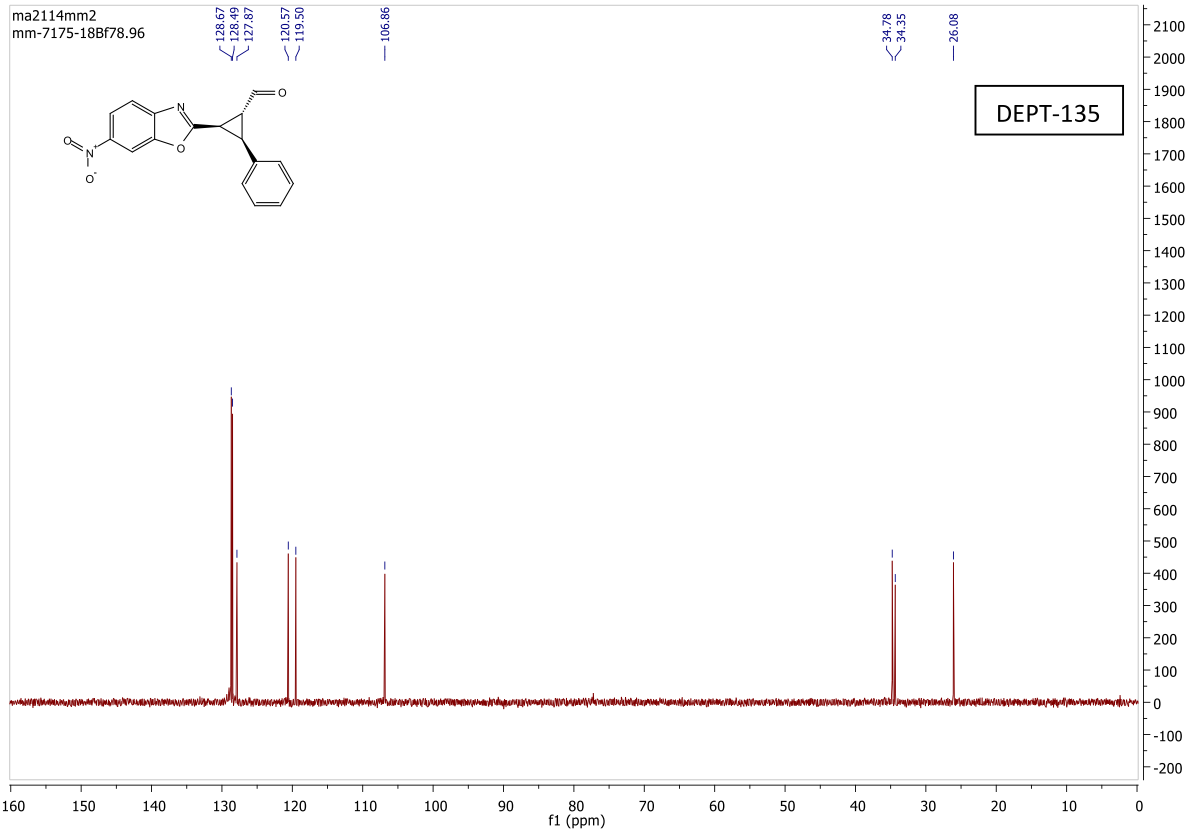

Product **4a** minor diastereomer:

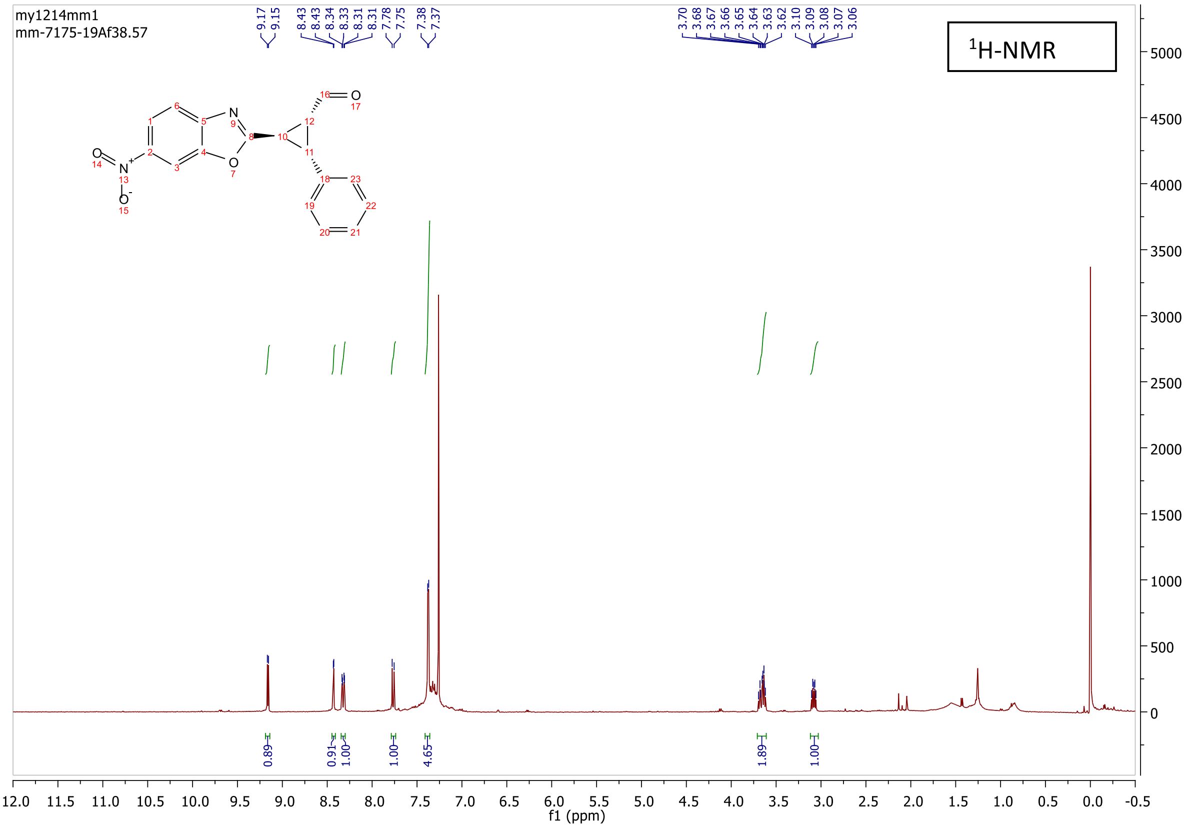

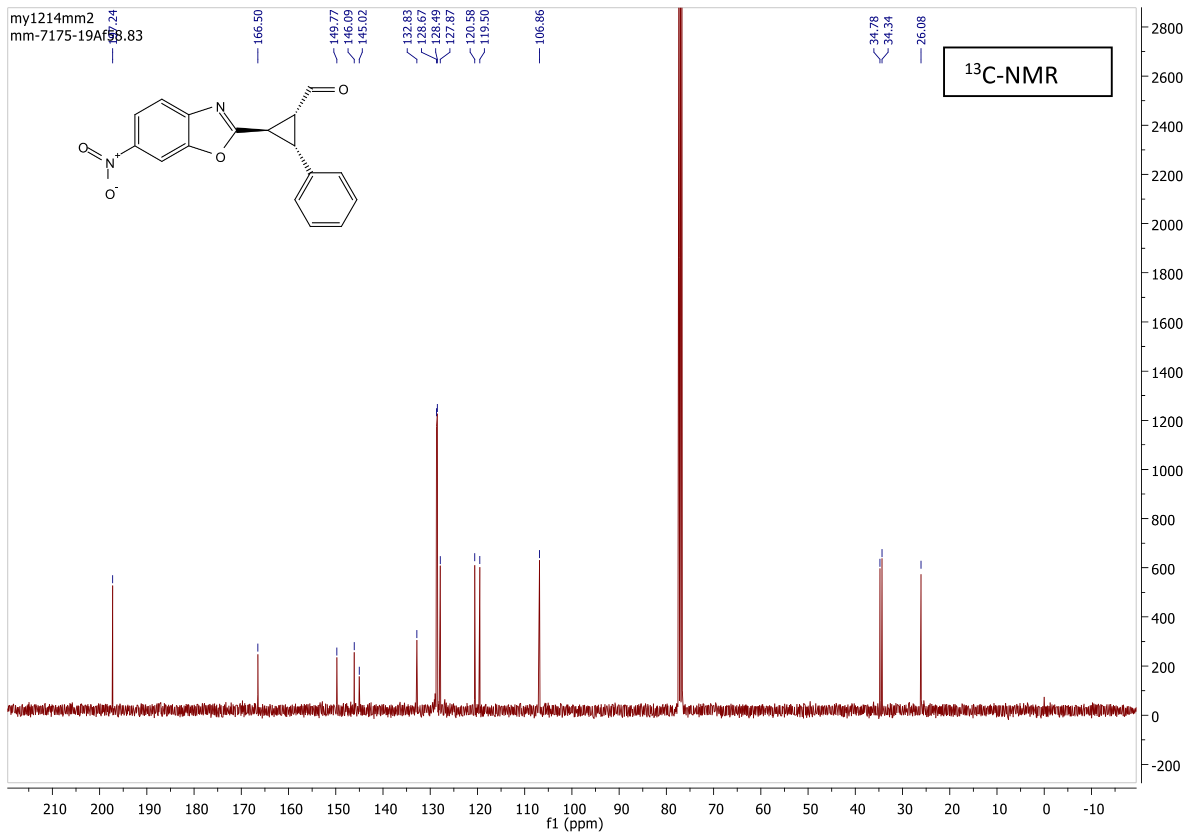
my1214mm2
mm-7175-19Af58.83
166.50
149.77
146.09
145.02
132.83
128.67
128.49
127.87
120.58
119.50
106.86
34.78
34.34
26.08
13C-NMR
f1 (ppm)

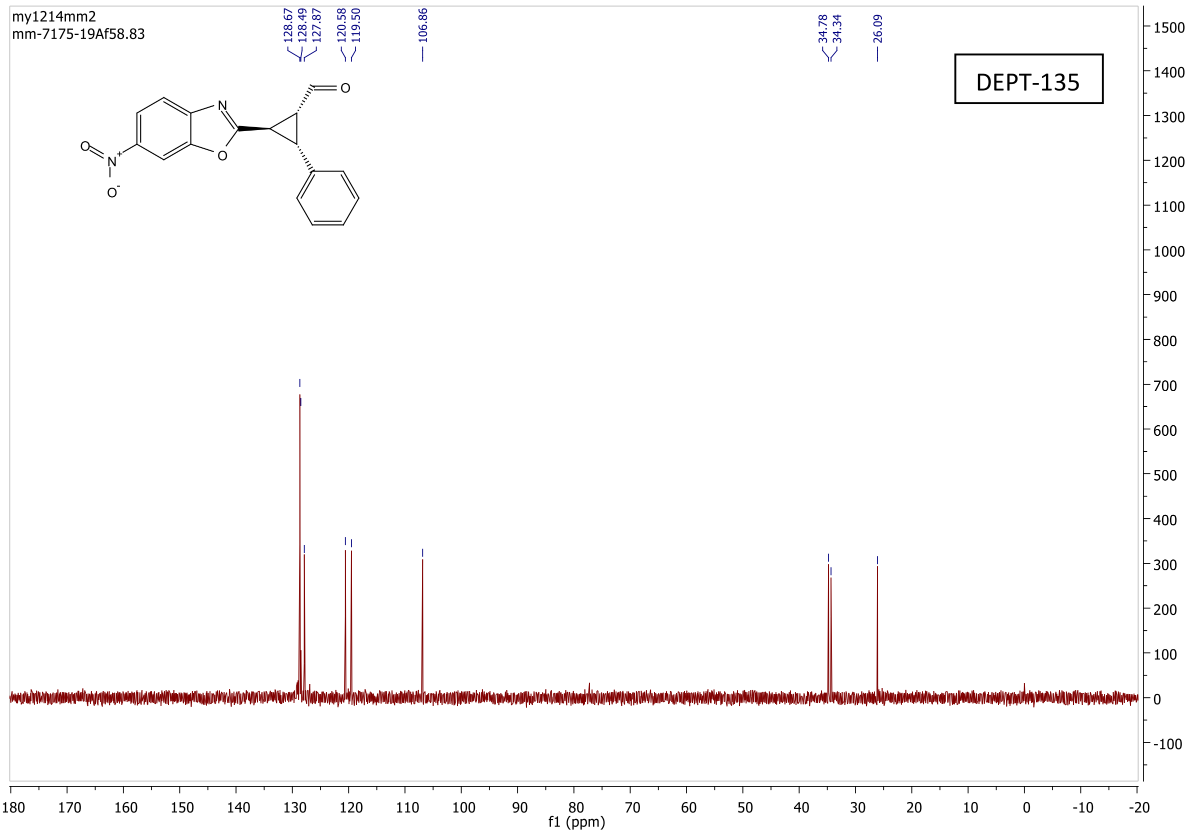
my1214mm2
mm-7175-19Af58.83
128.67
128.49
127.87
120.58
119.50
106.86
34.78
34.34
26.09
DEPT-135
f1 (ppm)

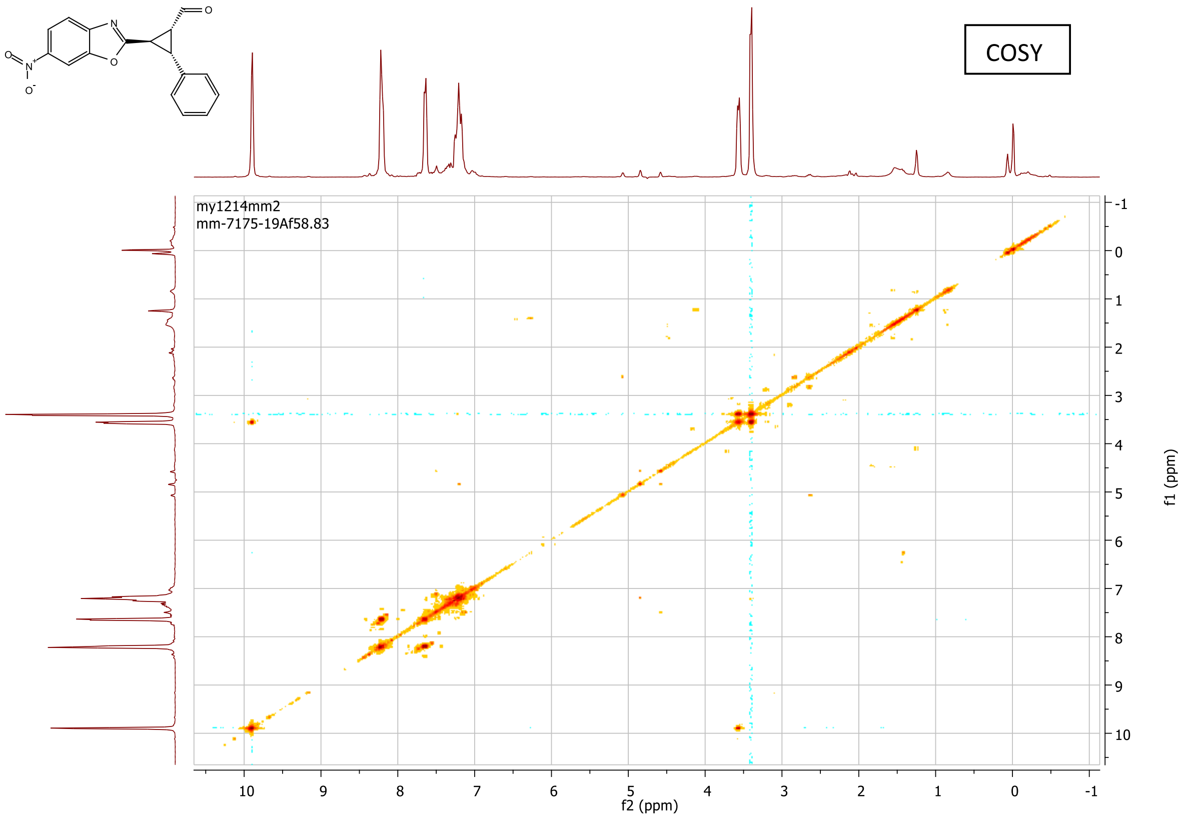
COSY
my1214mm2
mm-7175-19Af58.83
f2 (ppm)
f1 (ppm)

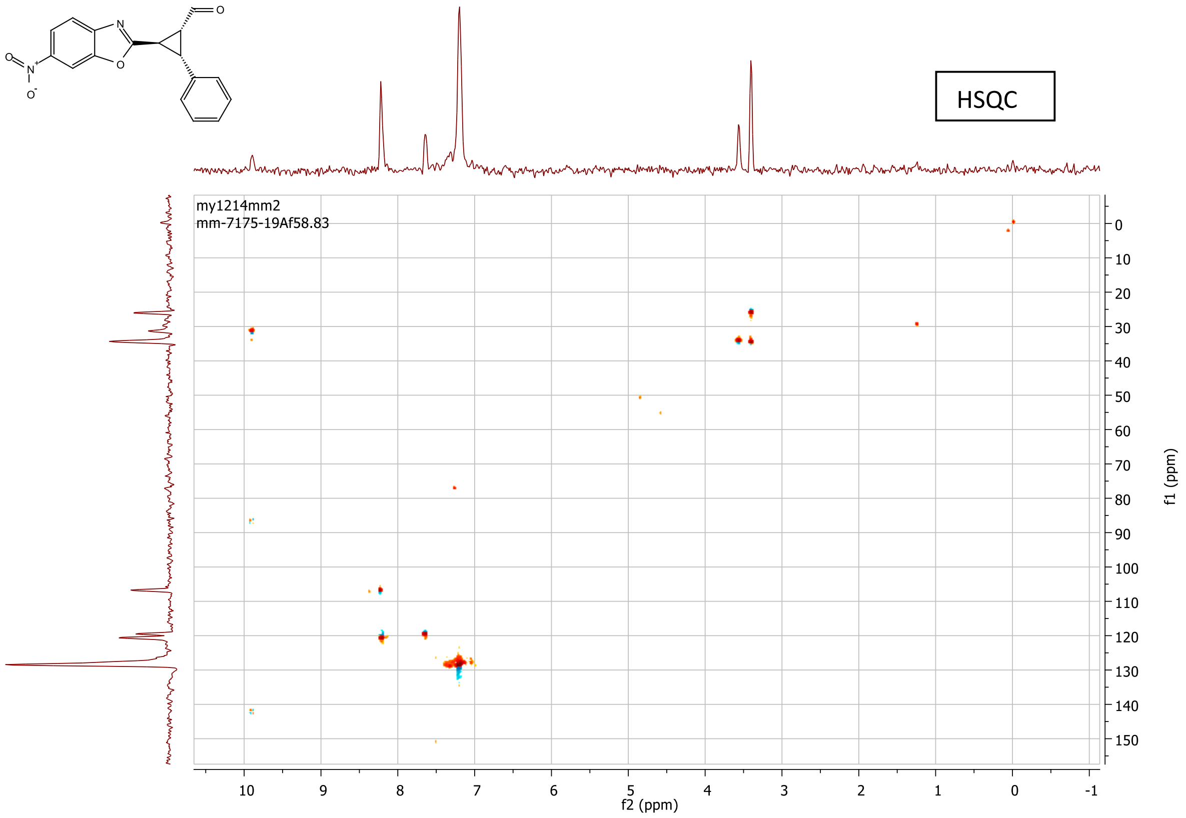
HSQC
my1214mm2
mm-7175-19Af58.83
f2 (ppm)
f1 (ppm)

Product **4a** mixture of minor and minor':

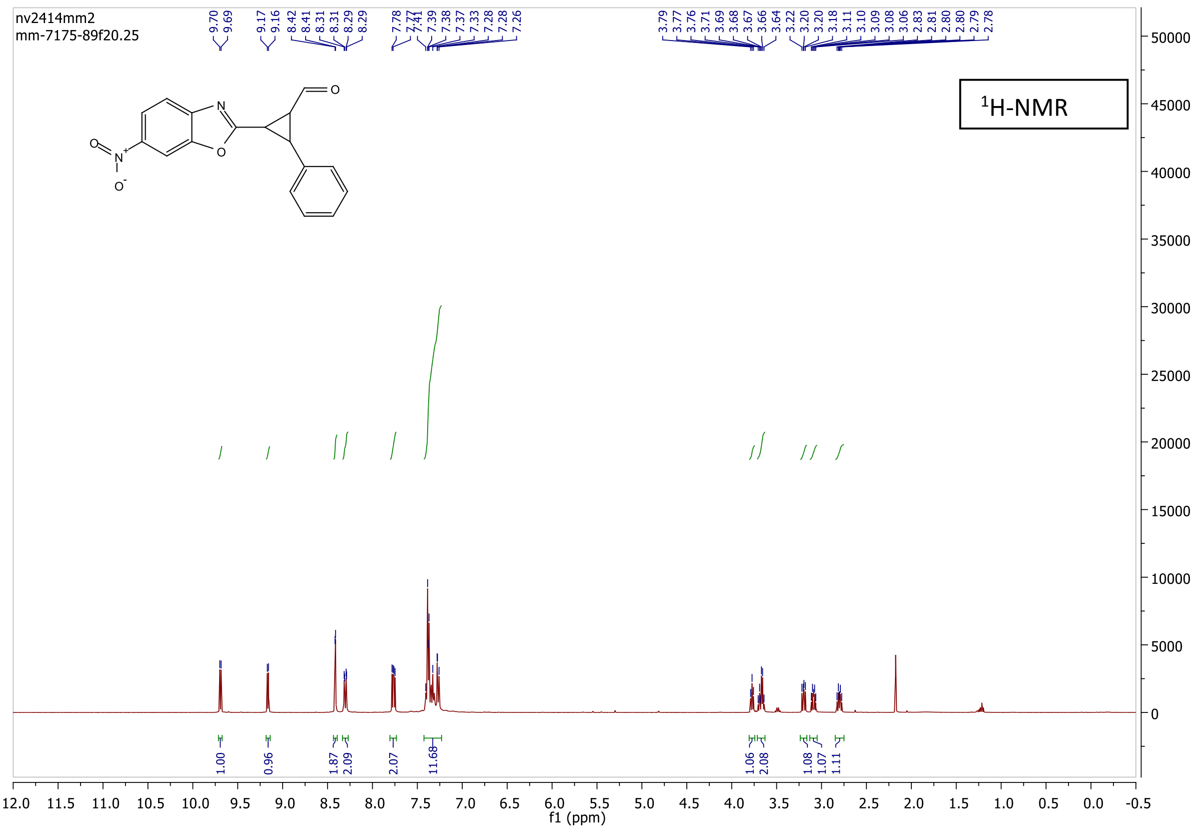

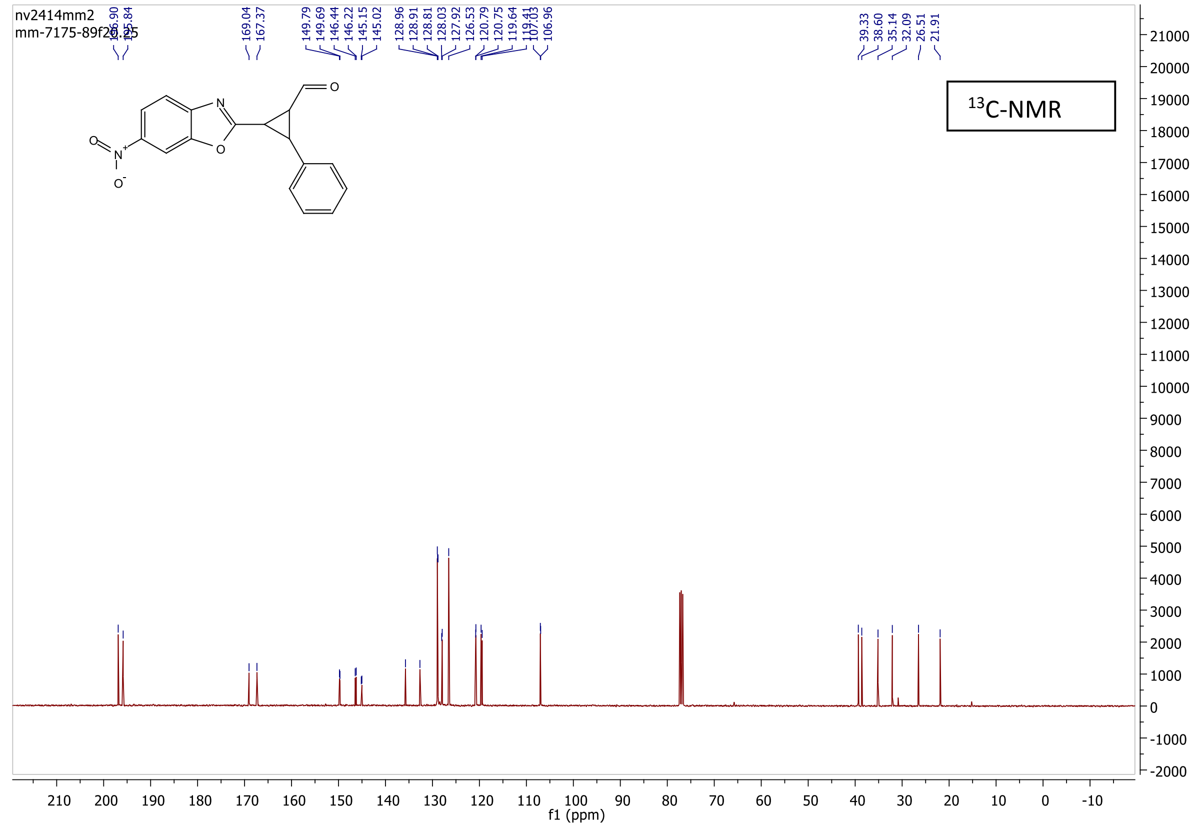

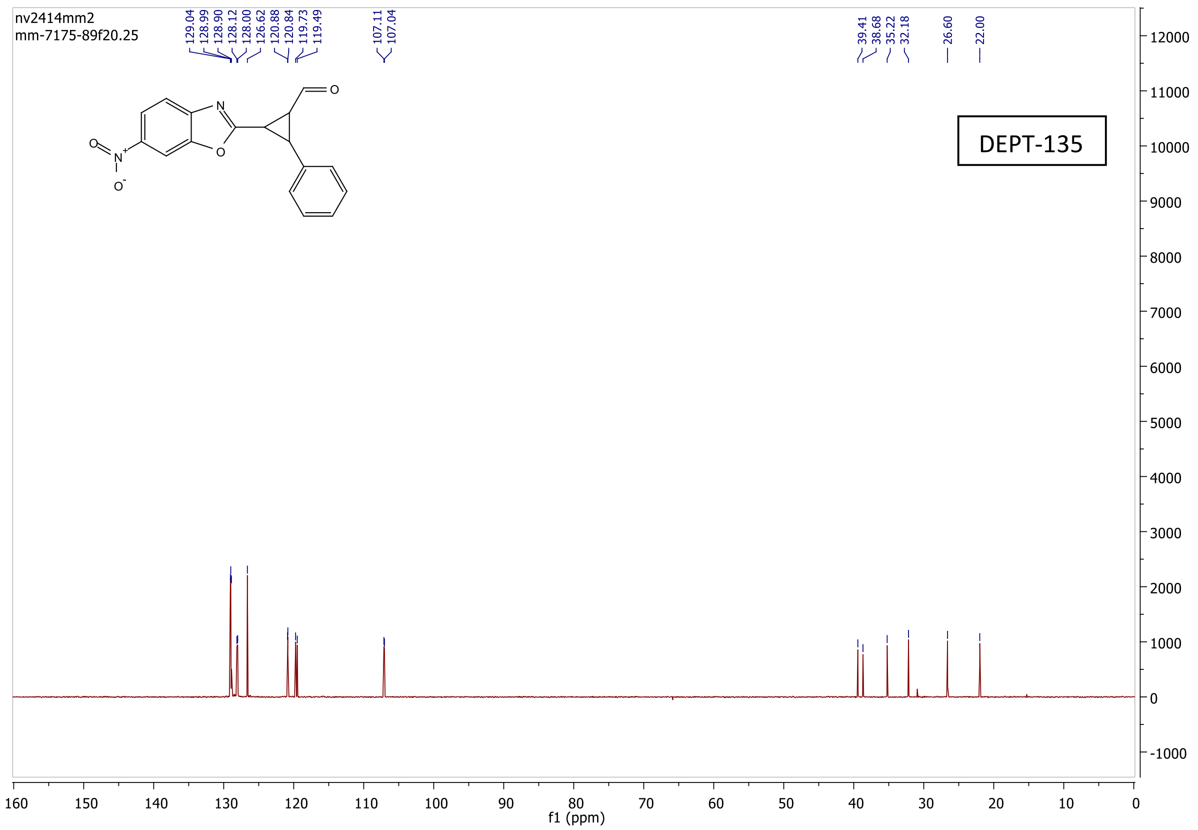

Product **4b** major diastereomer:

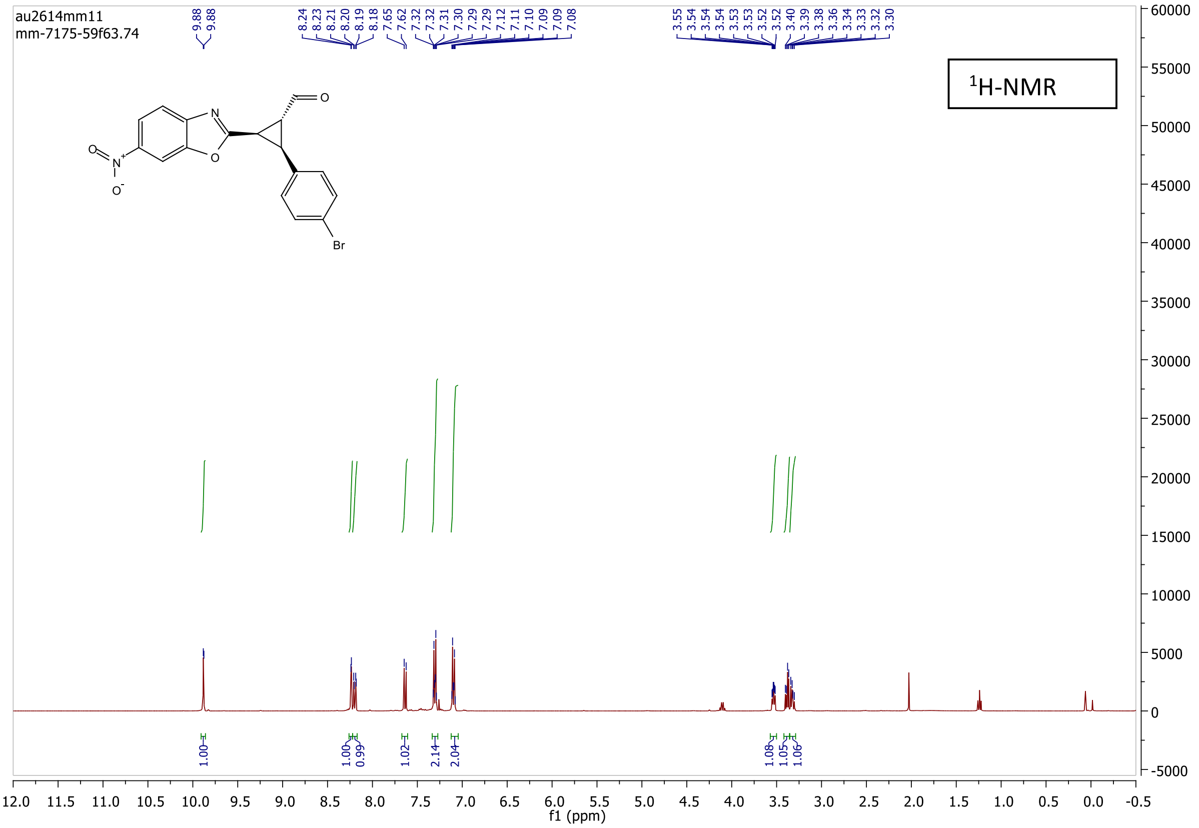

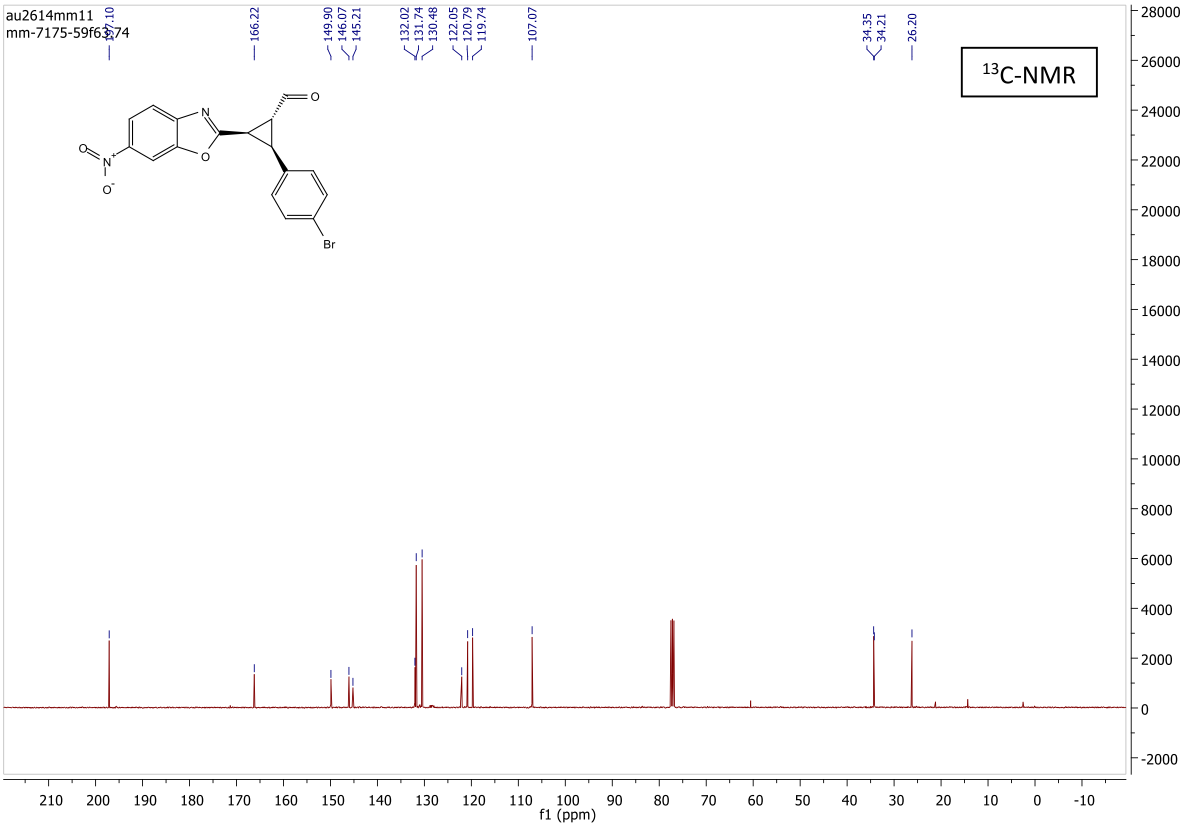
au2614mm11
mm-7175-59f63.74
197.10
166.22
149.90
146.07
145.21
132.02
131.74
130.48
122.05
120.79
119.74
107.07
34.35
34.21
26.20
^{13}C-NMR
f1 (ppm)
Br
N
O

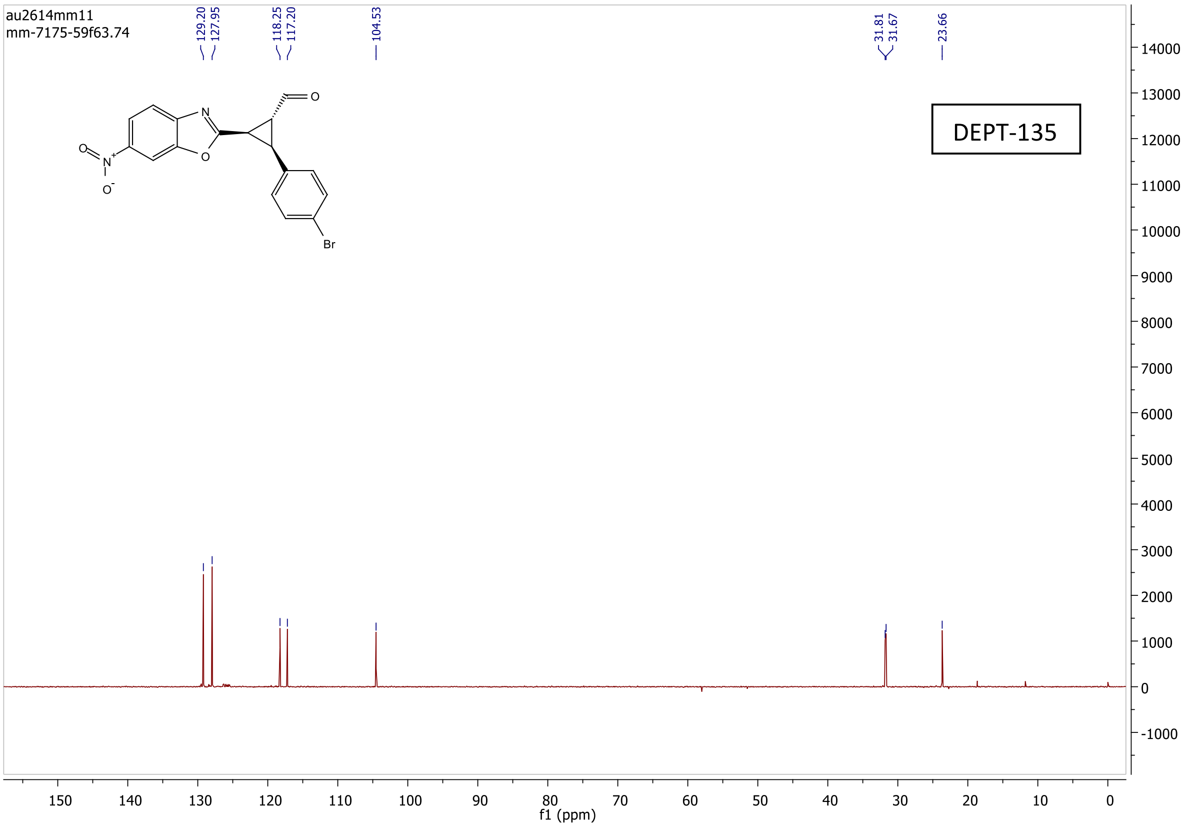
au2614mm11
mm-7175-59f63.74
129.20
127.95
118.25
117.20
104.53
31.81
31.67
23.66
DEPT-135
f1 (ppm)
Br
N
O

Product **4b** minor diastereomer:

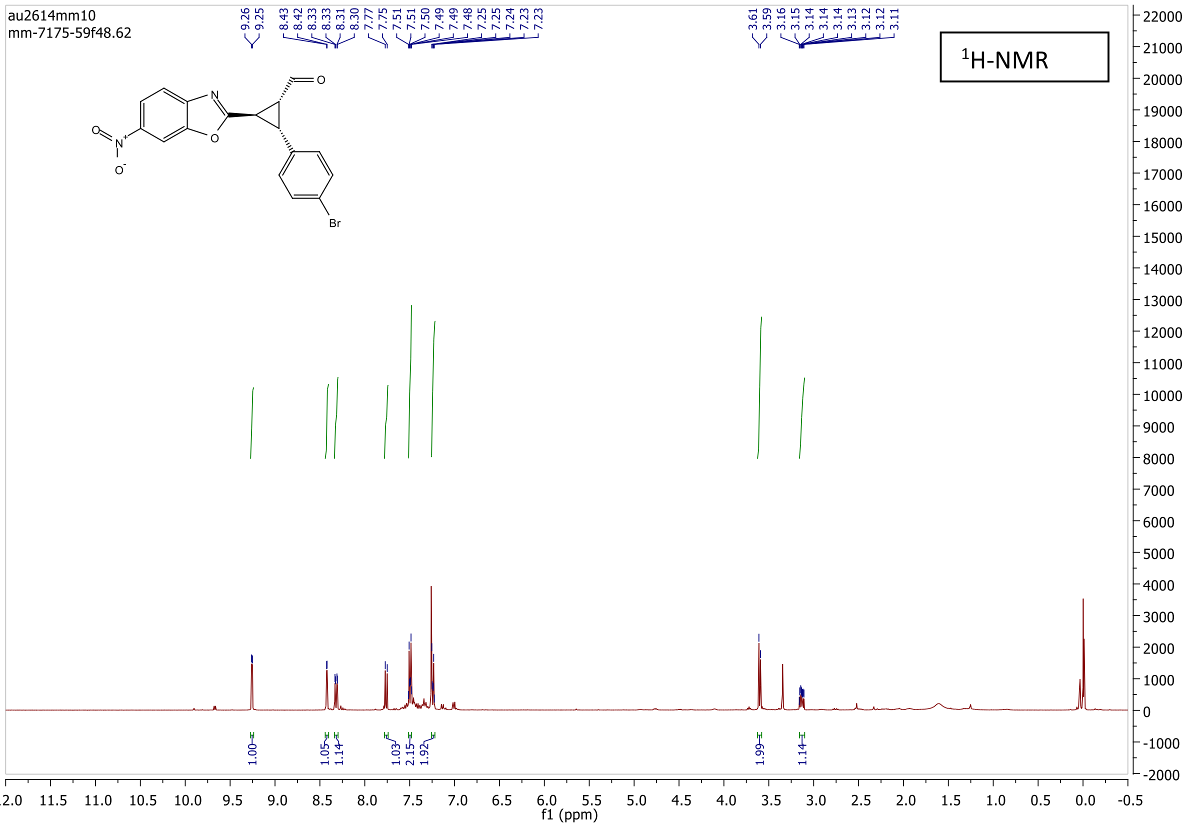

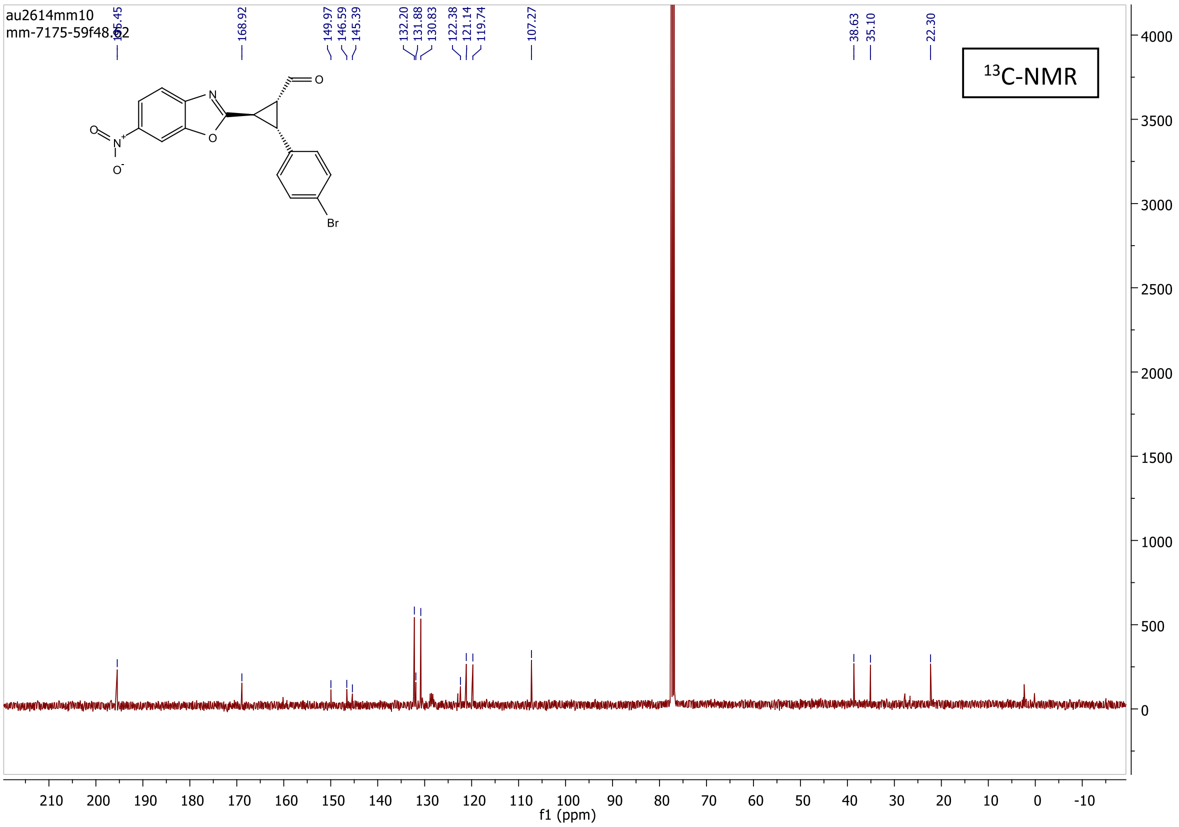

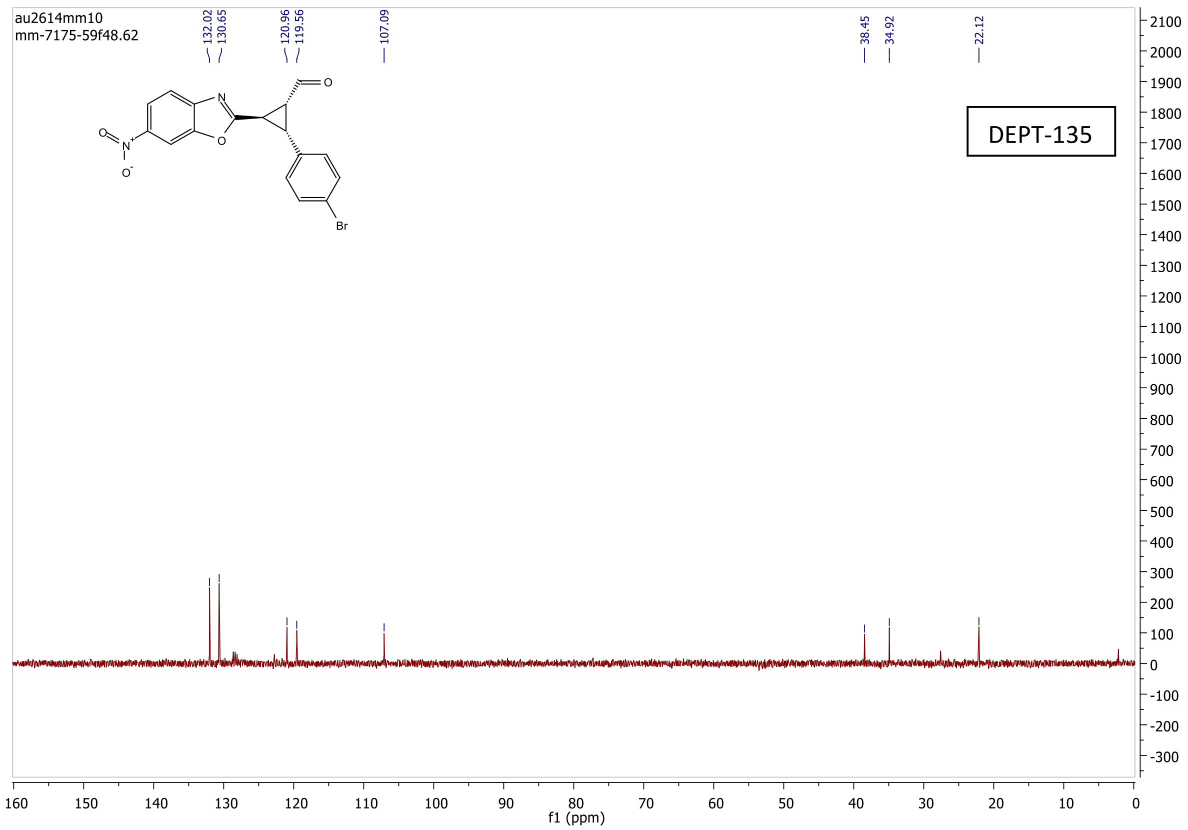

Product **4b** mixture of minor and minor' diastereomers:

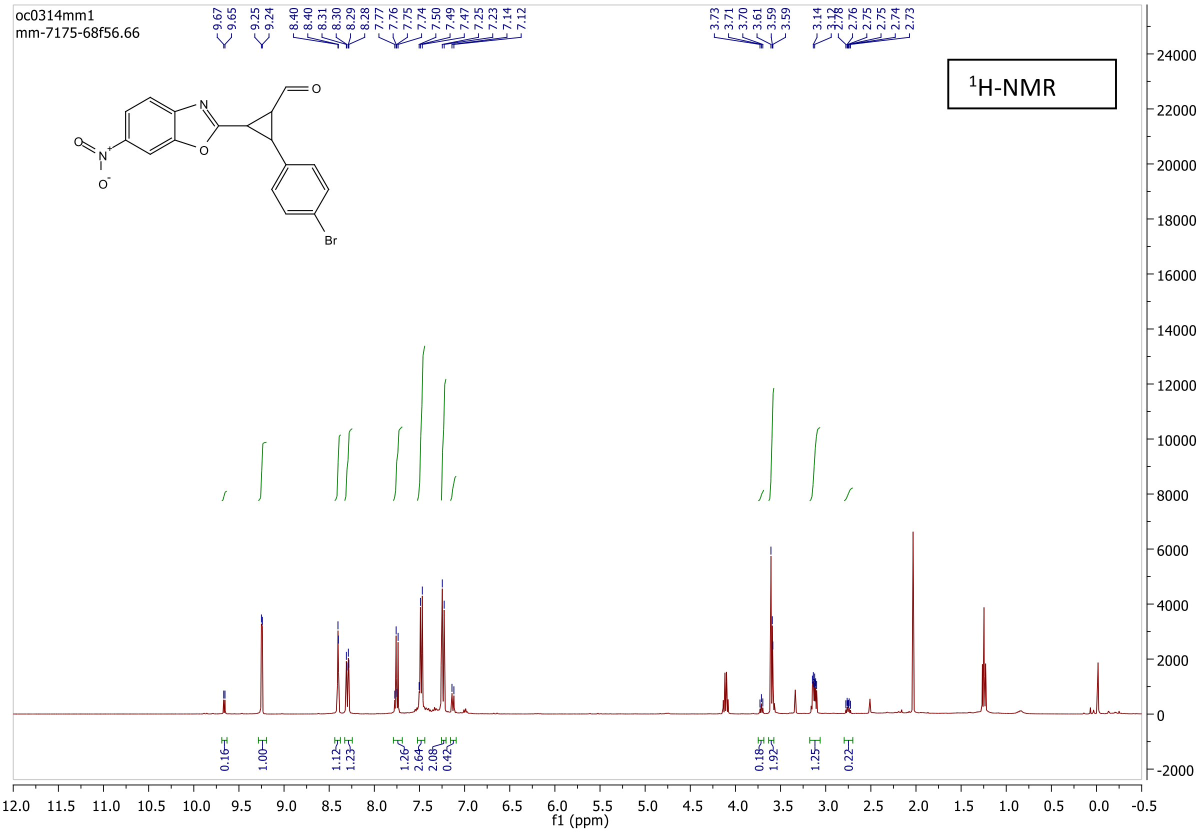

Product **4c** major diastereomer:

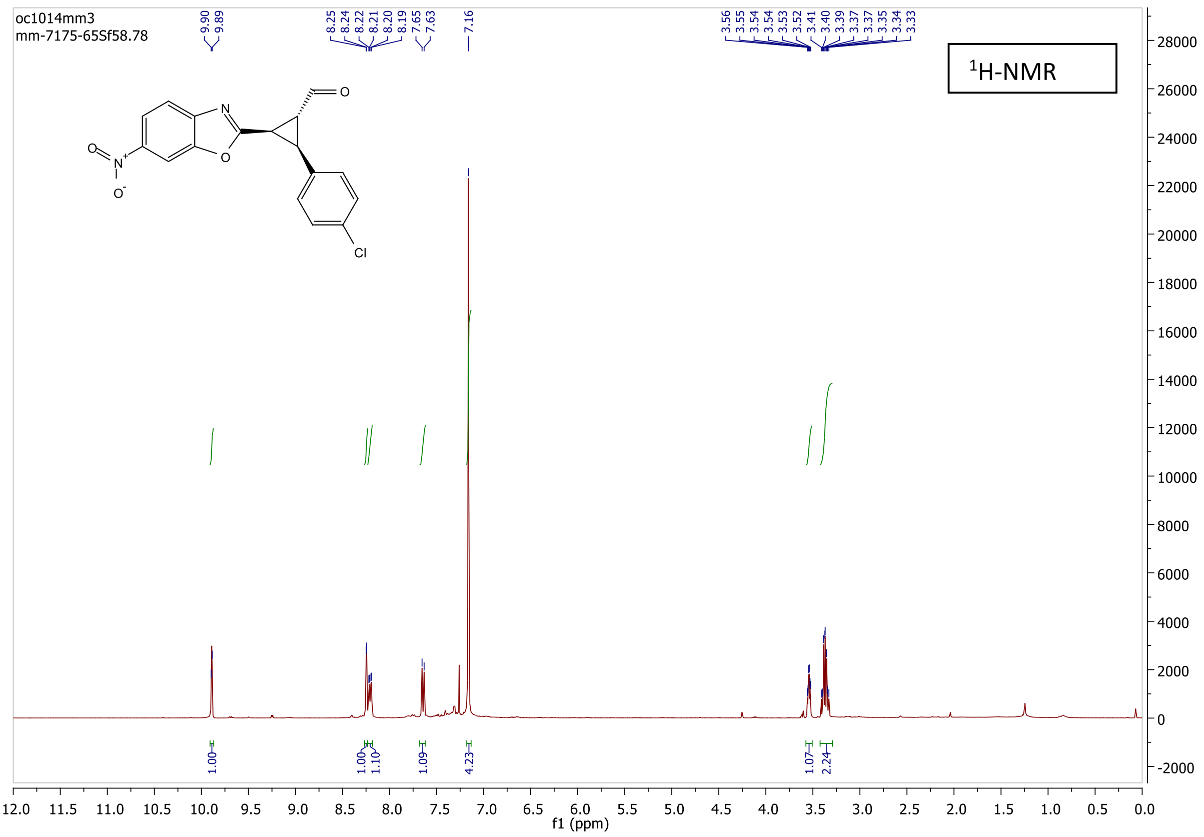

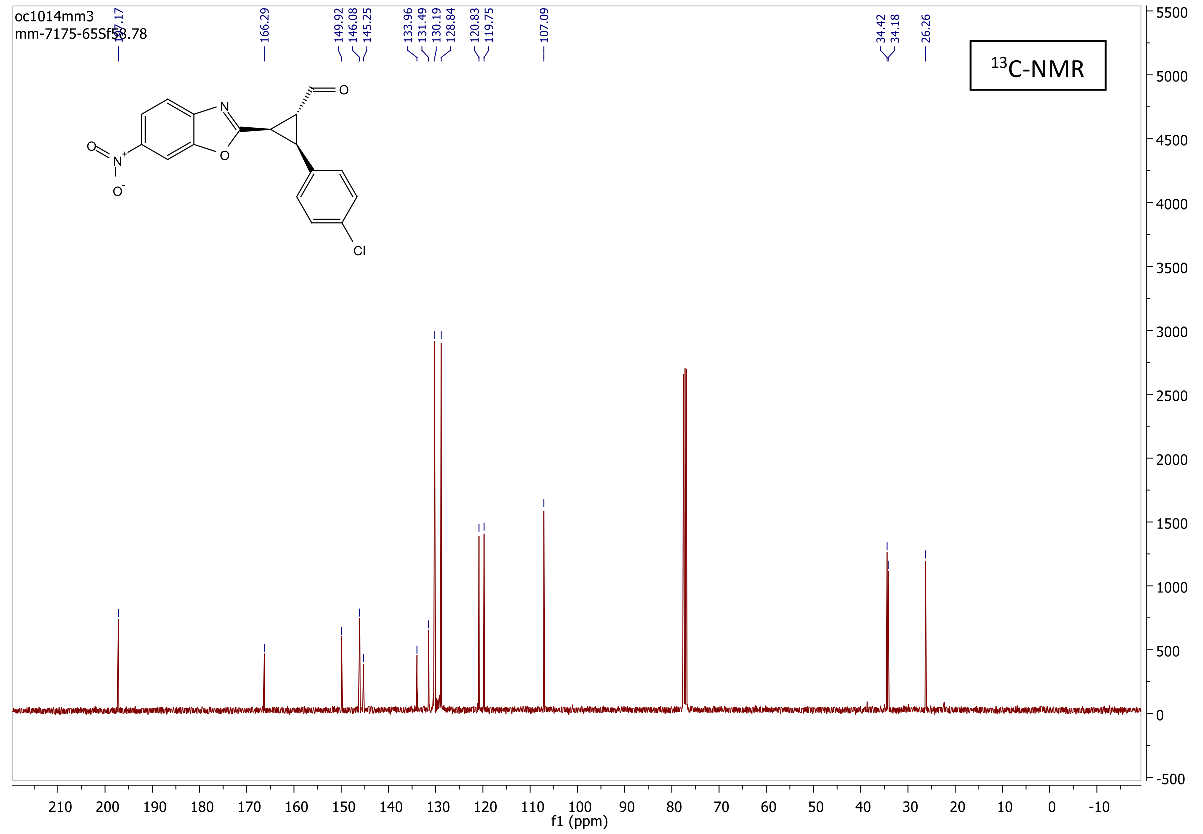

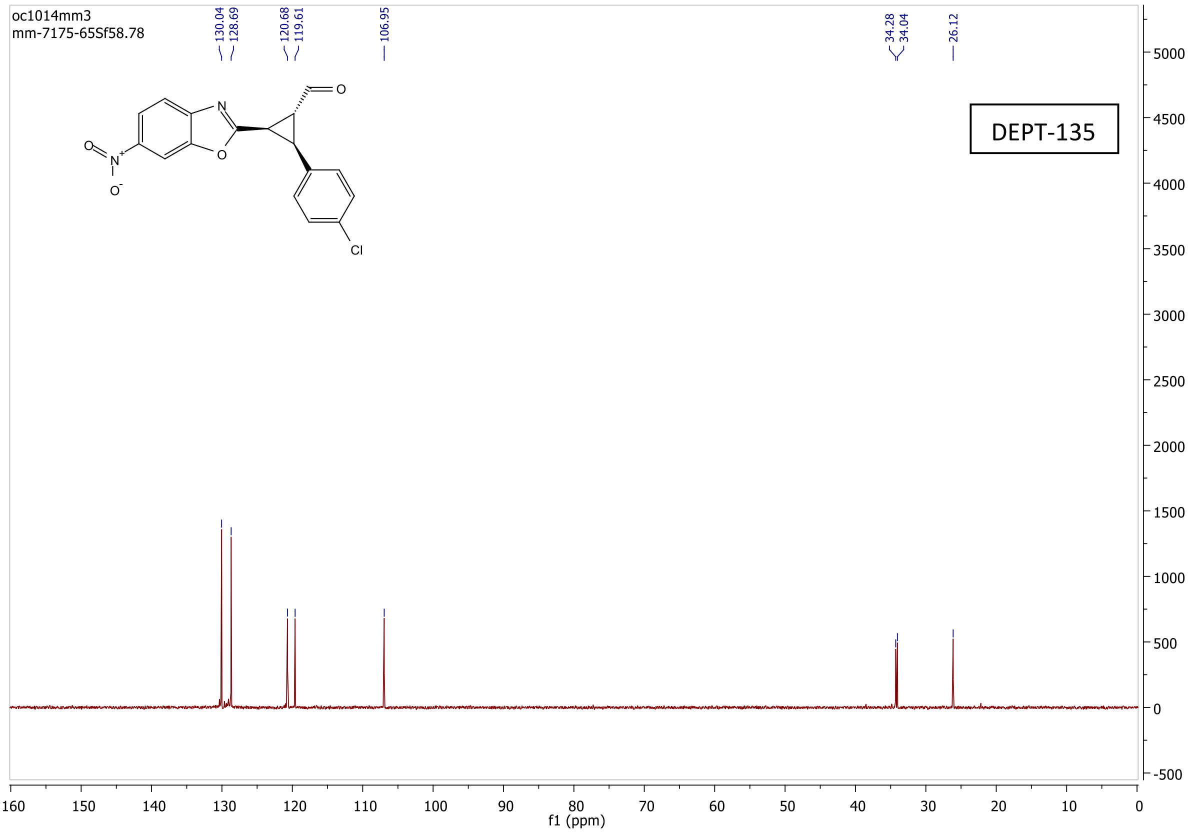

Product **4c** minor diastereomer with traces of minor':

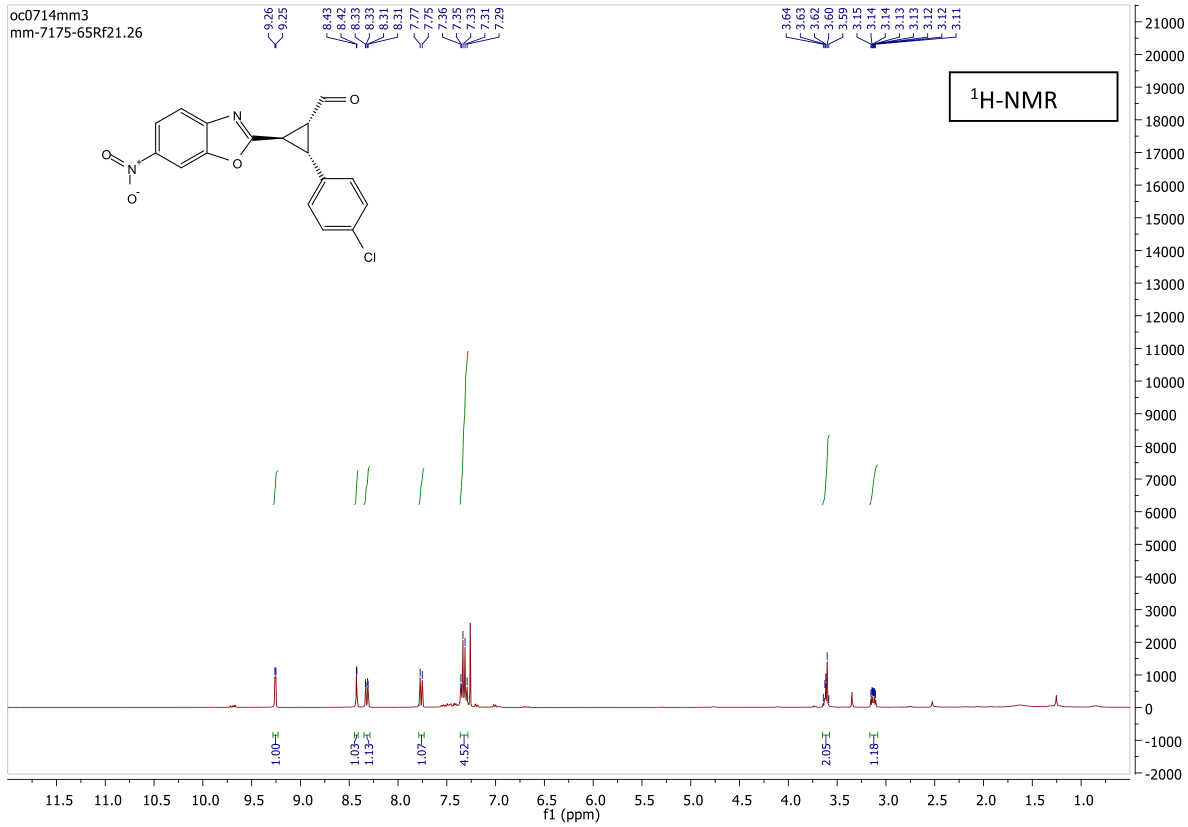

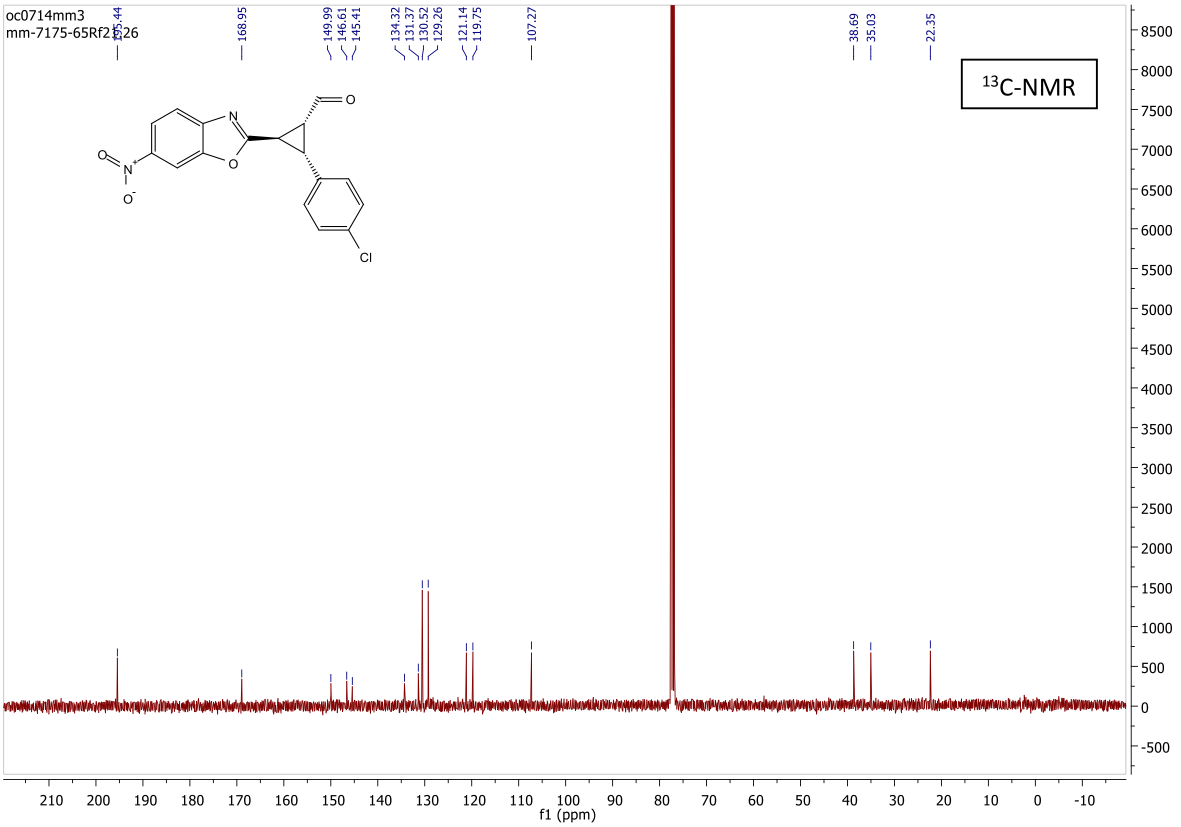
oc0714mm3
mm-7175-65Rf21.26
195.44
168.95
149.99
146.61
145.41
134.32
131.37
130.52
129.26
121.14
119.75
107.27
38.69
35.03
22.35
^{13}C-NMR
f1 (ppm)

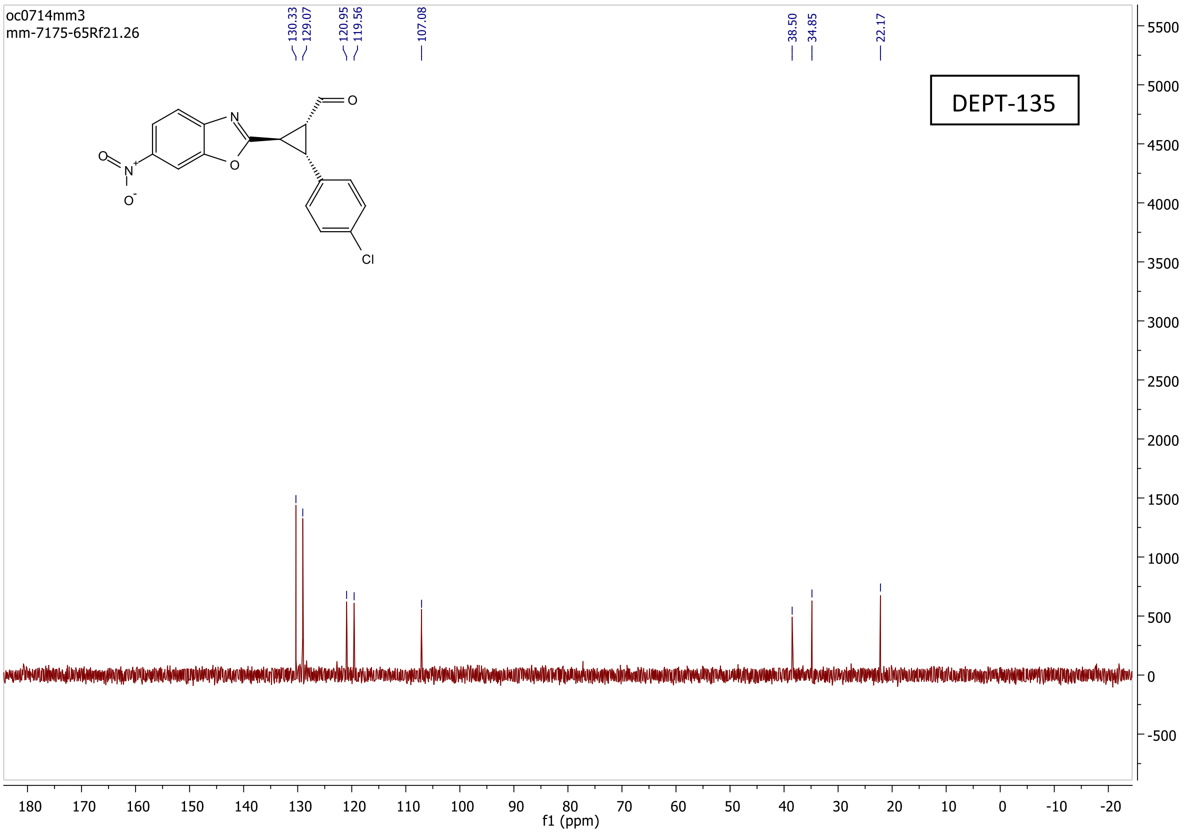
oc0714mm3
mm-7175-65Rf21.26
130.33
129.07
120.95
119.56
107.08
38.50
34.85
22.17
DEPT-135
f1 (ppm)

Product **4d** major diastereomer:

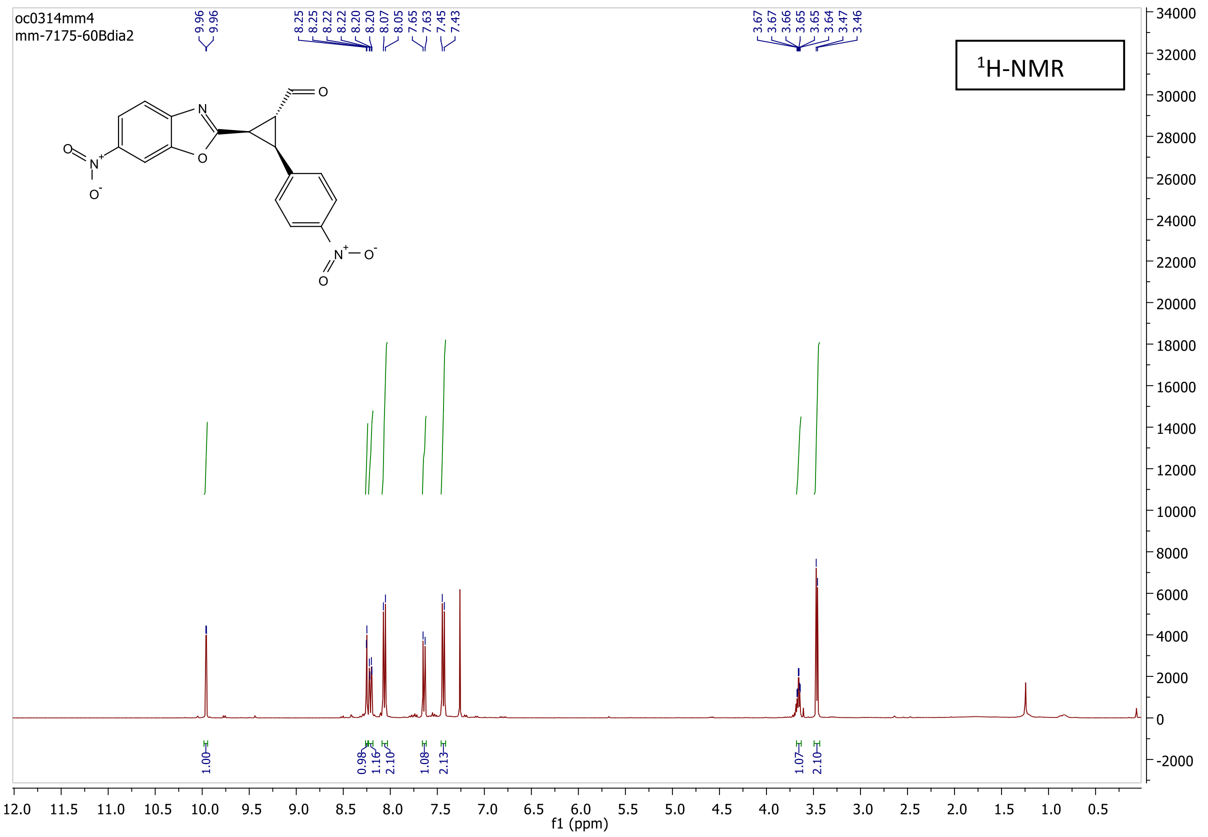

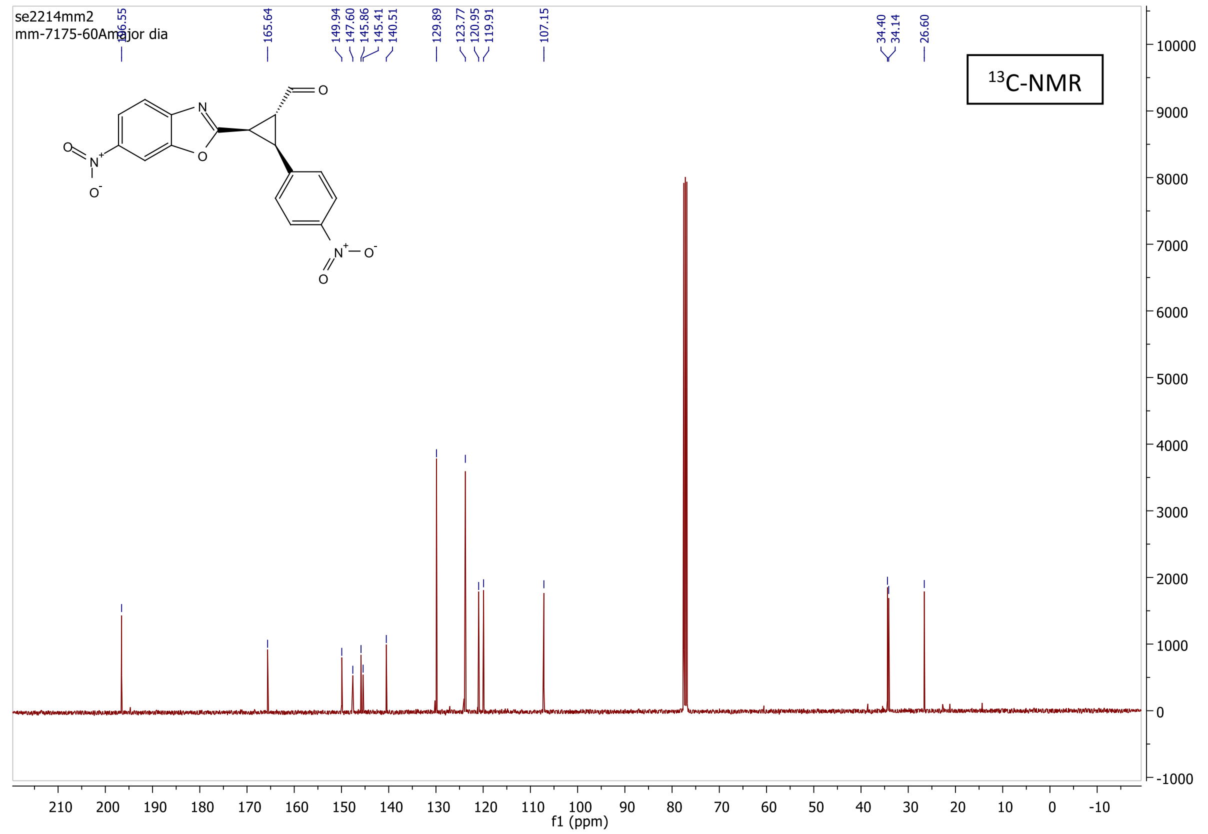

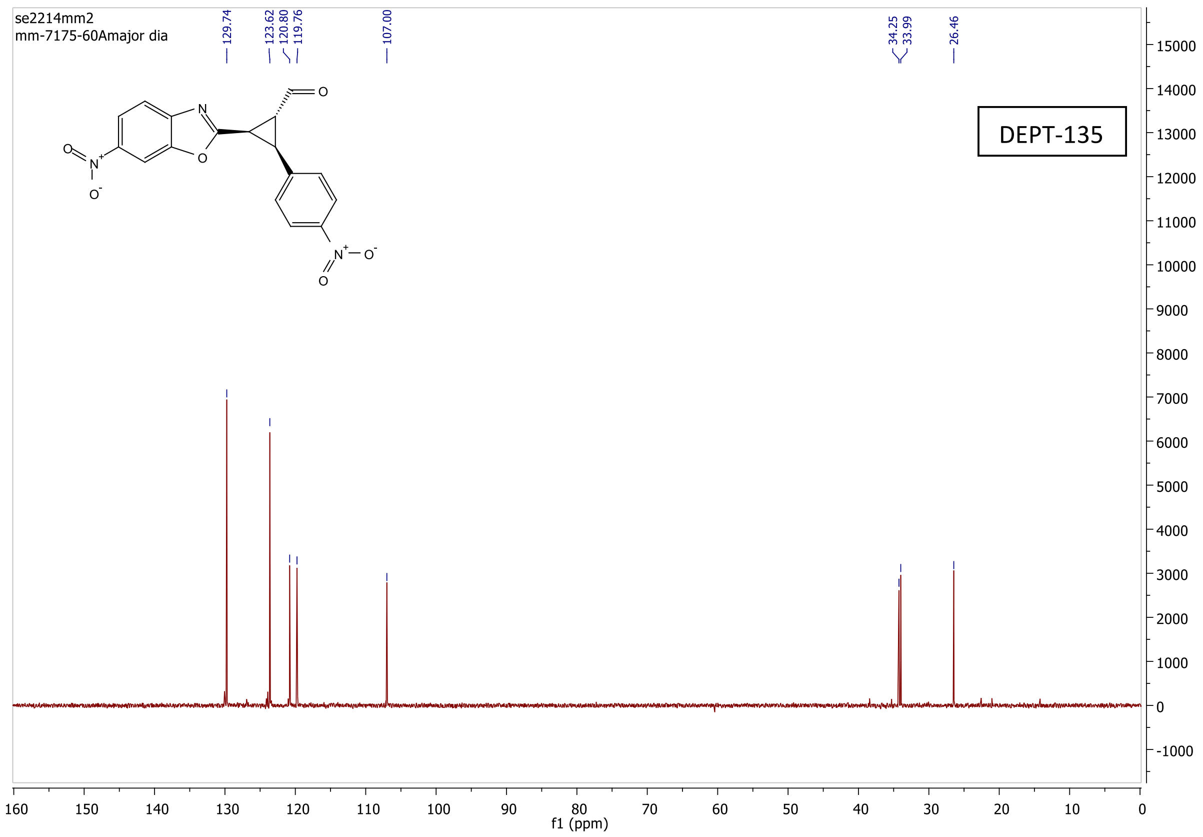

Product **4d** minor diastereomer:

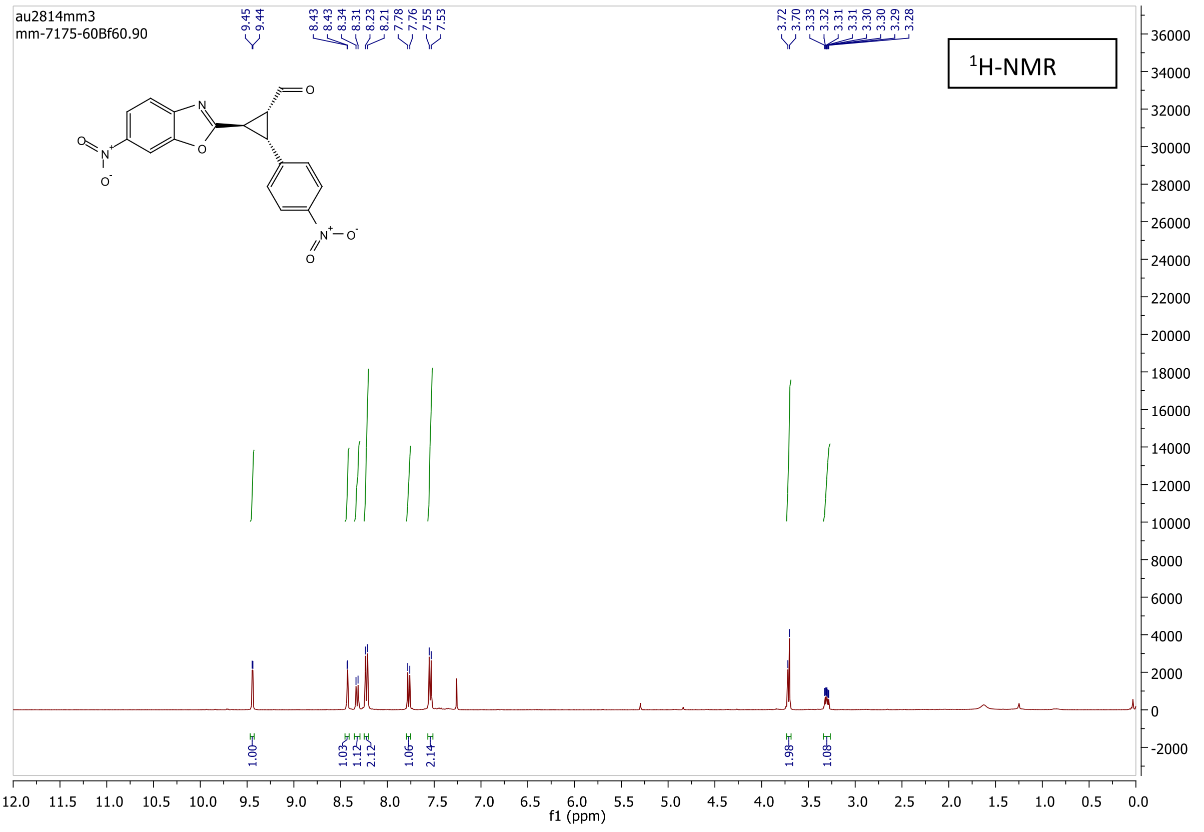

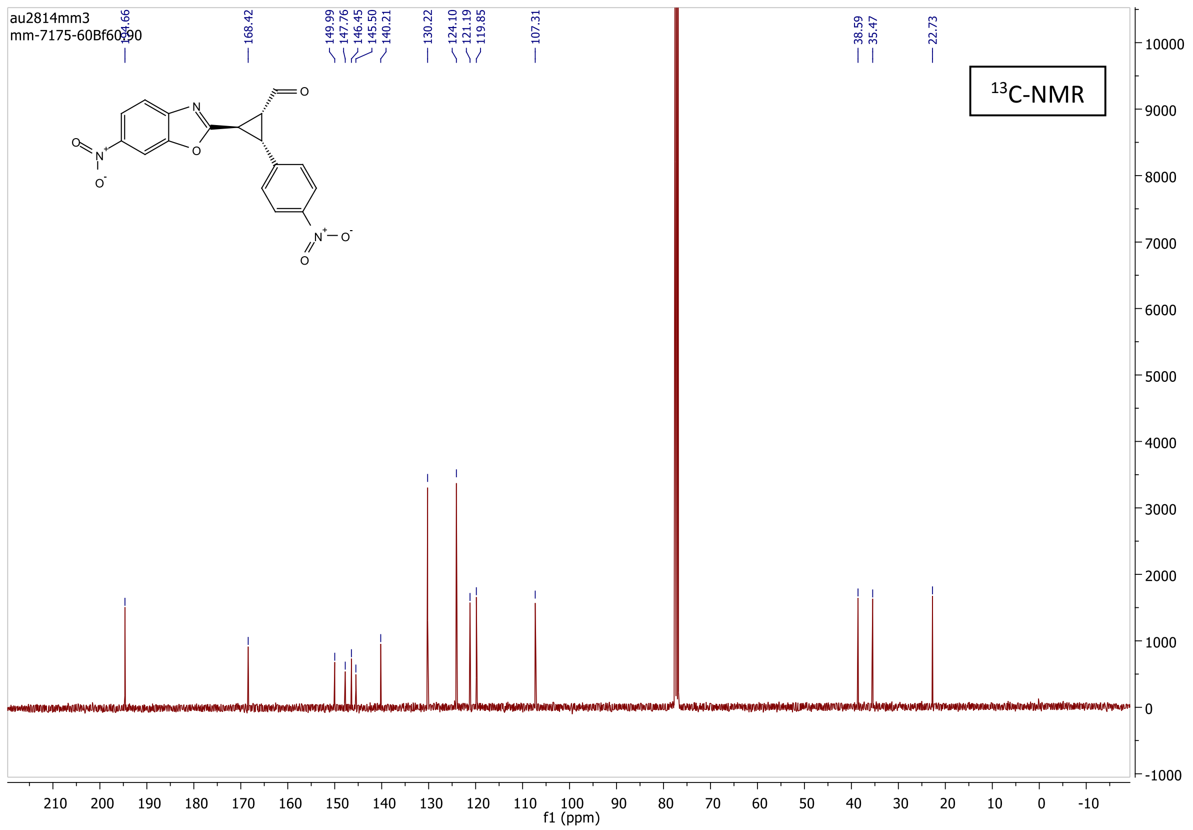
au2814mm3
mm-7175-60Bf60.90
194.66
168.42
149.99
147.76
146.45
145.50
140.21
130.22
124.10
121.19
119.85
107.31
38.59
35.47
22.73
13C-NMR
f1 (ppm)

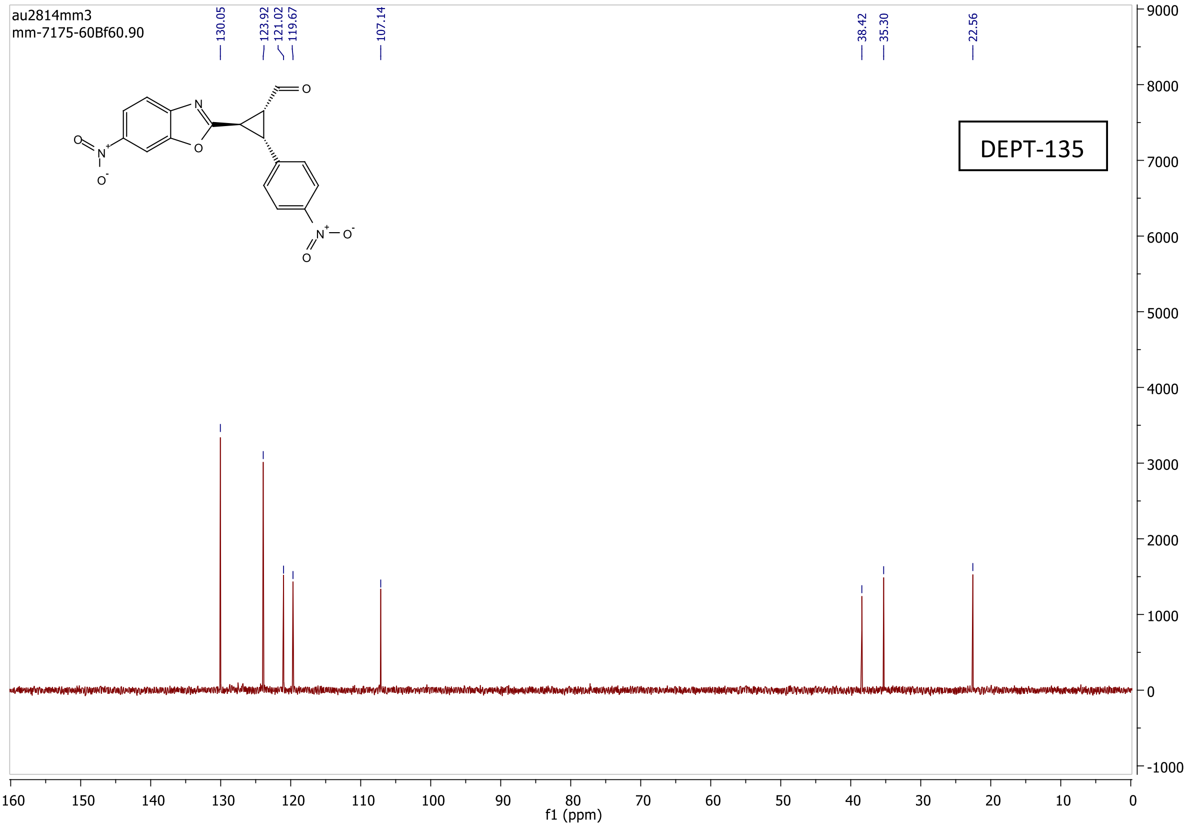
au2814mm3
mm-7175-60Bf60.90
130.05
123.92
121.02
119.67
107.14
38.42
35.30
22.56
DEPT-135
f1 (ppm)

Product **4f** major diastereomer:

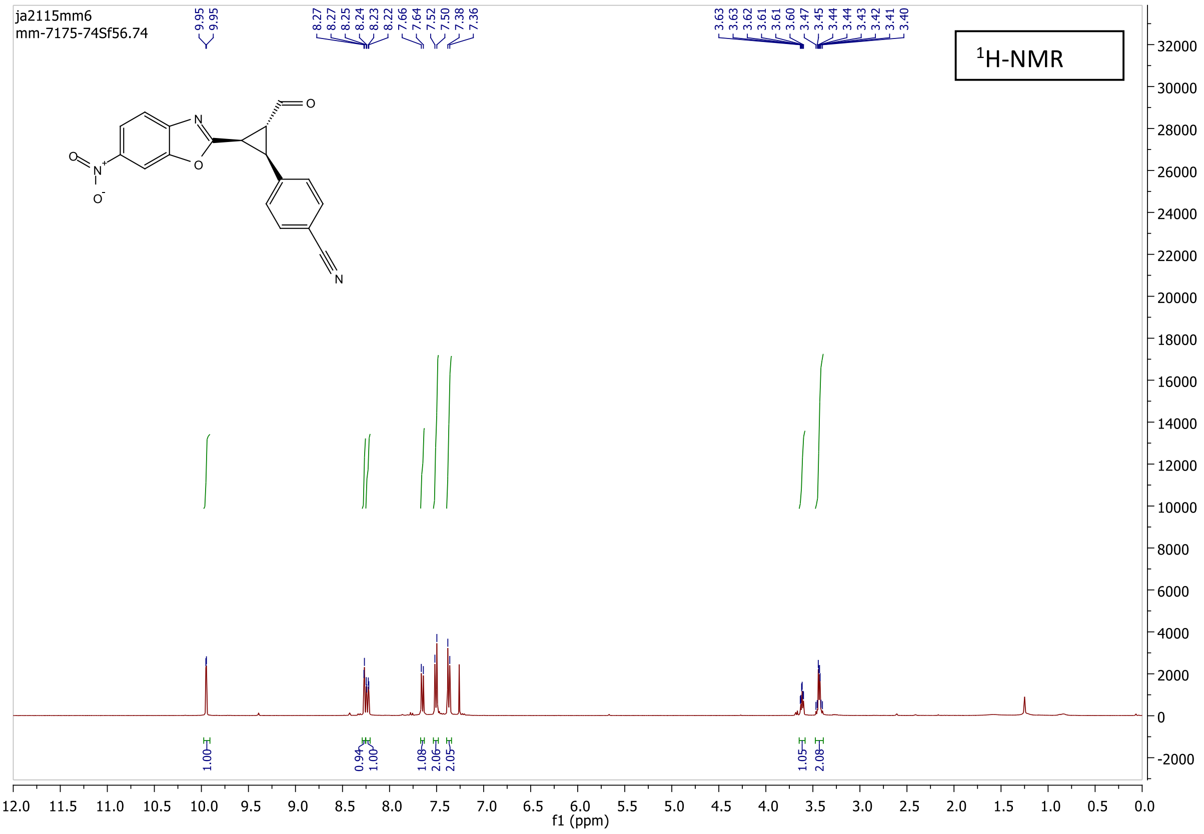

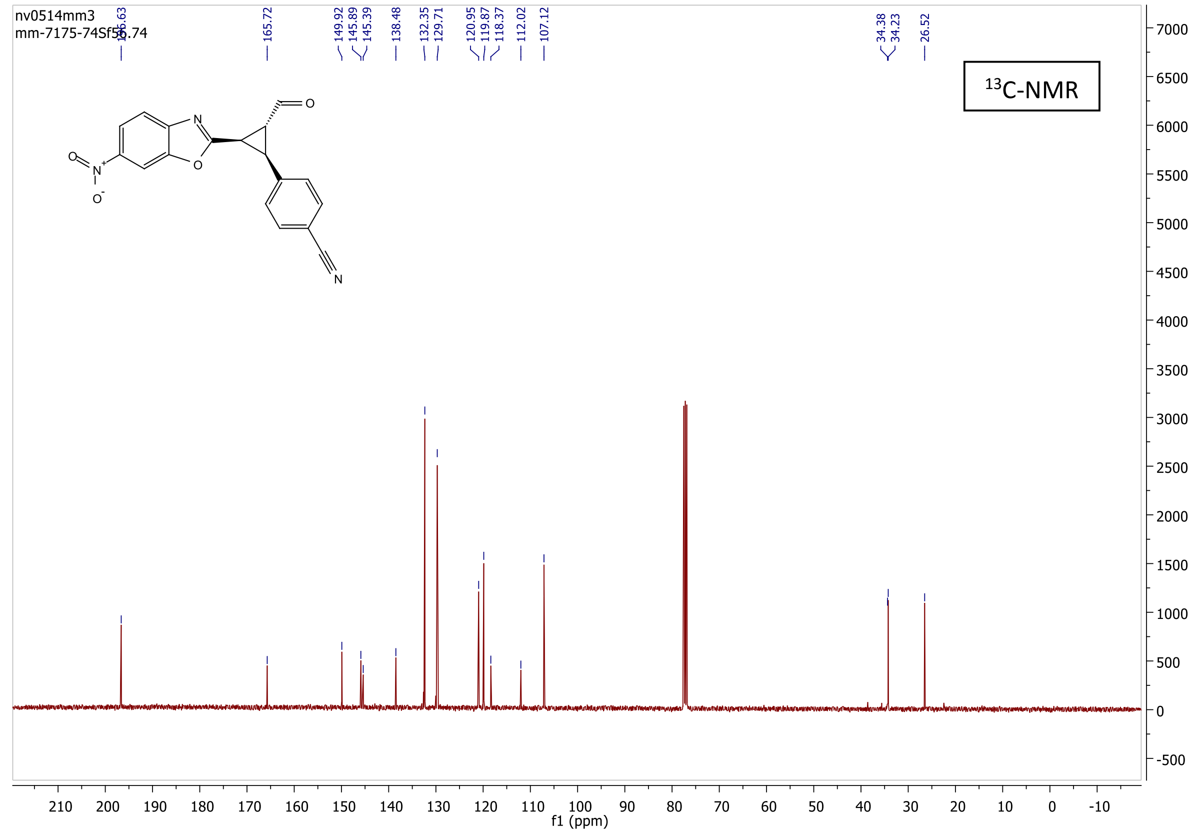

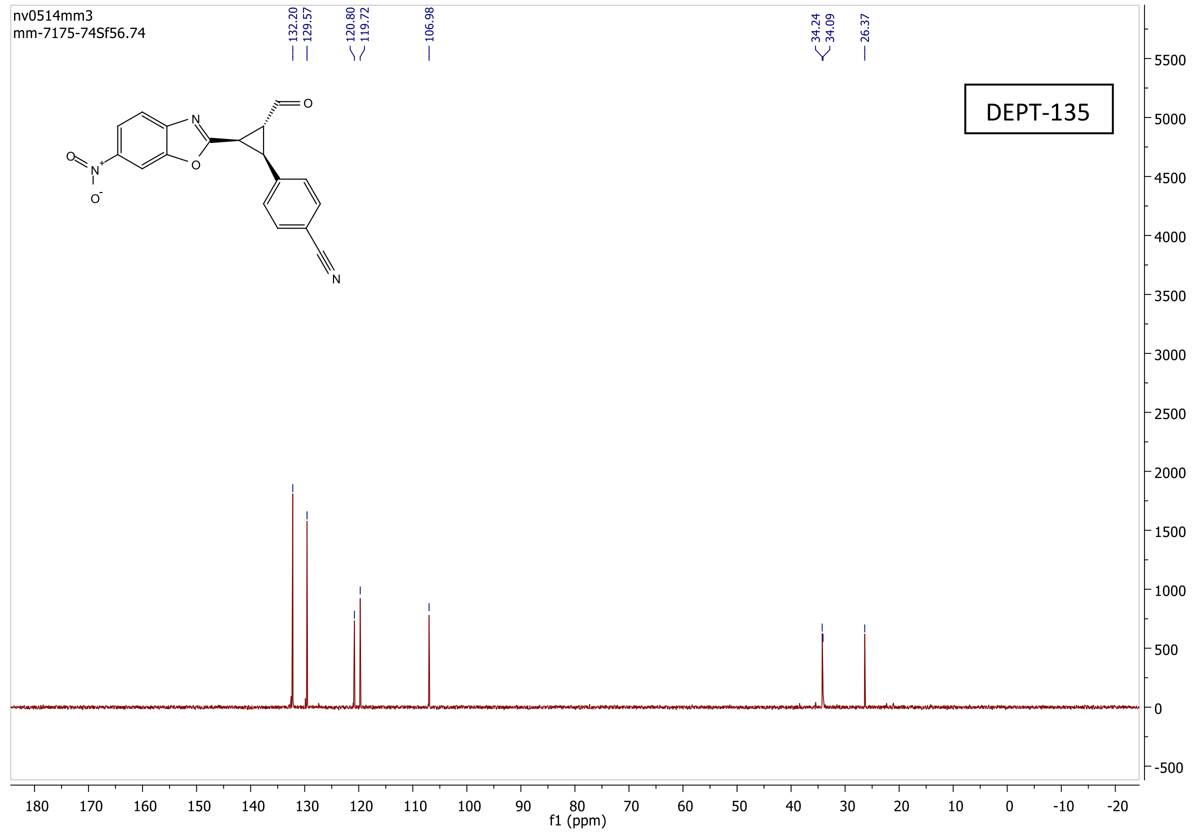

Product **4f** mixture of minor and minor’ diastereomers:

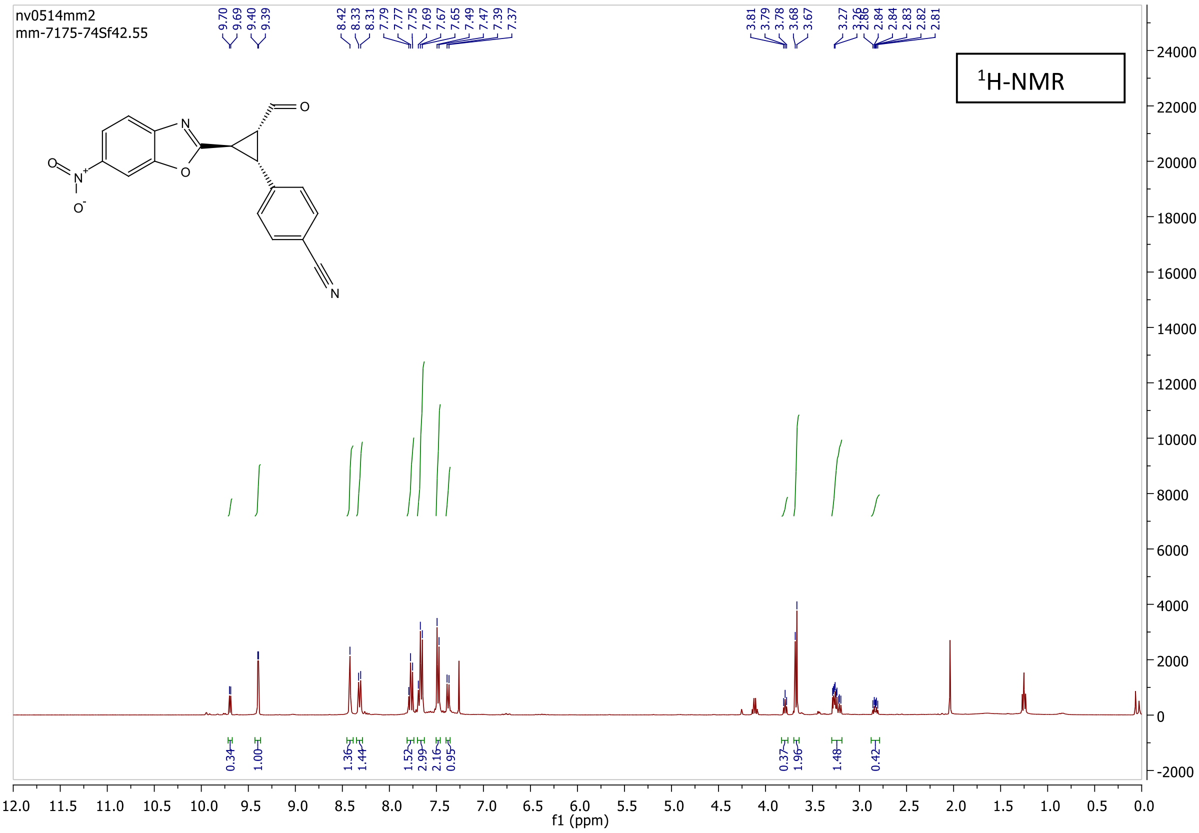

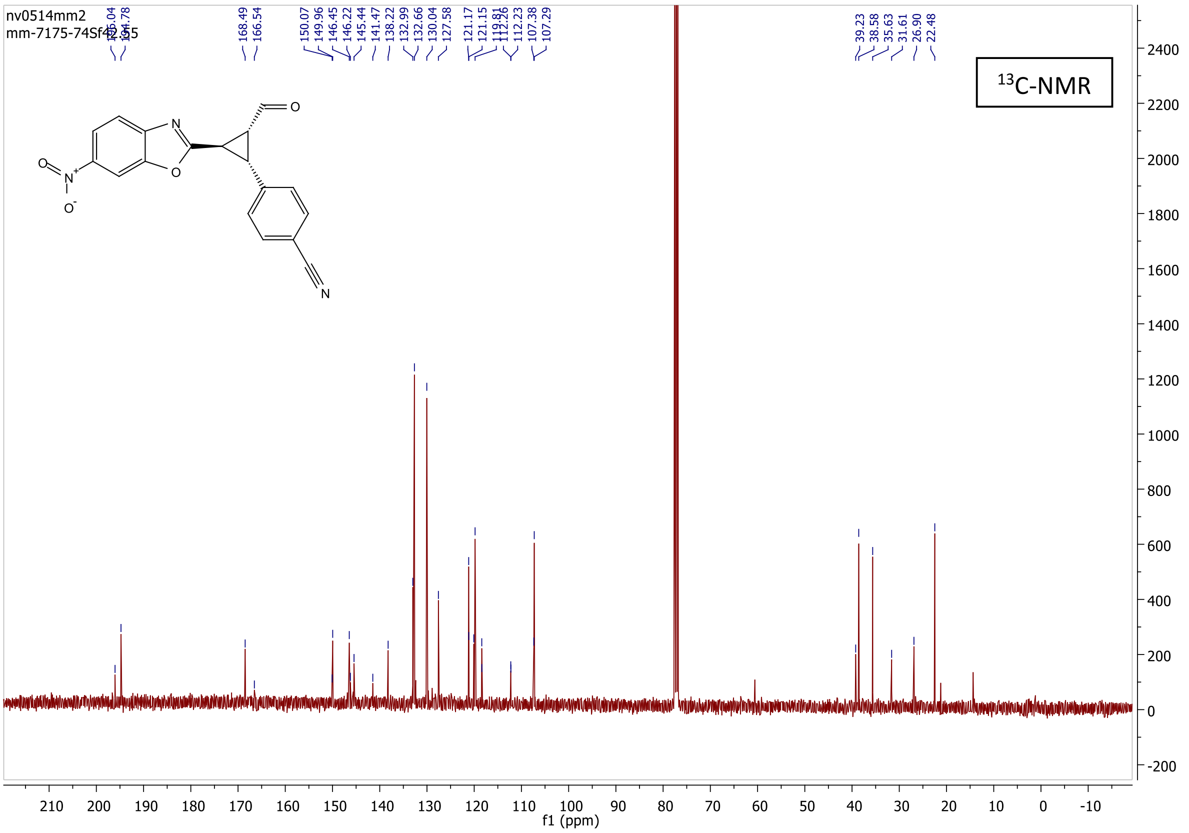

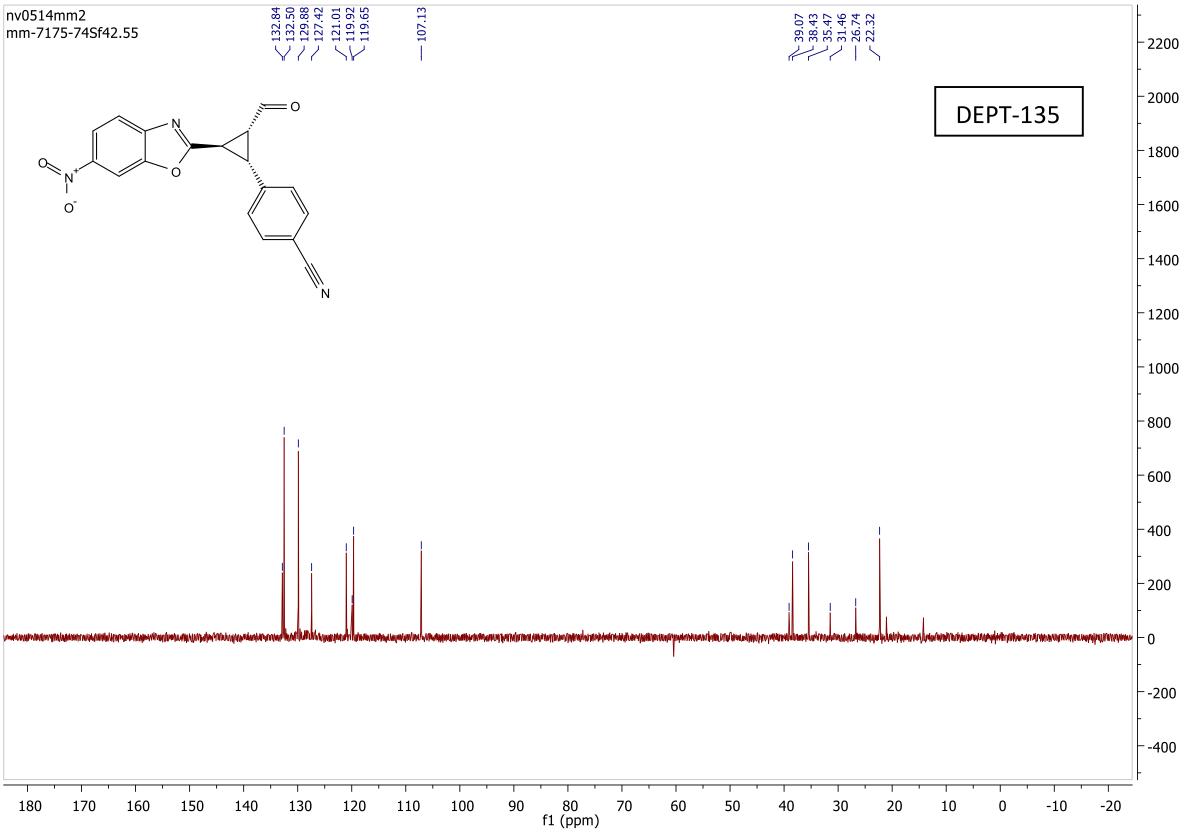

Product **4e** major diastereomer:

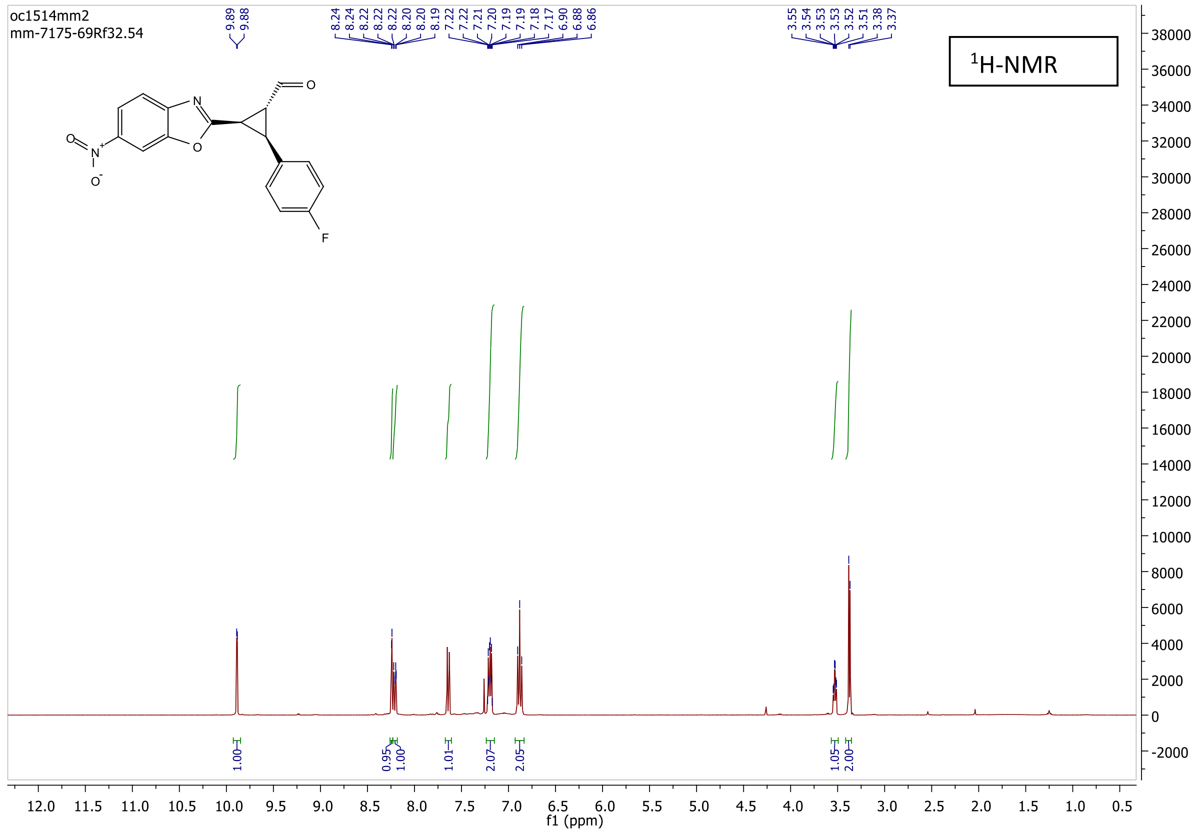

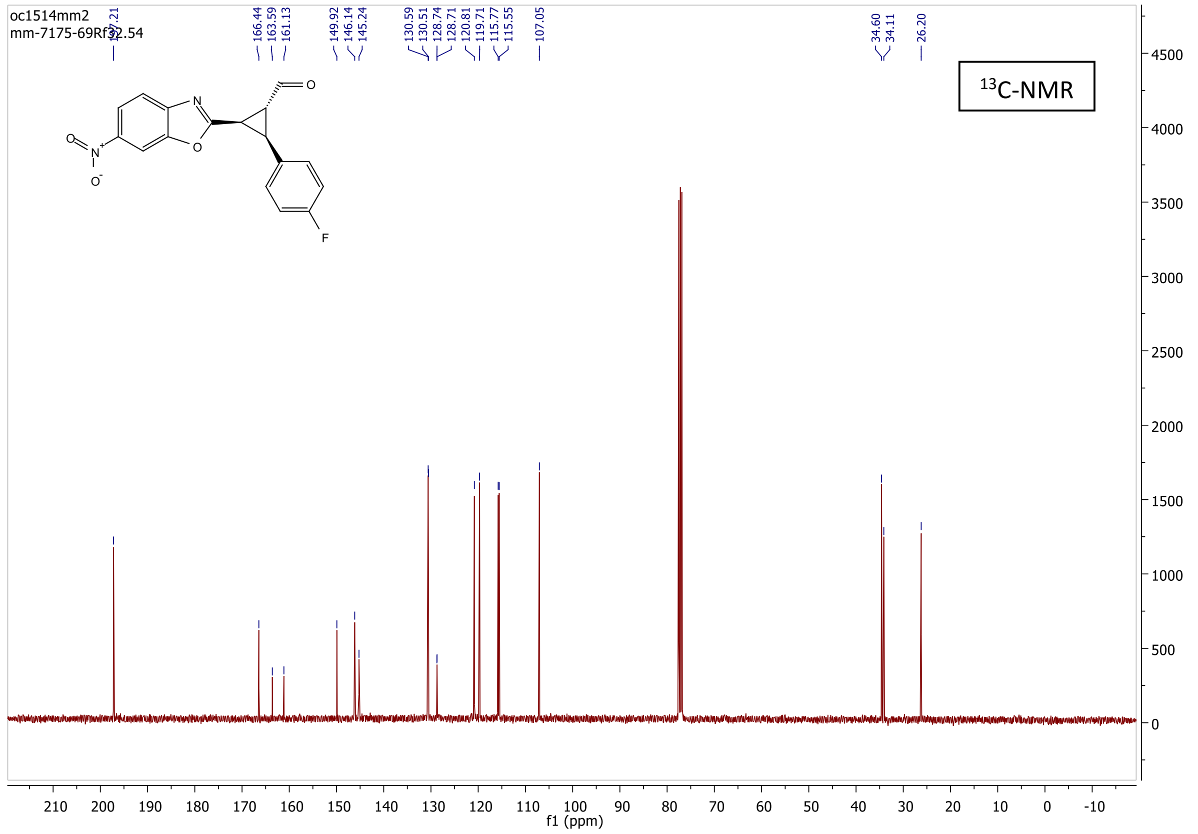

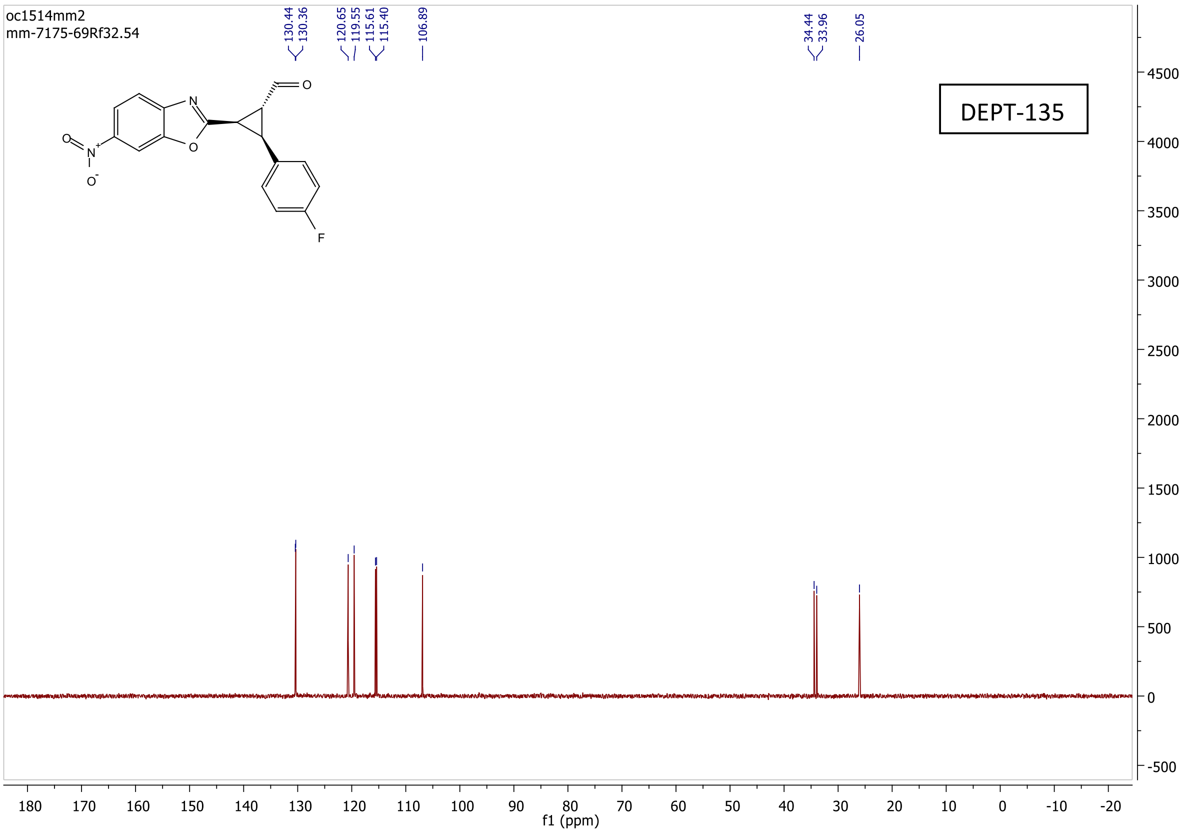
oc1514mm2
mm-7175-69Rf32.54
130.44
130.36
120.65
119.55
115.61
115.40
106.89
34.44
33.96
26.05
N
O
O
N
O
F
DEPT-135
f1 (ppm)

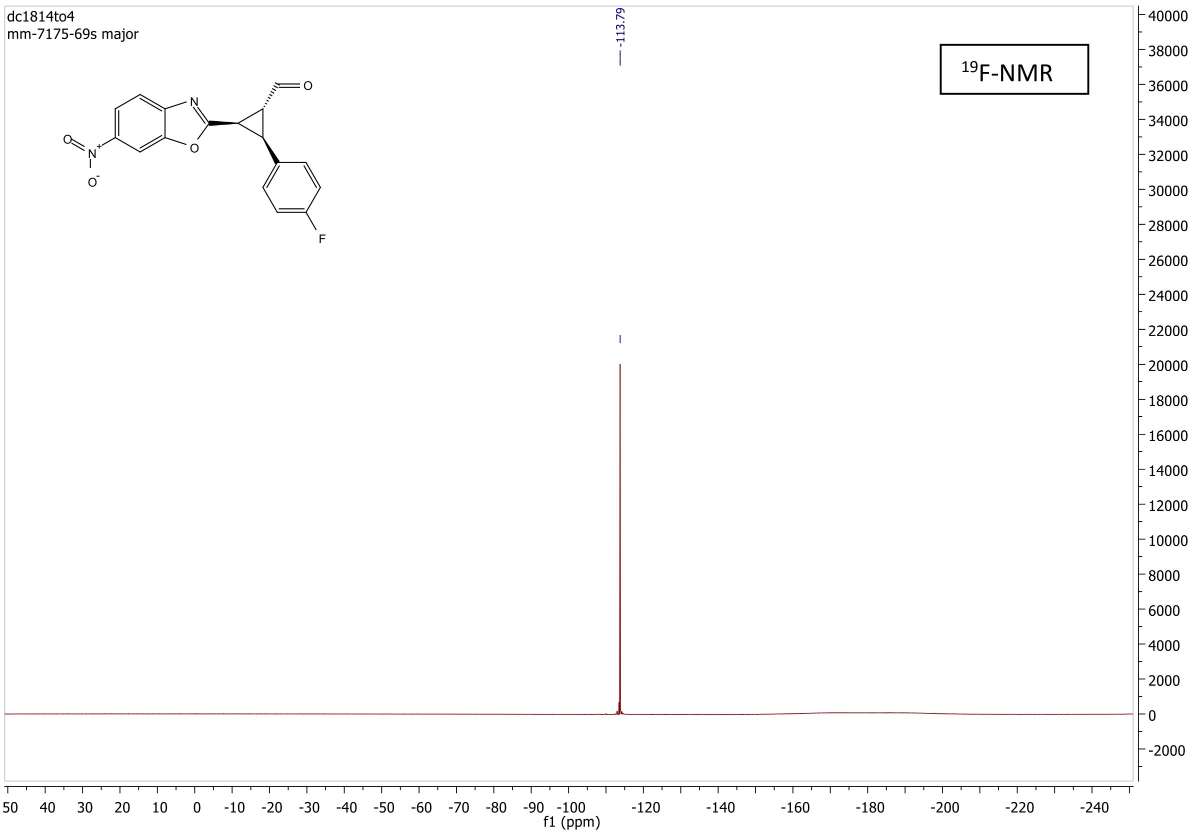
dc1814to4
mm-7175-69s major
-113.79
N
O
O
N
O
F
19F-NMR
f1 (ppm)

Product **4e** mixture of minor and minor' diastereomers:

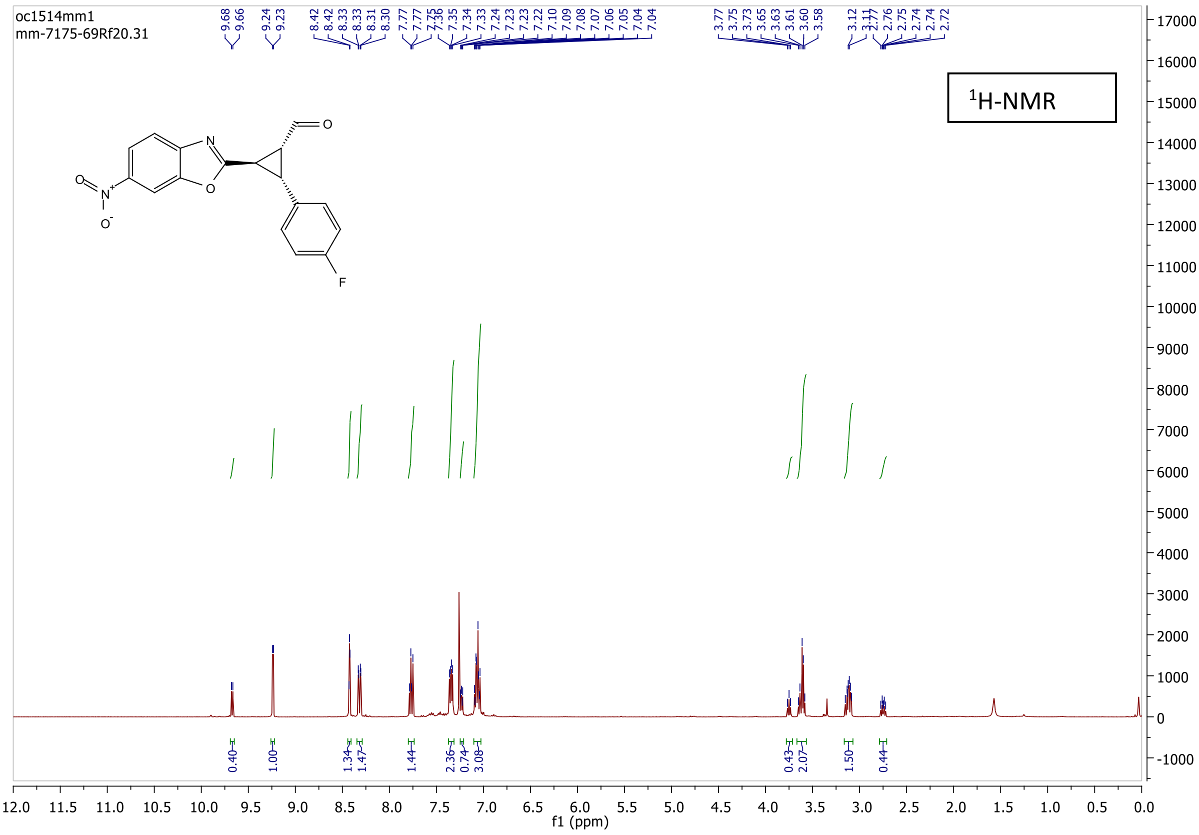

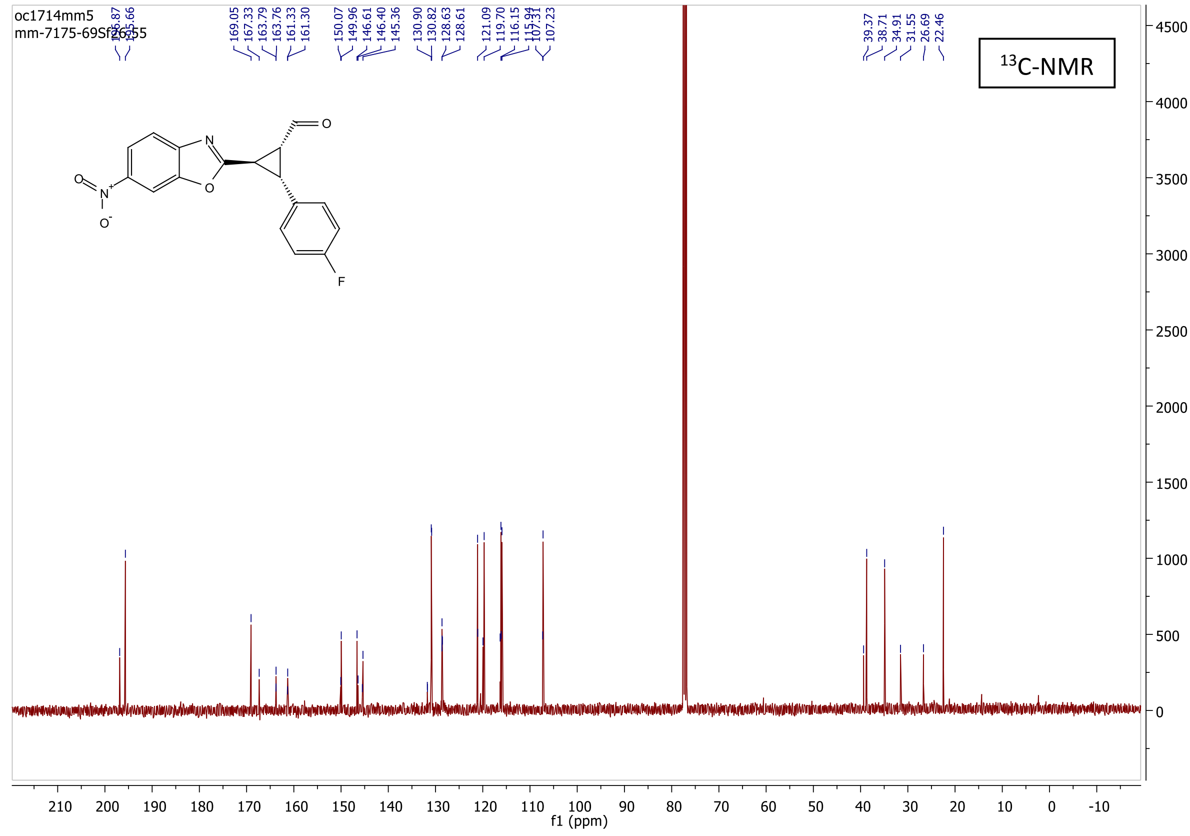

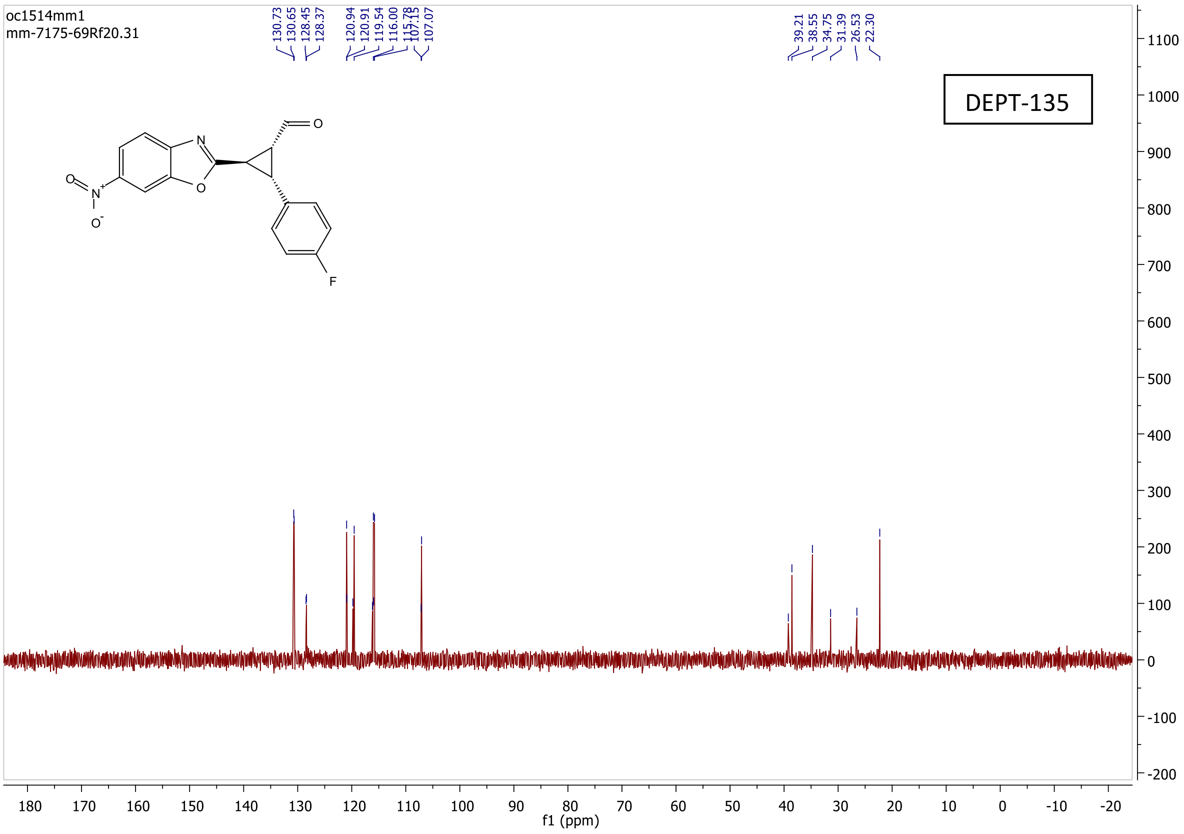

oc1514mm1
mm-7175-69Rf20.31
130.73
130.65
128.45
128.37
120.94
120.91
119.54
116.00
107.15
107.07
39.21
38.55
34.75
31.39
26.53
22.30
DEPT-135
f1 (ppm)

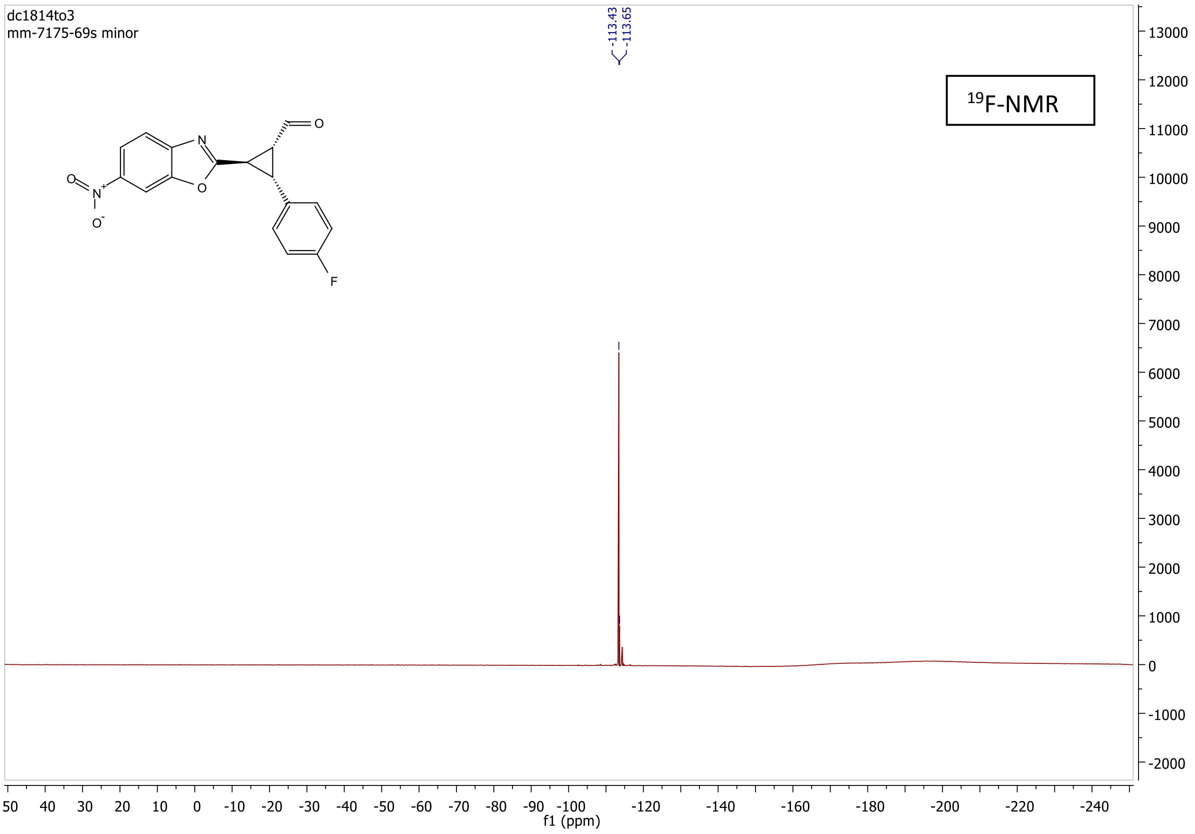

dc1814to3
mm-7175-69s minor
-113.43
-113.65
19F-NMR
f1 (ppm)

Product **4g** major diastereomer:

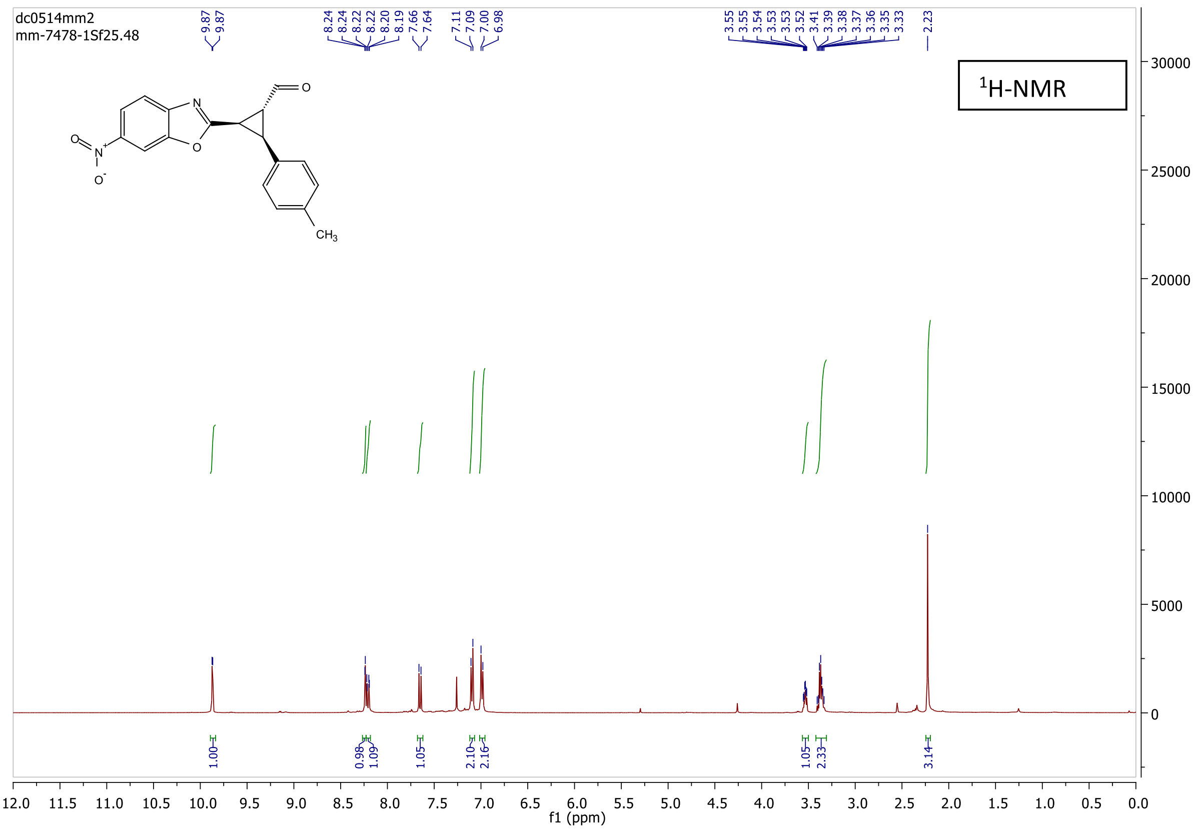

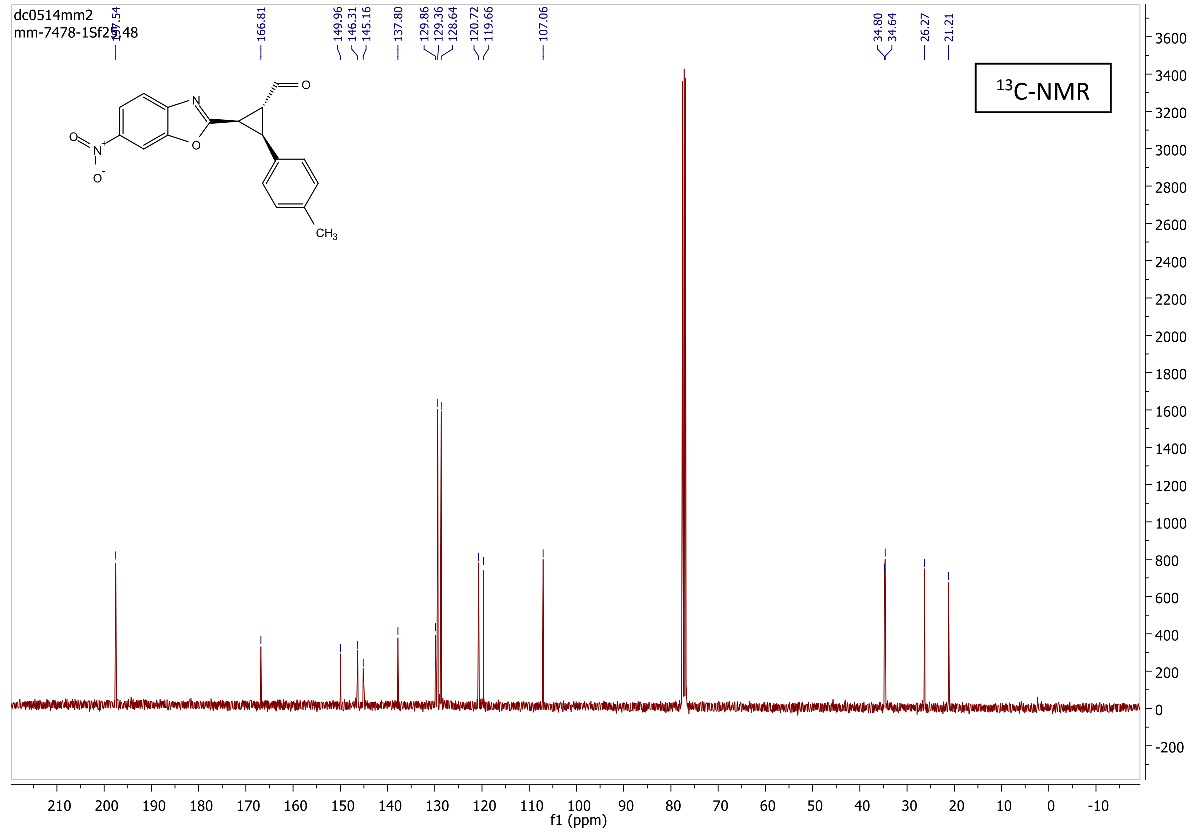

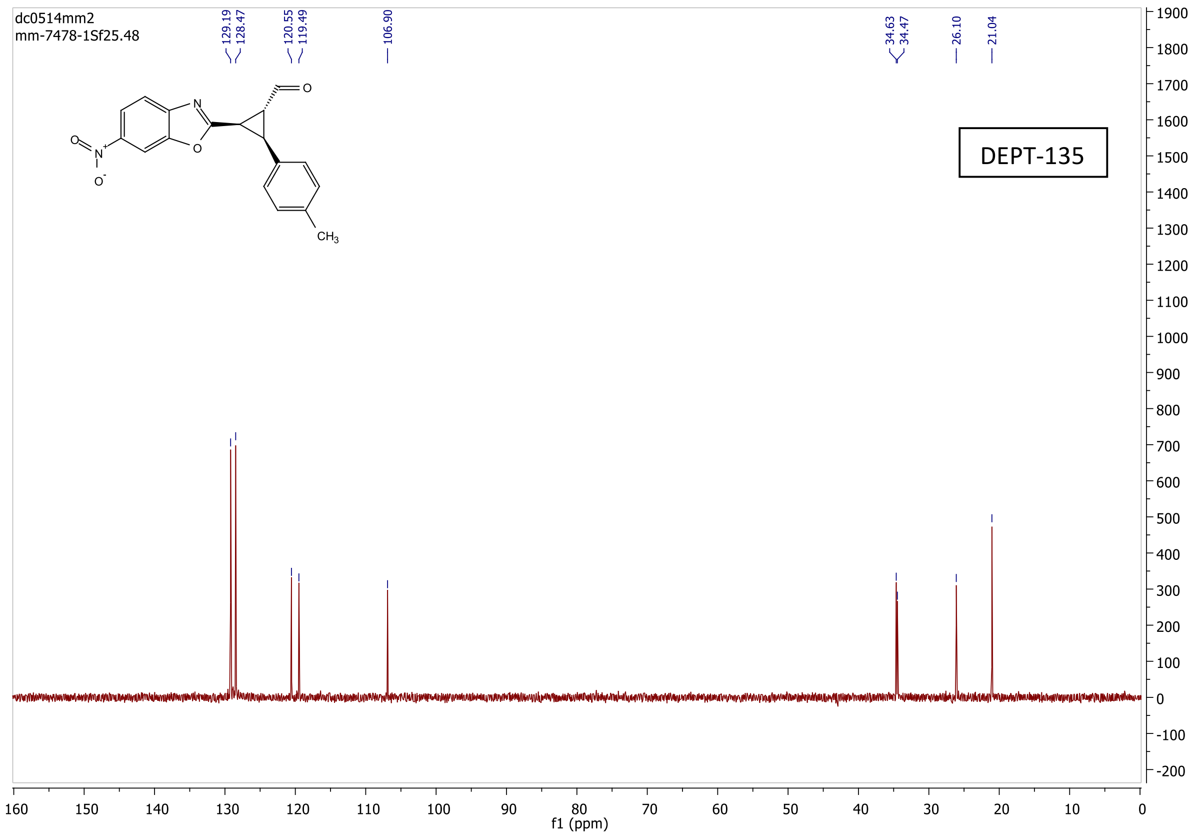

Product **4g** mixture of minor and minor' diastereomers:

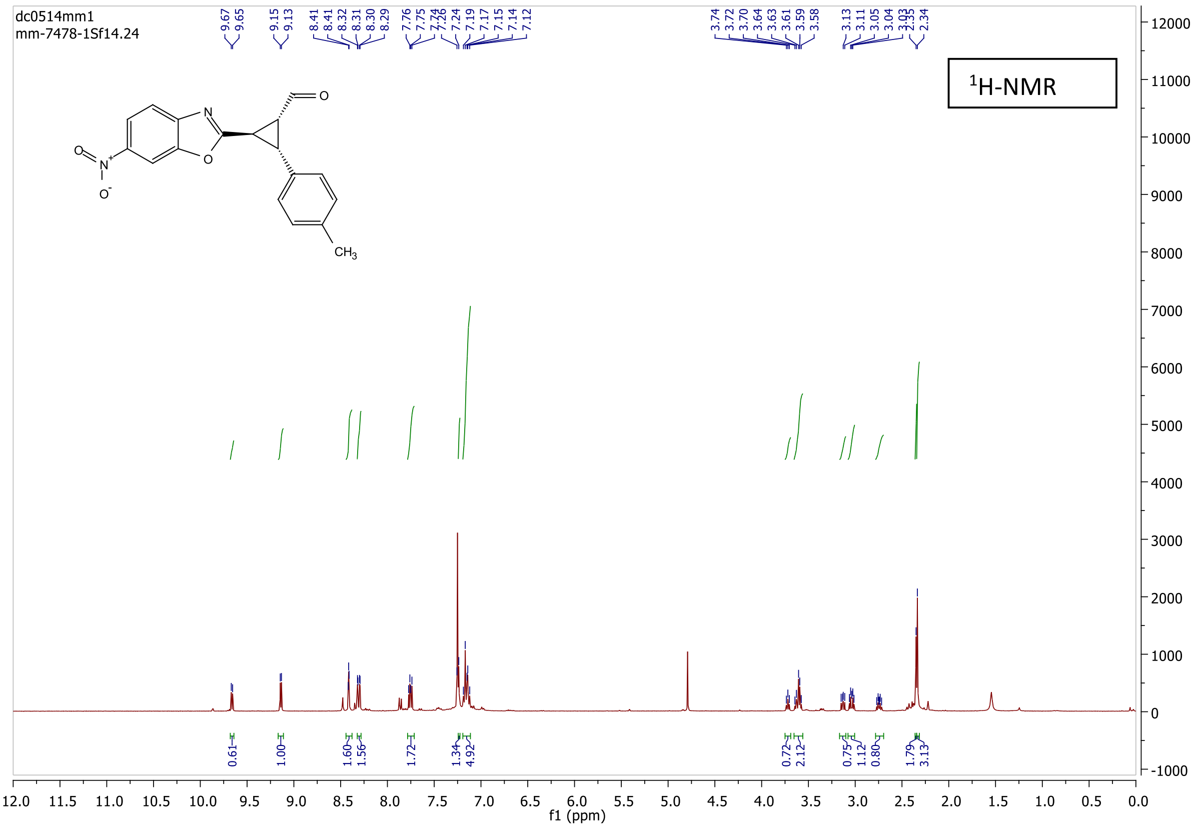

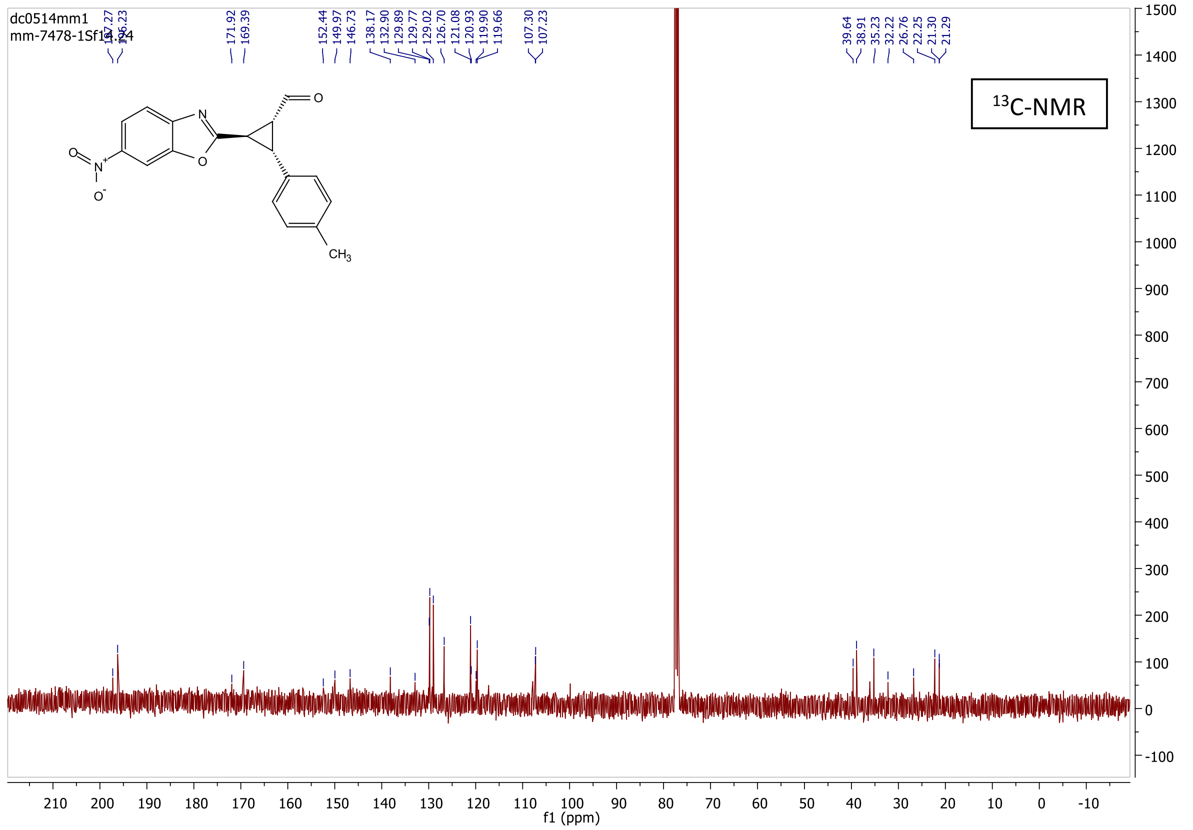

Product **4h** major diastereomer:

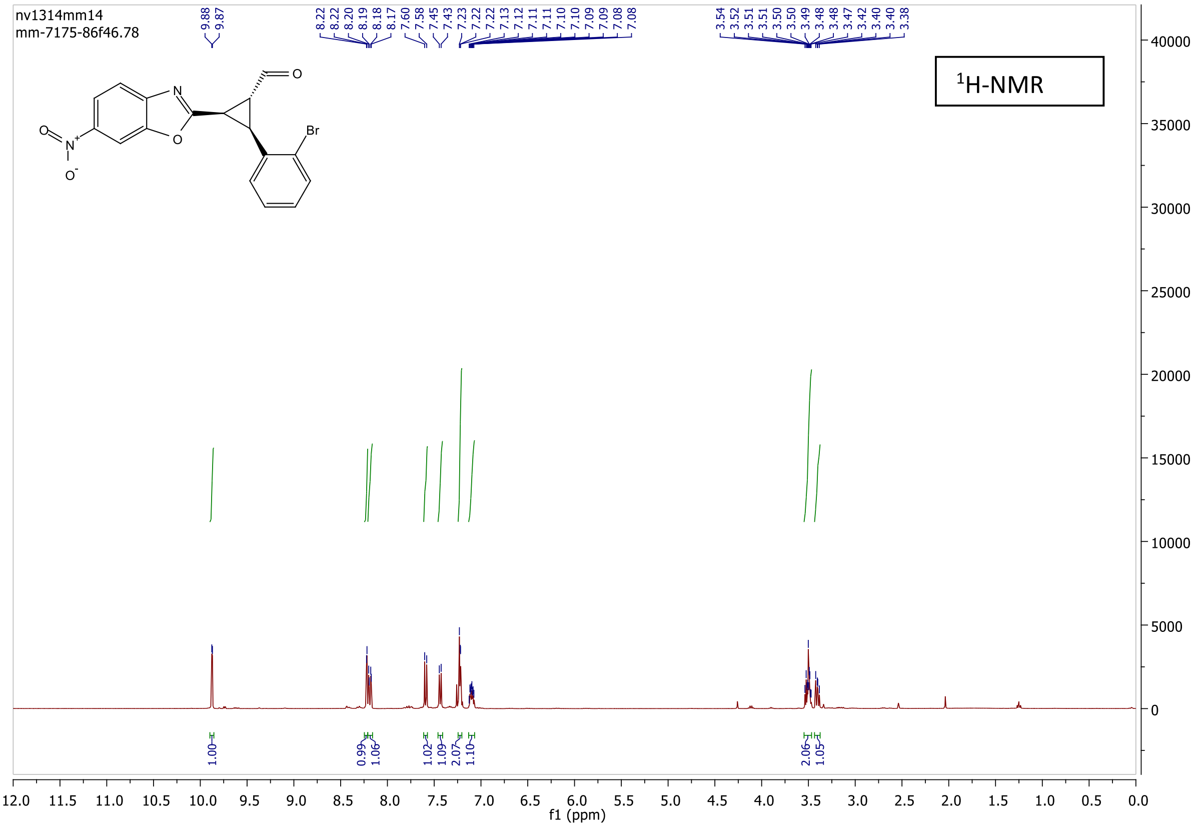

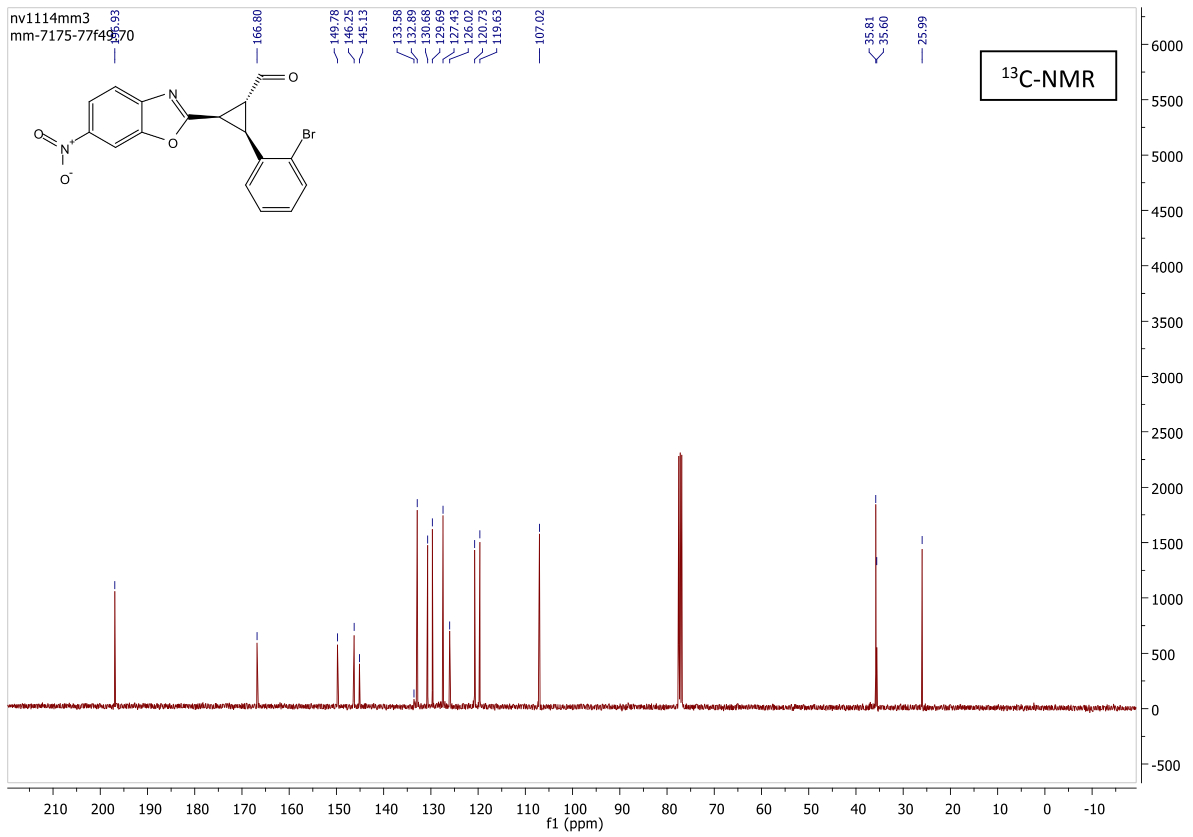
nv1114mm3
mm-7175-77f49.70
196.93
166.80
149.78
146.25
145.13
133.58
132.89
130.68
129.69
127.43
126.02
120.73
119.63
107.02
35.81
35.60
25.99
^{13}C-NMR
f1 (ppm)

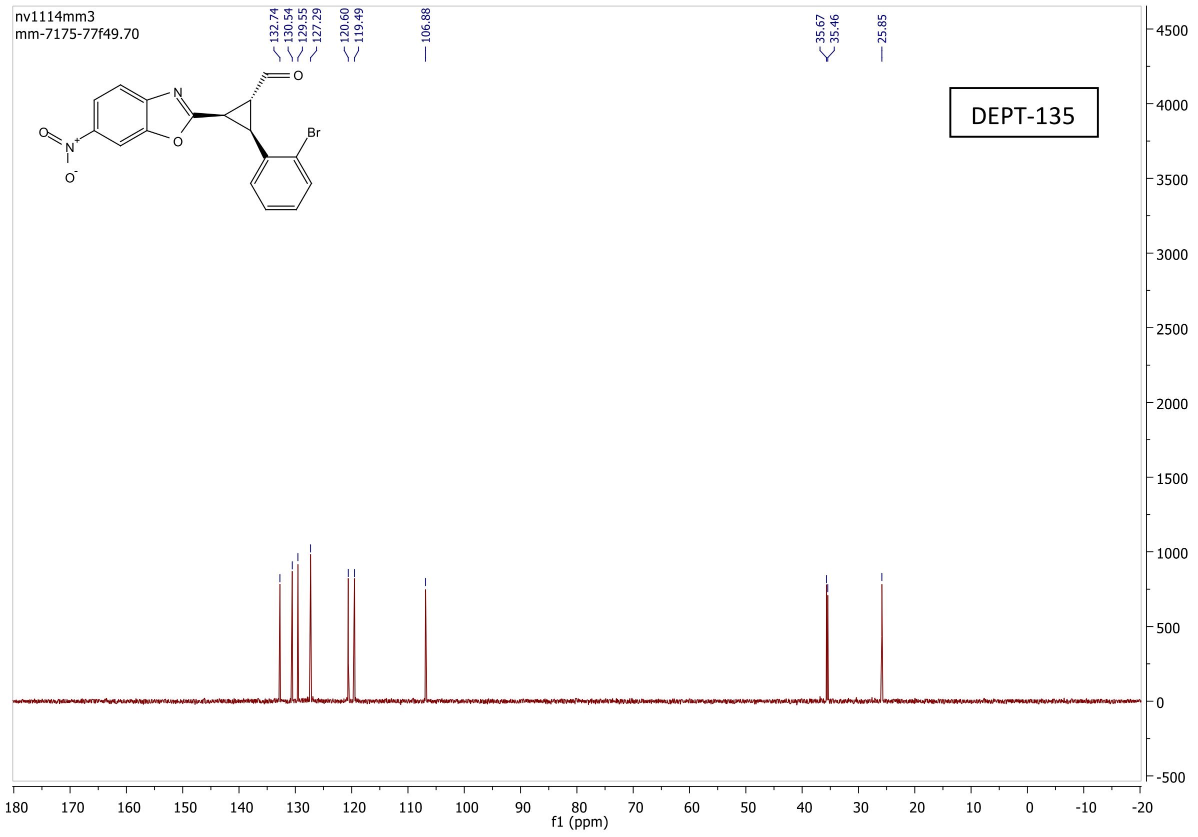
nv1114mm3
mm-7175-77f49.70
132.74
130.54
129.55
127.29
120.60
119.49
106.88
35.67
35.46
25.85
DEPT-135
f1 (ppm)

Product **4h** minor diastereomer + traces of minor’ and major diastereomers and starting enals

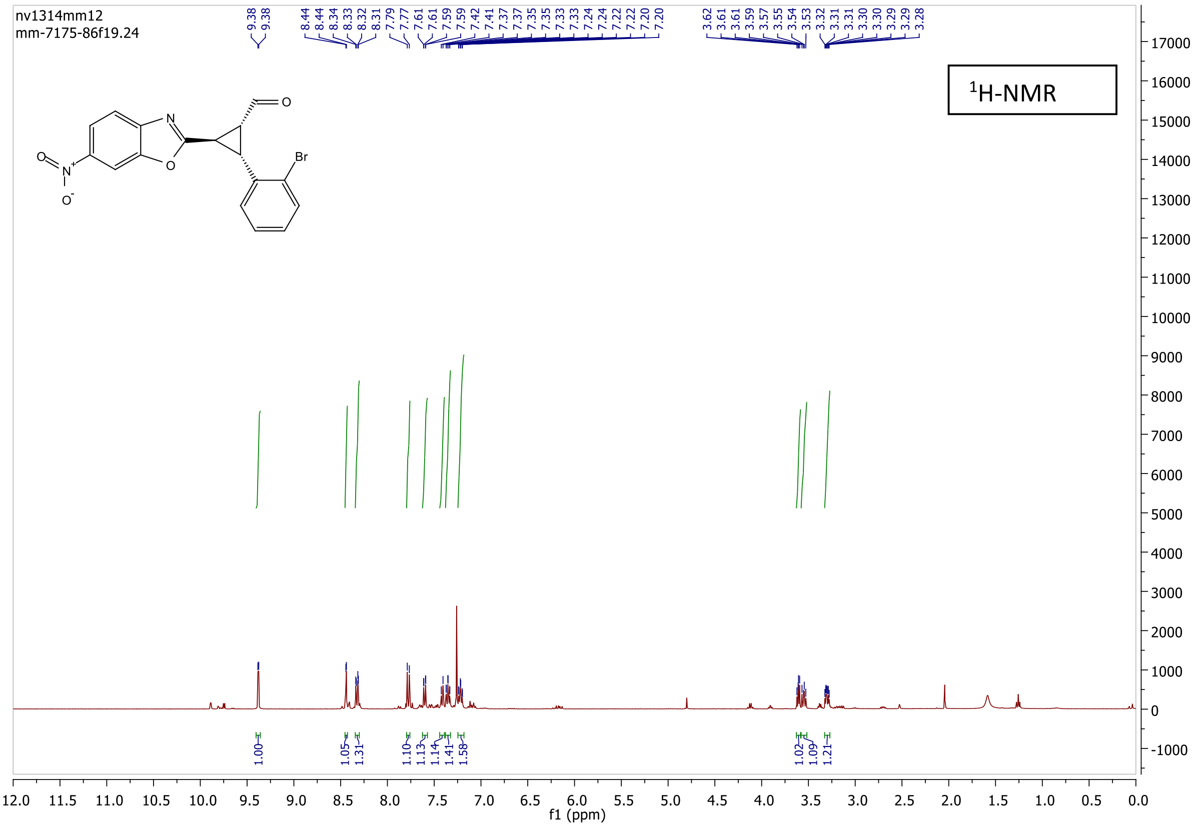

Product **4i**:

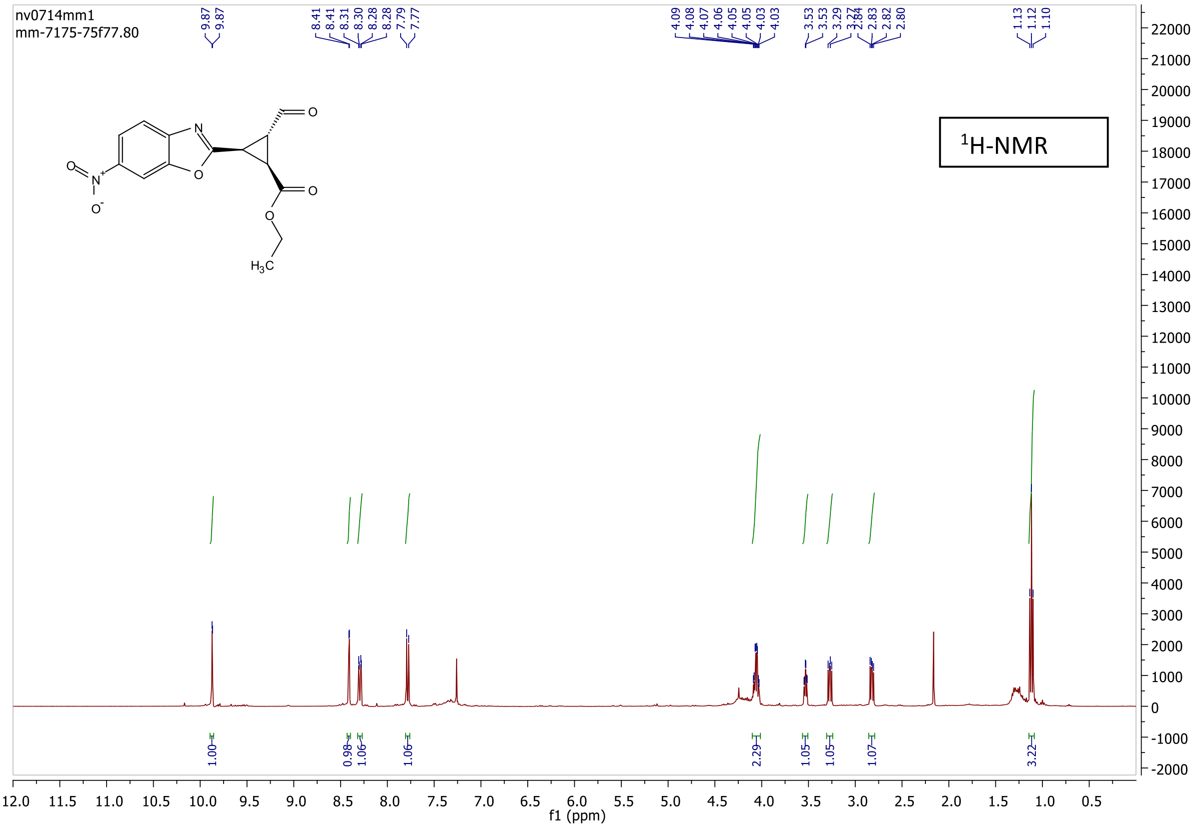

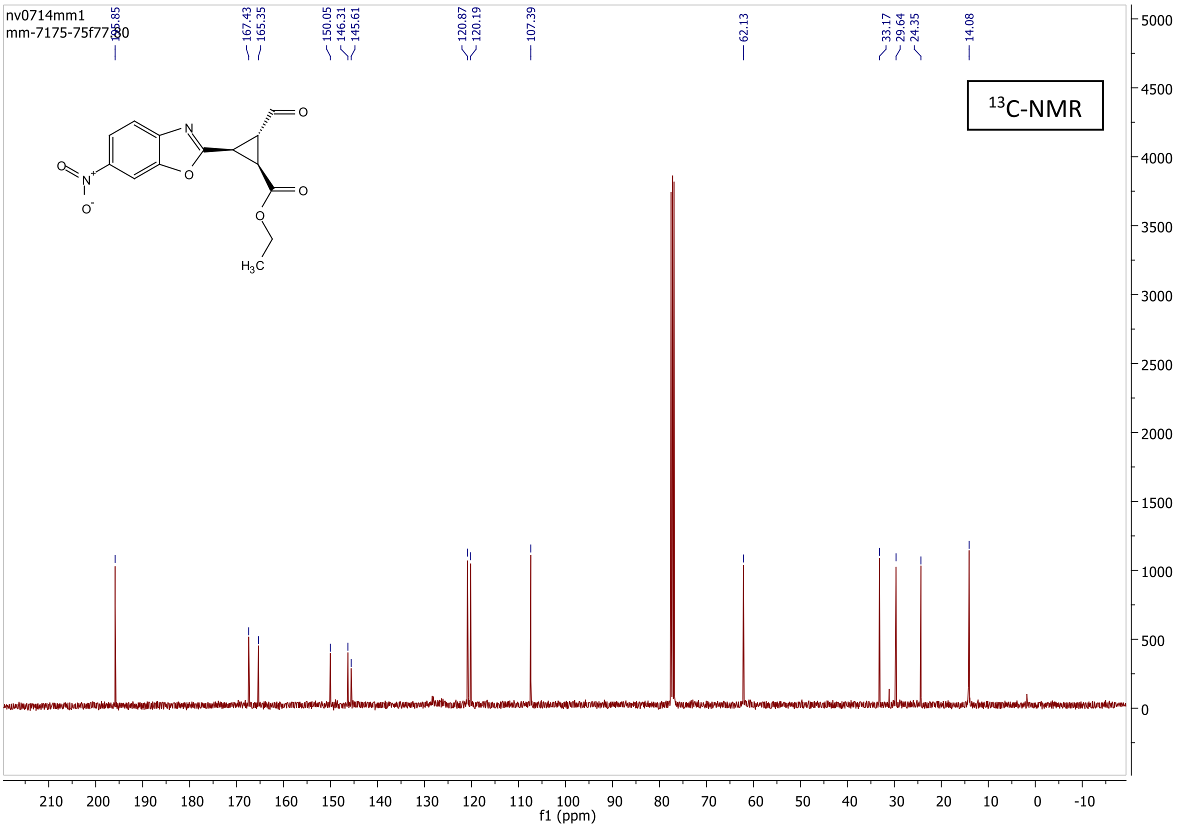
nv0714mm1
mm-7175-75f77.80
196.85
167.43
165.35
150.05
146.31
145.61
120.87
120.19
107.39
62.13
33.17
29.64
24.35
14.08
13C-NMR
f1 (ppm)

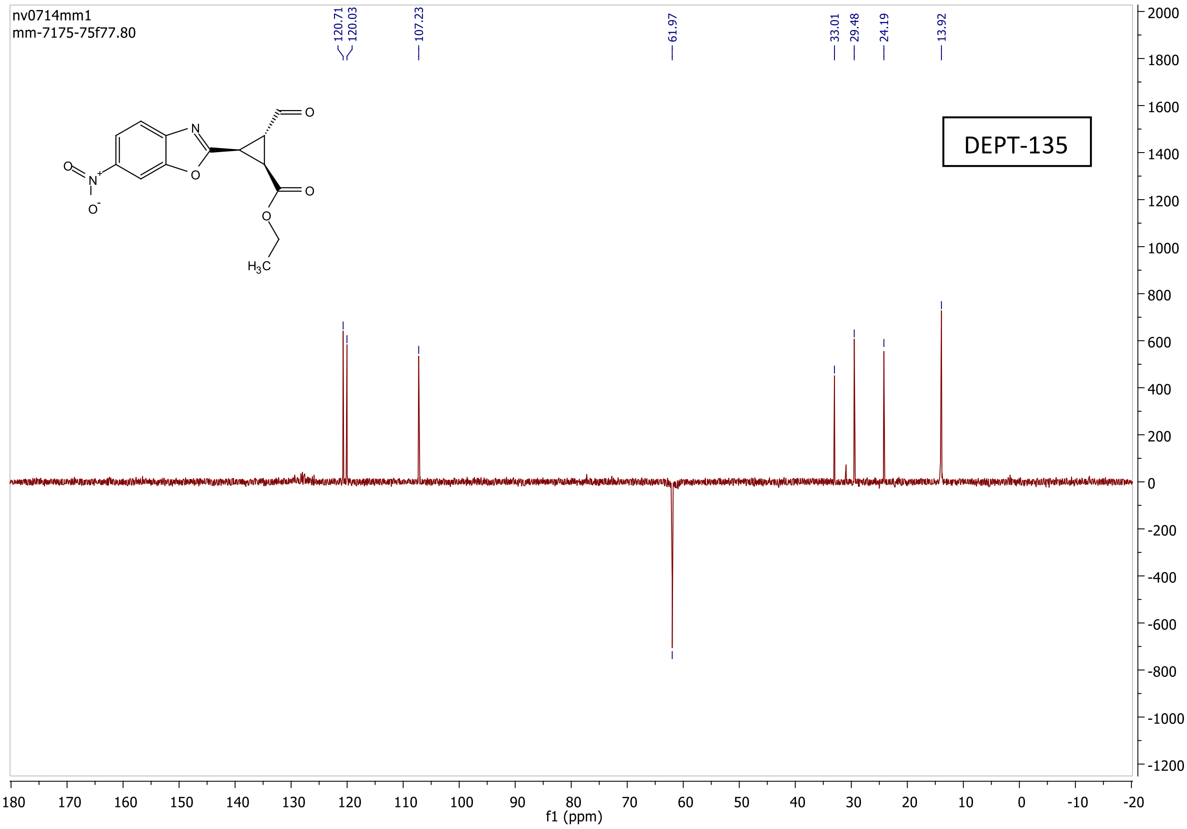
nv0714mm1
mm-7175-75f77.80
120.71
120.03
107.23
61.97
33.01
29.48
24.19
13.92
DEPT-135
f1 (ppm)

Product **5a** major diastereomer:

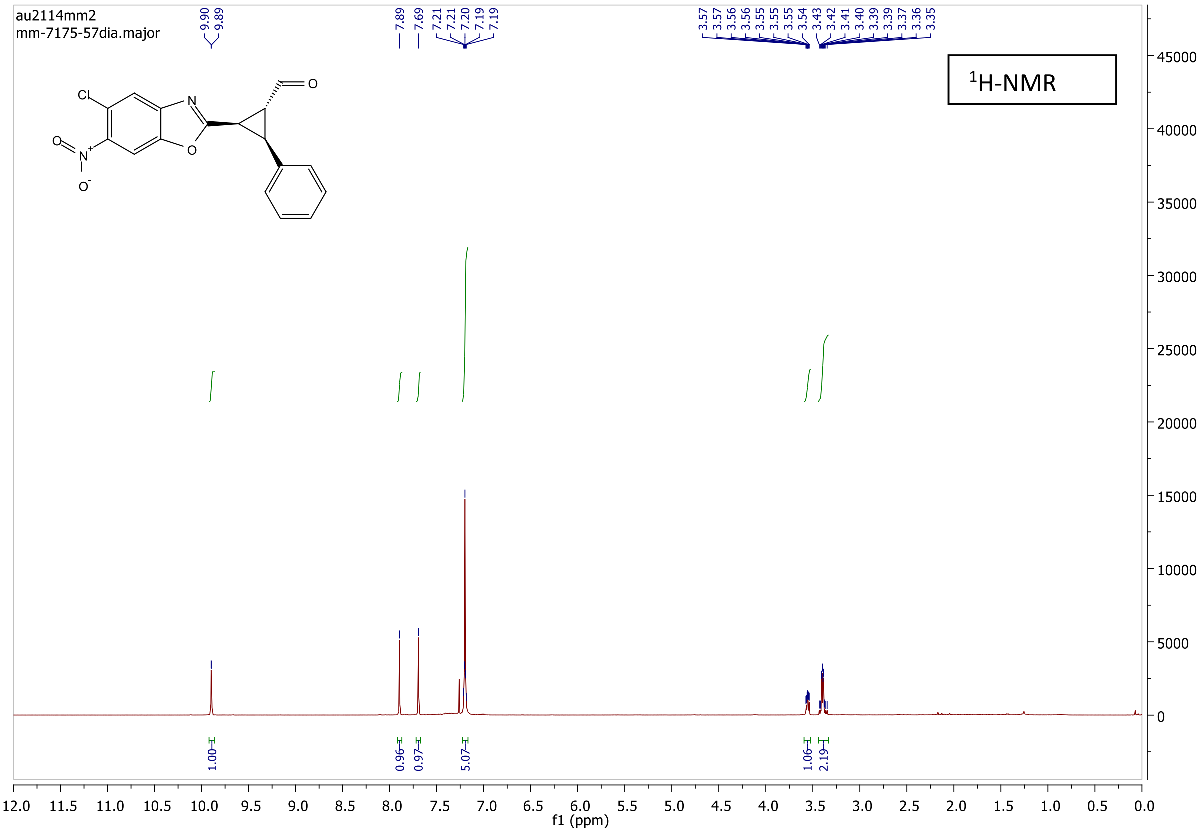

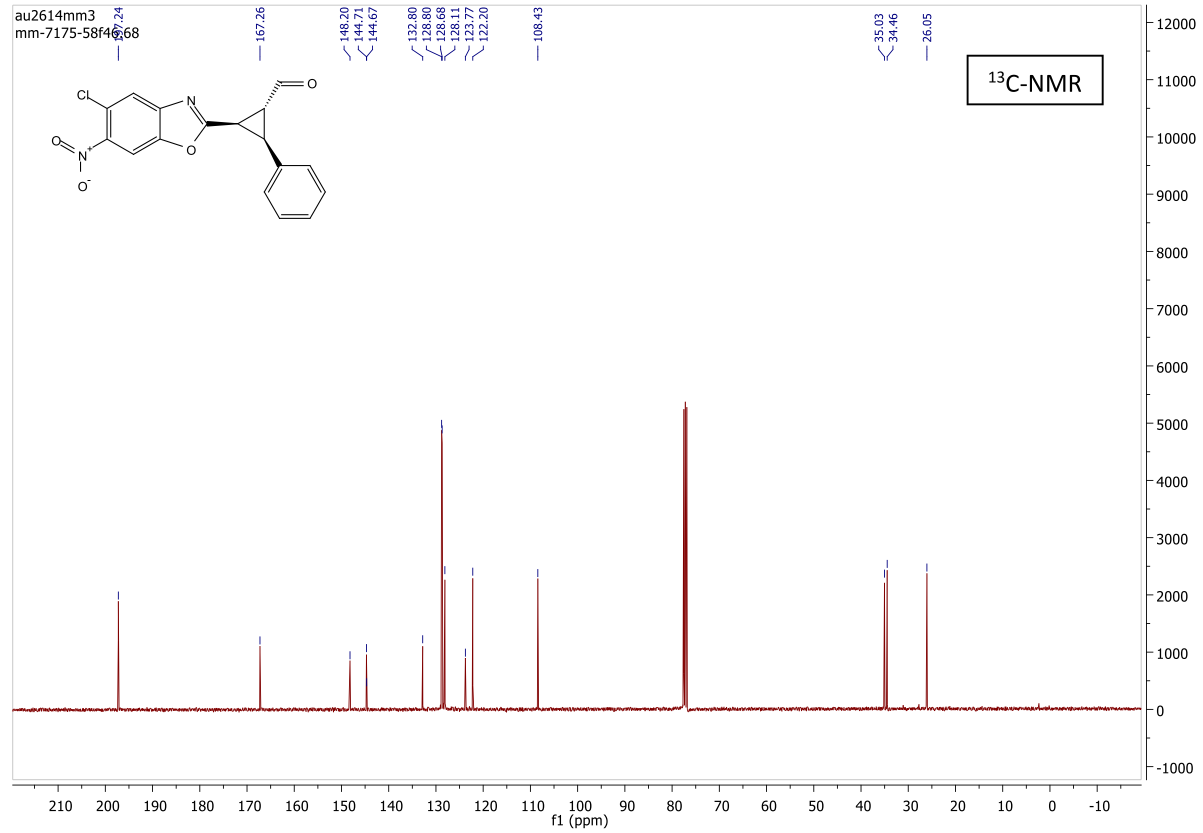

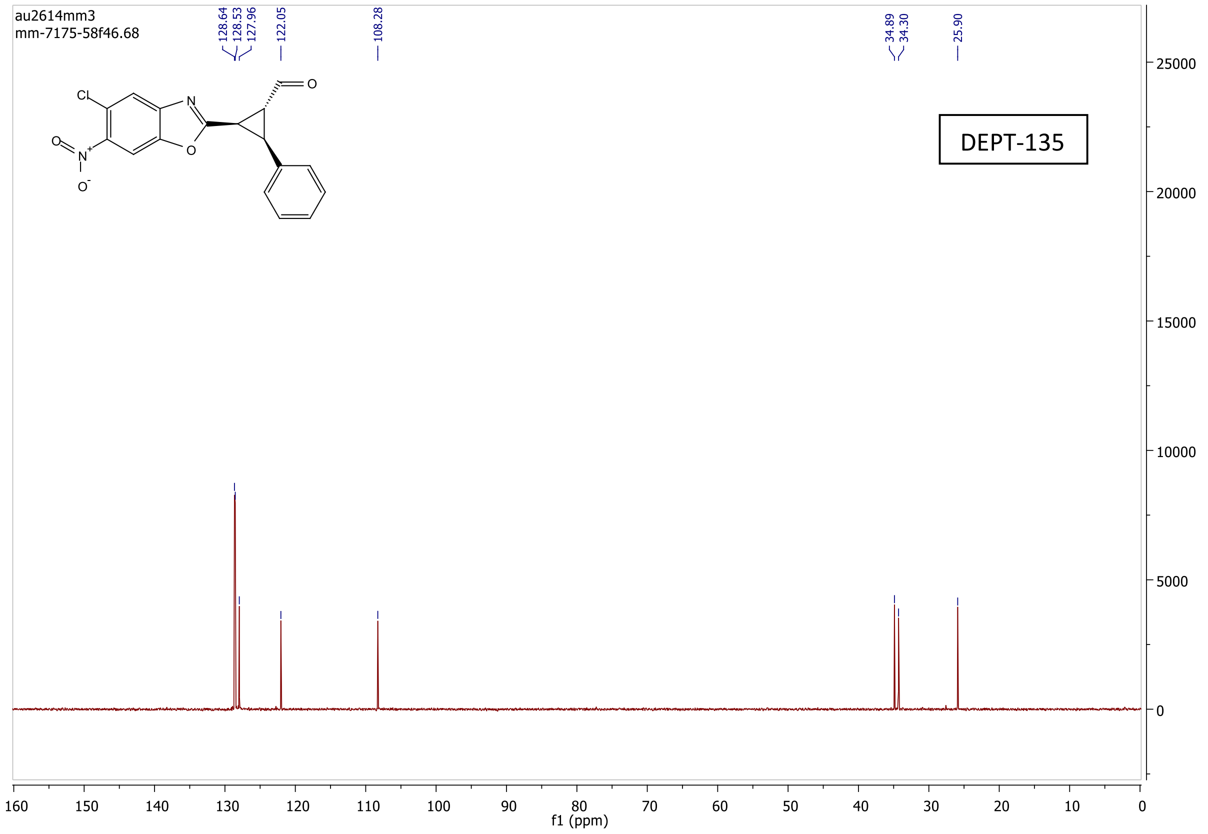

Product **5a** mixture of minor and minor' diastereomers:

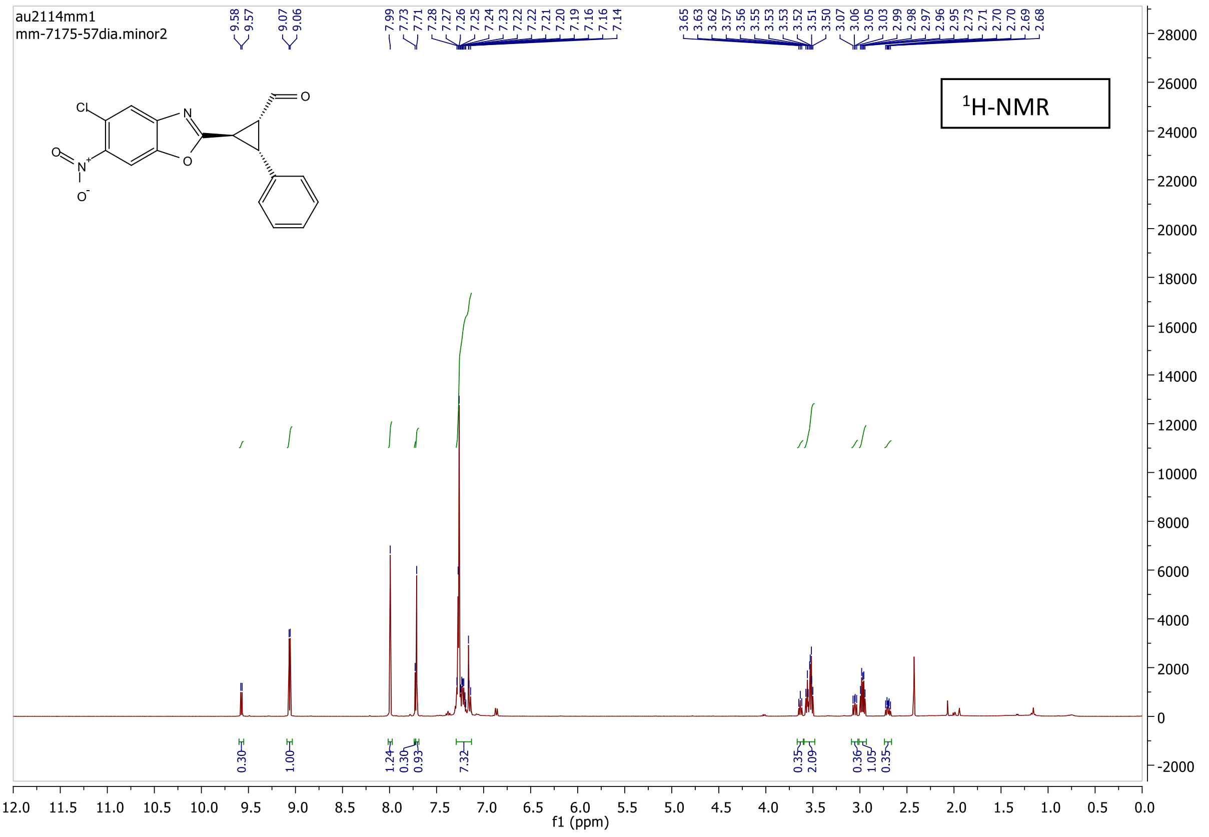

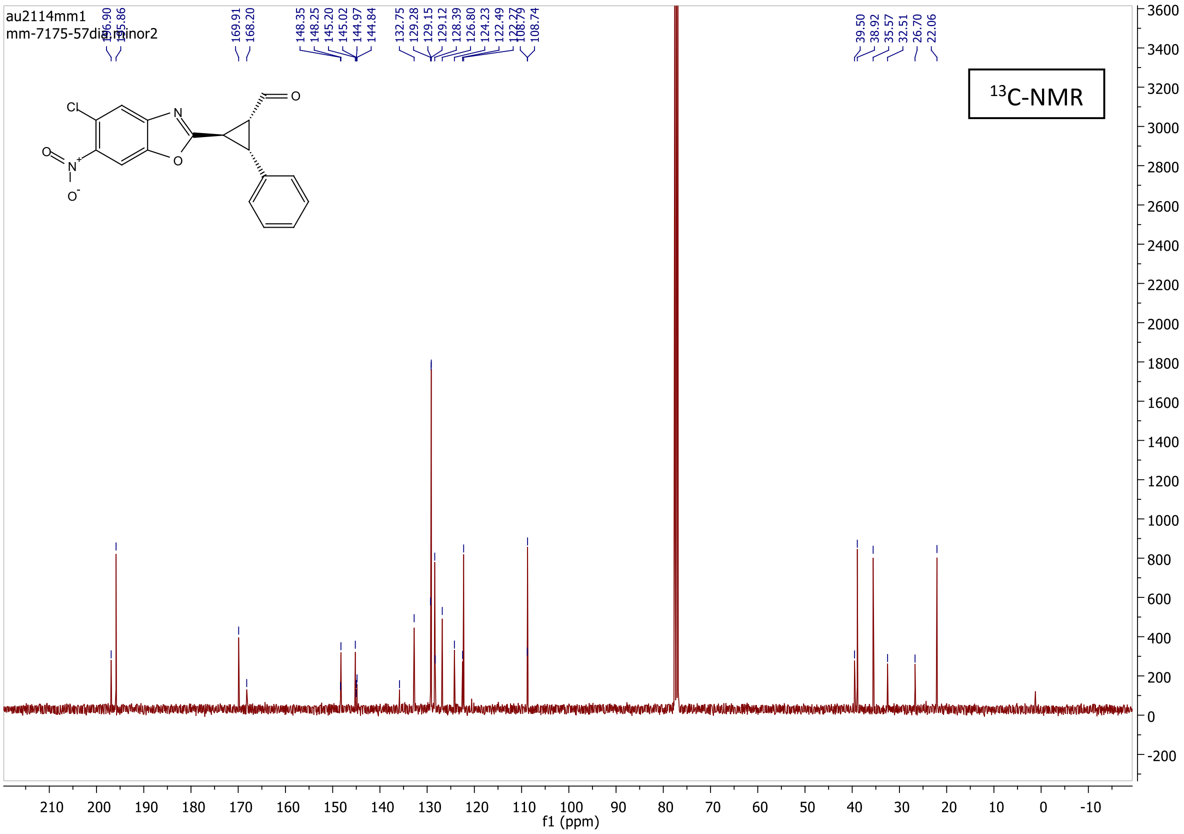
au2114mm1
mm-7175-57dia.minor2
196.90
195.86
169.91
168.20
148.35
148.25
145.20
145.02
144.97
144.84
132.75
129.28
129.15
129.12
128.39
126.80
124.23
122.49
122.27
108.79
108.74
39.50
38.92
35.57
32.51
26.70
22.06
^{13}C-NMR
f1 (ppm)

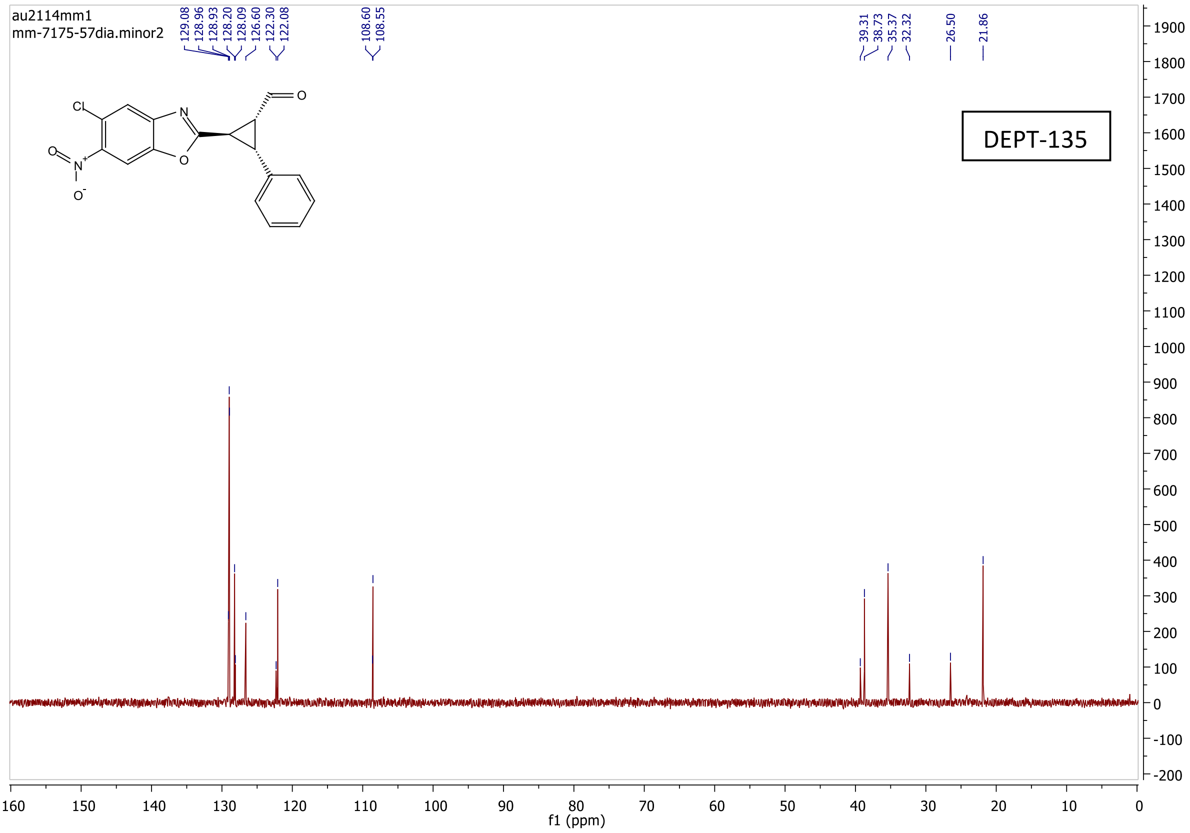
au2114mm1
mm-7175-57dia.minor2
129.08
128.96
128.93
128.20
128.09
126.60
122.30
122.08
108.60
108.55
39.31
38.73
35.37
32.32
26.50
21.86
DEPT-135
f1 (ppm)

Product **5c** major diastereomer:

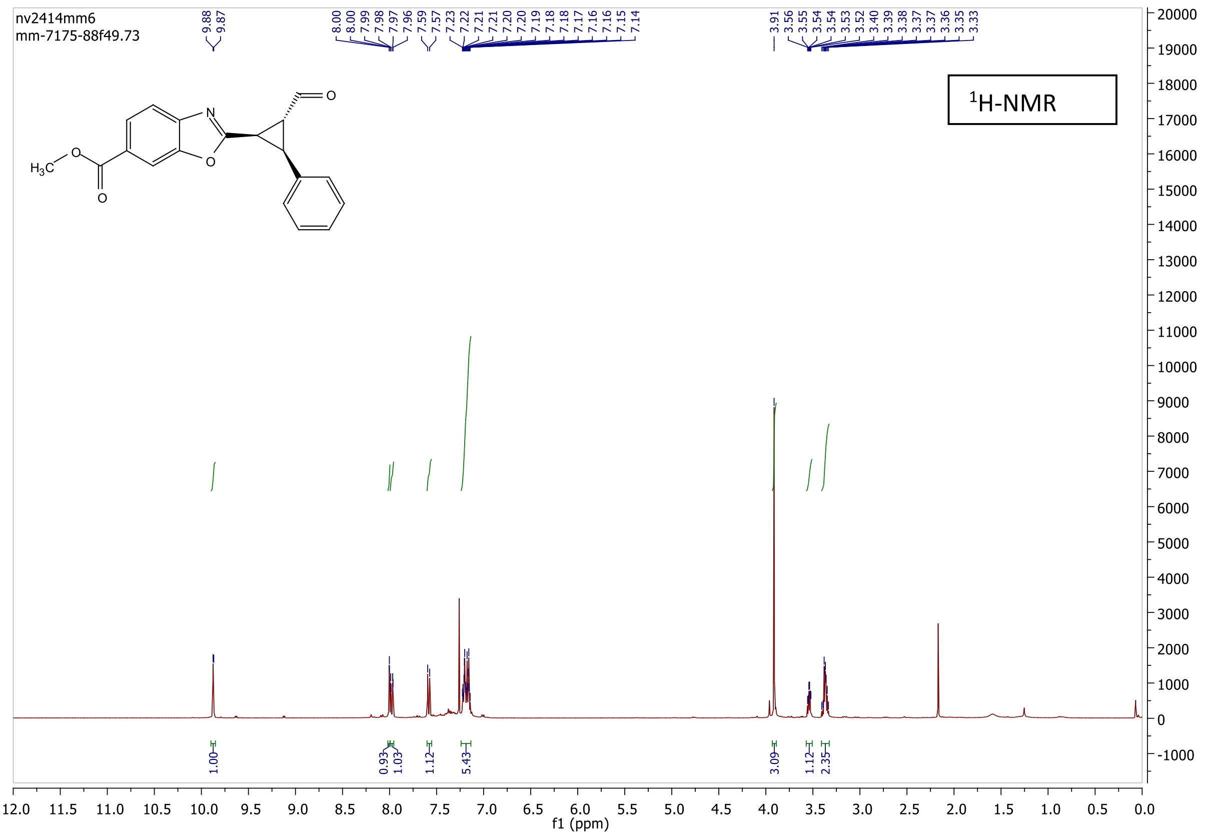

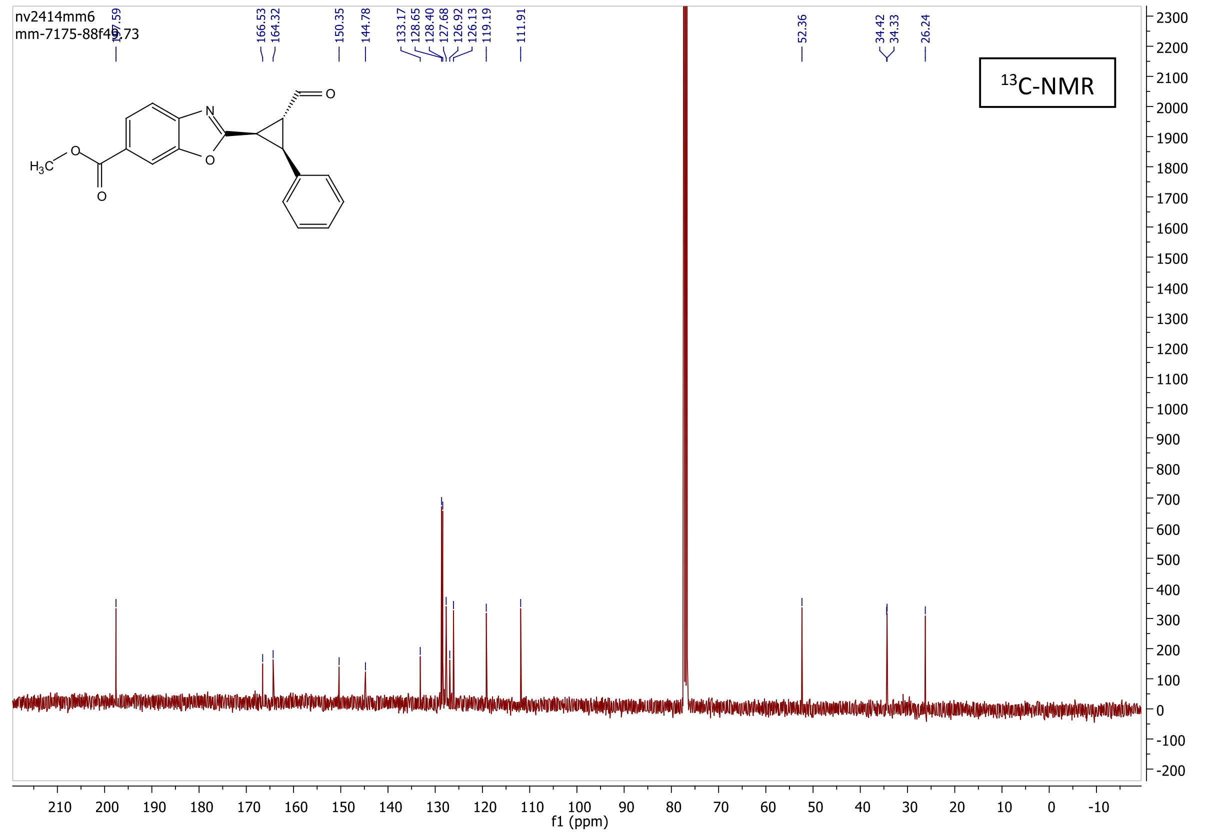

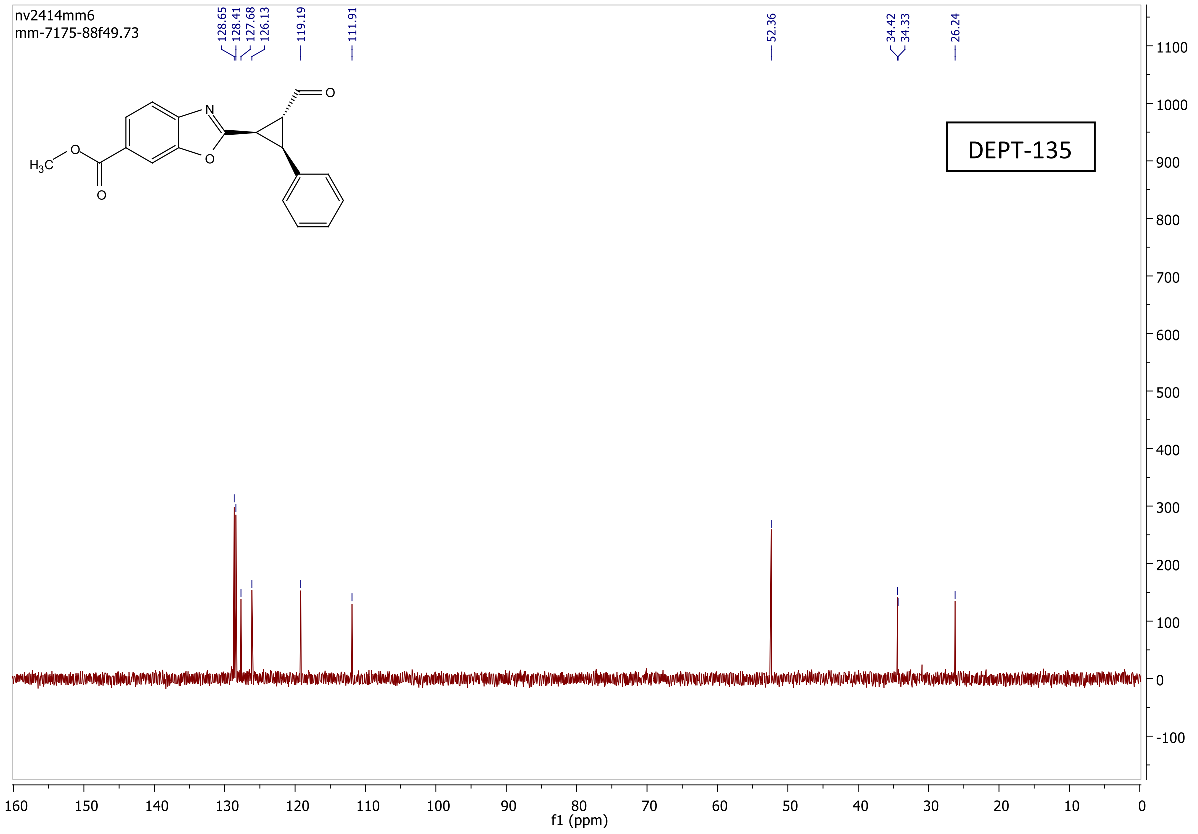

Product **5c** mixture of minor and minor' diastereomers:

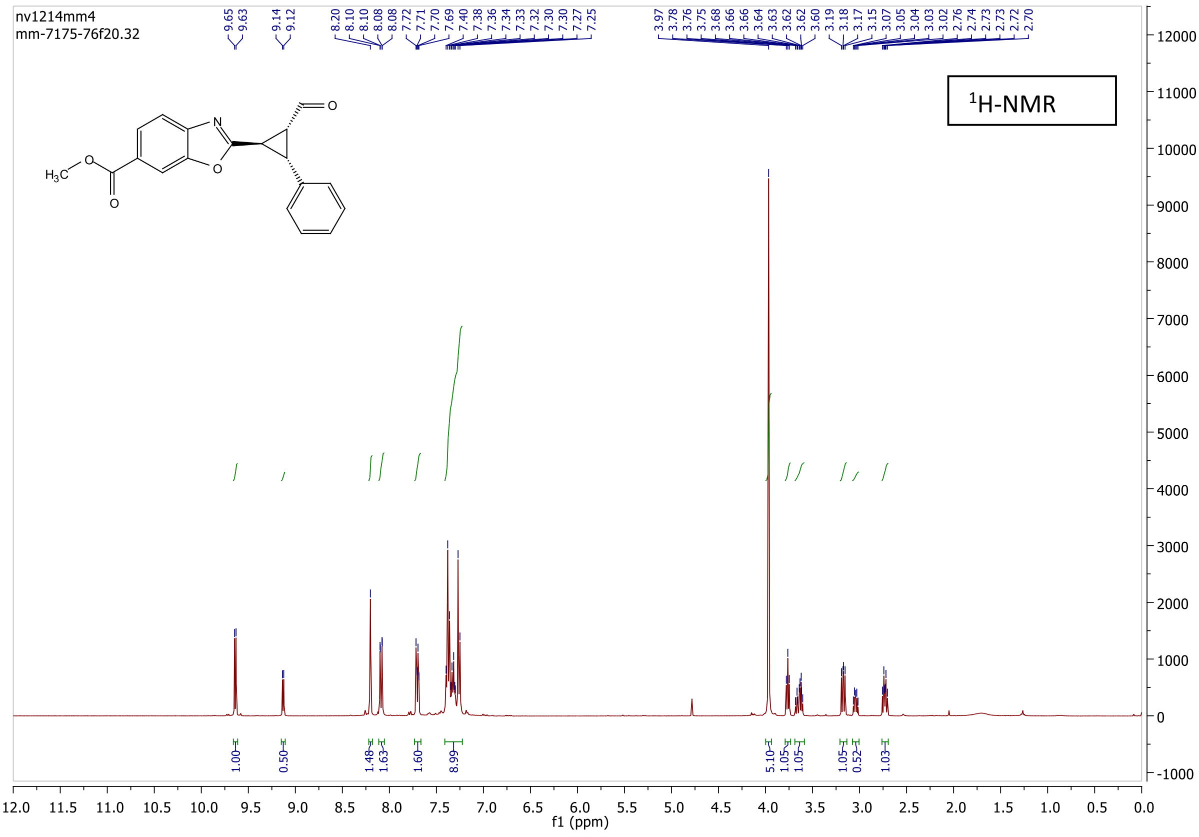

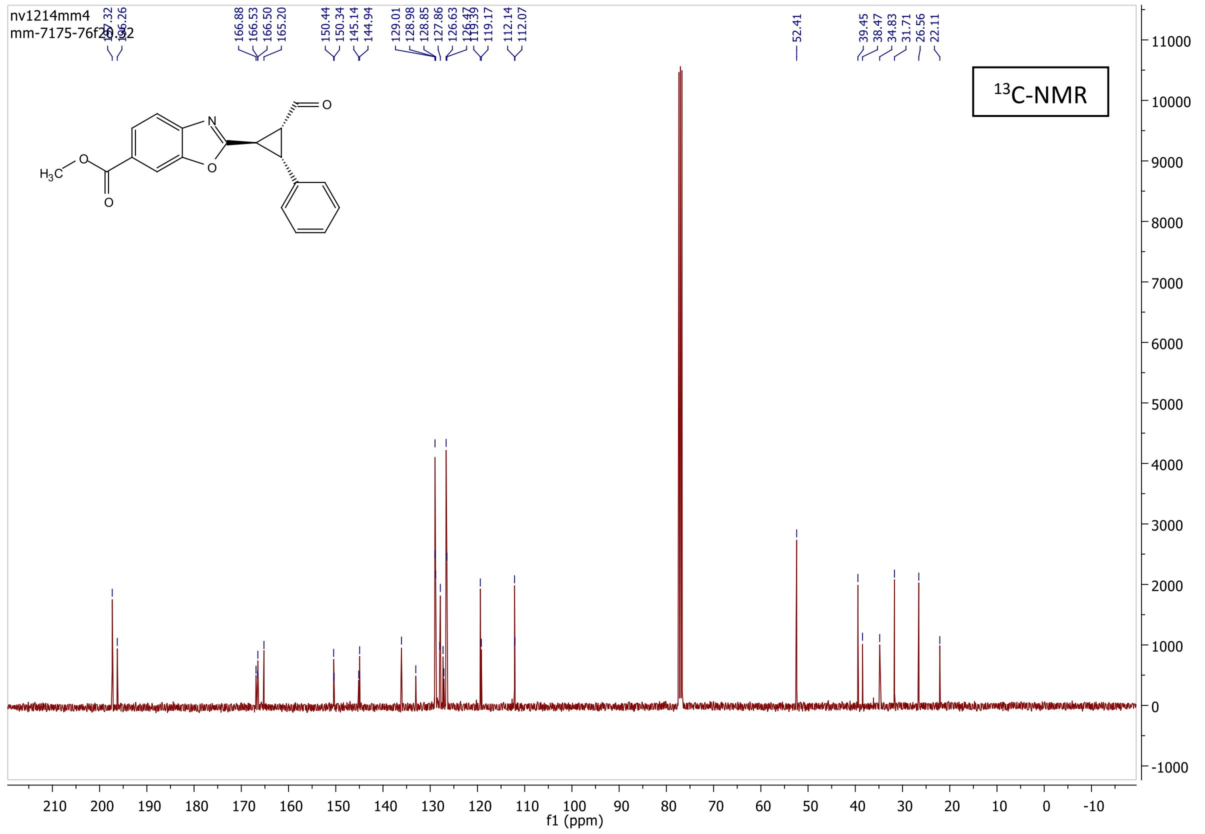
nv1214mm4
mm-7175-76f20.32
166.88
166.53
166.50
165.20
150.44
150.34
145.14
144.94
129.01
128.98
128.85
127.86
126.63
119.17
112.14
112.07
52.41
39.45
38.47
34.83
31.71
26.56
22.11
^{13}C-NMR
f1 (ppm)

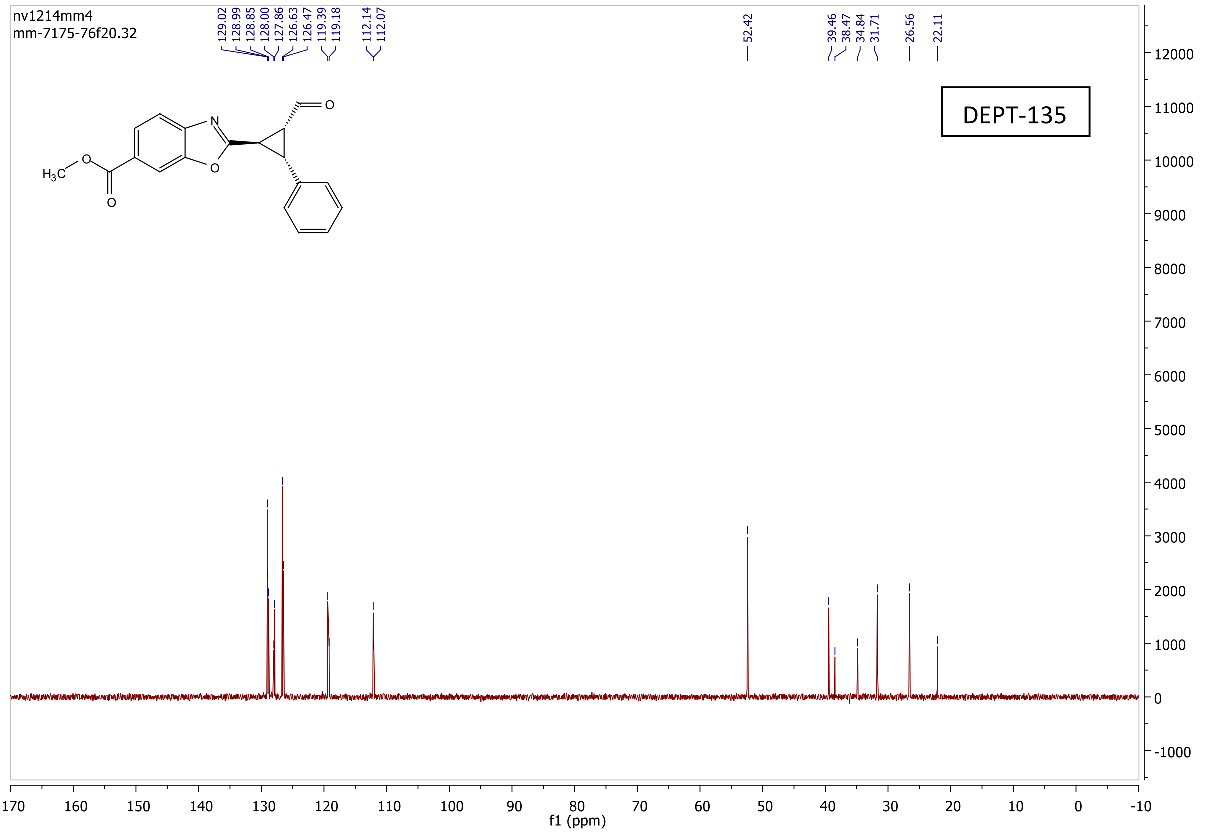
nv1214mm4
mm-7175-76f20.32
129.02
128.99
128.85
128.00
127.86
126.63
126.47
119.39
119.18
112.14
112.07
52.42
39.46
38.47
34.84
31.71
26.56
22.11
DEPT-135
f1 (ppm)

Product **5b** major diastereomer:

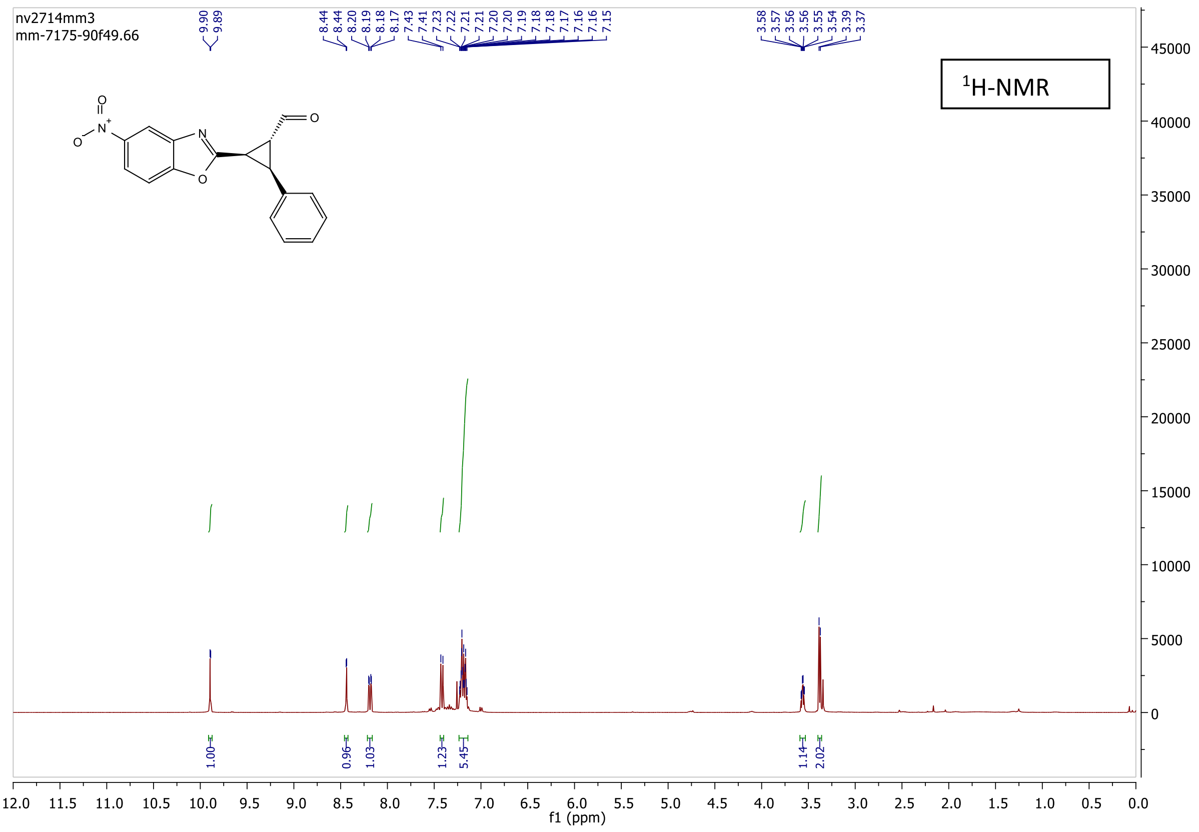

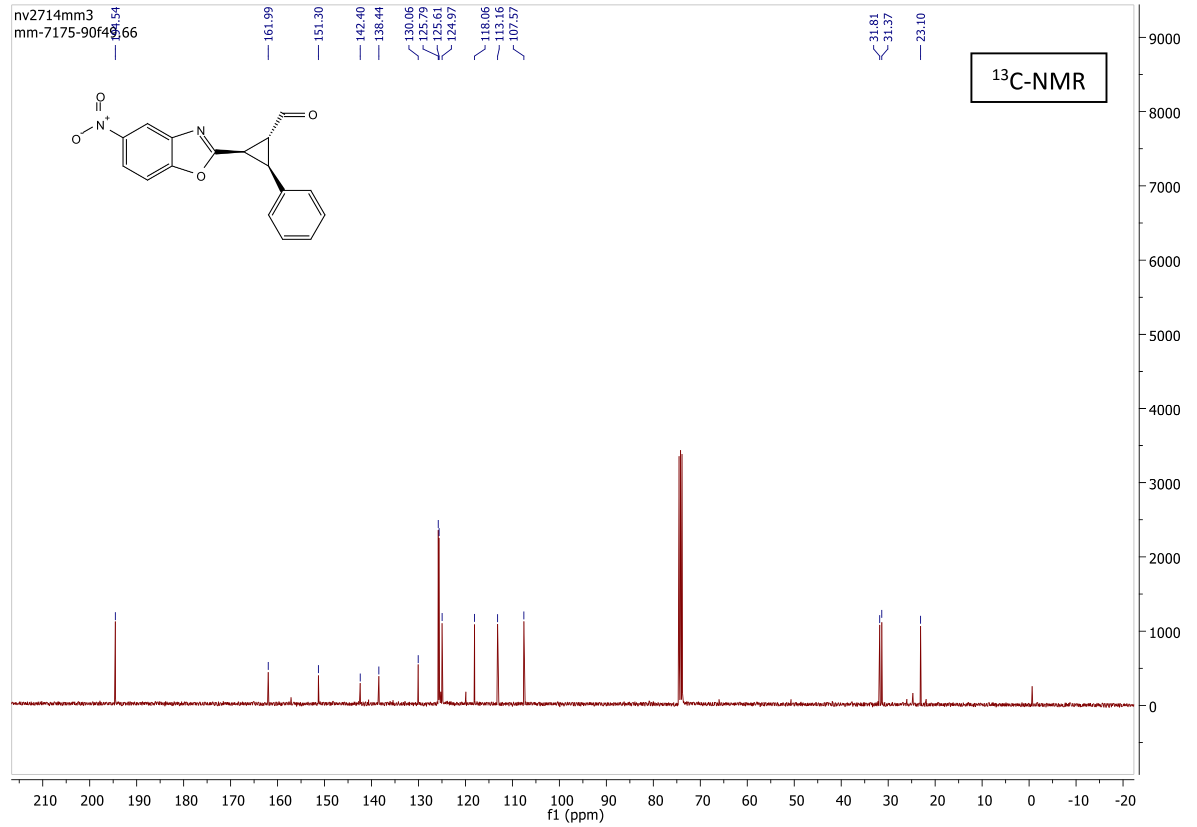

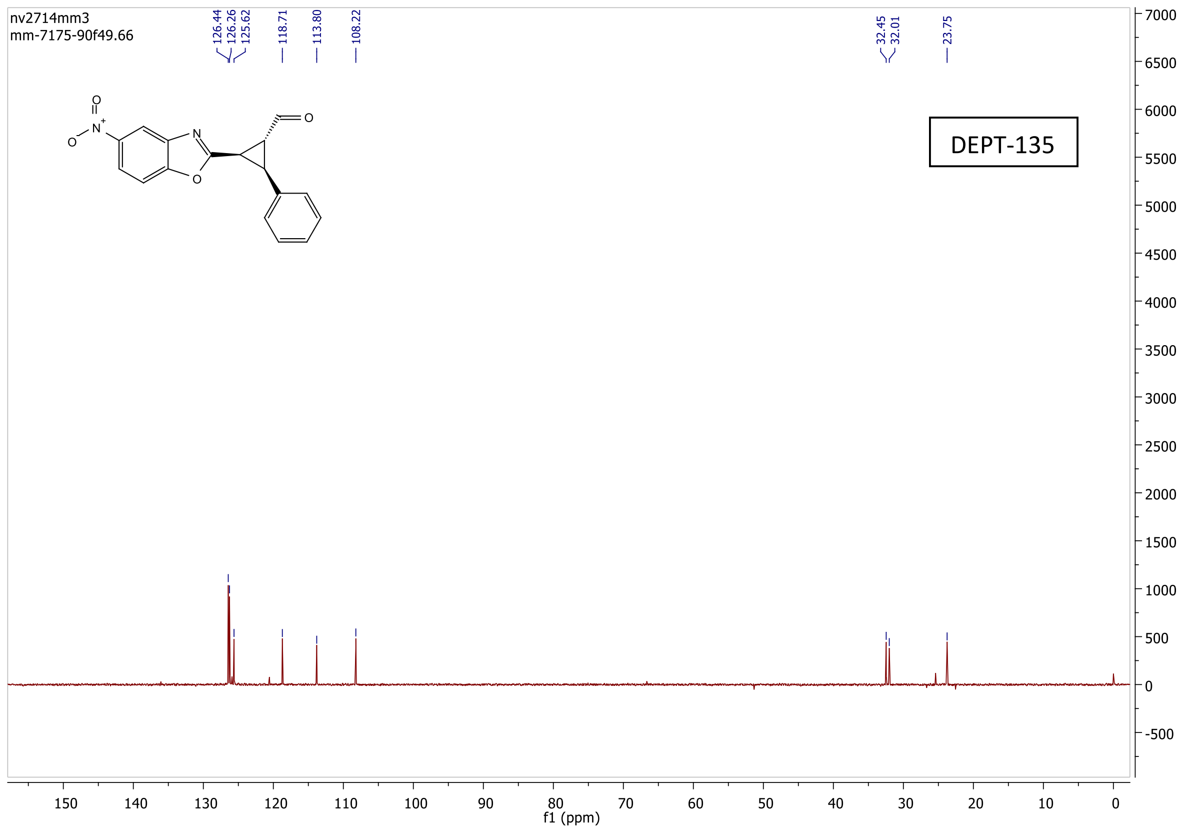

Product **5b** mixture of minor and minor’ diastereomers:

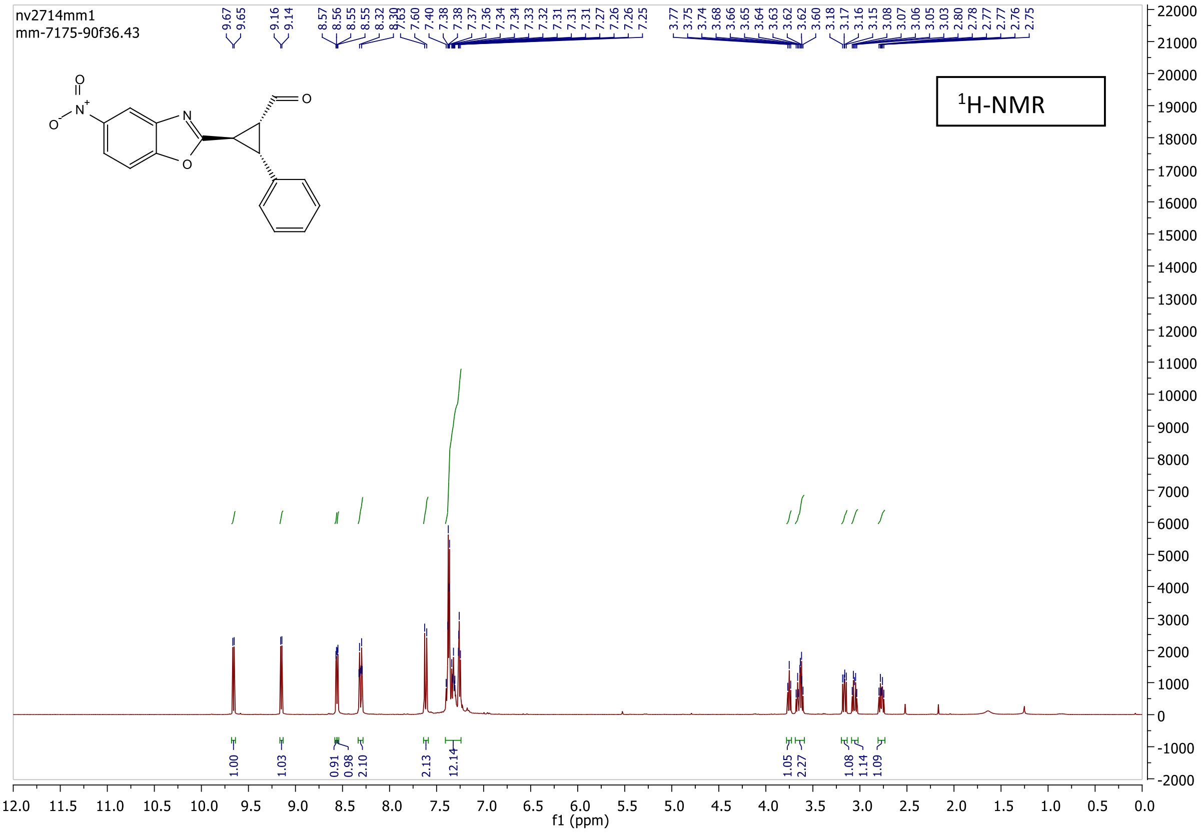

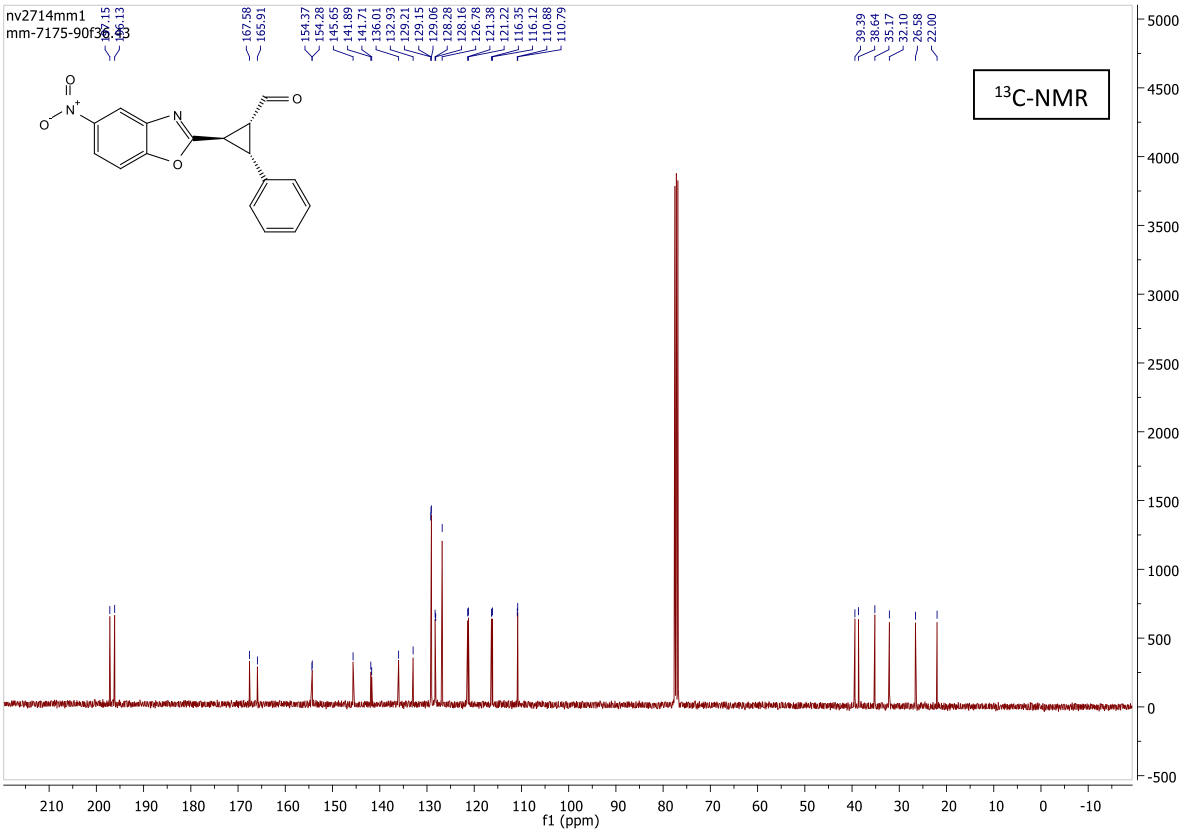
nv2714mm1
mm-7175-90f36.43
13C-NMR
f1 (ppm)

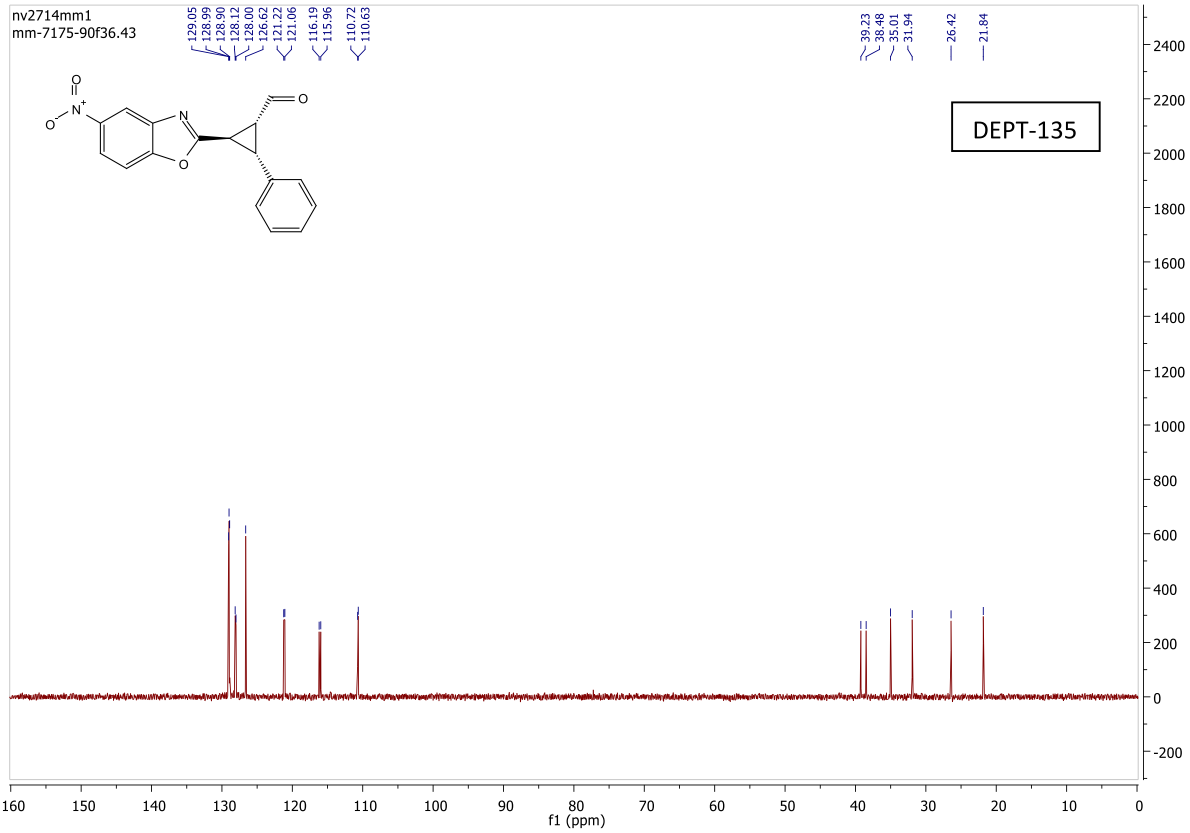
nv2714mm1
mm-7175-90f36.43
DEPT-135
f1 (ppm)

Product **5d** mixture of major and minor diastereomers:

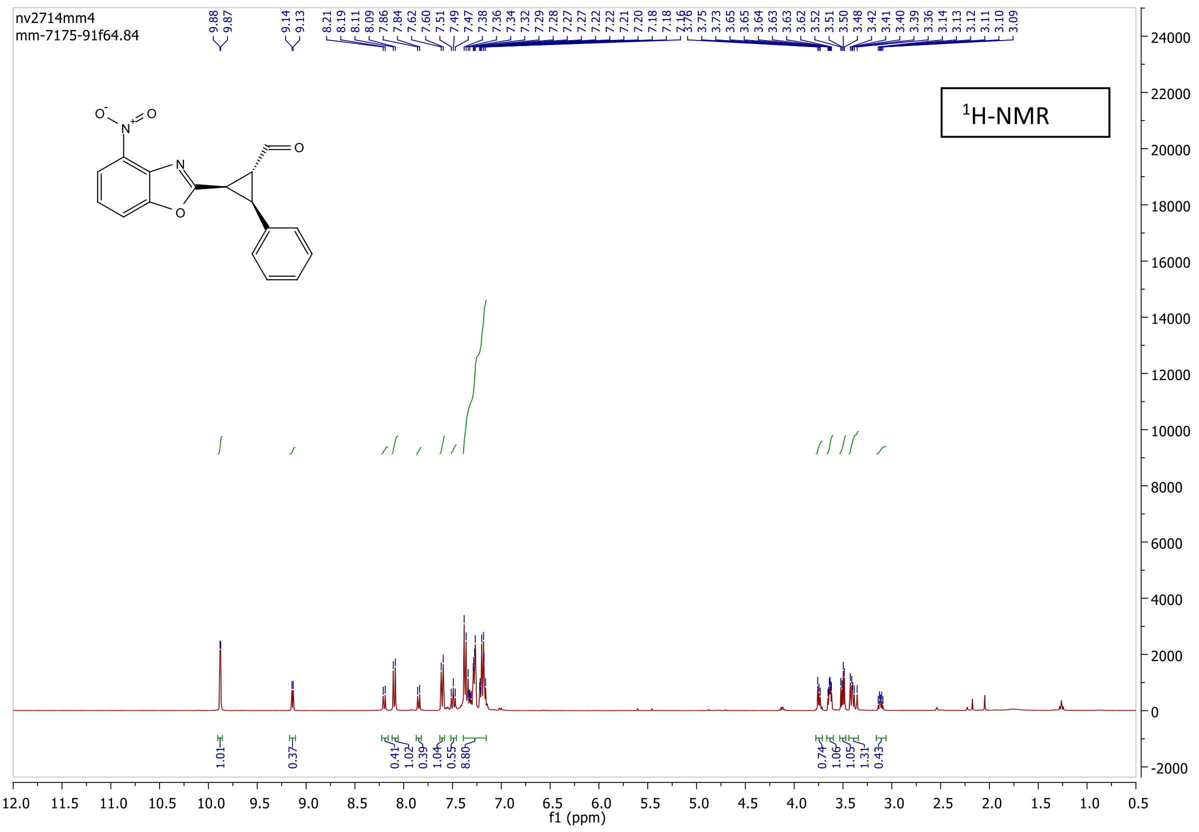

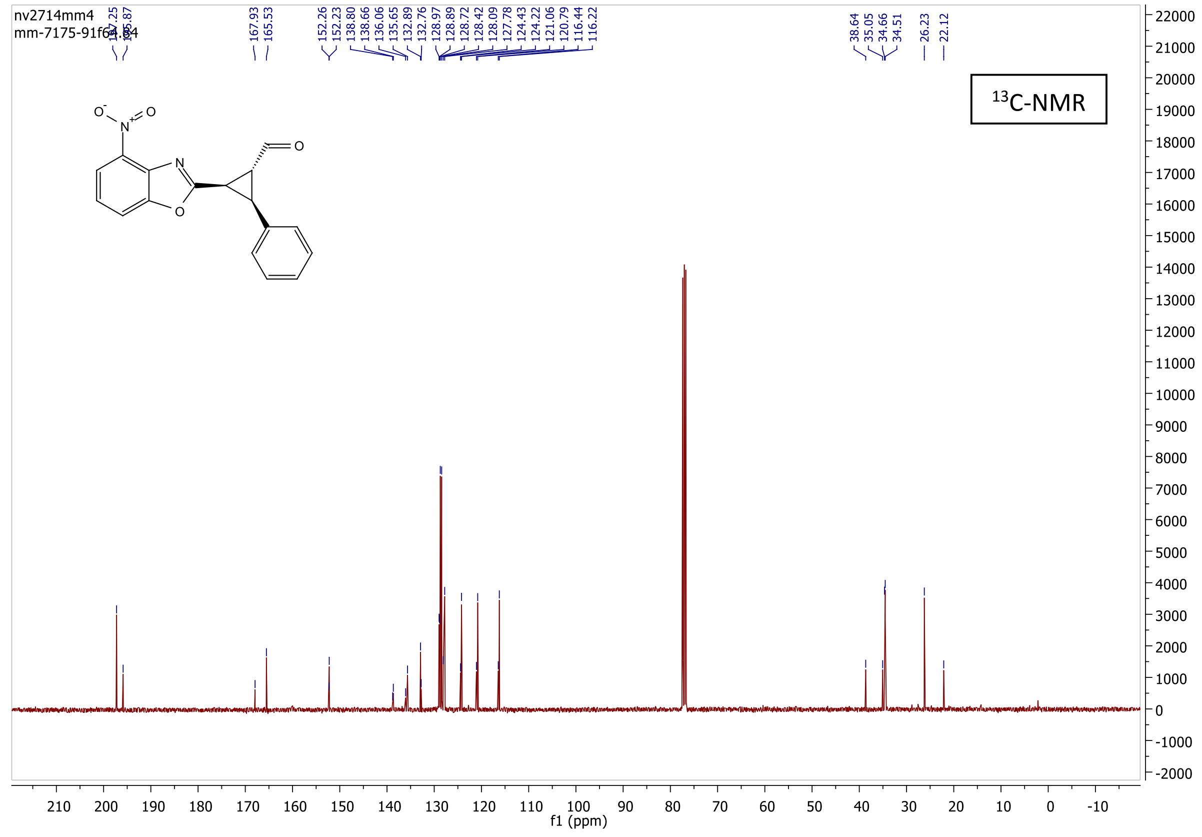

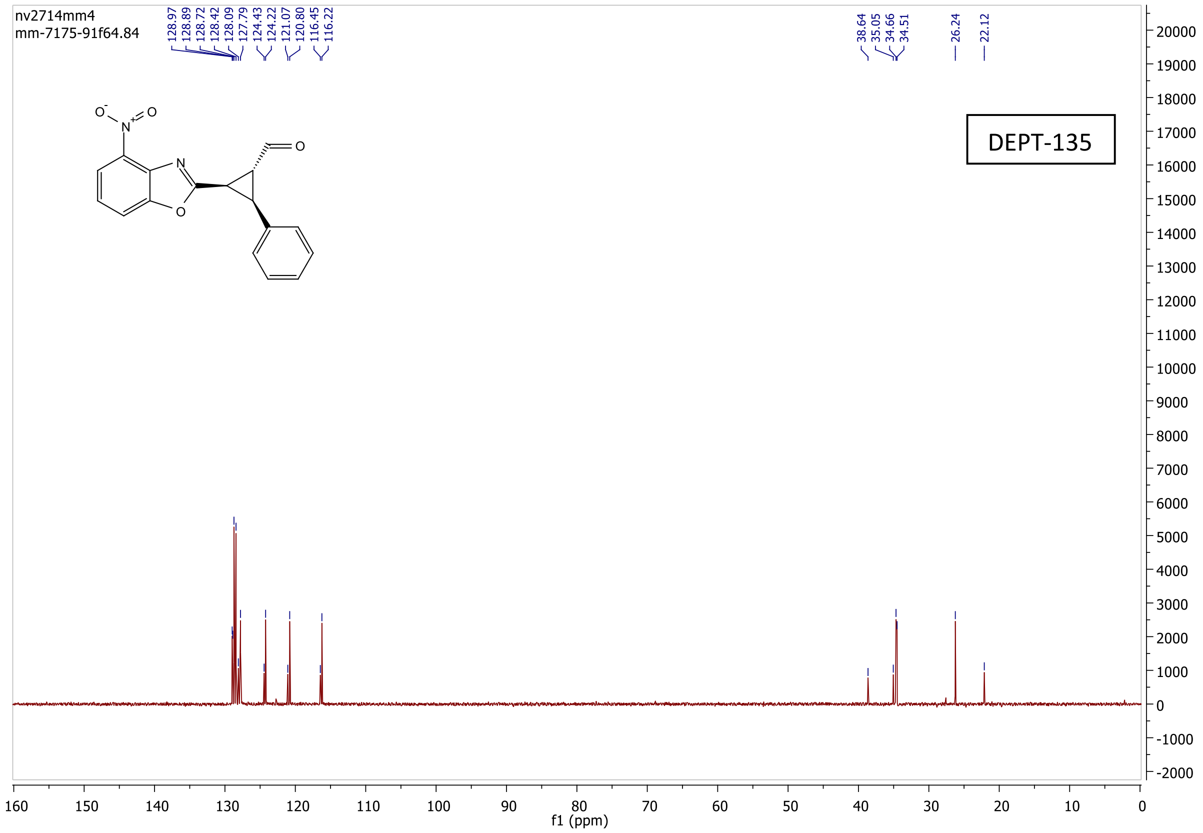

Product **5b** mixture of major minor ad minor’ diastereomers:

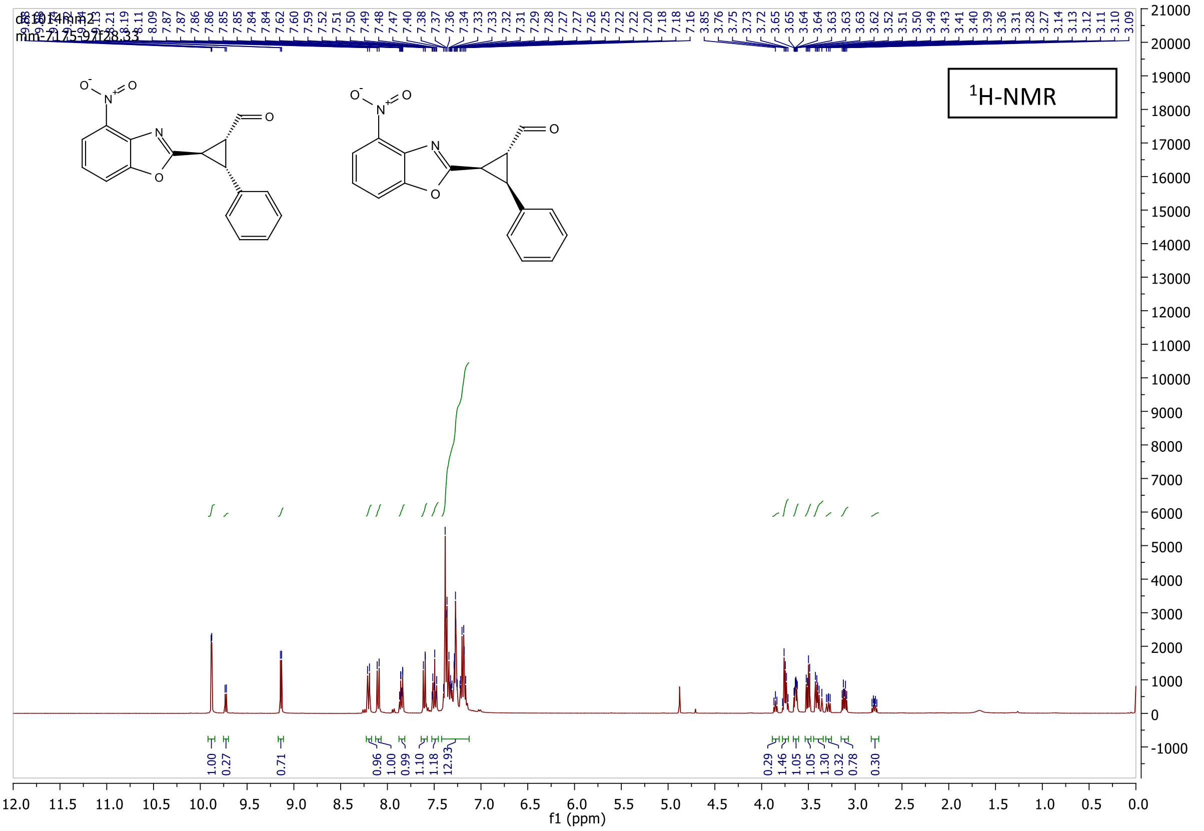

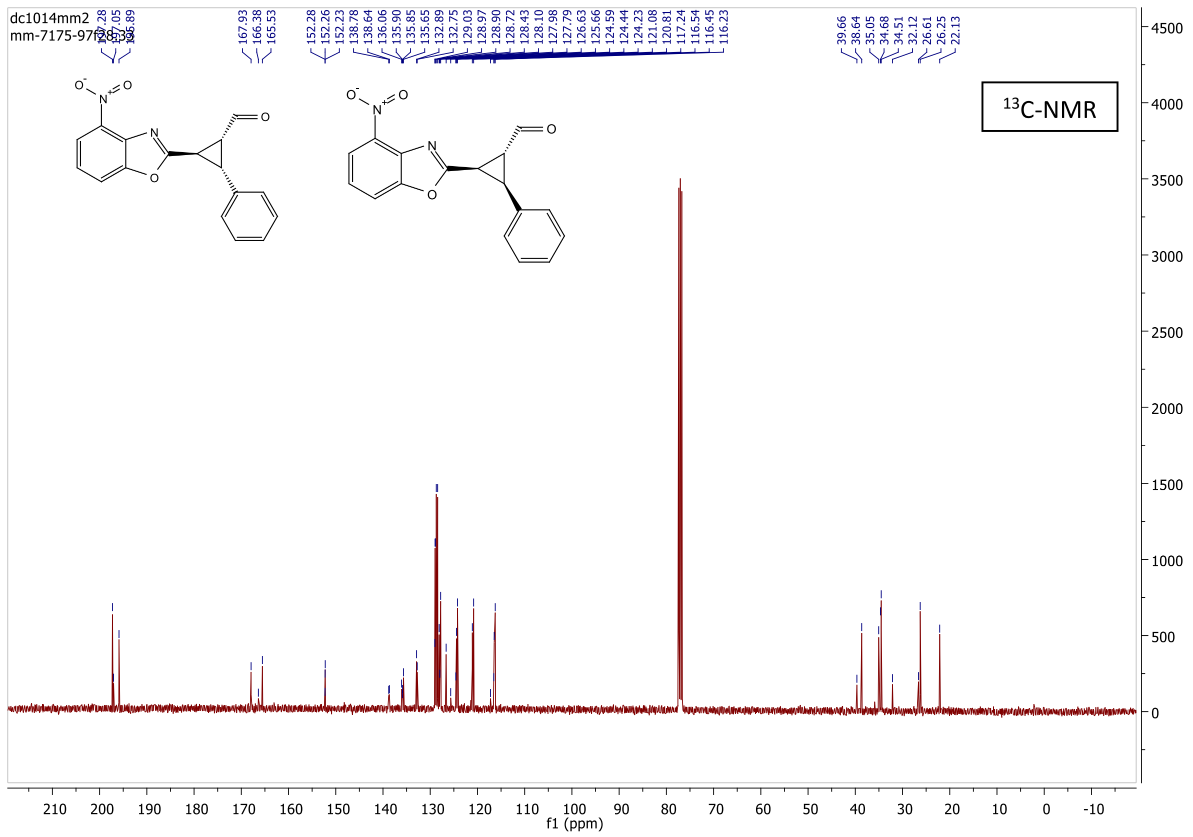
dc1014mm2
mm-7175-97f28.33
13C-NMR
f1 (ppm)

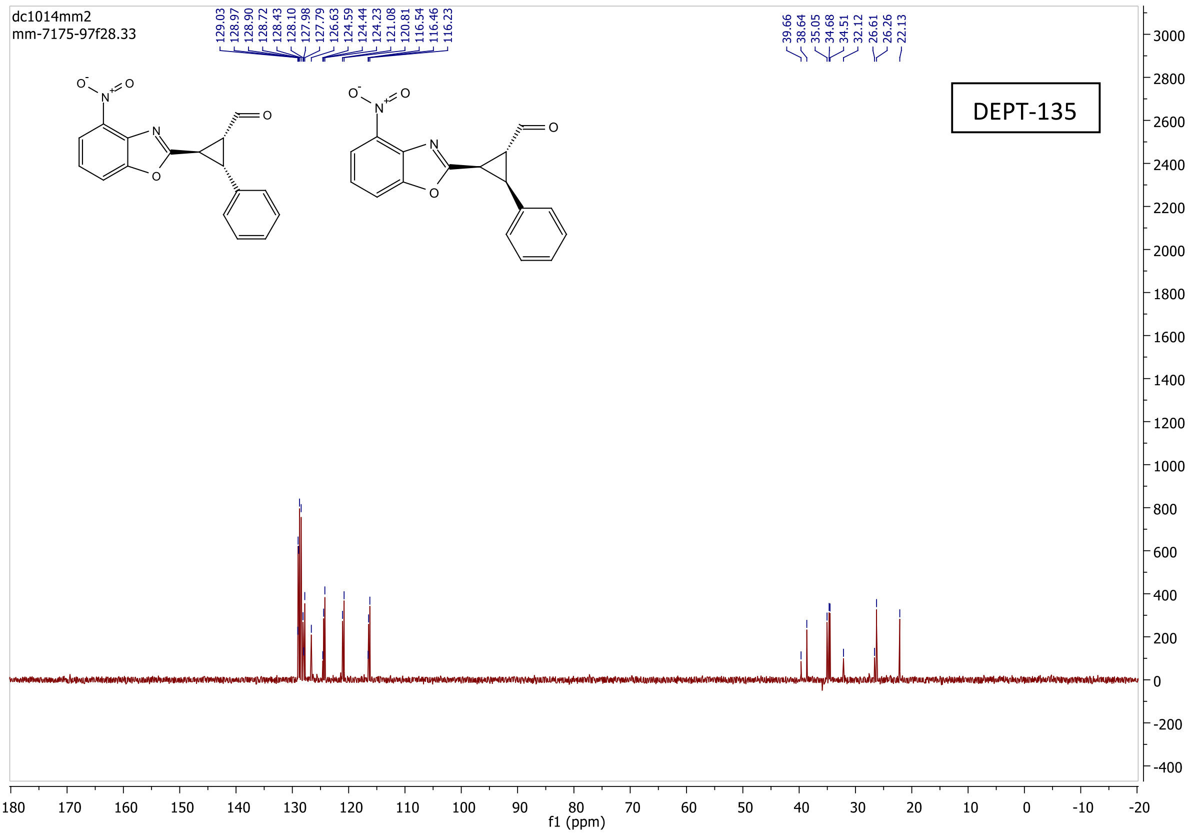
dc1014mm2
mm-7175-97f28.33
DEPT-135
f1 (ppm)

Product **5e** major diastereomer:

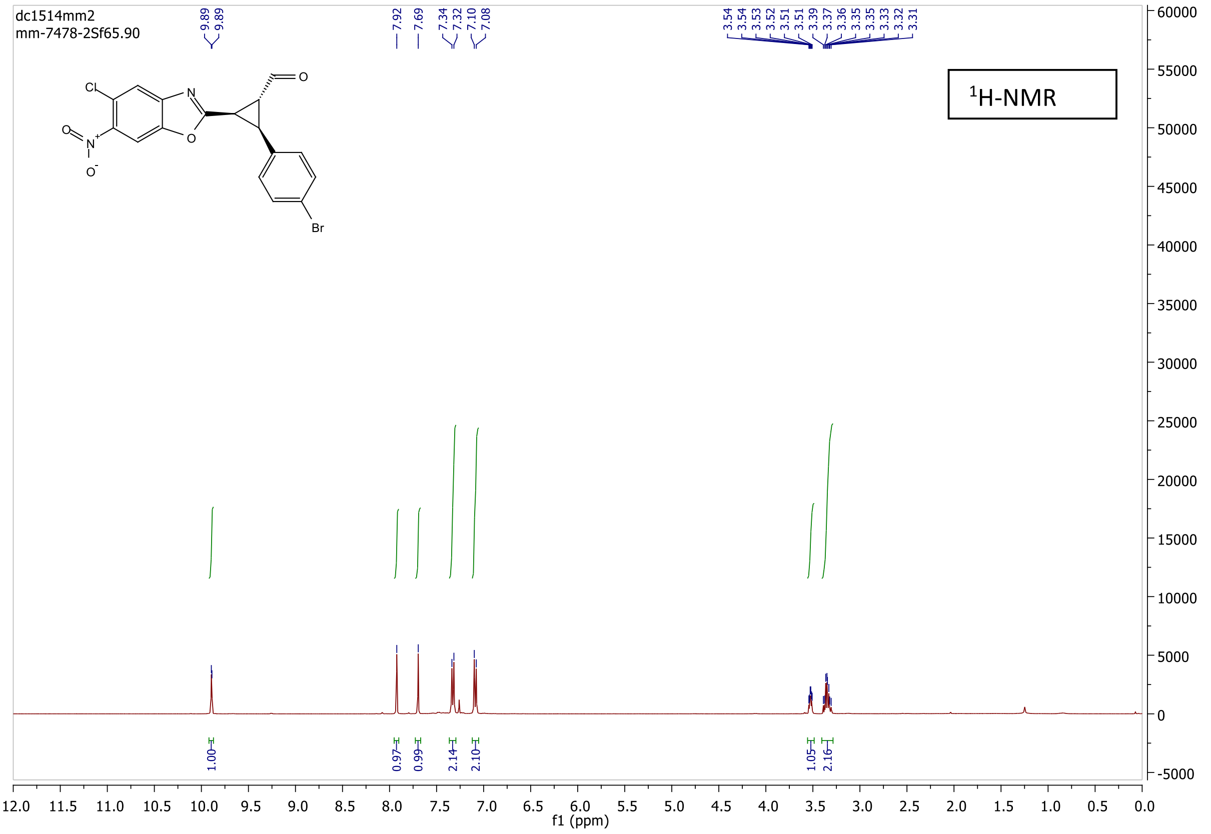

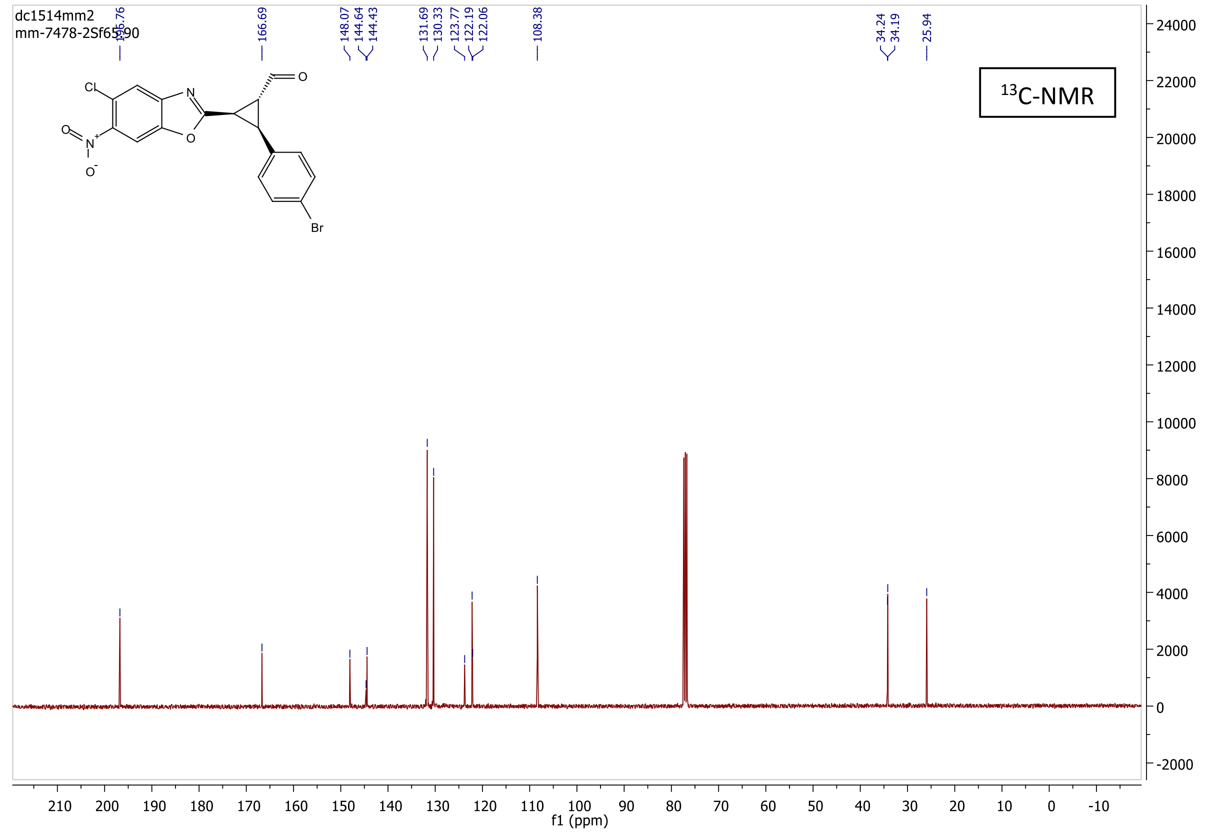

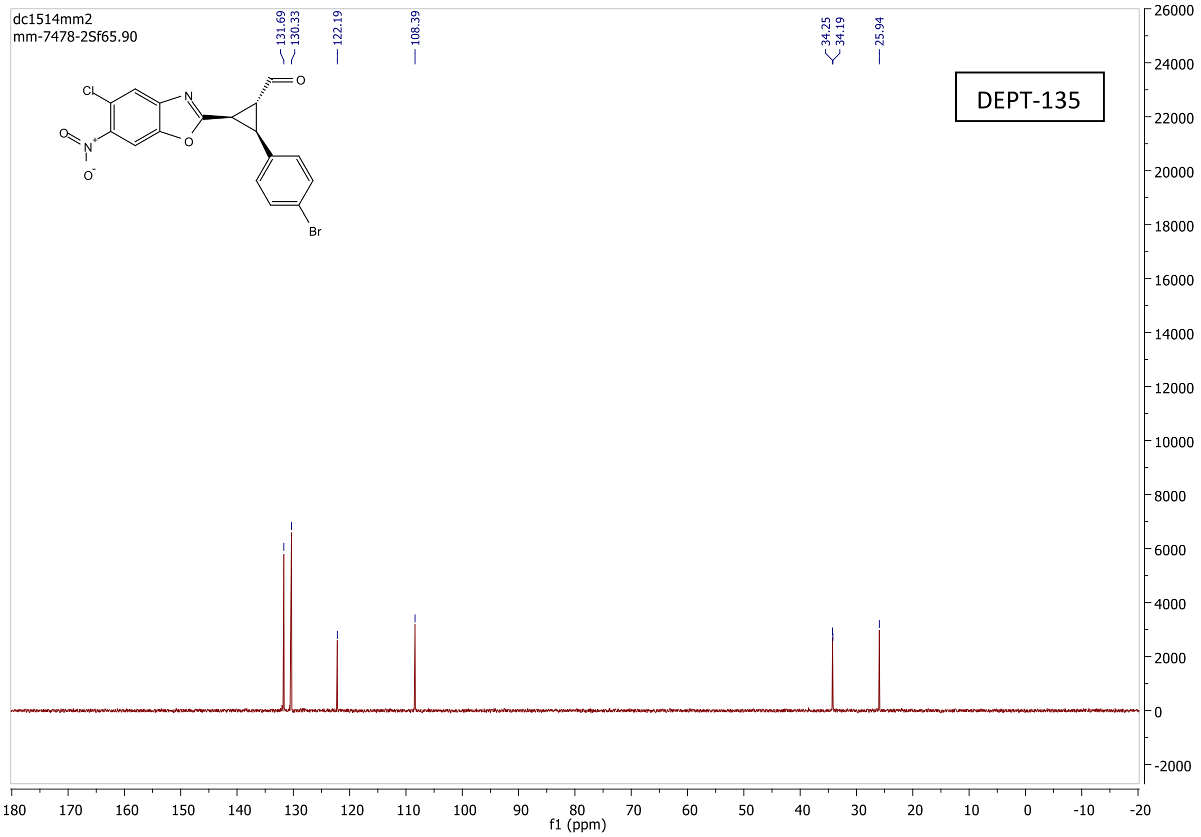

Product **5e** mixture of minor and minor ‘ diastereomers:

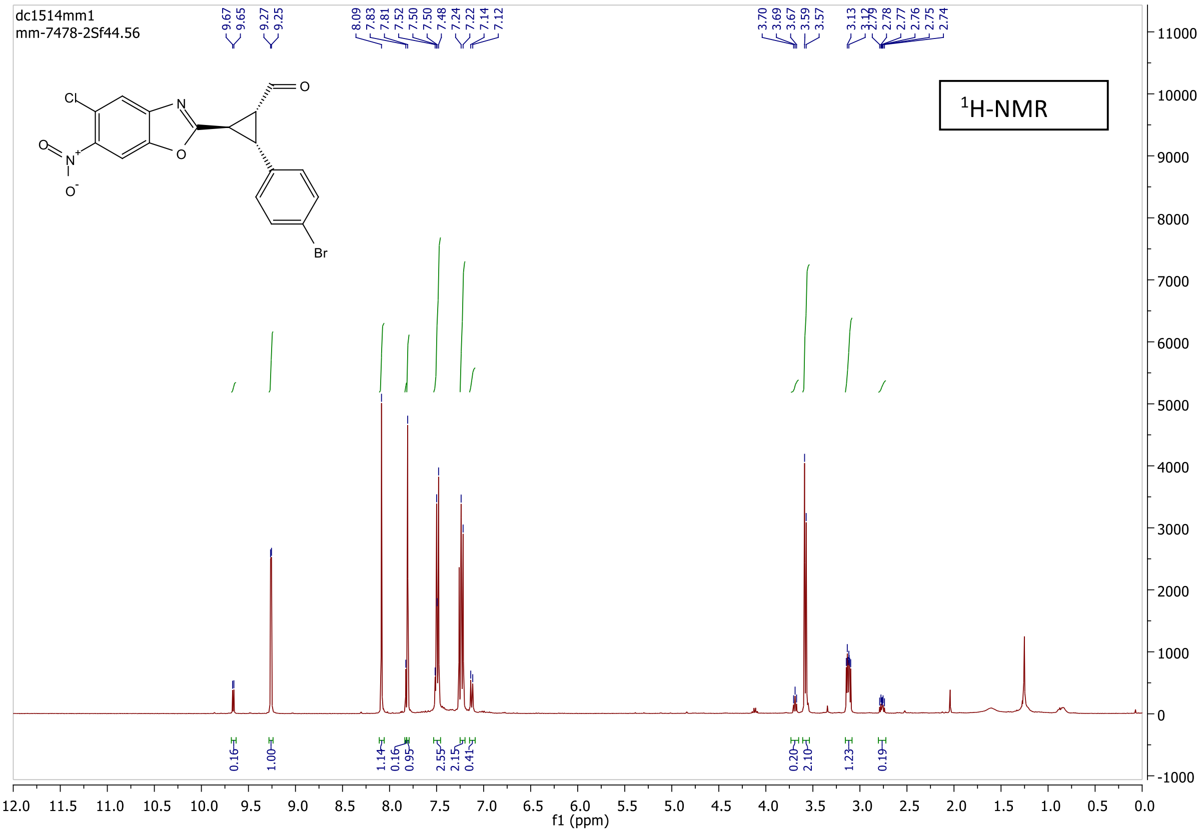

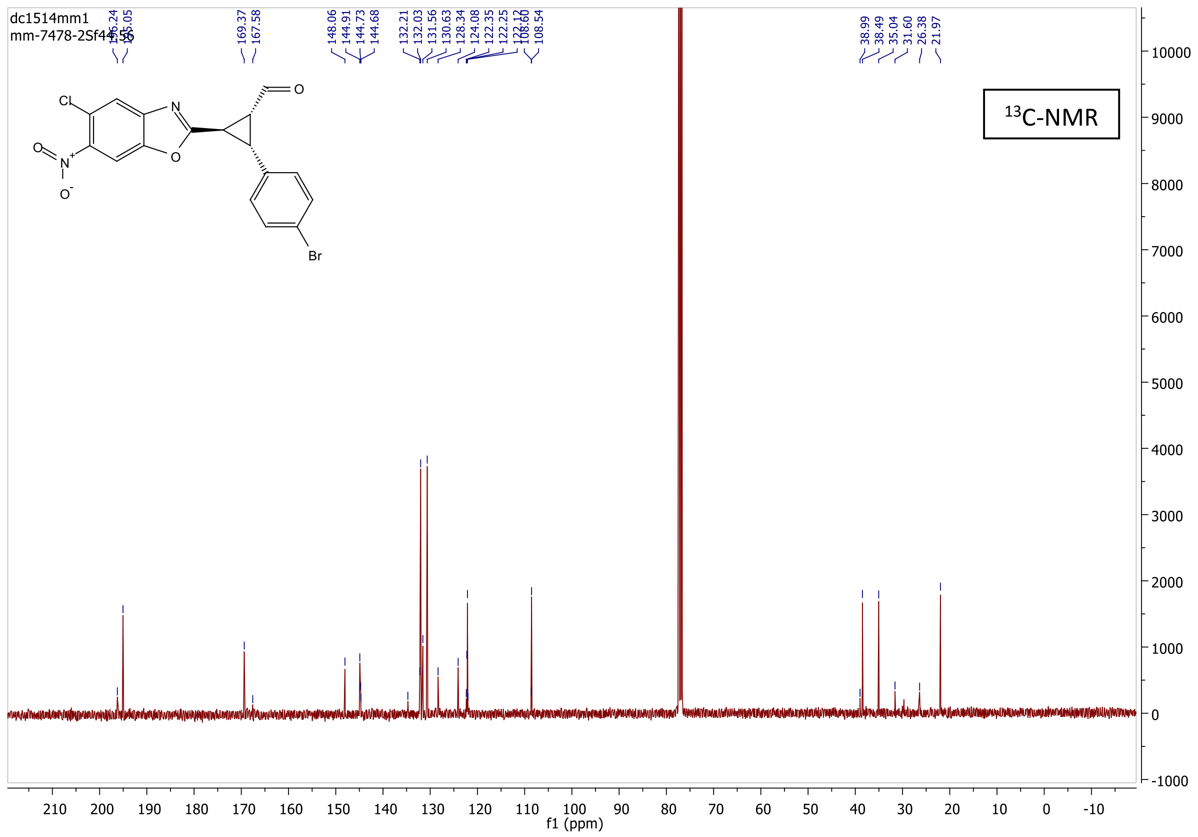
dc1514mm1
mm-7478-2Sf44.56
13C-NMR
f1 (ppm)

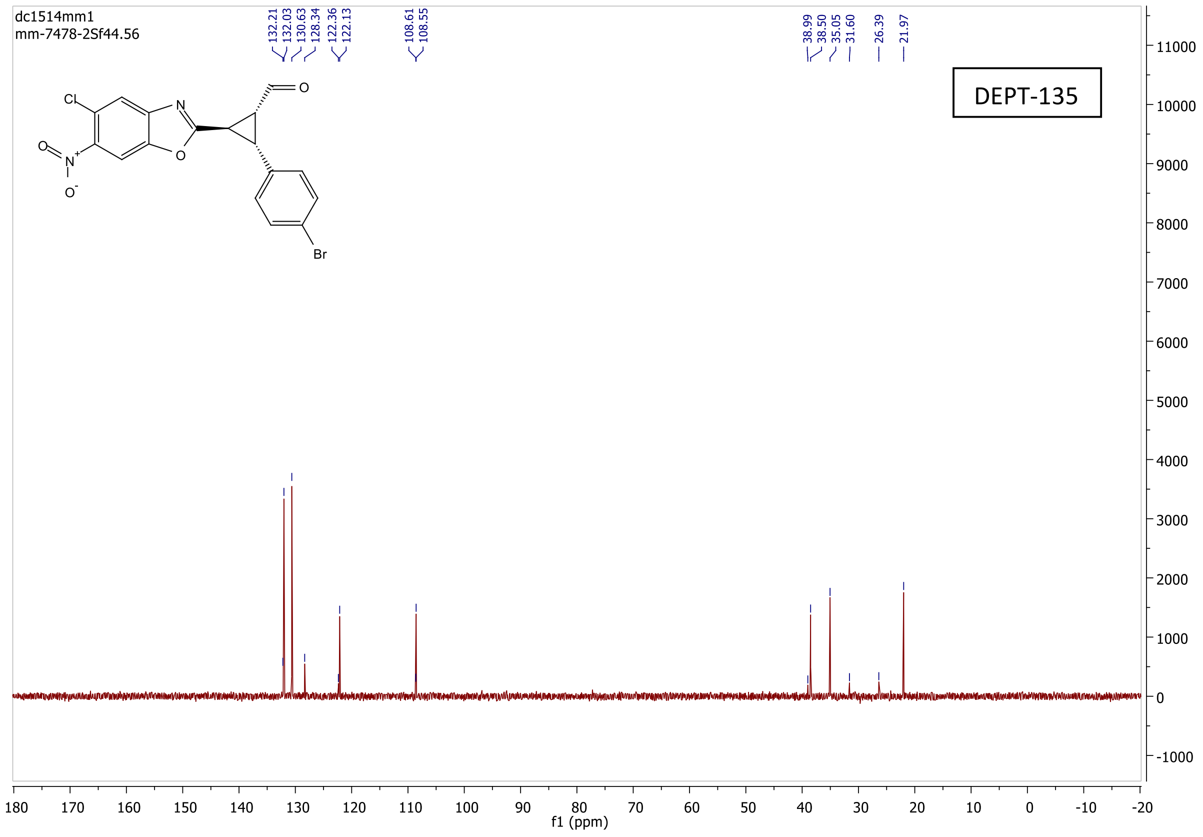
dc1514mm1
mm-7478-2Sf44.56
DEPT-135
f1 (ppm)

Product **5f** major diastereomer:

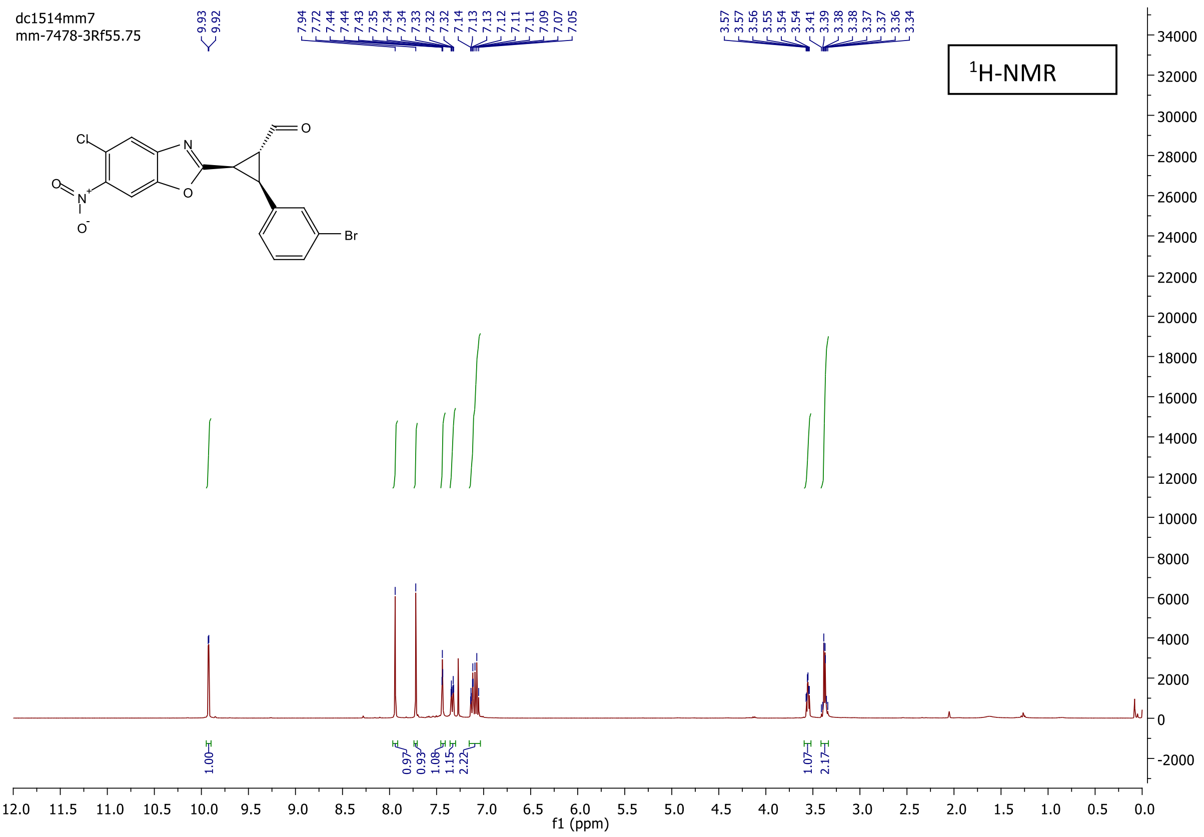

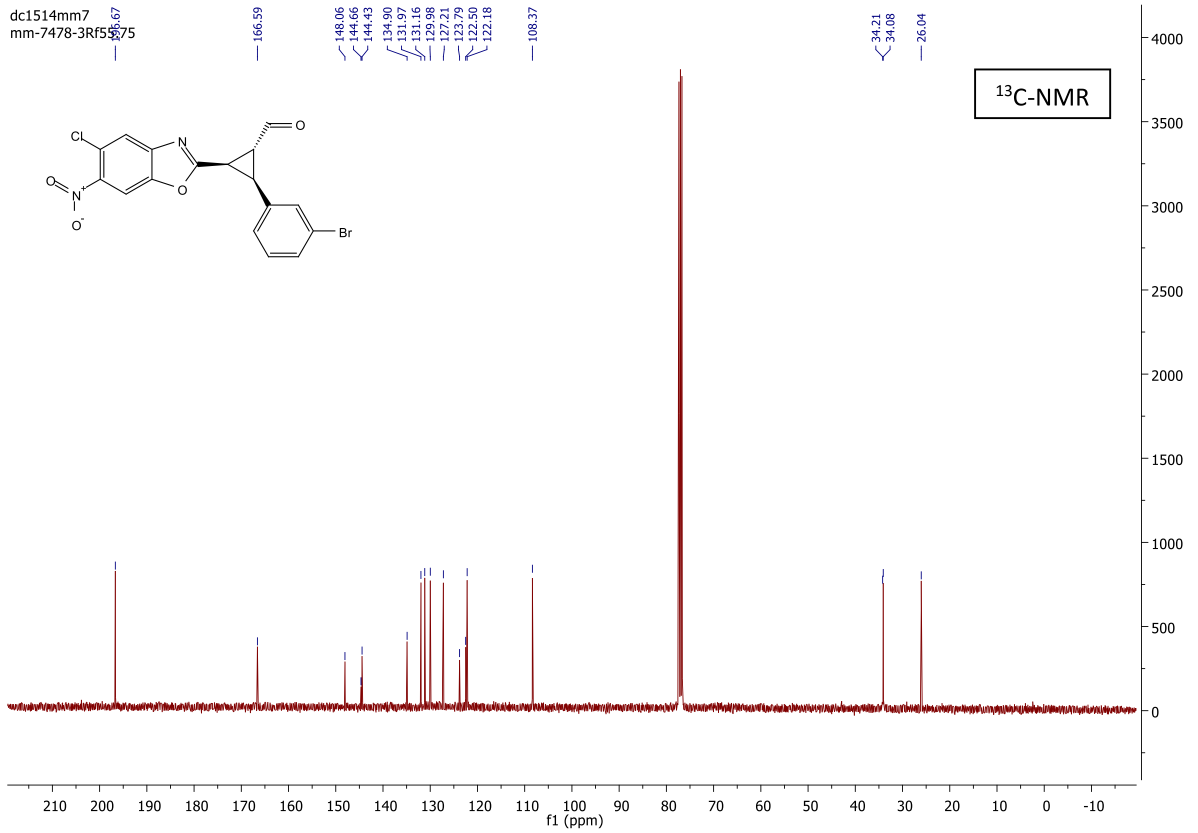

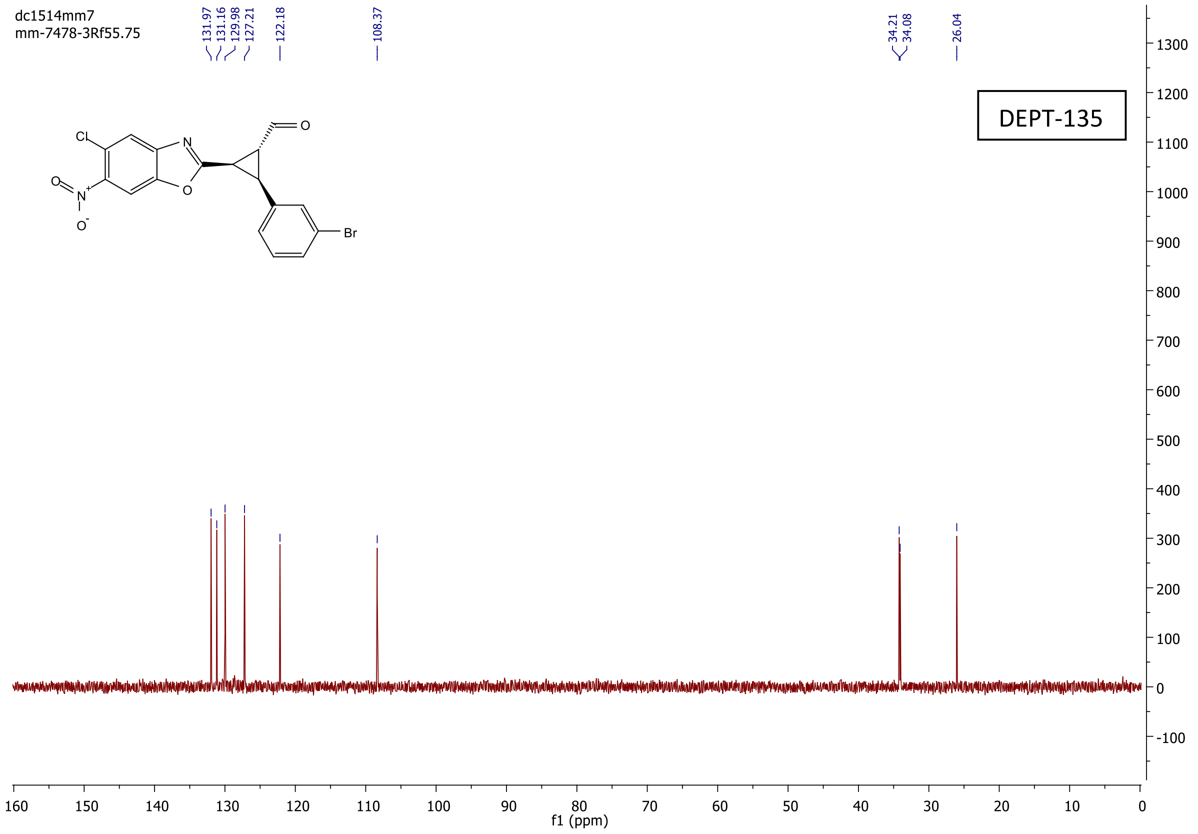

Product **5f** mixture of minor and minor' diastereomers:

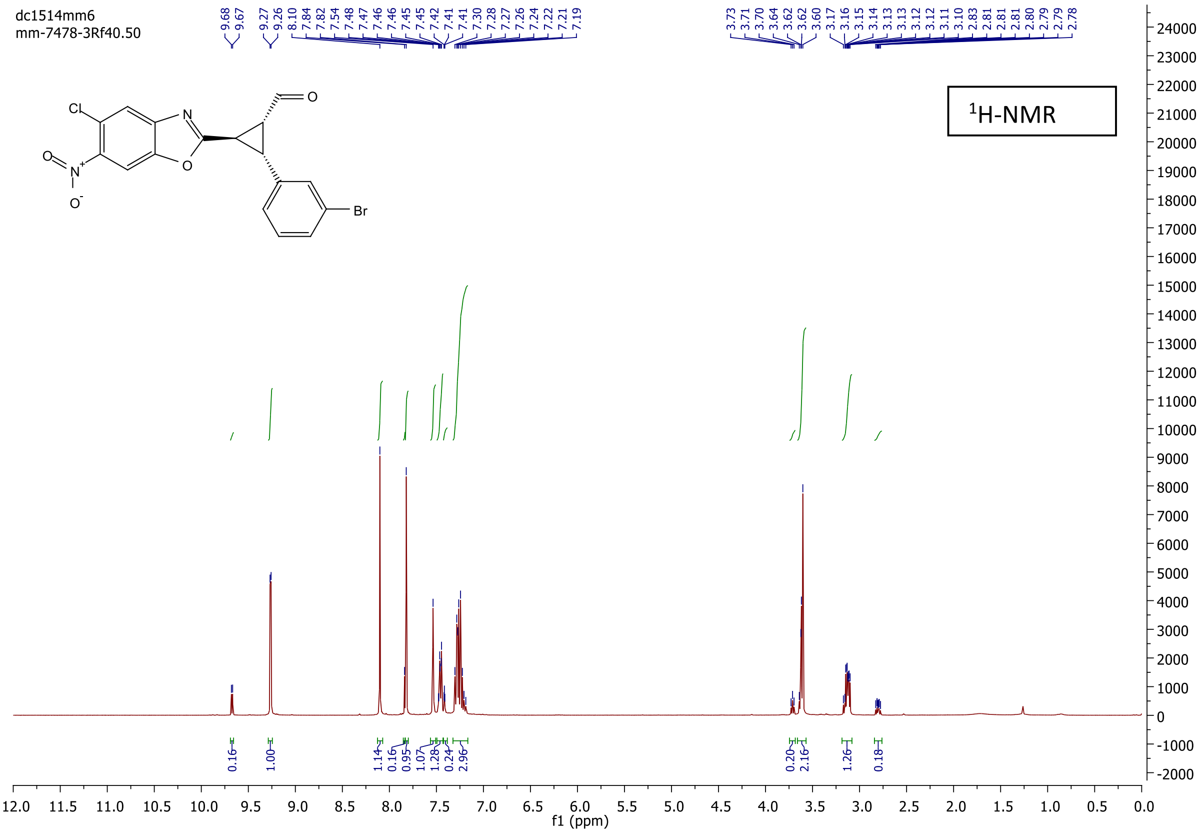

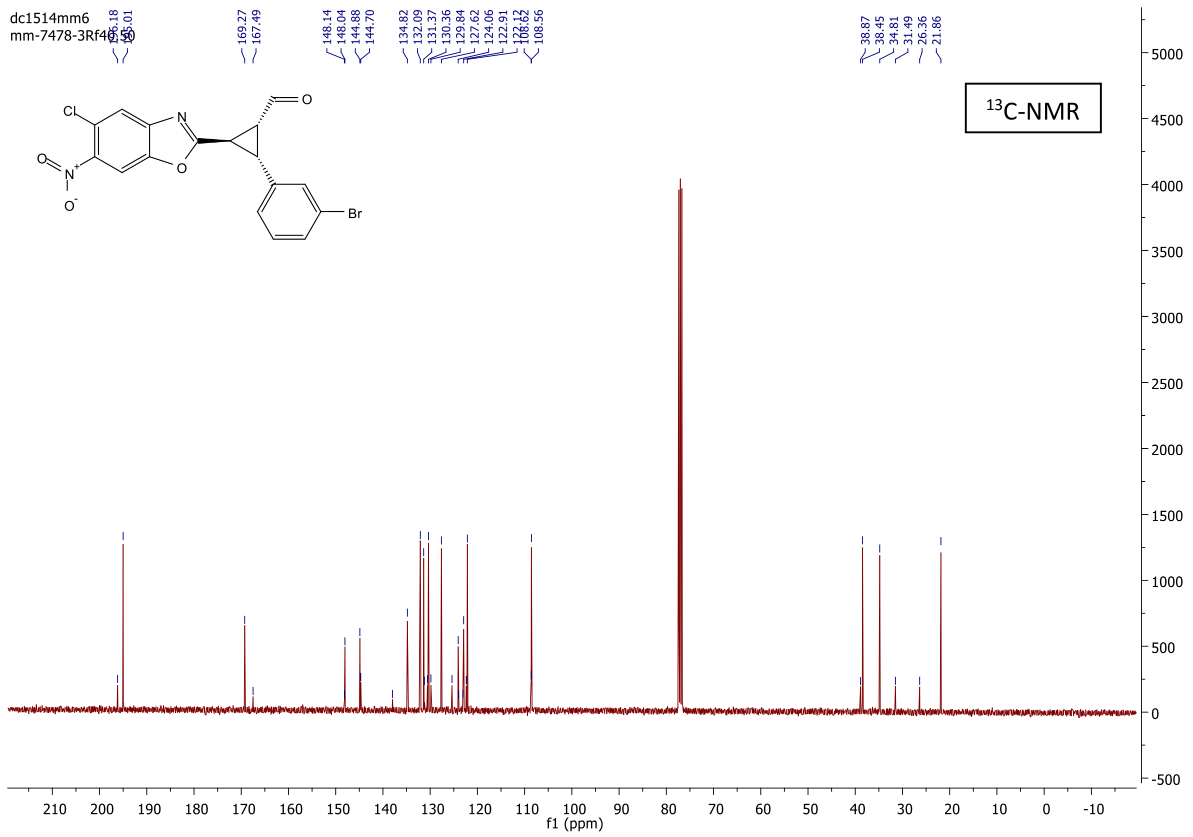
dc1514mm6
mm-7478-3Rf40.50
196.18
195.01
169.27
167.49
148.14
148.04
144.88
144.70
134.82
132.09
131.37
130.36
129.84
127.62
124.06
122.91
122.12
108.62
108.56
38.87
38.45
34.81
31.49
26.36
21.86
Cl
N
O
O
N+
O-
Br
^{13}C-NMR
f1 (ppm)

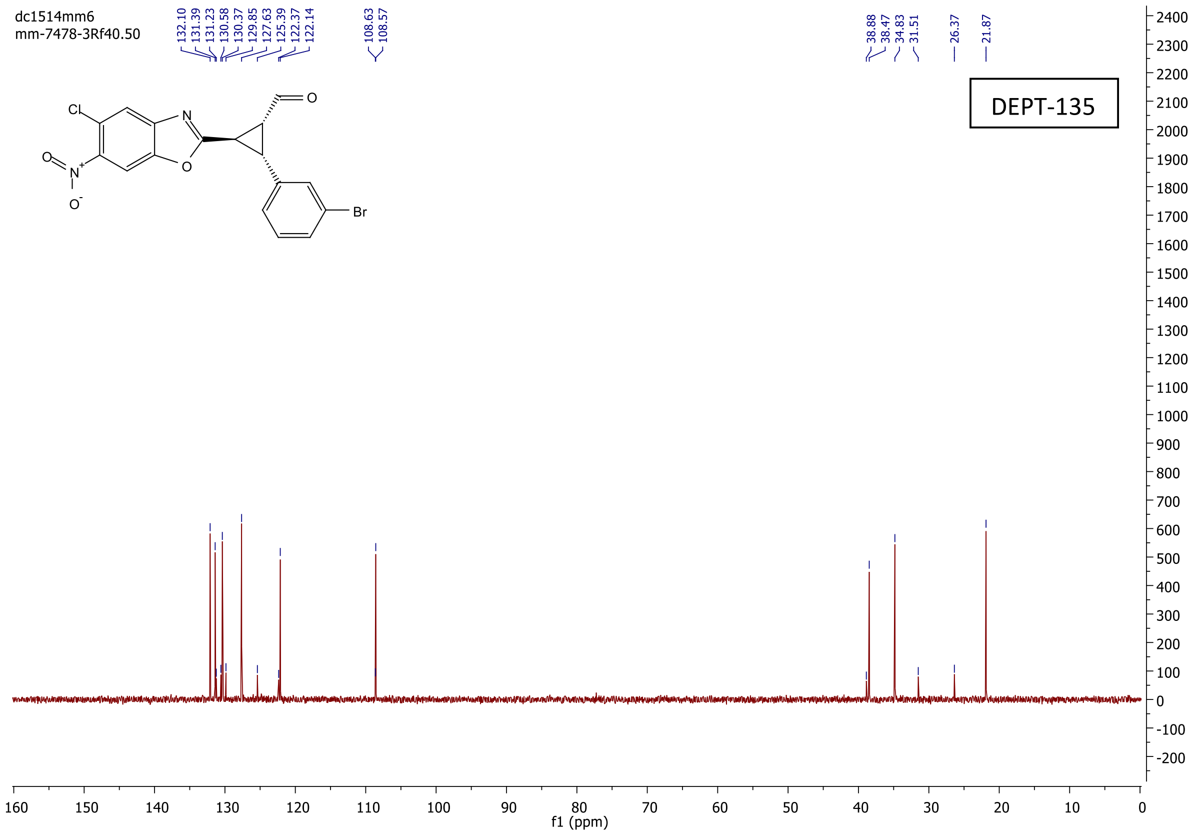
dc1514mm6
mm-7478-3Rf40.50
132.10
131.39
131.23
130.58
130.37
129.85
127.63
125.39
122.37
122.14
108.63
108.57
38.88
38.47
34.83
31.51
26.37
21.87
Cl
N
O
O
N+
O-
Br
DEPT-135
f1 (ppm)
